# Water-soluble β-strand peptidomimetics

**DOI:** 10.1039/d6ob00667a

**Published:** 2026-07-15

**Authors:** Rose C. Bannister, Emily F. Jones, Jonathan E. Ross, Mark E. Light, Graham J. Tizzard, Andrew D. Hamilton, Peter C. Knipe, Sam Thompson

**Affiliations:** a School of Chemistry and the Institute for Life Sciences, University of Southampton Southampton SO17 1BJ UK st3a15@soton.ac.uk; b School of Chemistry and Chemical Engineering, Queen's University Belfast David Keir Building Belfast BT9 5AG UK p.knipe@qub.ac.uk; c Department of Chemistry, University of Oxford Oxford OX1 3TA UK; d UK National Crystallography Service, School of Chemistry, University of Southampton Southampton SO17 1BJ UK; e Department of Chemistry, New York University 100 Washington Square East NY 10003 USA

## Abstract

Mediation of protein–protein interactions with molecules that bind strongly and selectively to one of the partners at the protein interface is a promising therapeutic strategy for myriad diseases. One such approach is the rational design of non-peptidic scaffolds that reproduce the display of amino acid side chains from one face of a secondary structural element. We have previously disclosed proof-of principle syntheses of β-strand mimetics composed of alternating (hetero)aromatic and cyclic urea units, conformationally preorganised through dipolar repulsion in organic solvents, that are in good agreement with the *i*, *i* + 2, and *i* + 4 side-chain vectors of a canonical strand. Here we demonstrate sequence diversity of the approach through the incorporation of hydrophobic and hydrophilic side-chain mimics *via* an improved synthetic route. The scaffold is conformationally preorganised for target binding in aqueous media including buffer, is readily soluble, and thus is suitable for elaboration and deployment against specific protein targets.

## Abstract 2

Non-peptidic β-strand mimetics composed of alternating cyclic ureas and pyridines are conformationally preorganised in aqueous solutions to reproduce the projection of anionic, cationic, and hydrophobic side-chains.

## Graphical abstract

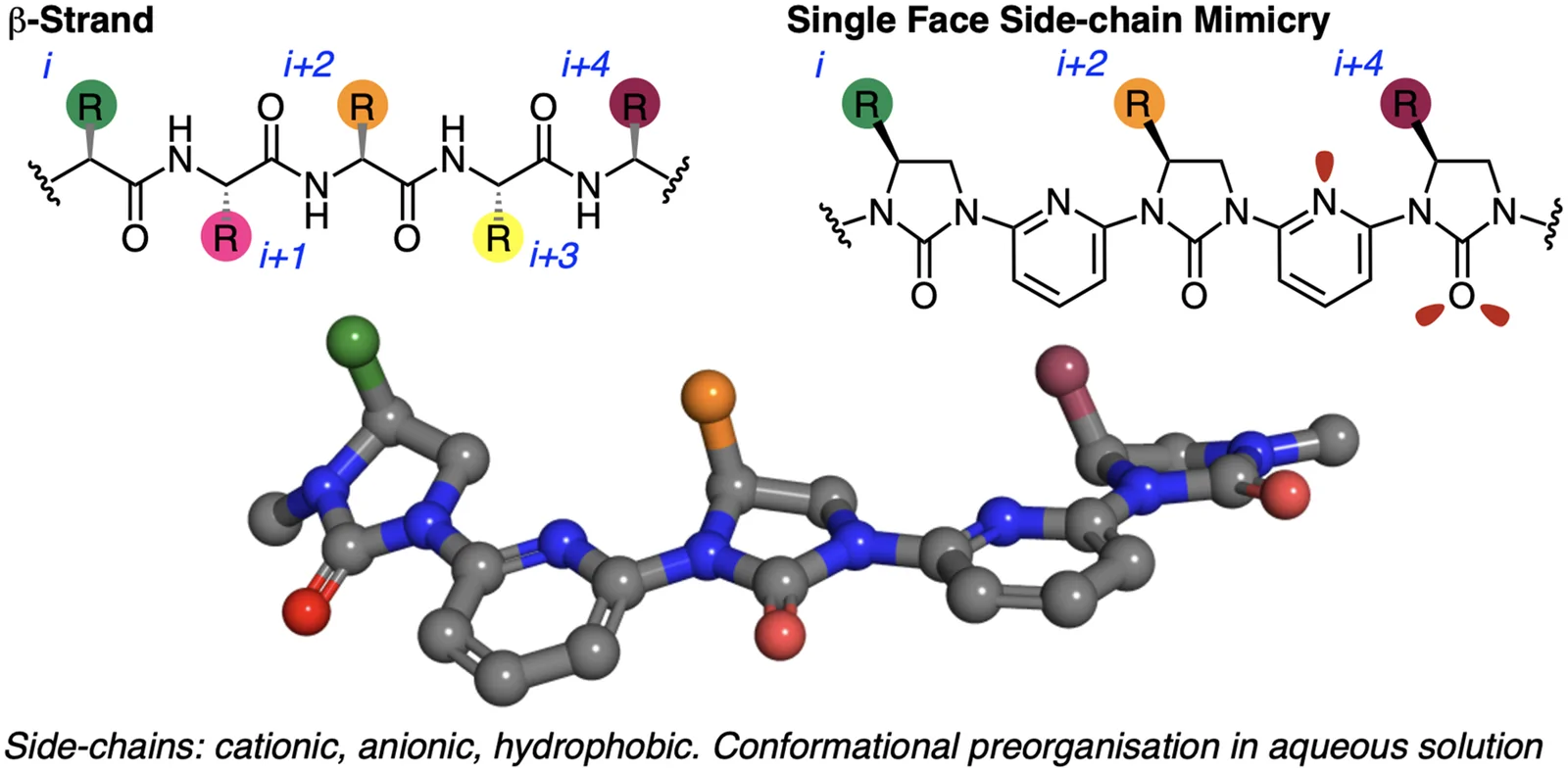

## Introduction

**Fig. 1:**
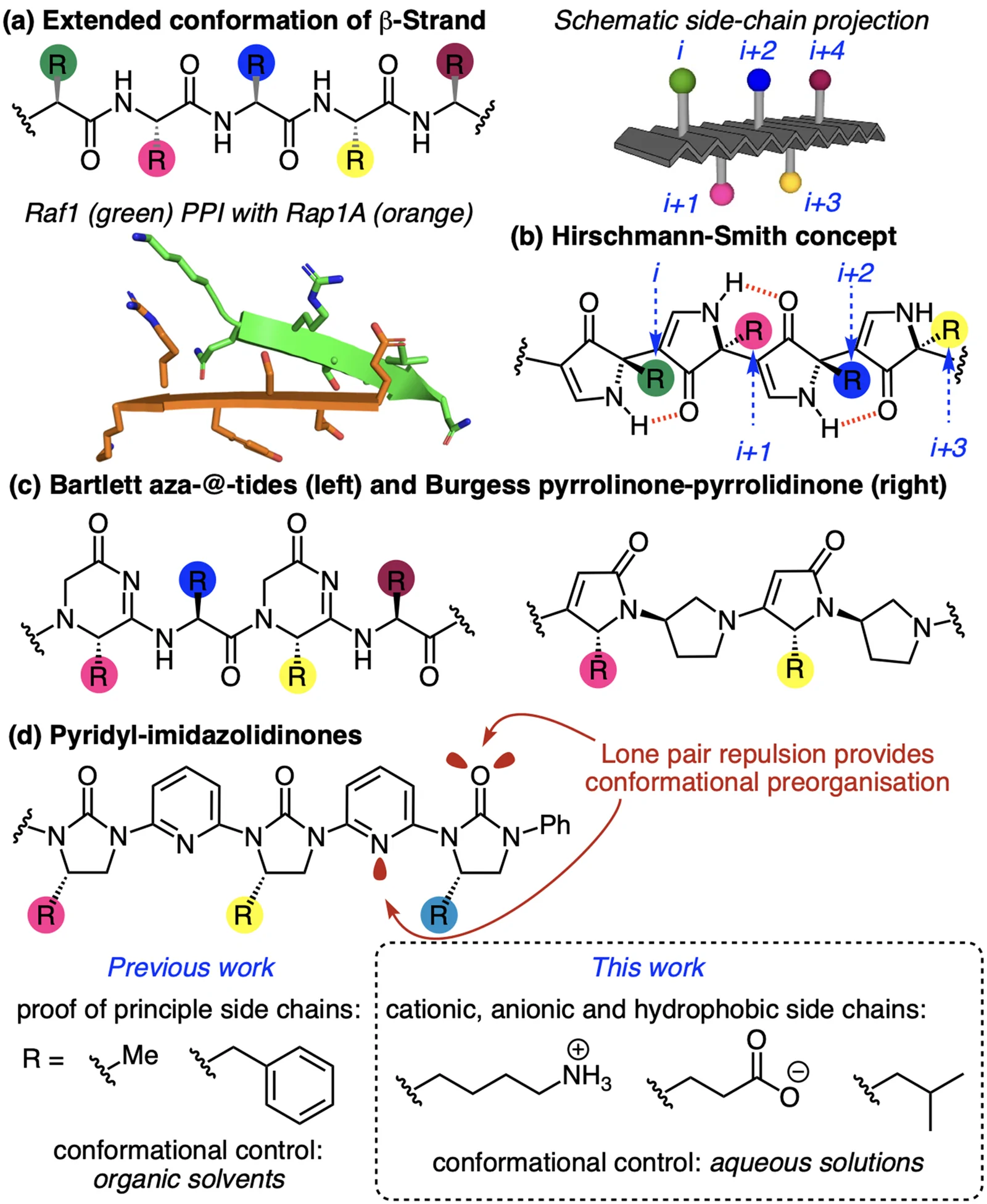
The importance of β-strands at the interface of protein–protein interactions (PPIs) and scaffolds for the mimicry of their side-chains. (a) β-Strand structure, schematic side-chain projection and importance at protein–protein interaction interface: Raf1 (green) with Rap1A (orange; RAS related: PDB 1GUA); (b) Hirschmann–Smith mimetic; (c) Bartlett and Burgess mimetics; (d) pyridyl-imidazolidinones conformationally preorganised by dipolar repulsion. The title work demonstrates side-chain diversity and conformational control in aqueous media (functional group charge states shown at pH 7).

In the search for inhibitors of therapeutically relevant protein–protein interactions (PPIs)^1–3^ we,^4–6^ and others,^7–9^ have rationally designed non-peptidic scaffolds for projection of groups that mimic constellations of side-chains displayed on secondary structural protein elements. Examples of biological targets where a β-strand plays a critical role at the protein interface include Akt-GSK3β ^10^ and Rap1A/Raf1 (Fig. 1a).^11^ Seminal work by Hirschmann and Smith defined a synthetic approach for conformational control providing mimicry of side-chain vectors on both faces of the strand (Fig. 1b).^12^ Other contributions to the field include the work of Bartlett,^13^ and Burgess^14^ (Fig. 1c).

In reproducing the side-chain positions on a single face of the strand – for example, those side-chains found at a PPI interface of interest – simpler designs become possible. The attendant benefits are shorter syntheses and the potential for facile production of focused libraries for inhibitor optimisation. We have previously developed a series of single-sided strand mimetics consisting of amino acid derived cyclic ureas linked through aromatic spacers (Fig. 1d).^15–17^ These proof-of-principle scaffolds include the benefits of iterative and modular syntheses, with demonstration of conformational preorganisation in organic solvents. In other work, we have also exploited the expediency of the approach, and the cyclic urea building block, to prepare contrasting abiotic foldamer architectures.^18,19^ Herein, we report an expansion of our pyridyl-imidazolidinone approach to give amphipathic β-strand mimetics bearing hydrophobic and hydrophilic side-chains and to an investigation of their conformational behaviour in biologically relevant media including buffer.

## Results and discussion

### Synthesis

**Scheme 1:**
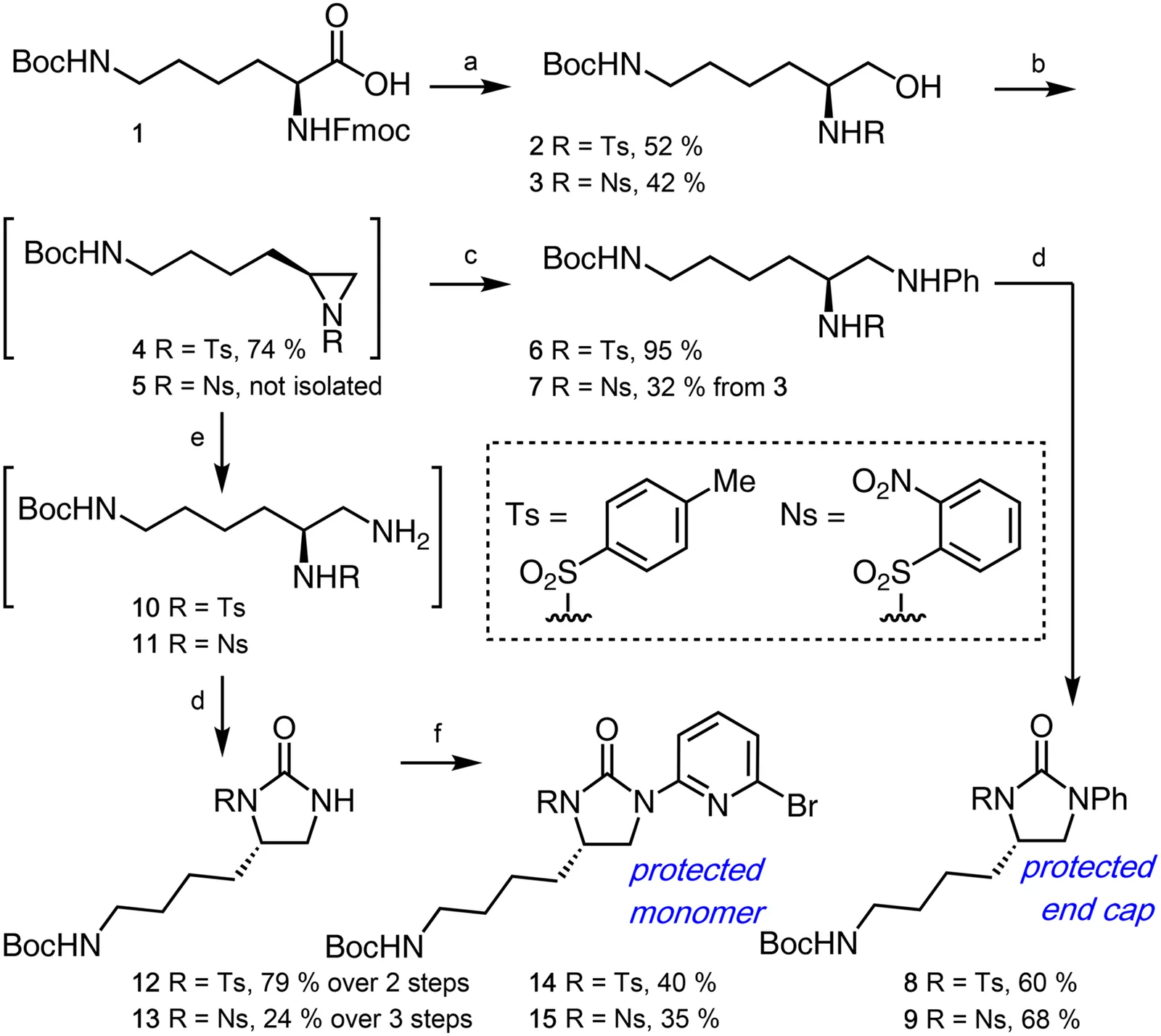
Synthesis of protected lysine mimetic monomers. (a, i) ethyl chloroformate, *N*-methylmorpholine, DME, −15 °C, 5 min, then NaBH_4_ in H_2_O, 1 h; (ii) HNMe_2_ (1 M in EtOH), CH_2_Cl_2_, RT, 1 h; (iii) NEt_3_, RSO_2_Cl, CH_2_Cl_2_, RT, 1 h; (b) DIAD, PPh_3_, THF, 0 °C to RT, 1 h; (c) PhNH_2_ (neat), RT, 16 h; (d) triphosgene, iPr_2_NEt, MeCN, RT, 2 h; (e) NH_3_, MeOH, sealed tube, RT, 4 h; (f) 2,6-dibromopyridine, Pd_2_dba_3_, Xantphos, Cs_2_CO_3_, dioxane, 80 °C, 2 h. Fmoc = 9-fluorenylmethyloxycarbonyl; Boc = *tert*-butoxycarbonyl; Ts = toluenesulfonyl; Ns = 2-nitrobenzenesulfonyl; DME = dimethoxyethane; DIAD = diisopropyl azodicarboxylate; Ph = phenyl; RT = room temperature; Me = methyl; Pr = propyl; Et = ethyl; dba = dibenzylideneacetone; Xantphos = 4,5-bis(diphenylphosphino)-9,9-dimethylxanthene; R = Ts or Ns. Refer to the SI for full experimental procedures.

We first investigated the synthesis of a monomer and the corresponding end cap as a mimic of *N*-ε-Boc-l-lysine. Commercial Fmoc-Lys(Boc)-OH 1 was reduced to the C-terminal alcohol^20^ and the Fmoc protecting group replaced with either tosyl to give 2 (52% over three steps) or 2-nosyl to give 3 (42% over three steps). The synthetic route then followed an adaptation of our previously reported methods: aziridines 4 and 5 were formed *via* a Mitsunobu reaction.^17,21^ Although we successfully isolated 4, given the reported propensity of sulfonyl aziridines to anionic polymerisation,^22^ we did not isolate 5 (see SI for the precautions that were taken in handling sulfonyl aziridines). Opening of aziridines was performed either with ammonia to give 10 and 11, or aniline to give 6 and 7. Diamines 6 and 7 were readily isolable by column chromatography before forming cyclic ureas 8 and 9 as end caps in good yields of 60 and 68% respectively. The increased polarity of 10 and 11, both bearing primary amines, made it preferable to subject the crude mixtures to urea formation with triphosgene, giving 12 and 13. The side-chain protected monomers 14 and 15 were formed in moderate yields *via* Buchwald–Hartwig cross coupling (Scheme 1).

**Scheme 2:**
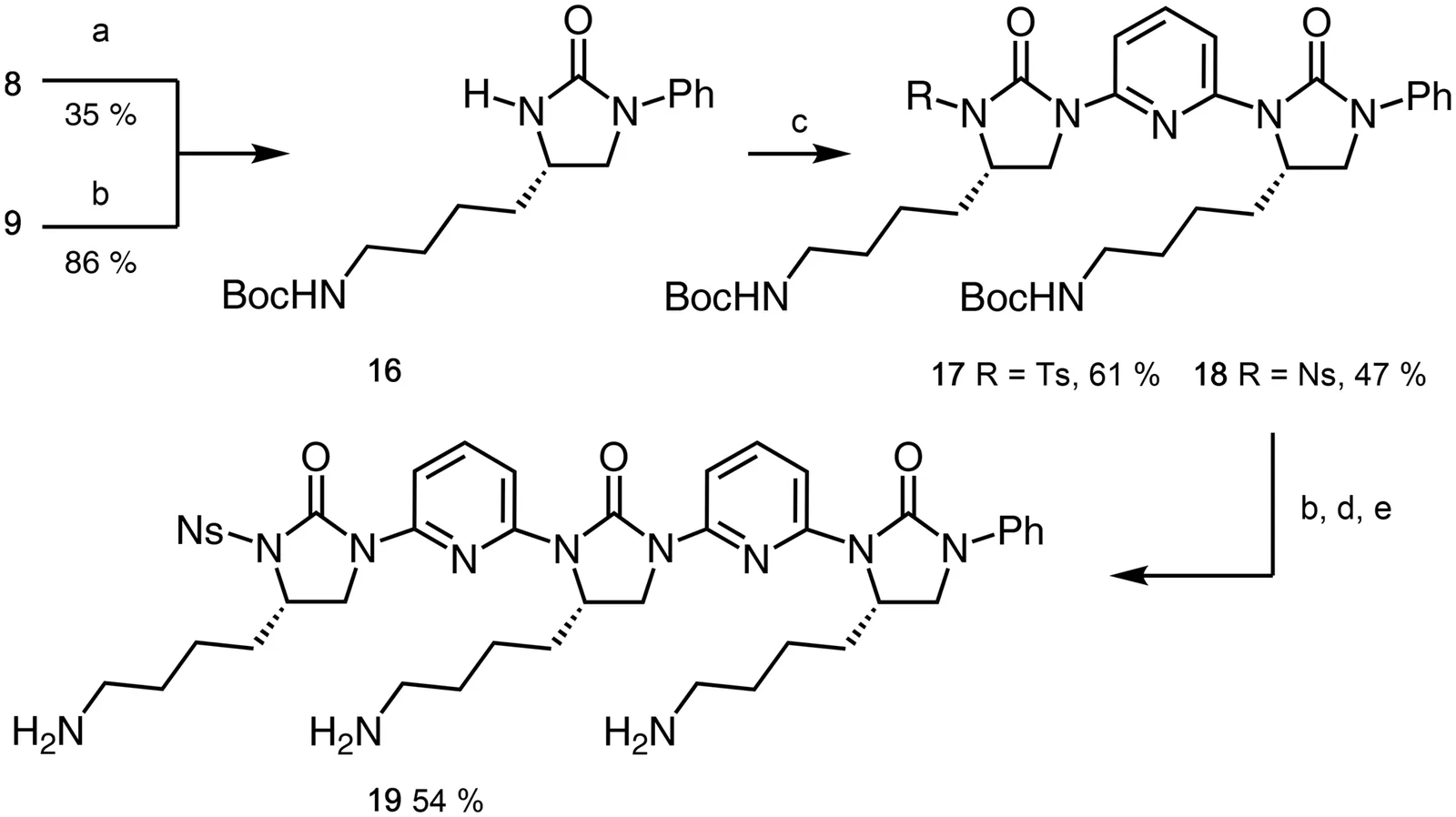
Synthesis of Lys-Lys-Lys mimetic. (a) magnesium powder, MeOH, sonication, RT, 1 h; (b) PhSH, K_2_CO_3_, DMF, 0 °C to RT, 1 h; (c) conditions X with 14 to give 17 or 15 to give 18; (d) conditions X with 15; (e) TFA, CH_2_Cl_2_, RT, 30 min. Conditions X: Pd_2_dba_3_, Xantphos, Cs_2_CO_3_, 1,4-dioxane, 80 °C, 2 h. Boc = *tert*-butoxycarbonyl; Ts = toluenesulfonyl; Ns = 2-nitrobenzenesulfonyl; DMF = *N*,*N*-dimethylformamide, Ph = phenyl; RT = room temperature; Me = methyl; dba = dibenzylideneacetone; Xantphos = 4,5-bis(diphenylphosphino)-9,9-dimethylxanthene; TFA = trifluoroacetic acid. Refer to the SI for full experimental procedures.

Removal of the *N*-sulfonyl groups from either 8 or 9 gave the deprotected end cap 16. On multiple occasions, clean removal of the tosyl group of 8 proved difficult, giving markedly poorer yields (35%) than those for *ortho*-nosyl removal from 9 (86%). Buchwald–Hartwig coupling of deprotected end cap 16 with either tosyl- 14 or nosyl-protected 15 monomer gave protected Lys-Lys mimetics 17 and 18 in moderate to good yield. Given our experience in finding removal of the nosyl group cleaner and higher yielding, when compared to tosyl, 18 was selected for deprotection, cross-coupling with monomer 15, and finally side-chain Boc group removal to give Lys-Lys-Lys mimetic 19 (Scheme 2).

**Scheme 3:**
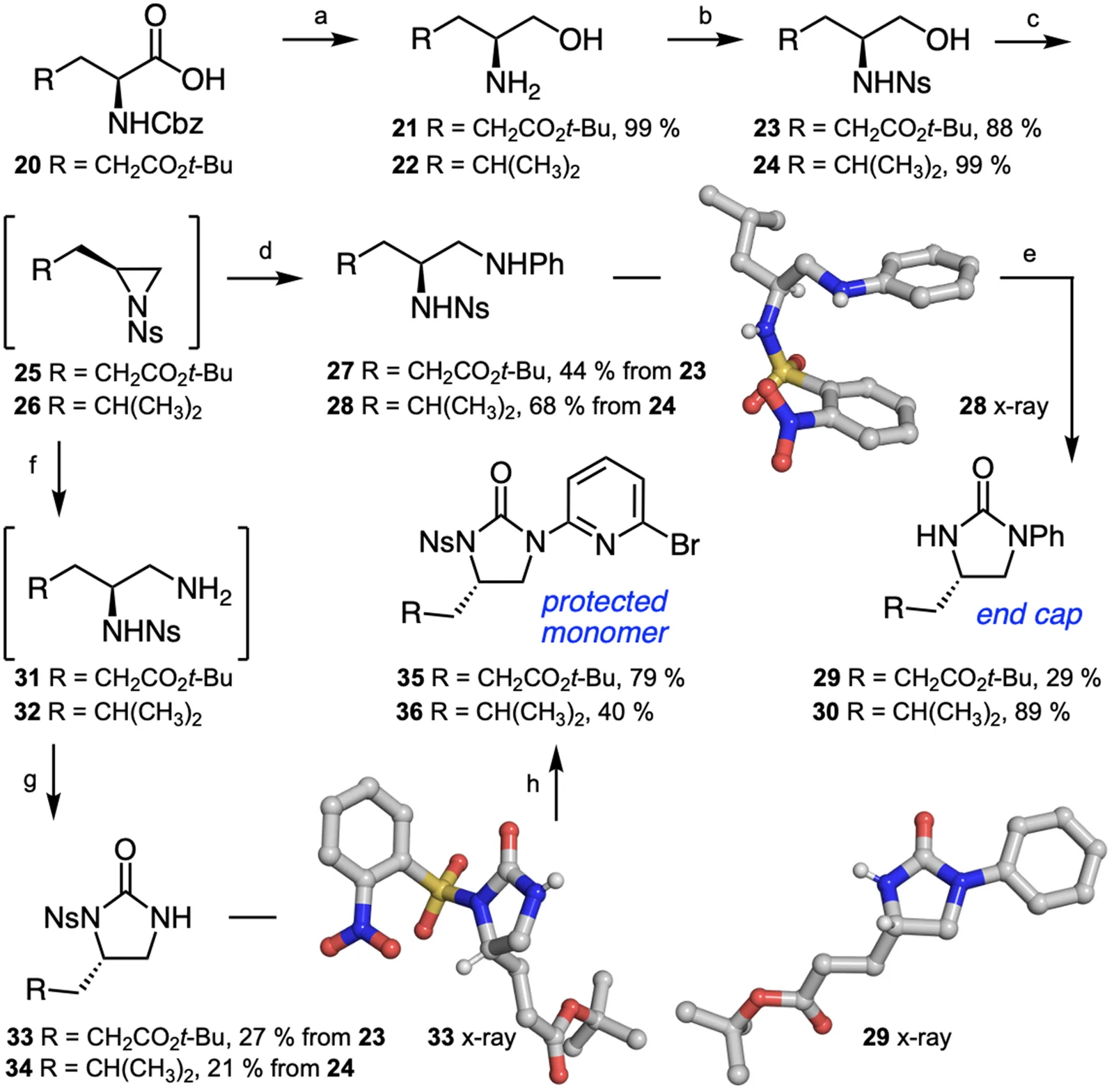
Synthesis of lysine and glutamic acid mimetic monomers. (a, i) ethyl chloroformate, *N*-methylmorpholine, DME, −15 °C, 5 min, then NaBH_4_ in H_2_O, 1 h; (ii) Pd/C, H_2_, MeOH, RT, 2 h; (b) NEt_3_, NsCl, CH_2_Cl_2_, RT, 18 h; (c, i) Ms_2_O, DMAP, pyridine, CH_2_Cl_2_, 0 °C to RT, 30 min; (ii) K_2_CO_3_, THF, 70 °C, 1 h; (d) PhNH_2_ (neat), RT, 18 h; (e, i) triphosgene, iPr_2_NEt, MeCN, RT, 4 h; (ii) PhSH, K_2_CO_3_, DMF, 0 °C to RT, 2 h; (f) aq. NH_4_OH, MeOH, RT, 2 h; (g) triphosgene, iPr_2_NEt, MeCN, RT, 2 h; (h) 2,6-dibromopyridine, Pd_2_dba_3_, Xantphos, Cs_2_CO_3_, 1,4-dioxane, 80 °C, 2 h. Cbz = benzyl carbamate; Ns = 2-nitrobenzenesulfonyl; DME = dimethoxyethane; Ms = methanesulfonyl; DMAP = *N*,*N*-dimethylpyrid-4-amine; THF = tetrahydrofuran; Ph = phenyl; RT = room temperature; Me = methyl; Pr = propyl; Et = ethyl; dba = dibenzylideneacetone; Xantphos = 4,5-bis(diphenylphosphino)-9,9-dimethylxanthene. Refer to the SI for full experimental procedures.

To explore the amino acid scope of the synthetic method, and thus to validate the approach for targeting specific PPIs, we chose leucine, as a more sterically demanding side-chain than already studied alanine and phenylalanine, and glutamic acid as a charge counterpart of lysine. Commercial *Z*-Glu(O*t*-Bu)–OH 20 was *C*-terminally reduced *via* the intermediacy of the mixed anhydride, in analogous fashion to that of protected lysine 1, before hydrogenolysis of the *N*-terminal protecting group to give 21. The two-step sequence proceeded reproducibly in excellent yields on a multi-gram scale. l-Leucinol 22, is commercially available, obviating the need for *C*-terminal reduction of the corresponding amino acid. Given the difficulties in removing *N*-tosyl protecting groups during the lysine mimetic synthesis (Scheme 1), we added nosyl groups to Glu and Leu derivates 21 and 22 to give sulfonamides 23 and 24 respectively (Scheme 3).

In our previous syntheses, primary alcohol activation of the amino alcohol and intramolecular displacement to the aziridine was performed under Mitsunobu conditions, for example 4 and 5 from 2 and 3 respectively (Scheme 1). Whilst yields of the aziridines themselves, and the diamines resulting from their nucleophilic opening, were generally satisfactory, it was sometimes laborious to isolate the target compounds from the 1,2-diisopropylhydrazinedicarboxylate byproduct. To avoid such difficulties, we formed mesylates of alcohols 23 and 24, based on the method of Jacobsen,^23^ which ring-closed cleanly under basic conditions to the *N*-nosyl aziridines 25 and 26. Nucleophilic opening at the least hindered end of the aziridines with either ammonia to give 31 and 32, or aniline to give 27 and 28, proceeded regioselectively. Urea formation from each of diamines 27 and 28 followed by *N*-nosyl deprotection with phenylthiolate (formed *in situ*) provided the Glu and Leu end caps 29 and 30. *N*-Nosyl protected monomers 35 and 36 were formed in moderate to very good yields *via* urea formation from 31 and 32 to give 33 and 34, followed by Buchwald Hartwig coupling. Notable features of these syntheses are the avoidance of hydrazine byproducts and the ability to minimise stepwise purification – for example, urea 33 is formed over five synthetic steps (including the mesylation) from commercially available l-leucinol 22 with two chromatographic operations.

**Scheme 4:**
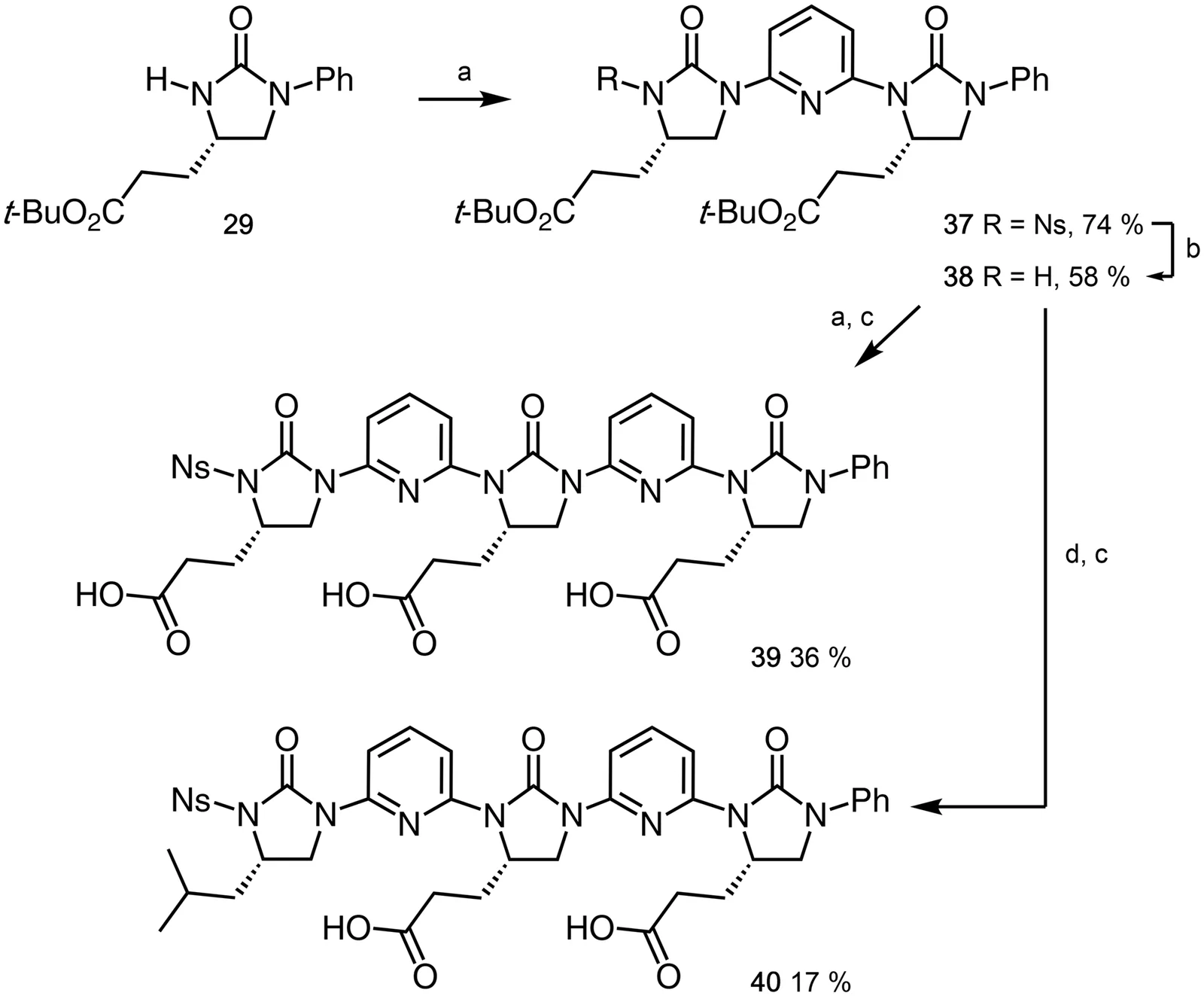
Synthesis of Glu-Glu-Glu and Leu-Glu-Glu mimetics. (a) conditions X with 35; (b) PhSH, K_2_CO_3_, DMF, 0 °C to RT, 1 h; (c) TFA, CH_2_Cl_2_, RT, 1 h; (d) conditions X with 36. Conditions X: Pd_2_dba_3_, Xantphos, Cs_2_CO_3_, 1,4-dioxane, 80 °C, 2 h. Ns = 2-nitrobenzenesulfonyl; Ph = phenyl; RT = room temperature; DMF = *N*,*N*-dimethylformamide; dba = dibenzylideneacetone; Xantphos = 4,5-bis(diphenylphosphino)-9,9-dimethylxanthene; TFA = trifluoroacetic acid. Refer to the SI for full experimental procedures.

The connectivity and stereochemical fidelity of the compounds formed *via* this revised synthetic route is supported by single crystal X-ray diffraction structures of leucine derived diamine 28 and glutamic acid derived ureas 29 and 33 (Scheme 3 and SI). In contrast to hydrophilic tri-Lys mimetic 19, which is expected to exist as the triply charged cation at physiologically relevant pH, we set out to synthesise the tri-Glu analogue 39 and an amphiphilic Leu-Glu-Glu mimic 40. Side-chain protected Glu end-cap 29 was iteratively cross coupled with two Glu monomers 35, with an intermediate *N*-nosyl deprotection step (37 to 38), to give Glu-Glu-Glu mimic 39 following global side-chain deprotection under acidic conditions. Interception of Glu-Glu dimer 38, cross coupling with Leu monomer 36, and deprotection with TFA, provided trimer 40 (Scheme 4).

### Conformational analysis

**Fig. 2:**
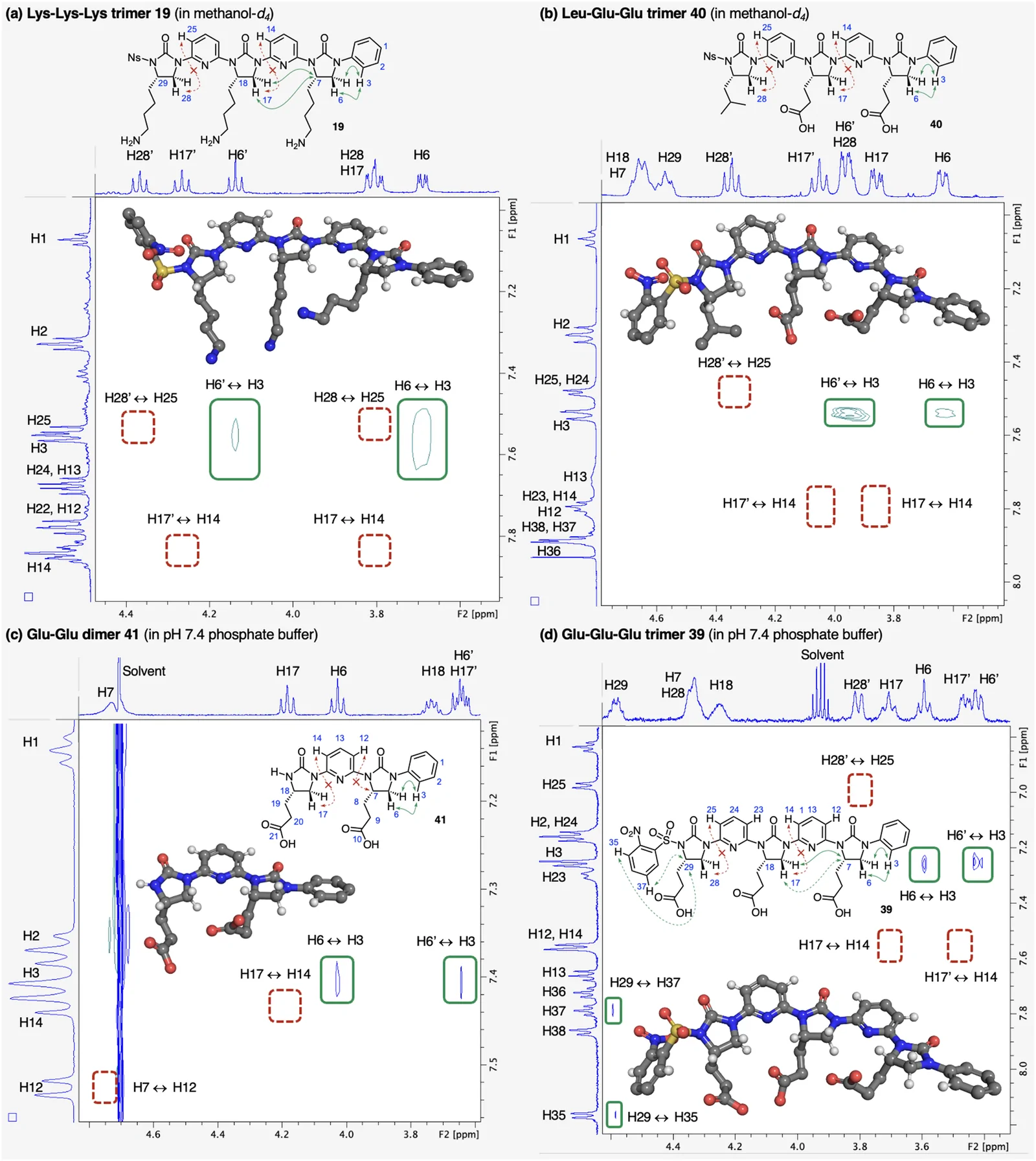
Solution phase conformational analysis of non-peptidic β-strand mimetics. Selected regions of ROESY/NOESY NMR spectrum with arrows showing diagnostic nOes. Observed (green, solid = strong, dashed = weak) and absent (red, dashed). Molecular models of low energy conformers (Spartan MMFF) with NMR constraints applied. (a) Lys-Lys-Lys trimer 19 (600 MHz, ROESY, spin lock = 400 ms), (b) Leu-Glu-Glu trimer 40 (500 MHz, ROESY, spin lock = 400 ms), (c) Glu-Glu dimer 41 (NOESY, 500 MHz, mixing time = 800 ms), (d) Glu-Glu-Glu trimer 39 (NOESY, 600 MHz, mixing time = 800 ms). Methanol-*d*_4_ (a and b), pH 7.4 phosphate buffer (c and d), 12 mM, 298 K. Refer to the SI for complete spectra.

The conformational properties of Lys-Lys-Lys mimetic 19 was explored in methanol-*d*_4_. The ROESY spectrum provided more informative nOes than the NOESY spectrum,^24^ with the former showing the expected through space correlations between both urea H6 and H6′ with end-cap phenyl H3, whereas correlations between diastereotopic urea H28 and H28′ with pyridyl H25, or between urea H17/H17′ and pyridyl H14, were absent. These data are consistent with significant weighting of the ensemble towards the desired conformation in which lone-pair repulsion places urea carbonyls *anti* to pyridine nitrogens (Fig. 2a). Further support for the populational weighting of this desired conformer comes from cross-strand correlations. However, the separation of the Hs on adjacent ureas places them at the limit of detection owing to the 1/*r*^6^ nOe dependence. Accordingly, some correlations are observed, and some are not. H7 ↔ H17, and H7 ↔ H17′ are observed, while H18 ↔ H28/H28′ are not observed (see SI). Analogous conformational behaviour was observed for Leu-Glu-Glu mimetic 40 in methanol-*d*_4_. The ROESY spectrum showed urea (H6 & H6′) correlation with end-cap phenyl (H3) and the absence of urea correlations with the corresponding pyridine hydrogens, *i.e.*, H14 ↔ H17/H17′ and H25 ↔ H28/H28′ were absent (Fig. 2b). To establish representative solution phase conformational behaviour in more competitive and physiologically relevant media, a small quantity of Glu-Glu dimer 38 was side-chain deprotected with TFA to give diacid 41. This di-carboxylic acid, along with Glu-Glu-Glu trimer 39, were dissolved in pH 7.4 phosphate buffer and subjected to NOESY experiments. The same pattern of cross peaks was observed as for mimics 19 and 40 in methanol-*d*_4_, thus consistent with the desired conformational preorganisation. For dimeric mimic 41, ^1^H spectral dispersion was sufficient to assign diagnostic urea and pyridine Hs, thus allowing confirmation of the absence of H14 ↔ H17, and H7 ↔ H12 in the NOESY spectrum despite the observed transfer of polarisation from H6 to H3 (Fig. 2c). For Glu-Glu-Glu 39, the global absence of pyridine H ↔ urea H correlations is consistent with the desired conformation. In addition to end-cap (H3) ↔ urea (H6) and nosyl (H35 & H37) ↔ urea (H29) correlations, support for the desired conformation came from cross-strand H7 ↔ H17 and tentative H7 ↔ H17′ correlations. H18 ↔ H28/H28′ was not observed (Fig. 2d, refer to the SI for full spectra).

**Fig. 3:**
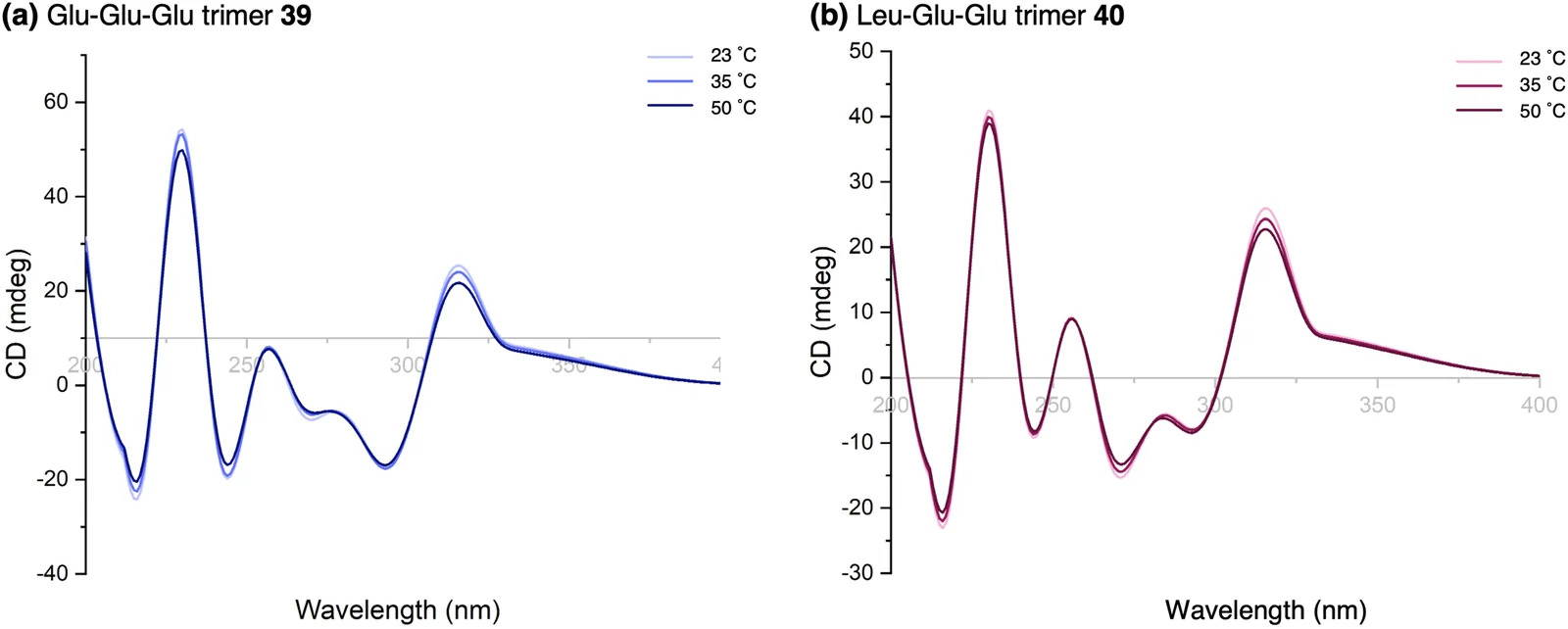
Variable temperature circular dichroism solution phase conformational analysis of (a) Glu-Glu-Glu trimer 39 (1.15 mM), and (b) Leu-Glu-Glu trimer 40 (2.82 mM) in acetonitrile.

The solution phase conformational behaviour of three-residue mimics Glu-Glu-Glu 39 and Leu-Glu-Glu 40 were further probed by variable temperature circular dichroism in acetonitrile. Glu-Glu-Glu 39 exhibited strong minima at 220, 245 and 285 nm, while amphiphilic Leu-Glu-Glu 40 displayed strong minima at 220, 245, 270 and 290 nm. Both mimetics displayed strong maxima at 200, 230 and 320 nm. The spectral signatures of 39 and 40 changed little over the temperature range of 23 to 50 °C, which is consistent with minimal perturbation of conformational ensemble (Fig. 3). Moreover, they are consistent with the CD spectra of previously studied and conformationally well-characterised three-residue strand mimetics with backbones composed of alternating pyridyl and cyclic ureas. Measured in acetonitrile, these have minima at 220, 245, 270, 295 and maxima at 200, 230, 255, 280 and 315 nm.^21^

## Conclusions

We have demonstrated side-chain generality in modular syntheses of non-peptidic strand mimetics and the preservation of conformational preorganisation in aqueous media. Future work will focus on the synthesis of appropriately functionalised mimetics for the mediation of therapeutically relevant PPIs.

## Conflicts of interest

There are no conflicts to declare.

## Supplementary Material

OB-024-D6OB00667A-s001

OB-024-D6OB00667A-s002

## Data Availability

Supplementary information (SI) is available it containing experimental procedures; ^1^H, ^13^C and selected NOESY and ROESY spectral; HRMS; IR and optical rotation values; crystallographic data in CIF or other electronic format. See DOI: https://doi.org/10.1039/d6ob00667a. CCDC 2221030 (28), 2546212 (29) and 2546213 (33) contain the supplementary crystallographic data for this paper.^25*a*–*c*^
