# Supplementary material for: Water-soluble β-strand peptidomimetics
Source: Org Biomol Chem. 2026 Jul 15. Online ahead of print. doi: 10.1039/d6ob00667a (PMC13397914; doi:10.1039/d6ob00667a)
Supplement: OB-OLF-D6OB00667A-s001 [file OB-OLF-D6OB00667A-s001.pdf]

Supplementary Information (SI) for Organic & Biomolecular Chemistry.
This journal is © The Royal Society of Chemistry 2026

Supporting information for

Water Soluble β-Strand Peptidomimetics

Rose C. Bannister, [a] Emily F. Jones, [a] Jonathan E. Ross, [c] Mark E. Light, [d] Graham J. Tizzard, [d] Andrew D. Hamilton, [c,e] Peter C. Knipe [*b,c] and Sam Thompson [*a,c]

School of Chemistry and Chemical Engineering, Queen's University Belfast, David Keir Building, Belfast, BT9 5AG, U.K.
p.knipe@qub.ac.uk

School of Chemistry, University of Southampton, Southampton, SO17 1BJ, U.K.
st3a15@soton.ac.uk

Table of contents

1. Synthesis ---- 2
1.1 General Experimental ---- 2
Solvents and Reagents ---- 2
Chromatography ---- 2
Spectroscopy ---- 2
1.2 Experimental Procedures and Characterisation Data ---- 3
2. NMR Spectra of Synthetic Compounds ---- 36
3. Solution-phase Conformational Analysis ---- 76
4. X-Ray Crystallography ---- 88
4.1 Experimental ---- 88
4.2 Results ---- 89
4.3 Molecular Structures ---- 90
5. References ---- 92

1. Synthesis

1.1 General Experimental

Solvents and Reagents

Reactions were carried out under an argon atmosphere in oven-dried glassware unless otherwise stated. Standard inert atmosphere techniques were used in handling all air and moisture sensitive reagents. Tetrahydrofuran (THF), dichloromethane (DCM), *N*,*N*-dimethylformamide (DMF) and methanol (MeOH) were anhydrous: either dried on an MB-SPS-800 solvent purification system or purchased in Sureseal™, or equivalent, bottles. Other solvents and reagents were used directly as received from commercial suppliers. Petrol refers to the fraction of petroleum ether that boils in the range 40-60 °C. All aqueous solutions were saturated unless specified otherwise.

Chromatography

Flash column chromatography was carried out using Merck 60 silica gel. Thin-layer chromatography was carried out using Merck Kieselgel 60 F254 (230-400 mesh) fluorescent treated silica, visualized under UV light (254 nm) or by staining with aqueous potassium permanganate solution.

High performance liquid chromatography was performed using a Waters 1525 pump, 2707 autosampler, and 2849 detector. Phenomonex Luna columns (250 mm long, 5 µm beads, C18 reverse-phase medium) were used for HPLC separations. Analytical HPLC was run using 1 mL min^{-1} flow through a 4.6 mm diameter column. Sample injections for analytical runs consisted of 40 µL of a 1 $mg.mL^{-1}$ sample solution. Semi-preparative HPLC was run using 10$mL.min^{-1}$ flow through a 21.1 mm diameter column. Sample injections for semi-preparative runs consisted of 500 µL of solution containing no more than 50 mg of sample. HPLC solvents were degassed by sonication for 30 min and contained 0.1 % v/v TFA.

Spectroscopy

^{1}H and ^{13}C NMR spectra were recorded using a Bruker spectrometer (400, 500 or 600 MHz) running TopSpin™ software and are quoted in ppm for measurement against residual solvent peaks. Compound names are those generated by ChemBioDraw™ (CambridgeSoft) following IUPAC nomenclature. Chemical shifts (δ) are given in parts per million (ppm) and coupling constants (*J*) are given in Hertz (Hz). The ^{1}H NMR spectra are reported as follows: δ (number of protons, multiplicity, coupling constant). Multiplicity is abbreviated as follows: s = singlet, d = doublet, t = triplet, q = quartet, quint. = quintet, m = multiplet, br = broad.

IR spectra were recorded either on a Bruker Tensor 27 FT-IR or a Thermo Scientific Nicolet is5 spectrometer from a thin film deposited onto a diamond ATR module. 16 scans were acquired per spectrum in the region 500 – 4000 cm^{-1} with a resolution of 4 wavenumber per cm^{-1}. Only selected maximum absorbances (ν_{max}) of the most intense peaks are reported (cm^{-1}).

High-resolution mass spectra were acquired by the internal service of the Chemistry Department at the University of Southampton or University of Oxford using either a Bruker MicroTof (ESI), an Agilent 7200 Accurate Mass Q-TOF GC/MS with an MSD Direct Inlet Probe (ammonia CI), a MaXis (Bruker Daltonics, Bremen, Germany) time of flight (TOF) mass spectrometer or a solariX (Bruker Daltonics, Bremen, Germany) mass spectrometer equipped with a 4.7 T magnet and FT-ICR cell.

Meting points were recorded using either a Leica Galen III hot-stage microscope apparatus or a Stuart (Bibby Scientific Limited) SMP20 machine and are reported uncorrected in degrees Celsius (˚C).

Circular dichroism spectra were acquired using an Applied Photophysics qCD Chirascan™-plus spectrometer.

Optical rotations were recorded using either a Perkin Elmer 341 or Optical Activity POLAAR 2001 polarimeter and are reported in degrees using concentrations in g.100 mL^{-1}.

1.2 Experimental Procedures and Characterisation Data

General procedure A: *C*-terminal Carboxylic Acid Reduction

According to a literature procedure,[1] ethyl chloroformate (1.10 eq.) was added to a stirring solution of acid (1.00 eq.) and *N*-methylmorpholine (1.05 eq.) in dimethoxyethane (0.1 M) at -15 °C. The reaction mixture was stirred for 5 min and the resultant slurry filtered and washed with diethyl ether (20 $mL.mmol^{-1}$). The filtrate was cooled to -15 °C and $NaBH_4$ (3.00 eq.) in water (1 M) was added dropwise. The reaction flask was left open to allow for evolution of gas and upon cessation of effervescence ten times the initial volume of water was added. The reaction was warmed to room temperature and stirred for 1 h, at which point the reaction was further diluted with water (20 $mL.mmol^{-1}$) and extracted with dichloromethane (3 x 10 $mL.mmol^{-1}$). The combined organic layers were dried and concentrated *in vacuo*.

General procedure B: *N*-terminal Fmoc Deprotection

To a stirring solution of Fmoc protected amine (1.0 eq.) in dichloromethane (0.1 M) was added a solution of dimethylamine in ethanol (1 M, final ratio *v*:*v*, 1:1). The reaction was stirred for 1 h at room temperature and concentrated *in vacuo*.

General procedure C: *N*-terminal Tosylation

To a stirring solution of amine (1.0 eq.) in dichloromethane (0.1 M) was added triethylamine (3.0 eq.) and *p*-tosyl chloride (2.0 eq.). The reaction was stirred at room temperature for 1 h and subsequently diluted with dichloromethane (0.05 M). The solution was partitioned with saturated aqueous ammonium chloride (10 $mL.mmol^{-1}$) and extracted with dichloromethane (3 x 10 $mL.mmol^{-1}$). The combined organic layers were dried over magnesium sulfate, filtered and the reaction concentrated *in vacuo*.

General procedure D: *N*-terminal Nosylation

To a stirring solution of amine (1.0 eq.) in dichloromethane (0.10 M) was added triethylamine (2.0 eq.) and *ortho*-nosyl chloride (2.0 eq.). The reaction was stirred at room temperature for 16 h and subsequently diluted with dichloromethane (0.05 M). The solution was partitioned with saturated aqueous ammonium chloride (10 $mL.mmol^{-1}$) and extracted with dichloromethane (3 x 10 $mL.mmol^{-1}$). The combined organic layers were dried over magnesium sulfate, filtered and the organic layers concentrated *in vacuo*.

General procedure E: Aziridination *via* Mitsunobu Reaction

Triphenylphosphine (1.5 eq.) and DIAD (1.5 eq.) were added to a stirring solution of alcohol (1.0 eq.) in tetrahydrofuran (0.1 M). The reaction was stirred at room temperature for 30 min and concentrated *in vacuo*.

General procedure F: Aziridination *via O*-Mesylation and Displacement

According to a literature procedure,[5] N-sulfonyl amino alcohol (0.26 mmol), methanesulfonic anhydride (1.5 eq.) and 4-DMAP (0.1 eq.) were dissolved in CH_2Cl_2 (4 M) before pyridine (2 eq.) was added and stirred at rt for 30 minutes. The reaction mixture was washed with 10% $CuSO_4$ (10 $mL.mmol^{-1}$) and extracted with CH_2Cl_2 (3 x 10 $mL.mmol^{-1}$). The organic layers were concentrated *in vacuo* and the residue redissolved in THF (4.0 M) and K_2CO_3 solution (1 $mL.mmol^{-1}$) added before being heated to 70 °C for 1 h. Water (40 $mL.mmol^{-1}$) was added to the reaction mixture followed by extraction with CH_2Cl_2 (3 x 10 $mL.mmol^{-1}$).

General procedure G: Aziridine Ring-opening with Aniline

Aziridine (1.0 eq.) was dissolved in aniline (1.0 M) and the reaction stirred at 80 °C for 2 h and concentrated *in vacuo*.

General procedure H: Aziridine Ring-opening with Ammonia

Aziridine (1.0 eq.) was dissolved in methanol (0.1 M) in a sealed tube. At 0 °C, ammonia gas was bubbled through the solution for 20 min, before the tube was sealed, heated to 80 °C and stirred for 2 h. The reaction mixture was transferred to a round-bottomed flask and the reaction concentrated *in vacuo*.

General procedure I: Urea Formation

To a stirring solution of diamine (1.0 eq.) and *N*,*N*-diisopropylethylamine (1.1 eq.) in acetonitrile (0.25 M) was added triphosgene (0.4 eq., 0.25 M in acetonitrile) by syringe pump over the course of 1 h. The reaction was stirred for a further 30 min, diluted with water (0.05 M) and extracted with dichloromethane (3 x 10 $mL.mmol^{-1}$). The combined organic layers were dried over magnesium sulfate, filtered and the reaction concentrated *in vacuo*.

General procedure J: One-pot Aziridine Ring-opening with Ammonia and Urea Formation

N-sulfonyl aziridine (4.81 mmol) was stirred in aqueous ammonium hydroxide (22%, 3.3 $mL.mmol^{-1}$) and MeOH (10 $mL.mmol^{-1}$) for 2 h. The reaction mixture was diluted with H_2O (10 $mL.mmol^{-1}$) and extracted with CH_2Cl_2 (3 x 10 $mL.mmol^{-1}$). The organic layers were concentrated *in vacuo* before being redissolved in MeCN (0.5 M). DIPEA (1.1 eq.) was added followed by cautious addition of a solution of triphosgene (0.4 eq.) in MeCN (1 M) and the mixture stirred for 2 h. The reaction mixture was washed with H_2O (10 $mL.mmol^{-1}$) and extracted with CH_2Cl_2 (3 x 10 $mL.mmol^{-1}$).

General procedure K: *N*-Tosyl Deprotection

Magnesium powder (2.0 eq.) was added to tosyl protected amine (1.0 eq.) in methanol. The solution was sonicated for 1 h, diluted with water (10 $mL.mmol^{-1}$) and extracted with dichloromethane (3 x 10 $mL.mmol^{-1}$). The combined organic layers were dried over magnesium sulfate, filtered and the reaction concentrated *in vacuo*.

General procedure L: Buchwald-Hartwig Coupling

To a stirring solution of amine (1.0 eq.) in 1,4-dioxane was added caesium carbonate (2.0 eq.), $Pd_2(dba)_3$ (0.05 eq.) and Xantphos (0.15 eq.). The mixture was degassed with argon for 15 min before the addition of aryl bromide (1.2 eq.). The reaction was heated to 80 ˚C and stirred for 2 h. Upon completion the mixture was diluted with water (10 $mL.mmol^{-1}$) and extracted with dichloromethane (3 x 10 $mL.mmol^{-1}$). The combined organic layers were dried over magnesium sulfate, filtered and the reaction concentrated *in vacuo*.

General procedure M: *N*-Nosyl deprotection

To a stirring solution of *N*-nosylurea (1.0 eq.) in DMF (0.1 M) was added potassium carbonate (3.0 eq.) and the suspension degassed with argon for 10 min. Thiophenol (1.5 eq.) was added with the immediate development of a rich yellow colour. The reaction was stirred at room temperature for 1 h and subsequently diluted with diethyl ether (10 $mL.mmol^{-1}$), washed with saturated sodium bicarbonate solution (3 x 10 $mL.mmol^{-1}$), and the combined aqueous layers extracted with diethyl ether (3 x 10 $mL.mmol^{-1}$). The combined organic layers were dried over magnesium sulfate, filtered and the reaction concentrated *in vacuo*.

General procedure N: Sidechain Boc deprotection

To a stirring solution of Boc-protected amine (1 eq.) in dichloromethane (0.1 M) was added TFA (1:1 volume ratio with dichloromethane). The reaction was stirred at room temperature for 30 min before the solvent was removed *in vacuo*. The residual TFA was removed *via* co-evaporation three times with toluene.

(S)-(9H-Fluoren-9-yl)methyl tert-butyl (6-hydroxyhexane-1,5-diyl)dicarbamate 45

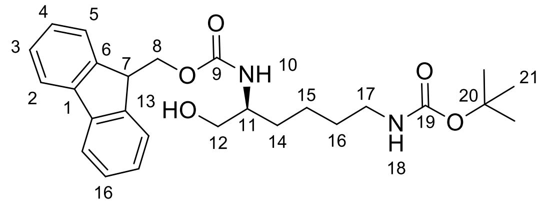

According to *general procedure A*: Fmoc-Lys(Boc)-OH **1** (5.0 g, 10.7 mmol) gave the *title compound **45*** (4.0 g, 82 %) as a white solid after purification by flash column chromatography (Et_2O); $[\alpha]_D^{25.0}$ -30.1 (*c* 0.1, $CHCl_3$); δ_H (400 MHz, $CDCl_3$) 7.76 (d, *J* 7.6, 2 H, H5), 7.60 (d, *J* 7.6, 2H, H2), 7.37 - 7.43 (m, 2 H, H4), 7.28 - 7.34 (m, 2 H, H3), 5.25 (d, *J* 7.6, 1 H, H10), 4.65 - 4.73 (m, 1 H, H18), 4.40 (d, *J* 6.6, 2 H, H8, H8'), 4.15 - 4.25 (m, 1 H, H7), 3.49 - 3.74 (m, 3 H, H11, H12, H12'), 2.99 - 3.23 (m, 2 H, H17, H17'), 1.57 - 1.68 (m, 1 H, H14), 1.41 - 1.52 (m, 12 H, H14', H16, H16', H21), 1.30 - 1.40 (m, 2 H, H15, H15'); δ_C (101 MHz, $CDCl_3$) 156.7 (C=O), 156.4 (C=O), 143.8 (C6), 141.2 (C1), 127.6 (C4), 127.0 (C3), 125.0 (C2), 119.9 (C5), 79.2 (C20), 66.5 (C8), 64.6 (C12), 52.9 (C11), 47.2 (C7), 39.6 (C17), 30.4 (C14), 29.8 (C16), 28.3 (C21), 22.6 (C15); HRMS calculated for $C_{26}H_{43}N_2O_5Na$ $[M+Na]^+$: 477.2360, found 477.2357; IR (CH_2Cl_2) 3330, 2936, 2864, 2361, 2340, 1759, 1689, 1524.

(S)-tert-Butyl (6-hydroxy-5-(4-methylphenylsulfonamido)hexyl)carbamate 2

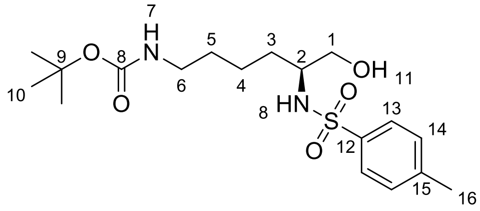

According to *general procedure B*: Fmoc-protected amine (*S*)-(9H-Fluoren-9-yl)methyl tert-butyl (6-hydroxyhexane-1,5-diyl)dicarbamate **45** (2.00 g, 4.4 mmol) gave a residue that was subjected to *general procedure C* with tosyl chloride (1.67 g, 8.8 mmol) to give *title compound* **2** (1.07 g, 63 %) as a colourless oil after purification by flash column chromatography (Et_2O); $[\alpha]_D^{25.0}$ +9.00 (*c* 1.00, $CHCl_3$); δ_H (500 MHz, DMSO-d_6) 7.68 (d, *J* 7.0, 2 H, H13), 7.35 - 7.39 (m, 3 H, H8, H14), 6.64 - 6.70 (m, 1 H, H7), 4.60 (t, *J* 6.0, 1 H, H11), 3.20 - 3.29 (m, 1 H, H1), 3.05 - 3.13 (m, 1 H, H1'), 2.92 - 3.01 (m, 1 H, H2), 2.71 – 2.75 (m, 2 H, H6, H6'), 2.38 (s, 3 H, H16), 1.42 - 1.50 (m, 1 H, H3), 1.38 (s, 9 H, H10), 1.16 (m, 4 H, H3', H4, H5, H5'), 0.84 - 0.99 (m, 1 H, H4'); δ_C (126 MHz, DMSO-d_6) 156.0 (C8), 142.7 (C12), 139.6 (C15), 129.9 (C14), 126.9 (C13), 77.8 (C9), 63.9 (C1), 55.4 (C2), 39.9 (C6), 31.1 (C3), 29.7 (C5), 28.7 (C10), 22.6 (C4), 21.4 (C16); HRMS calculated for $C_{18}H_{30}N_2O_5NaS$ $[M+Na]^+$: 409.1768, found 409.1768; IR (CH_2Cl_2) 3528, 3366, 3282, 2930, 2865, 1681, 1598, 1526.

(S)-tert-Butyl (6-hydroxy-5-(4-nitrophenylsulfonamido)hexyl)carbamate 3

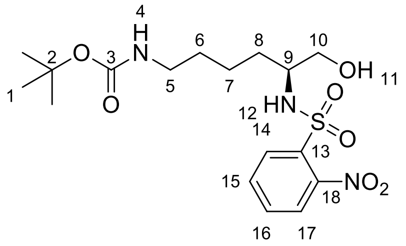

According to *general procedure B*: Fmoc-protected amine (*S*)-(9H-Fluoren-9-yl)methyl tert-butyl (6-hydroxyhexane-1,5-diyl)dicarbamate **45** (1.60 g, 3.50 mmol) gave a white solid residue that was subjected to *general procedure D* with *o*-nosyl chloride (750 mg, 3.39 mmol) to give the *title compound* ***3*** (750 mg, 51 %) as a white foam after purification by flash column chromatography (PE:Et_2O, 1:4); $[\alpha]_D^{25.0}$ +2.1 (*c* 1.00, $CHCl_3$); δ_H (400 MHz, $CDCl_3$) 8.08 - 8.13 (m, 1 H, Ar-H), 7.80 - 7.84 (m, 1 H, Ar-H), 7.70 - 7.74 (m, 2 H, Ar-H), 4.72 (t, *J* 5.9, 1 H, H4), 4.28 (br. s., 2 H, H11, H12), 3.47 - 3.52 (m, 2 H, H10, H10'), 3.40 - 3.46 (m, 1 H, H9), 2.89 - 3.01 (m, 2 H, H5, H5'), 1.47 - 1.58 (m, 2 H, H8, H8'), 1.39 (s, 9 H, H1), 1.31 - 1.36 (m, 2 H, H6, H6'), 1.13 - 1.29 (m, 2 H, H7, H7'); δ_C (101 MHz, $CDCl_3$) 156.1 (C3), 147.5 (Ar-C), 134.5 (Ar-C), 133.4 (Ar-C), 132.8 (Ar-C), 130.4 (Ar-C), 125.1 (Ar-C), 79.0 (C2), 63.9 (C10), 56.4 (C9), 39.7 (C5), 30.9 (C6),

29.4 (C8), 28.2 (C1), 22.3 (C7); HRMS calculated for $C_{17}H_{26}N_3O_7S$ $[M-H]^-$: 416.1497, found 416.1497; IR (CH_2Cl_2) 3352 (broad), 2938, 2868, 2253, 1686, 1540.

(S)-tert-Butyl (4-(1-tosylaziridin-2-yl)butyl)carbamate 4

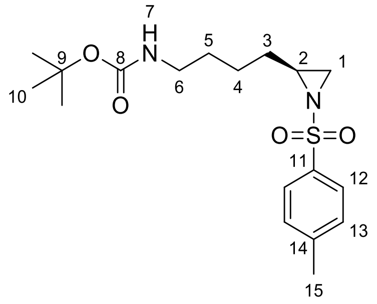

According to *general procedure E*: alcohol **2** (1.07 g, 2.77 mmol) gave the *title compound* ***4*** (750 mg, 74 %) as a colourless oil* after purification by flash column chromatography (Et_2O); [α]D$^{25.0}$ +80.3 (*c* 0.10, $CHCl_3$); δ_H (400 MHz, $CDCl_3$) 7.74 (d, *J* 7.2, 2 H, H12), 7.27 (d, *J* 8.6, 2 H, H13), 4.48 - 4.60 (m, 1 H, H7), 2.89 - 2.98 (m, 2 H, H6, H6'), 2.61 – 2.65 (m, 1 H, H2), 2.54 (d, *J* 6.8, 1 H, H1), 2.37 (s, 3 H, H15), 1.98 (d, *J* 4.4, 1 H, H1'), 1.50 - 1.58 (m, 1 H, H3), 1.30 - 1.41 (m, 11 H, H5, H5', H10), 1.18 - 1.28 (m, 3 H, H3', H4, H4'); δ_C (101 MHz, $CDCl_3$) 155.8 (C8), 144.4 (C11), 134.9 (C14), 129.5 (C13), 127.9 (C12), 78.9 (C9), 40.1 (C2), 39.9 (C6), 33.7 (C1), 30.7 (C3), 29.2 (C5), 28.3 (C10), 23.8 (C4), 21.5 (C15); HRMS calculated for $C_{18}H_{28}N_2O_4NaS$ $[M+Na]^+$: 391.1662, found 391.1668; IR (CH_2Cl_2) 3391 (broad), 2977, 2932, 2865, 1697, 1455.

* N-sulfonyl aziridines are prone to polymerisation upon standing[2,3] therefore, a small aliquot was purified and characterised and the bulk carried forward immediately as a dilute solution in CH_2Cl_2.

(S)-tert-Butyl (5-(4-methylphenylsulfonamido)-6-(phenylamino)hexyl)carbamate 6

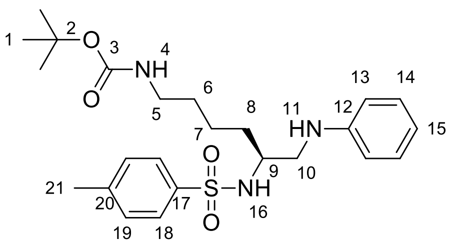

According to *general procedure G*: aziridine **4** (100 mg, 0.27 mmol) and aniline (1 mL) gave the *title compound* **6** (118 mg, 95 %) as a white solid after purification by flash column chromatography (diethyl ether); $[\alpha]_D^{25.0}$ -23.3 (*c* 0.90, $CHCl_3$); δ_H (400 MHz, $CDCl_3$) 7.67 (d, *J* 8.1, 2 H, H18), 7.17 (d, *J* 8.1, 2 H, H14), 7.03 (dd, *J* 8.2, 7.0, 2 H, H19), 6.57 - 6.63 (m, 1 H, H15), 6.36 (d, *J* 7.8, 2 H, H13), 5.23 (br. s., 1 H, H11), 4.47 (br. s, 1 H, H4), 3.23 - 3.34 (m, 1 H, H9), 2.96 - 3.05 (m, 2 H, H10, H10'), 2.88 - 2.94 (m, 2 H, H5, H5'), 2.33 (s, 3 H, H21), 1.35 - 1.37 (m, 11 H, H1, H8, H8'), 1.21 - 1.26 (m, 2 H, H6, H6'), 1.17 - 1.19 (m, 1 H, H7), 0.97 - 1.08 (m, 1 H, H7'); δ_C (101 MHz, $CDCl_3$) 156.1 (C3), 147.6 (C12), 143.4 (C17), 137.6 (C20), 129.6 (C14), 129.1 (C19), 127.1 (C18), 117.4 (C15), 112.8 (C13), 79.1 (C2), 53.1 (C9), 47.8 (C10), 39.8 (C5), 32.6

(C8), 29.6 (C6), 28.4 (C1), 22.2 (C7), 21.4 (C21); HRMS calculated for $C_{24}H_{36}N_3O_4S$ $[M+H]^+$: 462.2421 found 462.2420; IR (CH_2Cl_2) 3398 (broad), 2976, 2932, 2864, 1688, 1603, 1510.

(S)-tert-Butyl (5-(4-nitrophenylsulfonamido)-6-(phenylamino)hexyl)carbamate 7

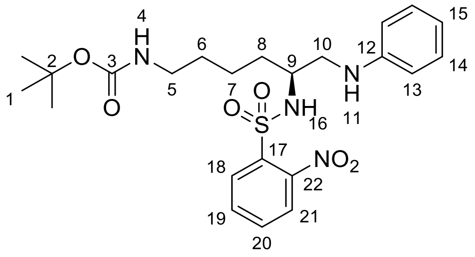

Amino-alcohol **3** (183 mg, 0.44 mmol) was subjected to *general procedure E*: and purified by flash column chromatography (PE:Et_2O, 1:4). The eluent was concentrated *in vacuo* to a total volume of 2 mL. According to *general procedure M*: aniline (2 mL) was added to the residue to give the *title compound **7*** (70 mg, 32 % across 2 steps) as a white foam after purification by flash column chromatography (Et_2O); $[\alpha]_D^{25.0}$ +38.5 (*c* 1.00, $CHCl_3$); δ_H (400 MHz, $CDCl_3$) 7.93 (dd, *J* 7.5, 1.6, 1 H, Ar-H), 7.65 - 7.69 (m, 1 H, Ar-H), 7.45 - 7.55 (m, 2 H, Ar-H), 6.97 (dd, *J* 8.3, 7.6 Hz, 2 H, H14), 6.54 - 6.59 (m, 1 H, Ar-H), 6.27 (d, *J* 7.8, 2 H, H13), 5.56 (d, *J* 7.6, 1 H, H16), 4.48 (br. s., 1 H, H4), 3.55 (td, *J* 7.6, 5.1, 2 H, H9, H11), 3.12 (dd, *J* 15.0, 5.0, 1 H, H10), 3.02 (dd, *J* 15.0, 5.0, 1 H, H10'), 2.90 - 2.98 (s, 2 H, H5), 1.44 - 1.54 (m, 2 H, H6, H6'), 1.33 - 1.40 (m, 13 H, H1, H7, H7', H8, H8'); δ_C (101 MHz, $CDCl_3$) 156.0 (C3), 147.4, 147.1, 134.3, 133.3, 132.7, 130.5, 129.1, 125.1, 117.5, 112.5 (10 x Ar-C), 79.1 (C2), 54.3 (C9), 47.8 (C10), 39.9 (C5), 32.9 (C8), 29.6 (C6), 28.4 (C1), 22.4 (C7); HRMS calculated for $C_{23}H_{33}N_4O_6S$ $[M+H]^+$: 493.2115, found 493.2112; IR (CH_2Cl_2) 3344 (broad), 2955, 2912, 2875, 2211, 1670, 1550.

tert-Butyl (S)-(4-(2-oxo-1-phenyl-3-tosylimidazolidin-4-yl)butyl)carbamate 8

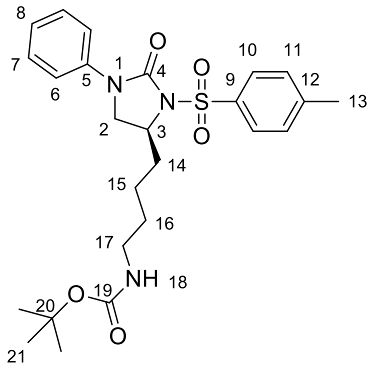

According to *general procedure I*: diamine **6** (110 mg, 0.24 mmol) and triphosgene (30 mg, 0.10 mmol) gave the *title compound **8*** (70 mg, 60 %) as a colourless residue after purification by flash column chromatography (PE:Et_2O, 1:2); $[\alpha]_D^{25.0}$ -30.1 (*c* 0.50, $CHCl_3$); δ_H (400 MHz, $CDCl_3$) 7.91 (d, *J* 8.0, 2 H, H10),7.33 - 7.39 (m, 2 H, H11), 7.21 - 7.28 (m, 4 H, H6, H7), 6.99 - 7.05 (m, 1 H, H8), 4.45 - 4.55 (m, 1 H, H18), 4.32 - 4.40 (m, 1 H, H3), 3.93 (t, *J* 9.2, 1 H, H2), 3.44 (dd, *J* 9.0, 3.0, 1 H, H2'), 2.98 - 3.06 (m, 2 H, H17, H17'), 2.34 (s, 3 H, H13), 1.92 - 2.02 (m, 1 H, H14), 1.75 - 1.86 (m, 1 H, H14'), 1.39 - 1.52 (m, 2 H, H16, H16'), 1.35 (s, 9 H, H21), 1.23 - 1.31

(m, 2 H, H15, H15'); δ_C (101 MHz, $CDCl_3$) 156.0 (C19), 151.5 (C4), 144.8, 138.3, 136.1, 129.5, 128.9, 128.2, 124.2, 118.6, 79.1 (C20), 53.3 (C3), 48.0 (C2), 40.0 (C17), 34.5 (C14), 29.8 (C16), 28.3 (C21), 21.6 (C13), 20.9 (C15); HRMS calculated for $C_{25}H_{33}N_3O_5NaS$ $[M+Na]^+$ 510.2033, found 510.2032; IR (CH_2Cl_2) 3396 (broad), 3064, 2975, 2930, 2866, 2115, 1725, 1598, 1503, 1458.

(S)-tert-Butyl (4-(3-((4-nitrophenyl)sulfonyl)-2-oxo-1-phenylimidazolidin-4-yl)butyl)carbamate 9

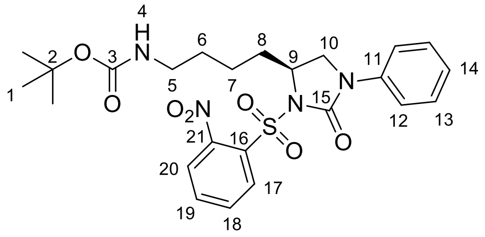

According to *general procedure I*: diamine **7** (50 mg, 0.10 mol) gave the *title compound* **9** (35 mg, 68 %) as a white solid after purification by flash column chromatography (PE:Et_2O, 1:1); $[\alpha]_D^{25.0}$ +154 (*c* 1.00, $CHCl_3$); δ_H (400 MHz, $CDCl_3$) 8.36 - 8.43 (m, 1 H, Ar- H), 7.64 - 7.69 (m, 3 H, Ar-H), 7.32 - 7.37 (m, 2 H, Ar-H), 7.22 - 7.28 (m, 2 H, Ar-H), 7.01 - 7.07 (m, 1 H, Ar-H), 4.42 - 4.57 (m, 2 H, H4, H9), 4.21 (t, *J* 8.9, 1 H, H10'), 3.49 (dd, *J* 9.2, 1.6, 1 H, H10), 3.03 - 3.12 (m, 2 H, H5, H5'), 1.84 - 1.98 (m, 2 H, H8, H8'), 1.41 - 1.53 (m, 4 H, H6, H6', H7, H7'), 1.35 (s, 9 H, H1); δ_C (101 MHz, $CDCl_3$) 156.1 (C3), 151.0 (C15), 147.9, 138.2, 134.9, 134.7, 132.0, 131.9, 129.1, 124.6, 124.3, 118.8 (10 x Ar-C), 79.2 (C2), 54.4 (C9), 48.6 (C10), 40.1 (C5), 35.6 (C8), 29.9 (C6), 28.4 (C1), 21.5 (C7); HRMS calculated for $C_{24}H_{30}N_4O_7SNa$ $[(M+H)]^+$ 541.1727 found 541.1723; IR (CH_2Cl_2) 2980, 2933, 2865, 1734, 1705.

(S)-tert-Butyl (4-(2-oxo-3-tosylimidazolidin-4-yl)butyl)carbamate 12

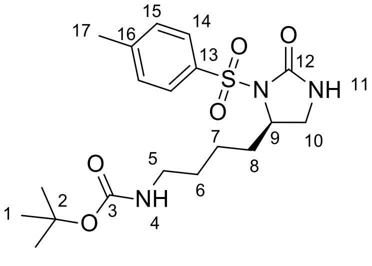

Aziridine **4** (250 mg, 0.67 mmol) was subjected to *general procedure H* followed by *general procedure O*: triphosgene (80 mg, 0.27 mmol) gave the *title compound* ***12*** (220 mg, 79 % over two steps) as a white solid after purification by flash column chromatography (Et_2O); $[\alpha]_D^{25.0}$ -23.0 (*c* 1.00, $CHCl_3$); δ_H (400 MHz, $CDCl_3$) 7.82 (d, *J* 8.3, 2 H, H14), 7.23 (d, *J* 8.1, 2 H, H15), 6.31 (s, 1 H, H11), 4.55 - 4.67 (m, 1 H, H4), 4.20 - 4.30 (m, 1 H, H9), 3.44 (t, *J* 9.2, 1 H, H10), 3.05 (dd, *J* 9.2, 3.5, 1 H, H10'), 2.96 - 3.03 (m, 2 H, H5, H5'), 2.35 (s, 3 H, H17), 1.78 - 1.89 (m, 1 H, H8),

1.67 - 1.78 (m, 1 H, H8’), 1.35 - 1.44 (m, 11 H, H1, H6, H6’), 1.19 - 1.27 (m, 2 H, H7, H7’); δ_C (101 MHz, $CDCl_3$) 156.1 (C=O), 155.9 (C=O), 144.4 (C13), 136.2 (C16), 129.4 (C15), 127.7 (C14), 79.0 (C2), 56.9 (C9), 42.9 (C10), 40.0 (C5), 34.3 (C8), 29.6 (C6), 28.3 (C1), 21.5 (C17), 20.9 (C7); HRMS calculated for $C_{19}H_{29}N_3O_5NaS$ $[M+Na]^+$: 434.1720, found 434.1720; IR (CH_2Cl_2) 3342 (broad), 2691, 2914, 2890, 2854, 1744, 1712.

(S)-tert-Butyl (4-(3-((4-nitrophenyl)sulfonyl)-2-oxoimidazolidin-4-yl)butyl)carbamate 13

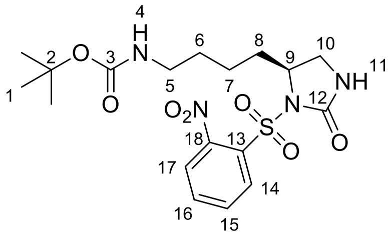

According to *general procedure E*: Amino-alcohol **3** (548 mg, 1.31 mmol) gave residue **A** that was purified by flash column chromatography (PE:Et_2O, 1:4) and the eluents concentrated *in vacuo* to a total volume of 4 mL. According to *general procedure H*: 4 mL solvent containing residue **A** gave residue **B**. According to *general procedure I*: residue **B** gave the *title compound* ***13*** (140 mg, 24 % across 3 steps) as a yellow foam after purification by flash column chromatography (CH_2Cl_2:MeOH, 19:1); $[\alpha]_D^{25.0}$ +9.00 (*c* 1.00, $CHCl_3$); δ_H (400 MHz, $CDCl_3$) 8.24 - 8.35 (m, 1 H, Ar-H), 7.62 - 7.70 (m, 3 H, Ar-H), 5.98 (s, 1 H, H4), 4.68 (br. s., 1 H, H11), 4.35 - 4.45 (m, 1 H, H9), 3.66 (t, *J* 8.9, 1 H, H10), 3.13 (d, *J* 9.0, 1 H, H10'), 3.06 (d, *J* 6.1, 2 H, H5, H5'), 1.78 - 1.85 (m, 2 H, H8, H8'), 1.42 - 1.51 (m, 2 H, H6, H6'), 1.37 (s, 9 H, H1), 1.31 - 1.34 (m, 2 H, H7, H7'); δ_C (101 MHz, $CDCl_3$) 169.3 (C12), 155.0 (C3), 147.7, 134.5, 134.3, 131.9, 131.7, 124.0 (6 x Ar-C), 79.0 (C2), 57.8 (C9), 43.4 (C10), 40.1 (C5), 35.3 (C8), 29.6 (C6), 28.3 (C1), 21.3 (C7); HRMS calculated for $C_{18}H_{26}N_4O_7SNa$ $[M+H]^+$: 465.1414, found 465.1411; IR (CH_2Cl_2) 3337, 3096, 2978, 2934, 2865, 1711, 1685.

(S)-tert-Butyl (4-(1-(6-bromopyridin-2-yl)-2-oxo-3-tosylimidazolidin-4-yl)butyl)carbamate 14

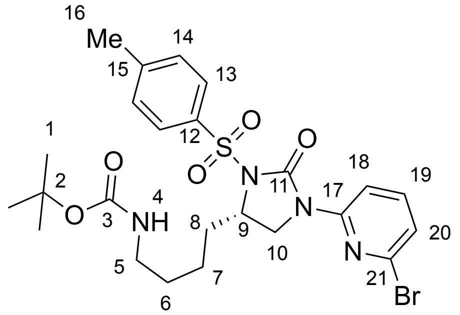

According to *general procedure L*: cyclic urea **12** (200 mg, 0.49 mmol) and 2,6-dibromopyridine (136 mg, 0.58 mmol) gave the *title compound* ***14*** (110 mg, 40 %) as a colourless oil after purification by flash column chromatography (PE:Et_2O, 1:1); $[\alpha]_D^{25.0}$ +24.5 (*c* 1.00, $CHCl_3$); δ_H (400 MHz, $CDCl_3$) 8.01 (dd, *J* 8.3, 0.7, 1 H, H20), 7.90 (d, *J* 8.5, 2 H, H13), 7.39 (t, *J* 8.1, 1 H, H19), 7.27 (d, *J* 8.1, 2 H, H14), 7.08 (dd, *J* 7.6, 0.5, 1 H, H18), 4.48 - 4.58 (m, 1 H, H4), 4.36 (br. s., 1 H, H9), 4.01 (dd, *J* 11.0, 9.3, 1 H, H10), 3.76 (dd, *J* 10.9, 3.3, 1 H, H10'), 2.98 – 3.06 (m, 2 H, H5, H5'), 2.35 (s, 3 H, H16), 1.90 - 2.02 (m, 1 H, H8), 1.69 - 1.83 (m, 1 H, H8'), 1.41 - 1.52 (m,

2 H, H6, H6’), 1.36 (s, 9 H, H1), 1.24 - 1.31 (m, 2 H, H7, H7’); δ_C (101 MHz, $CDCl_3$) 156.0, 156.0 (2 x C=O), 151.3 (Ar-C), 150.9 (Ar-C), 145.1 (Ar-C), 139.8 (C19), 135.9 (Ar-C), 129.7 (C14), 128.3 (C13), 123.0 (C18), 112.0 (C20), 79.2 (C2), 53.6 (C9), 46.8 (C10), 40.1 (C5), 34.5 (C8), 29.8 (C6), 28.4 (C1), 21.7 (C16), 21.0 (C7); HRMS calculated for $C_{24}H_{31}N_4O_5NaBrS$ $[M+Na]^+$: 591.1070, found 591.1070; IR (CH_2Cl_2) 3566 (broad), 2960, 2914, 2890, 2853, 1736, 1713, 1442.

(S)-tert-Butyl (4-(1-(6-bromopyridin-2-yl)-3-((2-nitrophenyl)sulfonyl)-2-oxoimidazolidin-4-yl)butyl)carbamate 15

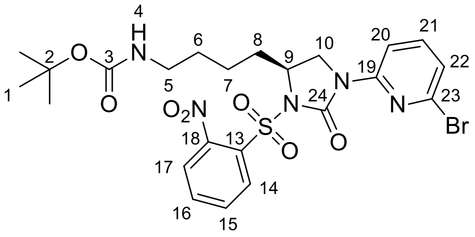

According to *general procedure L*: cyclic urea **13** (130 mg, 0.29 mmol) and 2,6-dibromopyridine (81 mg, 0.35 mmol) gave the *title compound **15*** (60 mg, 35 %) as a pale yellow foam after purification by flash column chromatography (PE:Et_2O, 1:1); $[\alpha]_D^{25.0}$ +254 (*c* 1.00, $CHCl_3$); δ_H (400 MHz, $CDCl_3$) 8.37 - 8.41 (m, 1 H, nosyl-H), 7.91 (d, *J* 8.1, 1 H, H22), 7.66 - 7.73 (m, 3 H, nosyl-H), 7.39 (t, *J* 8.1, 1 H, H21), 7.10 (d, *J* 7.6, 1 H, H20), 4.42 - 4.55 (m, 2 H, H4, H9), 4.22 (dd, *J* 10.9, 8.9, 1 H, H10), 3.87 (dd, *J* 10.9, 2.1, 1 H, H10’), 3.03 - 3.13 (m, 2 H, H5, H5’), 1.83 - 1.94 (m, 2 H, H8, H8’), 1.46 - 1.54 (m, 2 H, H6, H6’), 1.36 (s, 9 H, H1), 1.17 - 1.33 (m, 2 H, H7, H7’); δ_C (101 MHz, $CDCl_3$) 156.0 (C3), 150.8 (C24), 150.7 (C19), 148.0 (C18), 139.8 (C21), 139.4 (C23), 135.0 (nosyl-C), 134.8 (nosyl-C), 132.0 (nosyl-C), 131.6 (C13), 124.4 (nosyl-C), 123.2 (C20), 112.0 (C22), 79.2 (C2), 54.7 (C9), 47.4 (C10), 40.1 (C5), 35.6 (C8), 29.8 (C6), 28.4 (C1), 21.4 (C7); HRMS calculated for $C_{23}H_{29}N_5O_7SBr$ $[M+H]^+$: 598.0966, found 598.0969; IR (CH_2Cl_2) 3296 (broad), 2980, 1733, 1716, 1698, 1684, 1652.

(S)-tert-Butyl (4-(2-oxo-1-phenylimidazolidin-4-yl)butyl)carbamate 16

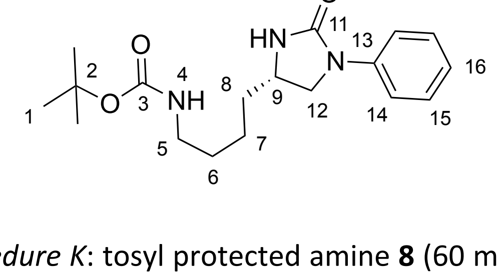

According to *general procedure K*: tosyl protected amine **8** (60 mg, 0.12 mmol) gave the *title compound **16*** (14 mg, 35 %) as a colourless oil after purification by flash column chromatography (Et_2O); $[\alpha]_D^{25.0}$ +5.00 (*c* 1.00, $CHCl_3$); δ_H (400 MHz, $CDCl_3$) 7.46 (dd, *J* 9.0, 1.0, 2 H, H14), 7.24 - 7.29 (m, 2 H, H15), 6.95 - 7.00 (m, 1 H, H16), 5.07 (br. s., 1 H, H10), 4.51 (br.

s., 1 H, H4), 3.93 (t, *J* 8.8, 1 H, H12), 3.68 - 3.75 (m, 1 H, H9), 3.46 (dd, *J* 8.8, 6.3, 1 H, H12'), 3.02 - 3.11 (m, 2 H, H5, H5'), 1.55 - 1.59 (m, 2 H, H8, H8'), 1.44 - 1.50 (m, 2 H, H6, H6'), 1.37 (s, 9 H, H1), 1.29 - 1.35 (m, 2 H, H7, H7'); δ_C (101 MHz, $CDCl_3$) 158.8 (C=O), 156.0 (C=O), 140.0 (C13), 128.8 (C15), 122.6 (C16), 117.7 (C14), 79.2 (C2), 50.9 (C12), 49.0 (C9), 40.2 (C5), 35.6 (C6), 30.3 (C8), 28.4 (C1), 22.4 (C7); HRMS calculated for $C_{18}H_{28}N_3O_3$ $[M+H]^+$: 334.2125, found 334.2119; IR (CH_2Cl_2) 2980, 2948, 2322, 1698, 1558, 1541.

Tosyl-protected Lys-Lys dimer 17

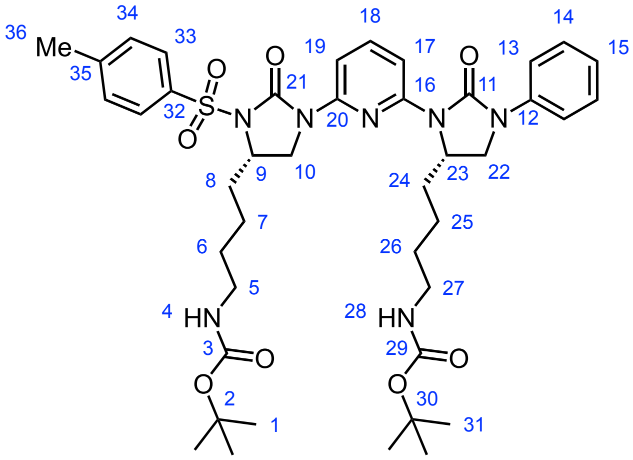

According to *general procedure L*: cyclic urea **16** (14 mg, 0.04 mmol) and bromide **14** (29 mg, 0.05 mmol) gave the *title compound* ***17*** (21 mg, 61 %) as a colourless oil after purification by flash column chromatography (PE:Et_2O, 1:4); $[\alpha]_D^{25.0}$ +25.4 (*c* 1.00, $CHCl_3$); δ_H (500 MHz, $CDCl_3$) 7.92 (d, *J* 8.4, 2 H, H13), 7.88 (d, *J* 8.0, 1 H, H17 or H19), 7.67 (d, *J* 7.9, 1 H, H17 or H19), 7.55 (t, *J* 8.0, 1 H, H18), 7.48 - 7.52 (m, 2 H, H33 or H34), 7.25 - 7.33 (m, 4 H, H14, H33 or H34), 7.01 - 7.07 (m, 1 H, H15), 4.91 - 5.00 (m, 1 H, H4 or H28), 4.76 - 4.84 (m, 1 H, H4 or H28), 4.53 - 4.61 (m, 1 H, H9 or H23), 4.32 - 4.39 (m, 1 H, H9 or H23), 4.03 (t, *J* 9.0, 1 H, H10 or H22), 3.98 (t, *J* 9.0, 1 H, H10 or H22), 3.64 - 3.72 (m, 1 H, H10’ or H22’), 3.53 (dd, *J* 9.0, 3.0, 1 H, H10’ or H22’), 2.96 - 3.06 (m, 4 H, H5, H5’, H27, H27’), 2.36 (s, 3 H, H36), 1.97 - 2.04 (m, 1 H, H6 or H26), 1.89 - 1.96 (m, 1 H, H6 or H26), 1.77 - 1.85 (m, 1 H, H6’ or H26’), 1.60 - 1.69 (m, 1 H, H6’ or H26’), 1.38 - 1.54 (m, 4 H, H8, H8’, H24, H24’), 1.36 (s, 9 H, H1 or H31), 1.34 (s, 9 H, H1 or H31), 1.24-1.30 (m, 4H, H7, H7’, H25, H25’); δ_C (126 MHz, $CDCl_3$) 156.1 (2C, C3, C29), 154.1 (C11 or C21), 151.3 (C11 or C21), 149.8 (Ar-C), 148.8 (Ar-C), 144.9 (Ar-C), 139.6 (C18), 139.5 (Ar-C), 136.0 (Ar-C), 129.6 (C14 or C33 or C34), 128.9 (C14 or C33 or C34), 128.3 (C13), 123.5 (C15), 118.4 (C33 or C34), 108.9 (C17 or C19), 107.4 (C17 or C19), 79.1 (2C, C2, C30), 53.4 (C9 or C23), 51.3 (C9 or C23), 47.5 (C10 or C22), 46.6 (C10 or C22), 40.4 (2C, C5, C27), 34.8 (C6 or C26), 32.8 (C6 or C26), 30.3 (C8 or C24), 30.0 (C8 or C24), 28.4 (2C, C1, C31), 22.2 (C7 or C25), 21.7 (C36), 21.4 (C7 or C25); HRMS calculated for $C_{42}H_{58}N_7O_8S$ $[M+H]^+$: 820.4062, found 820.4058; IR (CH_2Cl_2) 3015, 3001, 2998, 2963, 1657, 1545.

Nosyl-protected Lys-Lys dimer 18

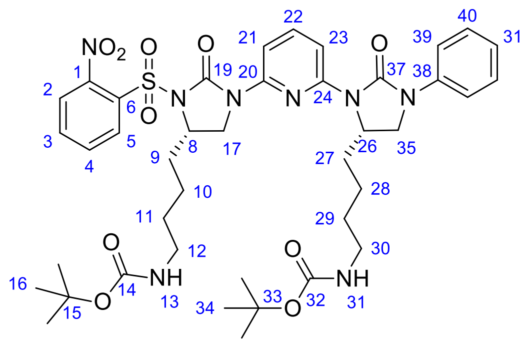

According to *general procedure L*: cyclic urea **16** (20 mg, 0.06 mmol) and bromide **15** (30 mg, 0.05 mmol) gave the *title compound* ***18*** (20 mg, 47 %) as a yellow residue after purification by flash column chromatography (CH_2Cl_2:MeOH, 9:1); $[\alpha]_D^{23.5}$ -164.0 (*c* 0.10, $CHCl_3$); δ_H (500 MHz, $CDCl_3$) 8.39 - 8.43 (m, 1 H), 7.91 (dd, *J* 6.0, 2.0, 1 H, Ar-H), 7.66 - 7.71 (m, 3 H, Ar-H), 7.53 - 7.56 (m, 2 H, Ar-H), 7.50 (d, *J* 7.9, 2 H, Ar-H), 7.30 (t, *J* 7.9, 2 H, Ar-H), 7.04 (t, *J* 7.4, 1 H, Ar-H), 4.95 - 5.10 (m, 1 H, H13 or H31), 4.72 - 4.83 (m, 1 H, H13 or H31), 4.57 - 4.65 (m, 1 H, H8 or H26), 4.43 - 4.50 (m, 1 H, H8 or H26), 4.25 (t, *J* 9.5, 1 H, H17 or H35), 3.98 (t, *J* 9.0, 1 H, H17 or H35), 3.76 (d, *J* 9.0, 1 H, H17' or H35'), 3.54 (dd, *J* 9.0, 2.9, 1 H, H17' or H35'), 3.01 - 3.10 (m, 4 H, H12, H12', H30, H30'), 1.88 - 2.00 (m, 3 H, 3 of H9, H9', H27, H27'), 1.65 - 1.72 (m, 1 H, 1 of H9, H9', H27, H27'), 1.39 - 1.56 (m, 8 H, H10, H10', H11, H11', H28, H28', H29, H29'), 1.34 - 1.37 (m, 18 H, H16, H34); δ_C (126 MHz, $CDCl_3$) 156.2, 156.1, 154.1, 150.8 (4 x C=O), 150.0, 148.6, 147.9, 139.6, 139.5, 134.9, 134.8, 132.0, 131.7, 128.9, 124.4, 123.5, 118.4, 109.1, 107.2 (15 x Ar-C), 79.1 (C15 or C33), 79.1 (C15 or C33), 54.5 (C8 or C26), 51.3 (C8 or C26), 47.5 (C17 or C35), 47.2 (C17 or C35), 40.4 (2C, C12, C30), 35.8 (C9 or C27), 32.9 (C9 or C27), 30.5 (C11 or C29), 30.3 (C11 or C29), 28.4 (2C, C16, C34), 22.2 (C10 or C28), 21.8 (C10 or C28); HRMS calculated for $C_{41}H_{54}N_8O_{10}SNa$ $[M+Na]^+$: 873.3576 found 873.3554; IR (CH_2Cl_2) 3367 (broad), 2928, 2858, 1709, 1584, 1485.

N-Deprotected Lys-Lys dimer 46

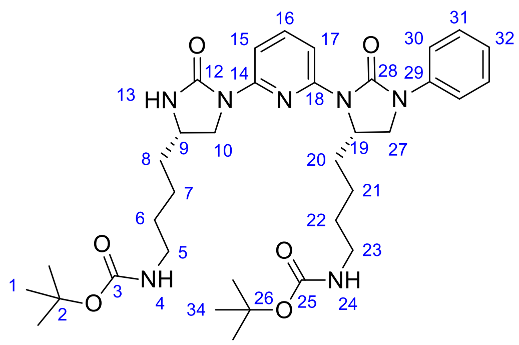

According to *general procedure M*: *N*-nosyl urea **18** (21 mg, 0.025 mmol) gave the *title compound* ***46*** (16 mg, 96 %) as a pale yellow residue after purification by flash column chromatography (CH_2Cl_2:MeOH, 9:1); $[\alpha]_D^{23.5}$ +20.7 (*c* 0.10, $CHCl_3$); δ_H (600 MHz, $CDCl_3$) 7.79 (dd, *J* 8.1, 3.9, 1 H, Ar-H), 7.65 - 7.73 (m, 1 H, Ar-H), 7.49 - 7.56 (m, 3 H, Ar-H), 7.30 (t, *J* 7.9, 2 H, Ar-H), 7.00 - 7.06 (m, 1 H, Ar-H), 5.04 (br. s., 1 H, H4 or H24), 4.70 - 4.80 (m, 1 H, H4 or H24), 4.56 - 4.68 (m, 1 H, H19), 4.21 - 4.28 (m, 1 H, H13), 4.16 (t, *J* 9.5, 1 H, H10), 3.99 (t, *J* 8.9, 1 H, H27), 3.66 - 3.75 (m, 1 H, H9), 3.55 - 3.60 (m, 1 H, H10'), 3.53 (dd, *J* 8.9, 3.2, 1 H, H27'), 2.99 - 3.09 (m, 4 H, H5, H5', H23, H23'), 1.84 - 2.02 (m, 2 H, H8, H20), 1.42 - 1.71 (m, 10 H, H6, H6', H7, H7', H8', H20', H21, H21', H22, H22), 1.33 - 1.38 (m, 18 H, H1, H34); δ_C (151 MHz, $CDCl_3$) 158.0, 156.1 (C12 or C28), 156.1, 154.3 (C25, C3), 150.3, 149.6, 139.8, 139.2, 134.8, 131.7, 128.9, 123.3, 118.3 (9 x Ar-C), 79.1, 79.1 (C26, C2), 51.2 (C19), 49.6 (C9), 48.8 (C10), 47.5 (C27), 40.4, 40.2 (C23, C5), 35.8, 32.8 (C20, C8), 30.3, 29.9 (C22, C6), 28.4, 28.4 (C34, C1), 22.5, 22.0 (C21, C7); HRMS calculated for $C_{35}H_{52}N_7O_6$ $[M+H]^+$: 666.3974, found 666.3968; IR ($CHCl_3$) 3344 (broad), 2923, 2853, 1710, 1522, 1399, 1366.

Boc-Protected Lys-Lys-Lys trimer 47

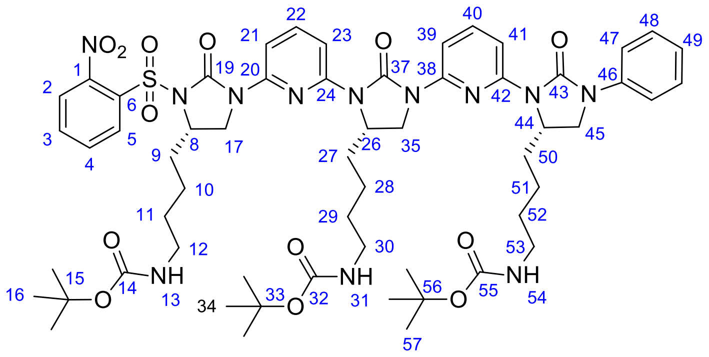

According to *general procedure L*: *N*-deprotected Lys-Lys dimer **46** (20 mg, 0.03 mmol) and bromide **15** (50 mg, 0.05 mmol) gave the *title compound* ***47*** (20 mg, 56 %) as a yellow residue after purification by flash column chromatography (CH_2Cl_2:MeOH, 9:1); $[\alpha]_D^{25.0}$ +14.0 (*c* 0.5, $CHCl_3$); δ_H (600 MHz, $CDCl_3$) 8.42 (s, 1 H, Ar-H), 7.90 - 7.92 (m, 1 H, Ar-H), 7.88 (d, *J* 8.0, 1 H, Ar-H), 7.84 (d, *J* 7.9, 1 H, Ar-H), 7.70 (d, *J* 1.3, 1 H, Ar-H), 7.69 (br. s., 1 H, Ar-H), 7.67 - 7.68 (m, 1 H, Ar-H), 7.61 (s, 1 H, Ar-H), 7.57 - 7.59 (m, 2 H, Ar-H), 7.53 (d, *J* 7.7, 2 H, Ar-H), 7.32 (d, *J* 1.1, 2 H, Ar-H), 7.03 - 7.05 (m, 1 H, Ar-H), 5.51 (br. s, 1 H, NH), 5.13 (br. s, 1 H, NH), 4.79 (br. s, 1 H, NH), 4.64 - 4.67 (m, 1 H, H8 or H26 or H44), 4.57 - 4.61 (m, 1 H, H8 or H26 or H44), 4.45 - 4.48 (m, 1 H, H8 or H26 or H44), 4.23 - 4.26 (m, 1 H, H17 or H35 or H45), 4.06 - 4.11 (m, 1 H, H17 or H35 or H45), 4.01 (t, *J* 9.0, 1 H, H17 or H35 or H45), 3.77 (br. d, *J* 9.0, 2 H, 2 of H17', H35', H45'), 3.56 (dd, *J* 9.0, 3.0, 1 H, H17' or H35' or H45'), 3.04 - 3.08 (m, 6 H, H12, H12', H30, H30', H53, H53'), 1.81 - 2.07 (m, 8 H, 8 of H9, H9', H11, H11', H27, H27', H29, H29', H50, H50', H52, H52'), 1.60 - 1.72 (m, 4 H, 4 of H9, H9', H11, H11', H27, H27', H29, H29', H50, H50', H52, H52'), 1.41 - 1.44 (m, 3 H, H10, H28, H51), 1.36 (d, *J* 2.0, 18 H, 2 of H16 or H34 or H57), 1.34 (s, 9 H,

H16 or H34 or H57), 1.17 - 1.21 (m, 3 H, H10', H28', H51'); δ_C (150 MHz, $CDCl_3$) 156.4, 156.2, 156.2, 154.2, 153.7, 150.7 (6 x C=O), 149.9, 149.8, 149.7, 148.7, 147.9, 147.6, 139.7, 139.4, 134.9, 134.9, 134.8, 132.1, 131.7, 128.9, 125.5, 124.4, 123.4, 118.4, 109.3, 108.1, 107.5, 107.1 (22 x Ar-C), 79.2, 79.2 (2 of C15, C33, C56), 79.0 (1 of C15, C33, C56), 54.5 (1 of C6, C26, C44), 51.4 (1 of C6, C26, C44), 51.2 (1 of C6, C26, C44), 47.5, 47.3, 47.1 (C17, C35, C45), 40.7, 40.5, 40.4 (C12, C30, C53), 35.8, 33.3, 33.0 (C9, C27, C50), 30.7, 30.5, 29.9 (C11, C29, C52), 28.5, 28.4, 28.4 (C16, C34, C57), 22.7, 22.5, 21.8 (C10, C28, C51); IR (CH_2Cl_2) 3350 (broad), 2945, 2956, 2844, 1723, 1699, 1535; HRMS calculated for $C_{58}H_{78}N_{12}O_{13}NaS$ [M+Na]$^+$: 1205.5424, found 1205.5432;

Deprotected Lys-Lys-Lys trimer 19

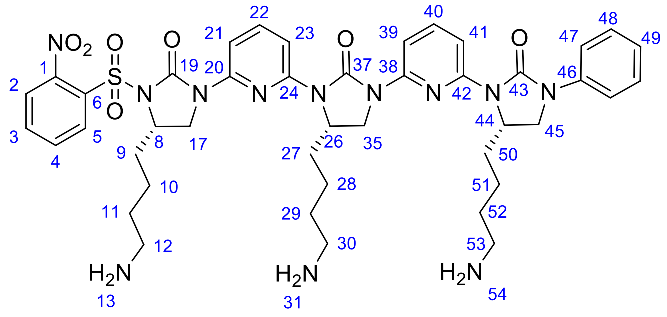

According to *general procedure N*: Boc-protected Lys-Lys-Lys trimer **47** (20 mg, 0.016 mmol) gave the *title compound* ***19*** as a colourless oil (quant.). A portion was purified by reverse phase HPLC (MeCN:H_2O); $[\alpha]_D^{25.0}$ +11.5 (*c* 0.10, H_2O); δ_H (600 MHz, CD_3OD) 8.35 (d, *J* 7.4, 1H, H2), 7.87 - 7.80 (m, 4H, H39, H5, H4, H3), 7.78 (d, *J* 8.4, 1H, H41 or H23), 7.77 (d, *J* 7.9, 1H, H41 or H23), 7.68 (td, *J* 8.2, 6.3, 2H, H40, H22), 7.56 (d, *J* 7.9, 2H, H47), 7.54 (d, *J* 8.2, 1H, H21), 7.33 (t, *J* 8.4, 2H, H48), 7.08 (t, *J* 7.4, 1H, H49), 4.88 - 4.82 (m, 2H, H44, H26), 4.50 - 4.55 (m, 1H, H8), 4.37 (dd, *J* 10.8, 9.2, 1H, H17), 4.27 (t, *J* 10.3, 1H, H35), 4.14 (t, *J* 9.2, 1H, H45), 3.80 (td, *J* 8.9, 3.1, 2H, H17', H35'), 3.69 (dd, *J* 8.9, 3.1, 1H, H45'), 2.91 - 2.95 (m, 2H, 2 of H12, H12', H30, H30', H53, H53'), 2.81 - 2.88 (m, 4H, 4 of H12, H12', H30, H30', H53, H53'), 1.87 - 2.00 (m, 6H, H9, H9', H27, H27', H50, H50'), 1.70 - 1.77 (m, 2H, 2 of H11, H11', H29, H29', H52, H52'), 1.63 - 1.69 (m, 2H, H11, H11', H29, H29', H52, H52'), 1.53 - 1.61 (m, 4H, 4 of H10, H10', H11, H11', H28, H28', H29, H29', H51, H51', H52, H52'), 1.40 - 1.47 (m, 4H, 4 of H10, H10', H28, H28', H51, H51'); δ_C (126 MHz, CD_3OD) 154.8, 154.2, 150.9 (3 x C=O), 150.2, 150.0, 149.9, 149.1 (C20, C24, C38, C42), 148.0 (C1), 139.6, 139.4, 139.0, 135.6 (4 x Ar-C), 134.2 (C2), 131.8 (6 x Ar-C), 130.8 (C6), 128.6 (C48), 124.5 (Ar-C), 123.4 (C49), 118.8 (C47), 109.4 (Ar-C), 108.2 (Ar-C), 107.7 (Ar-C), 107.4 (Ar-C), 54.2 (C8), 51.2 (C26), 50.8 (C17), 46.0 (C35), 39.2, 39.2, 39.1 (C12, C30, C53), 35.2, 32.4, 32.2 (C9, C27, C50), 27.4, 27.4, 26.8 (C11, C29, C52), 20.9, 20.5, 20.4 (C10 or C28 or C51); C45 and C44 are obscured by the solvent peak but were identified by HSQC as being at 47.0 and 47.4 respectively; LRMS calculated for $C_{43}H_{55}N_{12}O_7S$ [M+H]$^+$: 883.4 found 883.4; IR (CH_2Cl_2) 3420 (broad), 2928, 2465 (broad), 1582, 1484, 1450.

tert-Butyl (_S_)-4-(((benzyloxy)carbonyl)amino)-5-hydroxypentanoate 48

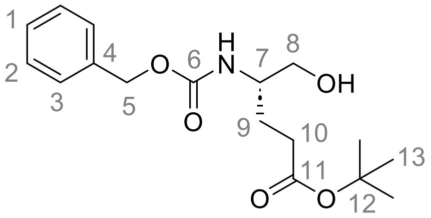

According to *general procedure A*; NMM (0.33 mL, 3.02 mmol), ethyl chloroformate (0.29 mL, 3.05 mmol), Z-Glu-OtBu-OH **20** (1.00 g, 2.96 mmol), and $NaBH_4$ (338 mg, 8.90 mmol) gave the *title compound* ***48*** (947 mg, 2.93 mmol, 99 %) as a colourless oil that was used without further purification; R_f 0.50 (EtOAc/Pet 50:50); δ_H (400 MHz, $CDCl_3$) 7.29-7.18 (5H, m, H1, H2 & H3), 5.18 (1H, br d, *J* 7.7, NH), 5.00 (2H, s, H5), 3.67-3.41 (3H, m, H7 & H8), 2.46 (1H, br s, OH), 2.28-2.17 (2H, m, H10), 1.82-1.61 (2H, m, H9), 1.35 (9H, s, H13); δ_C (100 MHz, $CDCl_3$) 173.3 (C11), 156.6 (C6), 136.4 (C4), 128.5 (C1/C2/C3), 128.1 (C1/C2/C3), 128.0 (C1/C2/C3), 80.0 (C12), 66.7 (C5), 64.7 (C8), 53.0 (C7), 32.0 (C10), 28.0 (C13), 26.0 (C9); $[\alpha]_D^{23.0}$ -14.2 (*c* 2.00, $CHCl_3$).

tert-Butyl (_S_)-5-hydroxy-4-((2-nitrophenyl)sulfonamido)pentanoate 23

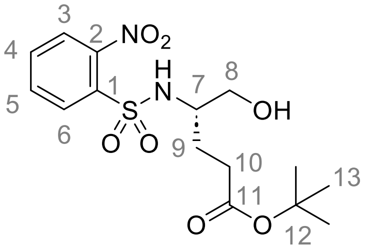

In an adaptation of a literature procedure,[4] *tert*-butyl (*S*)-4-(((benzyloxy)carbonyl)amino)-5-hydroxypentanoate **48** (3.00 g, 9.28 mmol) was dissolved in MeOH (20 mL) and Pd/C (10% by wt., 300 mg) was added before the flask was evacuated and backfilled with hydrogen, three times. The mixture was stirred under hydrogen for 2 h. The mixture was filtered through Celite™ and washed with CH_2Cl_2 (1 L) before the washings were concentrated *in vacuo* to give amino alcohol **21**, which was redissolved in CH_2Cl_2 (20 mL), Et_3N (2.60 mL, 18.5 mmol) and 2-nitrobenzenesulfonyl chloride (2.05 g, 9.28 mmol) were added portion-wise before the mixture was stirred at rt for 18 h. The reaction mixture was washed with H_2O (50 mL) and extracted with CH_2Cl_2 (3 x 50 mL). The organic layers were concentrated *in vacuo* and purified by flash column chromatography (EtOAc/Pet 50:50) to give the *title compound* ***23*** (3.04 g, 8.12 mmol, 88 %) as a colourless oil; R_f 0.60 (EtOAc/Pet 50:50); δ_H (400 MHz, $CDCl_3$) 8.16 (1H, m, H3), 7.86 (1H, m, H6), 7.72 (2H, m, H4 & H5), 5.71 (1H, br d, *J* 8.2, NH), 3.61-3.42 (3H, m, H7 & H8), 2.43-2.22 (2H, m, H10), 2.10-1.69 (3H, m, H9 & OH), 1.42 (9H, s, H13); δ_C (100 MHz, $CDCl_3$) 172.5 (C11), 147.8 (C2), 134.8 (C1), 133.5, 132.9 (C5, C4), 130.7 (C3), 125.4 (C6), 81.0 (C12), 64.1 (C8), 55.9 (C7), 31.2 (C10), 28.1 (C13), 26.7 (C9); IR 3462, 3267, 2976, 2916, 1708, 1537, 1431, 1358, 1333, 1255, 1154, 1105, 943, 852, 654, 593; HRMS (ESI) calculated for $C_{15}H_{21}N_2O_7S$ $[M-H]^-$ 373.1075 found 373.1067; $[\alpha]_D^{23.0}$ -70.5 (*c* 0.50, $CHCl_3$).

***tert*-Butyl (*S*)-5-hydroxy-4-((4-methylphenyl)sulfonamido)pentanoate 49**

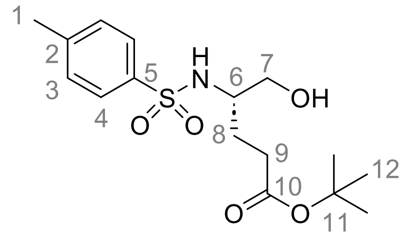

In an adaptation of a literature procedure,[4] *tert*-butyl (*S*)-4-(((benzyloxy)carbonyl)amino)-5-hydroxypentanoate **48** (200 mg, 0.61 mmol) was dissolved in MeOH (2.00 mL) and Pd/C added (10% by wt., 20 mg) before the flask was evacuated and backfilled with hydrogen, three times. The mixture was stirred under hydrogen for 2 h. The mixture was filtered through Celite™ and washed with CH_2Cl_2 (100 mL) before the washings were concentrated *in vacuo*. The residue was redissolved in CH_2Cl_2 (2 mL), DMAP (8.00 mg, 0.06 mmol) and Et_3N (0.17 mL, 1.24 mmol) were added, and the solution cooled to 0 °C. *p*-Toluensulfonyl chloride (117 mg, 0.61 mmol) in CH_2Cl_2 (2 mL) was added cautiously before the mixture was stirred at rt for 18 h. The reaction mixture was washed with H_2O (10 mL) and extracted with CH_2Cl_2 (3 x 10 mL). The organic layers were concentrated *in vacuo* and purified by flash column chromatography (EtOAc/Pet 50:50) to give the *title compound* ***49*** (45 mg, 0.13 mmol, 21 %) as a colourless oil; R_f 0.70 (EtOAc/Pet 50:50); δ_H (400 MHz, $CDCl_3$) 7.75 (2H, dt, *J* 8.5, 1.9, H4), 7.29 (2H, dd, *J* 8.5, 0.7, H3), 5.34 (1H, br d, *J* 8.2, NH), 3.44 (2H, d, *J* 4.0, H7), 3.30-3.21 (1H, m, H6), 2.41 (3H, s, H1), 2.26-2.10 (2H, m, H9), 1.81-1.65 (2H, m, H8), 1.41 (9H, s, H12); δ_C (100 MHz, $CDCl_3$) 173.4 (C10), 143.5 (C5), 137.7 (C2), 129.8 (C3), 127.1 (C4), 81.1 (C11), 64.2 (C7), 55.1 (C6), 31.4 (C9), 28.0 (C12), 26.4 (C8), 21.5 (C1); IR 3273, 2978, 1722, 1598, 1324, 1148, 1091, 980, 844, 663, 548; HRMS (ESI) calculated for $C_{16}H_{26}NO_5S$ $[M+H]^+$ 344.1526 found 344.1529; $[\alpha]_D^{24}$ +12.5 (*c* 0.50, $CHCl_3$).

***tert*-Butyl (*S*)-3-(1-((2-nitrophenyl)sulfonyl)aziridin-2-yl)propanoate 25**

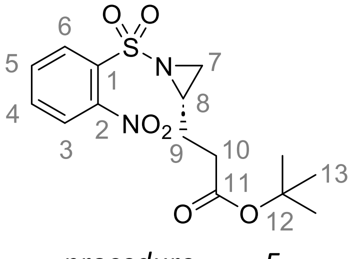

According to *general procedure F*: *tert*-butyl (*S*)-5-hydroxy-4-((4-nitrophenyl)sulfonamido)pentanoate **23** (100 mg, 0.26 mmol), methanesulfonic anhydride (70.0 mg, 0.40 mmol) and 4-DMAP (3.00 mg, 0.02 mmol) gave the *title compound* ***25*** as a pale yellow oil that was used directly in the next step without further purification;* R_f 0.40 (EtOAc/Pet 25:75); δ_H (400 MHz, $CDCl_3$) 8.19 (1H, m, H3), 7.74 (3H, m, H4, H5 & H6), 3.11-3.04 (1H, m, H8), 2.88 (1H, dd, *J* 7.0, 0.8, H7), 2.37-2.31 (3H, m, H7' & H10), 2.02-1.73 (2H, m, H9),

1.42 (9H, s, H13); δ$_C$ (100 MHz, $CDCl_3$) 170.6 (C11), 147.5 (C2), 133.5, 131.1 (2 of C4, C5, C6), 130.9 (C3), 130.2 (C1), 123.3 (1 of C4, C5, C6), 79.7 (C12), 40.0 (C8), 35.3 (C7), 31.1 (C10), 27.0 (C13), 25.7 (C9); IR 2977, 1721, 1541, 1365, 1335, 1153, 989, 851, 748, 576; HRMS (ESI) calculated for $C_{15}H_{20}N_2O_6SNa$ $[M+Na]^+$ 379.0934 found 379.0933; $[\alpha]_D^{21.0}$ +152.3 (*c* 0.50, $CHCl_3$).

* N-sulfonyl aziridines are prone to polymerisation upon standing[2,3] therefore, a small aliquot was purified and characterised and the bulk carried forward immediately as a dilute solution in CH_2Cl_2.

tert-Butyl (S)-4-((2-nitrophenyl)sulfonamido)-5-(phenylamino)pentanoate 27

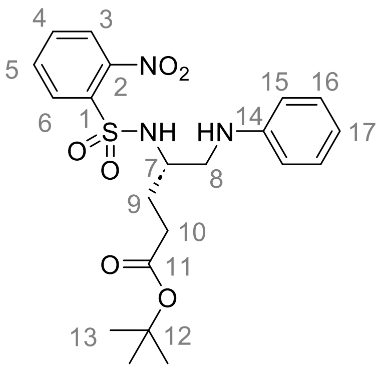

According to *general procedure G*: *tert*-Butyl (*S*)-3-(1-((4-nitrophenyl)sulfonyl)aziridin-2-yl)propanoate **25** (2.67 mmol) and aniline (2.67 mL, 1.0 M), after purification by flash column chromatography (EtOAc/Pet 15:85) gave the *title compound* ***27*** (530 mg, 1.18 mmol, 44 %) as a brown solid; R_f 0.20 (EtOAc/Pet 15:85); Mp 120 (recrystalised from $CHCl_3$); δ$_H$ (400 MHz, $CDCl_3$) 7.96 (1H, dd, *J* 7.5, 1.7, H3), 7.73 (1H, dd, *J* 7.8, 1.5, H6), 7.55 (1H, td, *J* 7.4, 1.6, H4/H5), 7.50 (1H, td, *J* 7.4, 1.6, H4/H5), 7.01 (2H, dd, *J* 8.4, 7.3, H16), 6.62 (1H, tt, *J* 8.4, 1.0, H17), 6.27 (2H, dd, *J* 8.6, 1.0, H15), 5.64 (1H, br s, NH), 3.96-3.66 (2H, br m, NH & H7), 3.20 (1H, dd, *J* 5.0, 14.1, H8), 3.08 (1H, dd, *J* 8.0, 14.1, H8'), 2.42-2.34 (2H, m, H10), 1.98-1.68 (2H, m, H9), 1.44 (9H, s, H13); δ$_C$ (100 MHz, $CDCl_3$) 172.5 (C11), 147.0 (C2), 134.5 (C1), 133.3, 132.8 (C4, C5), 130.6 (C3), 129.3 (C14), 129.2 (C16), 125.2 (C6), 117.7 (C17), 112.5 (C15), 80.8 (C12), 53.8 (C7), 47.9 (C8), 31.3 (C10), 28.5 (C9), 28.1 (C13); IR 3374, 2977, 1715, 1602, 1537, 1365, 1257, 1153, 910, 729, 592; HRMS (ESI) calculated for $C_{21}H_{28}N_3O_6S$ $[M+H]^+$ 450.1693 found 450.1701; $[\alpha]_D^{22.0}$ -66.0 (*c* 0.50, $CHCl_3$).

tert-Butyl (S)-3-(3-((2-nitrophenyl)sulfonyl)-2-oxo-1-phenylimidazolidin-4-yl)propanoate 50

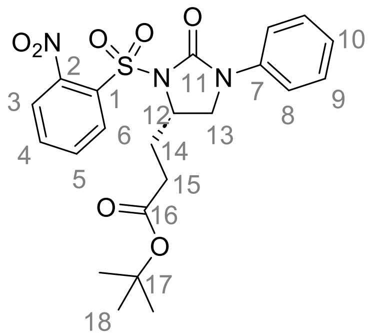

According to *general procedure I*: *tert*-Butyl (*S*)-4-((4-nitrophenyl)sulfonamido)-5-(phenylamino)pentanoate **27** (530 mg, 1.18 mmol), DIPEA (0.22 mL, 1.29 mmol) and triphosgene (140 mg, 0.47 mmol) followed by purification by flash column chromatography (EtOAc/Pet 20:80) gave the *title compound* ***50*** (370 mg, 0.77 mmol, 65%) as a brown solid; R_f 0.20 (EtOAc/Pet 20:80); Mp 68 (not recrystalised, concentrated from EtOAc); δ_H (400 MHz, $CDCl_3$) 8.50-8.44 (1H, m, H3), 7.76-7.71 (3H, m, H4, H5 & H6), 7.43-7.38 (2H, m, H8), 7.35-7.29 (2H, m, H9), 7.11 (1H, tt, *J* 8.4, 1.2 H10), 4.66-4.58 (1H, m, H12), 4.32 (1H, t, *J* 9.2, H13), 3.58 (1H, dd, *J* 9.2, 1.7, H13'), 2.58-2.34 (2H, m, H15), 2.33-2.15 (2H, m, H14), 1.43 (9H, s, H18); δ_C (100 MHz, $CDCl_3$) 171.6 (C16), 150.9 (C11), 147.9 (C2), 138.1 (C1), 135.1 (C3), 134.8, 132.0 (2 of C4, C5, C6), 131.8 (C7), 129.3 (C9), 124.6 (1 of C4, C5, C6), 124.3 (C10), 118.7 (C8), 81.1 (C17), 53.8 (C12), 48.8 (C13), 31.2 (C14), 30.7 (C15), 28.0 (C18); IR 3283, 2978, 1720, 1595, 1540, 1502, 1364, 1229, 1122, 852, 753, 594; HRMS (ESI) calculated for $C_{22}H_{26}N_3O_7S$ $[M+H]^+$ 476.1486 found 476.1497; $[\alpha]_D^{24.0}$ +177 (*c* 0.25, $CHCl_3$).

tert-Butyl (S)-3-(3-((2-nitrophenyl)sulfonyl)-2-oxoimidazolidin-4-yl)propanoate 33

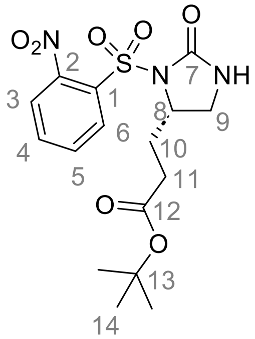

According to general procedure J: N-sulfonyl aziridine **25** (4.81 mmol), aqueous ammonium hydroxide (22%, 13.3 mL), DIPEA (0.91 mL, 5.29 mmol) and triphosgene (571 mg, 1.92 mmol) followed by flash column chromatography (EtOAc/Pet 50:50) gave the *title compound* ***33*** (513 mg, 1.28 mmol, 27%) as an orange oil; R_f 0.20 (EtOAc/Pet 50:50); δ_H (500 MHz, $CDCl_3$) 8.42-8.35 (1H, m, H3), 7.77-7.68 (3H, m, H4, H5 & H6), 5.34 (1H, br s, NH), 4.60-4.53 (1H, m, H8), 3.78 (1H, t, *J* 8.9, H9), 3.22 (1H, dd, *J* 9.2, 2.2, H9'), 2.49-2.34 (2H, m, H11), 2.24-2.10 (2H, m, H10), 1.45 (9H, s, H14); δ_C (125 MHz, $CDCl_3$) 171.8 (C12), 155.3 (C7), 148.0 (C2), 134.9 (C3), 134.8 (1 of C4, C5, C6), 132.1 (C1), 132.0 (1 of C4, C5, C6), 124.3 (1 of C4, C5, C6), 81.1 (C13), 57.5 (C8), 43.9 (C9) 31.2 (C10) 30.9 (C11) 28.2 (C14); IR 3322, 2978, 1723, 1541, 1364, 1168,

1151, 1127, 729, 587; HRMS (ESI) calculated for $C_{16}H_{21}N_3O_7SNa$ $[M+Na]^+$ 422.0992 found 422.0998; $[\alpha]_D^{23.0}$ +331 (*c* 0.50, $CHCl_3$).

tert-Butyl (S)-3-(1-(6-bromopyridin-2-yl)-3-((2-nitrophenyl)sulfonyl)-2-oxoimidazolidin-4-yl)propanoate 35

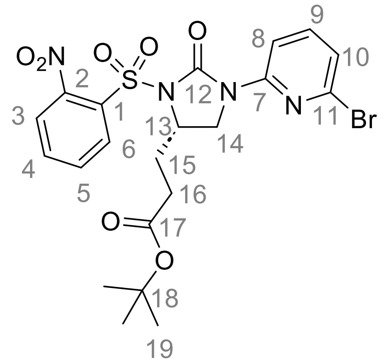

According to general procedure L: cyclic urea **33** (200 mg, 0.50 mmol), Cs_2CO_3 (325 mg, 1.00 mmol), $Pd(dba)_2$ (14.0 mg, 5 mol%), Xantphos (43 mg, 15 mol%) and 2,6-dibromopyridine (142 mg, 0.60 mmol) followed by flash column chromatography (EtOAc/Pet 20:80 to 50:50) gave the *title compound* ***35*** (218 mg, 0.39 mmol, 79%) as an off white solid; R_f 0.25 (EtOAc/Pet 50:50); Mp 172-174 (not recrystalised, concentrated from EtOAc); δ_H (400 MHz, $CDCl_3$) 8.50-8.43 (1H, m, H3), 7.96 (1H, dd, *J* 8.3, 0.7, H8), 7.80-7.71 (3H, m, H4, H5 & H6), 7.46 (1H, dd, *J* 8.3, 7.7, H9), 7.17 (1H, dd, *J* 7.7, 0.7, H10), 4.66-4.58 (1H, m, H13), 4.31 (1H, dd, *J* 11.1, 8.8, H14), 3.96 (1H, dd, *J* 11.1, 2.0, H14'), 2.56-2.35 (2H, m, H16), 2.34-2.14 (2H, m, H15), 1.45 (9H, s, H19); δ_C (100 MHz, $CDCl_3$) 171.4 (C17), 150.8 (C12), 150.8 (C7), 148.2 (C2), 139.9 (C9), 139.5 (C11), 135.1 (1 of C4, C5, C6), 135.0 (C3), 132.1 (1 of C4, C5, C6), 131.7 (C1), 124.6 (1 of C4, C5, C6), 123.4 (C10), 112.1 (C8), 81.2 (C18), 54.3 (C13), 47.7 (C14), 31.4 (C16), 30.9 (C15), 28.1 (C19); IR 2975, 2918, 1732, 1717, 1557, 1472, 1373, 1133, 982, 789, 713, 594, 563; HRMS (ESI) calculated for $C_{21}H_{23}{}^{79}BrN_4O_7SNa$ $[M+Na]^+$ 577.0363 found 577.0371; $[\alpha]_D^{22.0}$ +255 (*c* 0.50, $CHCl_3$).

tert-Butyl (S)-3-(2-oxo-1-phenylimidazolidin-4-yl)propanoate 29

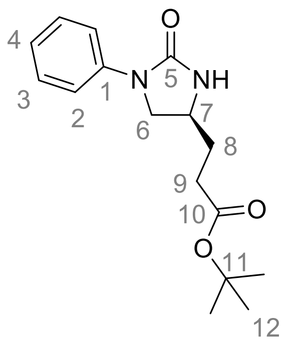

According to general procedure M: tert-butyl (*S*)-3-(3-((2-nitrophenyl)sulfonyl)-2-oxo-1-phenylimidazolidin-4-yl)propanoate **50** (300 mg, 0.63 mmol), K_2CO_3 (261 mg, 1.89 mmol) and thiophenol (0.09 mL, 0.94 mmol) followed by flash column chromatography (EtOAc/Pet 60:40) gave the *title compound **29*** (83.0 mg, 0.28 mmol, 45 %) as a pale brown oil; R_f 0.60 (EtOAc/Pet 50:50); δ_H (400 MHz, $CDCl_3$) 7.52 (2H, dd, *J* 8.8, 1.1, H2), 7.32 (2H, dd, *J* 8.7, 7.4, H3), 7.04 (1H, dt, *J* 7.4, 1.1, H4), 5.58 (1H, br s, NH), 4.01 (1H, t, *J* 8.9, H6) 3.89-3.81 (1H, m, H7), 3.53 (1H, dd, *J* 9.0, 6.2, H6') 2.35 (2H, t, *J* 7.5, H9) 2.00-1.83 (2H, m, H8) 1.45 (9H, s, H12); δ_C (100 MHz, $CDCl_3$) 172.2 (C10), 159.0 (C5), 140.1 (C1), 128.9 (C3), 122.8 (C4), 117.9 (C2), 81.1 (C11), 50.8 (C6), 48.5 (C7), 31.4 (C9), 31.2 (C8), 28.2 (C12); IR 3350, 2979, 2931, 1697, 1598, 1480, 1409, 1394, 1149, 1035, 753, 689, 602; HRMS (ESI) calculated for $C_{16}H_{22}N_2O_3Na$ $[M+Na]^+$ 313.1523 found 313.1521, $C_{16}H_{23}N_2O_3$ $[M+H]^+$ 291.1703 found 291.1700; $[\alpha]_D^{22.0}$ -35.2 (*c* 0.25, $CHCl_3$).

tert-Butyl 3-((S)-1-(6-((S)-5-(3-(tert-butoxy)-3-oxopropyl)-2-oxo-3-phenylimidazolidin-1-yl)pyridin-2-yl)-3-((2-nitrophenyl)sulfonyl)-2-oxoimidazolidin-4-yl)propanoate 37

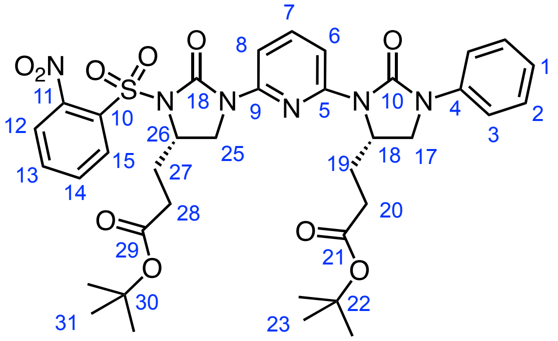

According to general procedure L: tert-Butyl (*S*)-3-(1-(6-bromopyridin-2-yl)-3-((2-nitrophenyl)sulfonyl)-2-oxoimidazolidin-4-yl)propanoate **35** (115 mg, 0.20 mmol), Cs_2CO_3 (112 mg, 0.34 mmol), $Pd(dba)_2$ (5.0 mg, 5 mol%), Xantphos (15 mg, 15 mol%) and *tert*-Butyl (*S*)-3-(2-oxo-1-phenylimidazolidin-4-yl)propanoate **29** (50 mg, 0.17 mmol) followed by flash column chromatography (EtOAc/Pet 20:80 to 30:70) gave the *title compound **37*** (98 mg, 0.12 mmol, 74 %) as a pale brown oil; R_f 0.4 (EtOAc/Pet 35:65); δ_H (400 MHz, $CDCl_3$) 8.52-8.45 (1H, m, H12), 8.00-7.93 (1H, m, H7), 7.80-7.71 (3H, m, H13, H14 & H15), 7.65-7.59 (2H, m, H6 & H8), 7.59-7.54 (2H, m, H3), 7.41-7.34 (2H, m, H2), 7.11 (1H, tt, *J* 7.4, 1.1, H1), 4.81- 4.70 (1H, m, H18), 4.67- 4.57 (1H, m, H26), 4.37 (1H, dd, *J* 10.8, 8.9, H25), 4.06 (1H, t, *J* 9.2, H17), 3.98 (1H, dd, *J* 10.8, 1.9, H25'), 3.62 (1H, dd, *J* 9.2, 3.3, H17'), 2.63-1.94 (8H, m, H19, H20, H27 & H28), 1.44 (9H, s, H23/H31), 1.41 (9H, s, H23/ H31); δ_C (100 MHz, $CDCl_3$) 171.9, 171.6 (C29, C21), 154.2, 151.0 (C24, C16), 150.0 (1 of C9, C5), 148.9 (C11), 148.2 (1 of C9, C5), 139.7 (1 of C8, C6), 139.6 (C4), 135.2 (C12), 135.0, 132.2 (1 of C15, C14, C13), 132.0 (C10), 129.1 (C2), 124.5 (1 of C15, C14, C13), 123.7 (C1), 118.6 (C3), 109.4 (C7), 107.8 (1 of C8, C6), 81.0, 81.0 (C30, C22), 54.2 (C26), 50.8 (C18), 47.7 (C25), 47.2 (C17), 31.5, 30.8, 30.6, 28.3 (C28, C27, C20, C19), 28.2, 28.1 (C31, C23); IR 2976, 2360, 1718, 1671, 1542, 1448, 1391, 1364, 1290, 1247,

1148, 1119, 797, 745, 595; HRMS (ESI) calculated for $C_{37}H_{44}N_6O_{10}SNa$ $[M+Na]^+$ 787.2732 found 787.2738, $C_{37}H_{45}N_6O_{10}S$ $[M+H]^+$ 765.2912 found 765.2921; $[\alpha]_D^{22\,°C}$ +130 (*c* 0.50, $CHCl_3$).

tert-Butyl 3-((_S_)-1-(6-((_S_)-5-(3-(_tert_-butoxy)-3-oxopropyl)-2-oxo-3-phenylimidazolidin-1-yl)pyridin-2-yl)-2-oxoimidazolidin-4-yl)propanoate 38

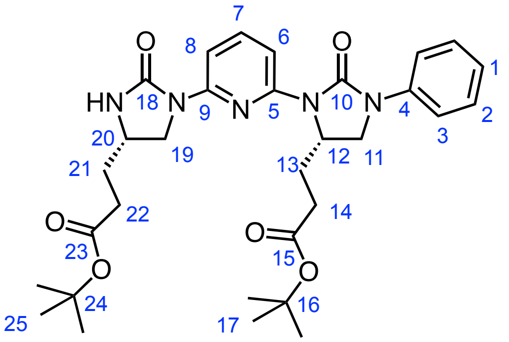

According to general procedure M: tert-Butyl 3-((*S*)-1-(6-((*S*)-5-(3-(*tert*-butoxy)-3-oxopropyl)-2-oxo-3-phenylimidazolidin-1-yl)pyridin-2-yl)-3-((2-nitrophenyl)sulfonyl)-2-oxoimidazolidin-4-yl)propanoate **37** (50 mg, 0.07 mmol), K_2CO_3 (27 mg, 0.19 mmol) and thiophenol (0.01 mL, 0.10 mmol) followed by flash column chromatography (EtOAc/Pet 50:50) gave the *title compound **38*** (22.0 mg, 0.03 mmol, 58%) as a pale brown oil; R_f 0.50 (EtOAc/Pet 50:50); δ_H (400 MHz, $CDCl_3$) 7.87 (1H, t, *J* 7.3, H7), 7.63 (1H, t, *J* 8.1, H6/H8), 7.60-7.56 (2H, m, H2/H3), 7.47-7.34 (3H, m, H2/H3 + H6/H8), 7.10 (1H, tt, *J* 7.4, 1.1, H1), 5.29 (1H, br s, NH), 4.83-4.75 (1H, m, H12), 4.25 (1H, dd, *J* 10.5, 8.9, H19), 4.06 (1H, t, *J* 9.1, H11), 3.88-3.79 (1H, m, H20), 3.72 (1H, dd, *J* 10.5, 6.4, H19'), 3.61 (1H, dd, *J* 9.1, 3.3, H11'), 2.44-2.23 (5H, m, H13/H14/H21/H22), 2.14-1.84 (3H, m, H13/H14/H21/H22), 1.45 (9H, s, H17/H25), 1.40 (9H, s, H17/H25); δ_C (100 MHz, $CDCl_3$) 172.3, 172.1 (C23, C15), 158.2 (C10), 154.4 (C18), 150.5 (1 of C9, C5), 149.6 (C4), 139.8 (1 of C9, C5), 139.4 (C8, C6), 129.1 (C3, C2), 123.5 (C1), 118.5 (1 of C3, C2), 107.7 (1 of C8, C6), 107.1 (C7), 81.1, 81.0 (C24, C16), 50.7 (C12), 49.6 (C19), 48.4 (C20), 47.3 (C11), 31.5, 31.2, 30.7, 28.4 (C22, C21, C14, C13), 28.2, 28.2 (C25, C17); IR 3327, 2976, 1709, 1582, 1449, 1395, 1285, 1145, 1028, 844, 797, 752, 690, 626; HRMS (ESI) calculated for $C_{31}H_{41}N_5O_6Na$ $[M+Na]^+$ 602.2949 found 602.2946, $C_{31}H_{42}N_5O_6$ $[M+H]^+$ 580.3130 found 580.3131; $[\alpha]_D^{22.0}$ -24.8 (*c* 1.00, $CHCl_3$).

tert-butyl 3-((_S_)-1-(6-((_S_)-5-(3-(_tert_-butoxy)-3-oxopropyl)-2-oxo-3-phenylimidazolidin-1-yl)pyridin-2-yl)-3-(6-((_S_)-4-(3-(_tert_-butoxy)-3-oxopropyl)-3-((4-nitrophenyl)sulfonyl)-2-oxoimidazolidin-1-yl)pyridin-2-yl)-2-oxoimidazolidin-4-yl)propanoate 51

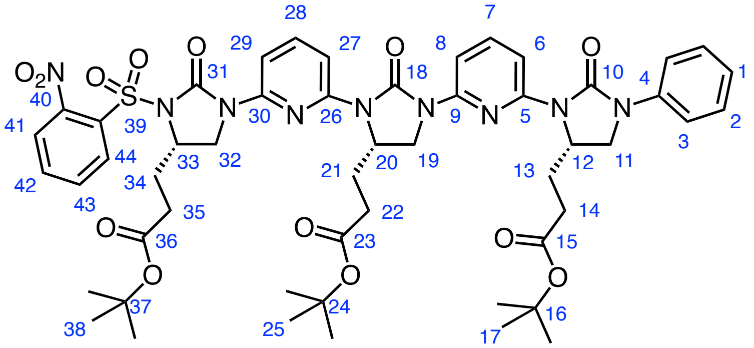

According to general procedure L: tert-butyl 3-((*S*)-1-(6-((*S*)-5-(3-(*tert*-butoxy)-3-oxopropyl)-2-oxo-3-phenylimidazolidin-1-yl)pyridin-2-yl)-2-oxoimidazolidin-4-yl)propanoate **38** (40 mg, 0.06 mmol), Cs_2CO_3 (45.0 mg, 0.13 mmol), $Pd(dba)_2$ (2.0 mg, 5 mol%), Xantphos (6.0 mg, 15 mol%) and *tert*-butyl (*S*)-3-(1-(6-bromopyridin-2-yl)-3-((2-nitrophenyl)sulfonyl)-2-oxoimidazolidin-4-yl)propanoate **35** (38 mg, 0.06 mmol) followed by flash column chromatography (EtOAc/Pet 30:70) gave the *title compound* ***51*** (26.0 mg, 0.02 mmol, 36%) as an off white film; R_f 0.6 (EtOAc/Pet 30:70); δ_H (500 MHz, $CDCl_3$) 8.51-8.47 (1H, m, H41), 7.98-7.92 (2H, m, pyr-H), 7.90 (1H, dd, *J* 0.6, 8.2, pyr-H), 7.78-7.73 (3H, m, H44, H43, H42), 7.68 (1H, t, *J* 8.2, pyr-H), 7.64-7.62 (2H, m, pyr-H), 7.60-7.57 (2H, dd, *J* 8.6, 1.1, H3), 7.38 (2H, dd, *J* 8.6, 7.5, H2), 7.12 (1H, tt, *J* 7.5, 1.1, H1), 4.84-4.77 (1H, m, H12), 4.77-4.70 (1H, m, H33, H20), 4.67-4.61 (1H, m, H33, H20), 4.38 (1H, dd, *J* 10.8, 9.0, H32, H19), 4.17 (1H, dd, *J* 10.8, 9.3, H32, H19), 4.09 (1H, t, *J* 9.1, H11), 4.07 (1H, dd, *J* 10.8, 1.9, H32, H19), 3.94 (1H, dd, *J* 10.8, 3.5, H32, H19), 3.64 (1H, dd, *J* 9.0, 3.2, H11’), 2.64-2.54 (1H, m, CH_2CH_2), 2.50-2.22 (9H, m, CH_2CH_2), 2.15-2.04 (1H, m, CH_2CH_2), 2.04-1.96 (1H, m, CH_2CH_2), 1.44 (9H, s, O*t*-Bu), 1.43 (9H, s, O*t*-Bu), 1.41 (9H, s, O*t*-Bu); δ_C (125 MHz, $CDCl_3$) 172.0, 172.0, 171.6 (C36, C23, C15), 154.3 (C10), 154.0, 151.1 (C31, C18), 150.1, 149.9, 149.9, 149.0 (C30, C26, C9, C5), 148.1 (C40), 139.7 (pyr-**C**H), 139.7 (C4), 139.6 (pyr-**C**H), 135.2 (C41), 135.0, 132.2 (2 of C44, C43, C42), 131.9 (C39), 129.1 (C2), 124.5 (1 of C44, C43, C42), 123.6 (C1), 118.5 (C3), 109.7, 108.5, 108.2, 107.7 (4 x pyr-**C**H), 80.9, 80.9, 80.8 (3 x O**C**(CH_3)$_3$), 54.2 (1 of C33, C20), 50.9, 50.8 (2 of C33, C20, C12), 47.7 (1 of C32, C19), 47.2 (C11), 45.8 (1 of C32, C19), 31.5, 30.8, 30.7, 30.4, 28.5, 28.4 (3 x CH_2CH_2), 28.3, 28.2, 28.2 (3 x OC(**C**H_3)$_3$); IR 2923, 2360, 1720, 1582, 1446, 1390, 1286, 1152, 798, 747, 597; HRMS (ESI) calculated for $C_{52}H_{64}N_9O_{13}S$ $[M+H]^+$ 1054.4339 found 1054.4339; $[\alpha]_D^{21.0}$ +130 (*c* 0.50, $CHCl_3$).

3-((S)-1-(6-((S)-5-(2-Carboxyethyl)-2-oxo-3-phenylimidazolidin-1-yl)pyridin-2-yl)-3-(6-((S)-4-(2-carboxyethyl)-3-((2-nitrophenyl)sulfonyl)-2-oxoimidazolidin-1-yl)pyridin-2-yl)-2-oxoimidazolidin-4-yl)propanoic acid 39

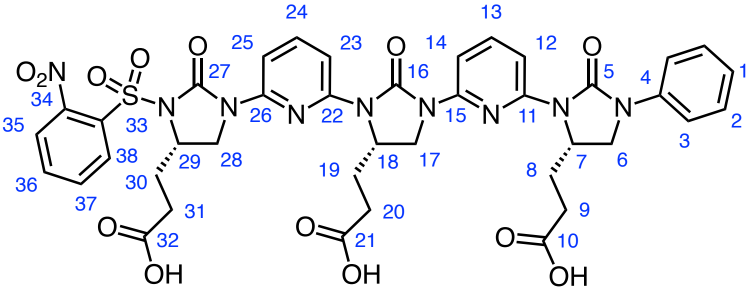

According to general procedure N: tert-Butyl 3-((*S*)-1-(6-((*S*)-5-(3-(*tert*-butoxy)-3-oxopropyl)-2-oxo-3-phenylimidazolidin-1-yl)pyridin-2-yl)-3-(6-((*S*)-4-(3-(*tert*-butoxy)-3-oxopropyl)-3-((4-nitrophenyl)sulfonyl)-2-oxoimidazolidin-1-yl)pyridin-2-yl)-2-oxoimidazolidin-4-yl)propanoate **51** (8.0 mg, 0.007 mmol) and TFA (0.1 mL, 0.015 mmol) gave the *title compound* **39** (4.0 mg, 0.004 mmol, 60%) as an off white film; R_f 0.0 (EtOAc/Pet 30:70); δ_H (500 MHz, CD_3OD) 8.41-8.38 (1H, m, H35), 7.90-7.86 (3H, m, H38, H37, H36), 7.84-7.79 (2H, m, 2 of H23, H14, H12), 7.79-7.76 (1H, m, 1 of H23, H14, H12), 7.74-7.69 (1H, m, H24 or H13), 7.56-7.50 (2H, m, H3), 7.50-7.44 (2H, m, H25 and 1 of H23, H14, H12), 7.34-7.28 (2H, m, H2), 7.07-7.02 (1H, m, H1), 4.74-4.63 (2H, m, H18, H7), 4.66-4.57 (1H, m, H29), 4.37-4.29 (1H, m, H28), 4.10-4.00 (2H, m, H28’, H17’), 3.99-3.91 (1H, m, H6’), 3.90-3.84 (1H, dd, *J* 10.5, 10.3, H17), 3.66-3.58 (1H, m, H6), 2.63-2.47 (2H, m, 2H, H31, H20, H9), 2.46-2.31 (6H, m, H31, H30, H20, H19, H9, H8), 2.27-2.19 (2H, m, H30, H19, H8), 2.01-1.93 (1H, m, H31, H20, H9), 1.89-1.79 (1H, m, H31, H20, H9); δ_C (125 MHz, CD_3OD) 176.5, 176.4, 176.3 (C32, C21, C10), 155.7 (C5), 155.0, 155.0 (C27, C16), 152.1, 152.2, 152.1, 150.9 (C26, C22, C15, C11), 149.3 (C34), 140.8 (C4), 140.8, 140.4 (C24, C13), 136.8 (C37), 135.5 (C35), 133.2 (C36), 132.2 (C33), 129.9 (C2), 125.9 (C38), 124.7 (C1), 120.1 (C3), 110.3 (C25), 108.7, 108.4, 107.9 (C23, C14, C12), 55.4 (C29), 52.4 (C7), 51.9 (C18), 48.7 (C6), 48.4 (C28), 46.9 (C17), 32.8, 32.7, 32.0, 31.9, 31.6, 30.3 (3 x CH_2CH_2); IR 3357, 2916, 2848, 2359, 1716, 1636, 1583, 1541, 1447, 1391, 1365, 1287, 1157, 756, 597; HRMS (ESI) calculated for $C_{40}H_{40}N_9O_{13}S$ $[M+H]^+$ found 886.2595; $[\alpha]_D^{22.0}$ +19.0 (*c* 0.20, CH_3OH).

(*S*)-*N*-(1-Hydroxy-4-methylpentan-2-yl)-2-nitrobenzenesulfonamide 24

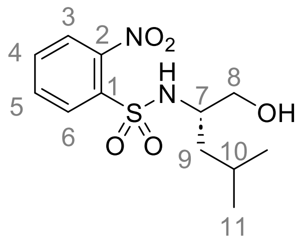

According to general procedure D: L-leucinol **22** (5.40 ml, 42.7 mmol), Et_3N (5.90 mL, 51.2 mmol), and 2-nitrobenzenesulfonyl chloride (11.3 g, 51.2 mmol) followed by flash column chromatography (EtOAc/Pet 20:80) gave the *title compound **24*** (7.96 g, 25.4 mmol, 61%) as a colourless oil; R_f 0.4 (EtOAc/Pet 20:80); δ_H (400 MHz, $CDCl_3$) 8.16-8.10 (1H, m, H3), 7.85-7.80 (1H, m, H5), 7.75-7.68 (2H, m, H4 & 6), 5.59 (1H, br d, *J* 7.6, NH), 3.60-3.44 (3H, m, H7 & H8), 2.37 (1H, br s, OH), 1.60-1.45 (1H, m, H10), 1.41-1.25 (2H, m, H9), 0.79 (3H, d, *J* 6.6, H11), 0.70 (3H, d, *J* 6.6, H11); δ_C (100 MHz, $CDCl_3$) 147.7 (C2), 134.7 (C1), 133.6, 133.0 (C4, C6), 130.7 (C3), 125.4 (C5), 65.2 (C8), 55.1 (C7), 40.9 (C9), 24.4 (C10), 22.9 (C11), 21.8 (C11); $[\alpha]_D^{24.0}$ +46.4 (*c* 1.00, $CHCl_3$).

(*S*)-2-Isobutyl-1-((2-nitrophenyl)sulfonyl)aziridine 26

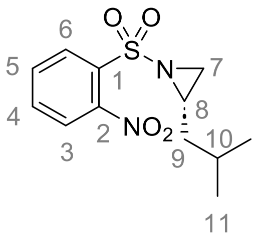

According to *general procedure F*: (*S*)-*N*-(1-hydroxy-4-methylpentan-2-yl)-2-nitrobenzenesulfonamide **24** (5.00 g, 16.5 mmol), methanesulfonic anhydride (4.32 g, 24.8 mmol) and 4-DMAP (201 mg,1.65 mmol) gave the *title compound **26*** as a pale yellow oil that was used directly in the next step without further purification;* R_f 0.60 (EtOAc/Pet 60:40); δ_H (500 MHz, $CDCl_3$) 8.21-8.18 (1H, m, H3), 7.78-7.70 (3H, m, H4, H5 & H6), 3.08-3.02 (1H, m, H8), 2.90 (1H, d, *J* 7.0, H7), 2.26 (1H, d, *J* 4.9, H7'), 1.81-1.72 (1H, m, H10), 1.59-1.52 (1H, m, H9), 1.37-1.30 (1H, m, H9'), 1.42 (6H, dd, *J* 2.8, 6.6, H11); δ_C (125 MHz, $CDCl_3$) 148.5 (C2), 134.3 (1 of C4, C5, C6), 132.2 (C1), 132.0 (1 of C4, C5, C6), 131.1 (C3), 124.3 (1 of C4, C5, C6), 41.0 (C8), 40.6 (C9), 36.6 (C7), 26.7 (C10), 22.7 (C11), 22.1 (C11); IR 2958, 1541, 1367, 1330, 1161, 1057, 931, 850, 778, 732, 653, 598; HRMS (ESI) calculated for $C_{12}H_{17}N_2O_4S$ $[M+H]^+$ 285.0904 found 285.0907; $[\alpha]_D^{22.0}$ +54.0 (*c* 0.50, $CHCl_3$).

* N-sulfonyl aziridines are prone to polymerisation upon standing[2,3] therefore, a small aliquot was purified and characterised and the bulk carried forward immediately as a dilute solution in CH_2Cl_2.

(*S*)-5-Isobutyl-1-((2-nitrophenyl)sulfonyl)imidazolidin-2-one 34

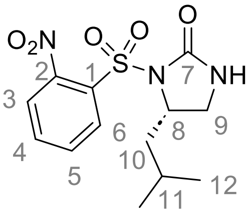

According to general procedure J: (*S*)-2-Isobutyl-1-((2-nitrophenyl)sulfonyl)aziridine **26** (3.27 mmol), aqueous ammonium hydroxide (22%, 9.0 mL), DIPEA (0.62 mL, 3.59 mmol) and triphosgene (388 mg, 1.30 mmol) followed by flash column chromatography (EtOAc/CH_2Cl_2 20:80) gave the *title compound* ***34*** (220 mg, 0.67 mmol, 21%) as a yellow solid; R_f 0.30 (EtOAc/CH_2Cl_2 20:80); Mp 137-138 (not recrystalised, concentrated from EtOAc); δ_H (400 MHz, $CDCl_3$) 8.33-8.28 (1H, m, H3), 7.74-7.63 (3H, m, H4, H5 & H6), 6.05 (1H, br s, NH), 4.52-4.42 (1H, m, H8), 3.68 (1H, t, *J* 8.8, H9), 3.17 (1H, ddd, *J* 9.1, 2.3, 1.1, H9'), 1.87-1.78 (1H, m, H10), 1.78- 1.71 (1H, m, H10'), 1.71-1.61 (1H, m, H11), 0.96 (6H, dd, *J* 6.5, 1.3, H12); δ_C (100 MHz, $CDCl_3$) 155.4 (C7), 147.8 (C2), 134.6 (1 of C4, C5, C6), 134.2 (C3), 132.1 (C1), 131.9, 124.2 (1 of C4, C5, C6), 57.0 (C8), 44.8 (C10), 44.0 (C9), 24.7 (C11), 23.4 (C12), 21.7 (C12); IR 3350, 2957, 1740, 1698, 1547, 1367, 1170 1130, 1070, 855, 777, 730, 654, 586, 543; HRMS (ESI) calculated for $C_{13}H_{18}N_3O_5S$ $[M+H]^+$ 328.0962 found 328.0967; $[\alpha]_D^{22.0}$ +500 (*c* 1.00, $CHCl_3$).

(*S*)-1-(6-Bromopyridin-2-yl)-4-isobutyl-3-((4-nitrophenyl)sulfonyl)imidazolidin-2-one 36

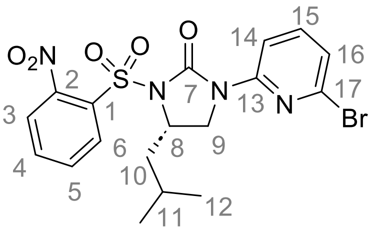

According to general procedure L: (*S*)-5-isobutyl-1-((2-nitrophenyl)sulfonyl)imidazolidin-2-one **34** (300 mg, 0.62 mmol), Cs_2CO_3 (404 mg, 1.24 mmol), $Pd(dba)_2$ (18 mg, 5 mol%), Xantphos (54.0 mg, 15 mol%) and 2,6-dibromopyridine (176 mg, 0.74 mmol) followed by flash column chromatography (EtOAc/Pet 40:60) gave the *title compound* **36** (12.0 mg, 0.02 mmol, 3 %) as a yellow solid; R_f 0.50 (EtOAc/Pet 50:50); Mp 162 (not recrystalised, concentrated from EtOAc); δ_H (400 MHz, $CDCl_3$) 8.48-8.42 (1H, m H3), 7.97 (1H, dd, *J* 8.3, 0.6, H14), 7.80-7.71 (3H, m, H4, H5 & H6), 7.46 (1H, dd, *J* 8.3, 7.8, H15), 7.16 (1H, dd, *J* 7.7, 0.6, H16), 4.61-4.52 (1H, m, H8), 4.28 (1H, dd, *J* 10.6, 8.6, H9), 3.93 (1H, dd, *J* 10.8, 2.0, H9'), 1.98-1.86 (1H, m, H10), 1.86-1.74 (2H, m, H10' & H11), 1.03 (6H, dd, *J* 8.6, 6.4, H12); δ_C (100 MHz, $CDCl_3$) 151.1 (C13), 151.0 (C7), 148.1 (C2), 139.9 (C15), 139.5 (C17), 135.0 (1 of C4, C5, C6), 134.9 (C3), 132.1 (1 of C4, C5, C6), 131.8 (C1), 124.6 (1 of C4, C5, C6), 123.3 (C16), 112.2 (C14), 53.8 (C8), 47.9 (C9), 45.1 (C10), 24.8 (C11), 23.6, 21.7 (2 x C12); IR 2955, 2925, 2866, 1731, 1545, 1436, 1365, 1240,

1157, 1115, 980, 846, 781, 716, 656, 596, 558; HRMS (ESI) calculated for $C_{18}H_{20}{}^{79}BrN_4O_5S$ $[M+H]^+$ 483.0332 found 483.0335; $[\alpha]_D^{23.0}$ +295 (*c* 0.50, $CHCl_3$).

tert-Butyl 3-((_S_)-1-(6-((_S_)-5-(3-(_tert_-butoxy)-3-oxopropyl)-2-oxo-3-phenylimidazolidin-1-yl)pyridin-2-yl)-3-(6-((_S_)-4-isobutyl-3-((4-nitrophenyl)sulfonyl)-2-oxoimidazolidin-1-yl)pyridin-2-yl)-2-oxoimidazolidin-4-yl)propanoate 52

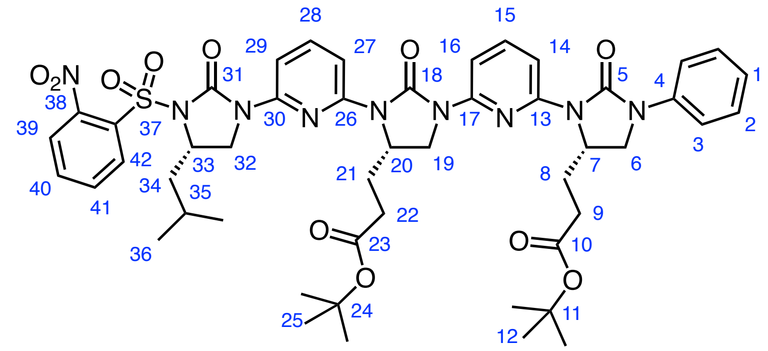

According to general procedure L: tert-Butyl 3-((*S*)-1-(6-((*S*)-5-(3-(*tert*-butoxy)-3-oxopropyl)-2-oxo-3-phenylimidazolidin-1-yl)pyridin-2-yl)-2-oxoimidazolidin-4-yl)propanoate **38** (17 mg, 0.02 mmol), Cs_2CO_3 (16 mg, 0.04 mmol), $Pd(dba)_2$ (1.0 mg, 5 mol%), Xantphos (1.0 mg, 15 mol%) and (*S*)-1-(6-bromopyridin-2-yl)-4-isobutyl-3-((4-nitrophenyl)sulfonyl)imidazolidin-2-one **36** (12 mg, 0.02 mmol) followed by flash column chromatography (EtOAc/Pet 0:100 to 30:70) gave the *title compound* ***52*** (6.0 mg, 0.006 mmol, 17%) as a pale brown film; R_f 0.6 (EtOAc/Pet 30:70); δ_H (500 MHz, $CDCl_3$) 8.51 (1H, m, H39), 7.95 (1H, dd, *J* 6.4, 2.4, pyr-H), 7.94 (1H, dd, *J* 7.2, 0.6, pyr-H), 7.90 (1H, dd, *J* 8.2, 0.6, pyr-H), 7.79 (3H, m, H40, H41 & H42), 7.68 (1H, t, *J* 8.2, pyr-H), 7.66-7.61 (2H, m, pyr-H), 7.59 (2H, dd, *J* 8.8, 1.1, H3), 7.38 (2H, dd, *J* 8.8, 7.4, H2), 7.12 (1H, tt, *J* 7.4, 1.1, H1) 4.83-4.76 (1H, m, H7), 4.76-4.70 (1H, m, H20), 4.61-4.52 (1H, m, H33), 4.35 (1H, dd, *J* 10.7, 8.6, H32), 4.18 (1H, dd, *J* 10.6, 9.3, H19), 4.09 (1H, t, *J* 9.1, H6), 3.97-3.90 (2H, m, H19' & H32'), 3.88 (1H, dd, *J* 9.1, 3.3, H6'), 2.46-2.26 (5H, m, CH_2CH_2), 2.16-1.80 (6H, m, CH_2CH_2), 1.42 (18H, d, *J* 2.2, H12 & H25), 0.82 (6H, d, *J* 6.7, H36); δ_C (125 MHz, $CDCl_3$) 172.0, 171.9 (C23, C10), 154.3 (C5), 154.0 (C18), 151.1 (C31), 150.1, 149.9, 149.9, 149.2 (4 x pyr-**C**), 148.1 (C38), 139.7 (pyr-**C**H), 139.7 (C4), 139.6 (pyr-**C**H), 135.1 (C39), 134.9 (1 of C40, C41, C42), 132.2 (C37), 132.1 (1 of C42, C41, C40), 129.1 (C2), 124.6 (1 of C42, C41, C40), 123.7 (C1), 118.6 (C3), 109.5, 108.5, 108.2, 107.8 (4 x pyr-**C**H), 81.0, 80.8 (C24, C11), 53.8 (C33), 51.1 (C35), 50.9, 50.8 (C20, C7), 48.0 (C32), 47.3 (C6), 45.8 (C19), 45.3, 30.8, 30.5, 28.6, 28.5 (C8, C9, C21, C22, C34), 28.2, 28.2 (C25, C12), 27.8 (C36); IR 3353, 2980, 2359, 1722, 1582, 1446, 1390, 1286, 1152, 955, 798, 587; HRMS (ESI) calculated for $C_{49}H_{60}N_9O_{11}S$ $[M+H]^+$ 982.4128 found 982.4135; $[\alpha]_D^{21.0}$ +161 (*c* 0.25, $CHCl_3$).

3-((S)-1-(6-((S)-5-(2-Carboxyethyl)-2-oxo-3-phenylimidazolidin-1-yl)pyridin-2-yl)-3-(6-((S)-4-isobutyl-3-((2-nitrophenyl)sulfonyl)-2-oxoimidazolidin-1-yl)pyridin-2-yl)-2-oxoimidazolidin-4-yl)propanoic acid 40

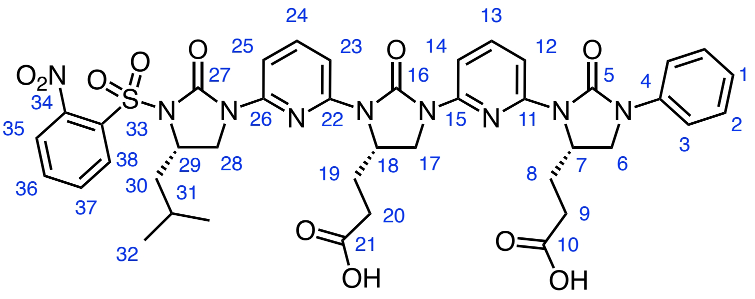

According to general procedure N: tert-Butyl 3-((*S*)-1-(6-((*S*)-5-(3-(*tert*-butoxy)-3-oxopropyl)-2-oxo-3-phenylimidazolidin-1-yl)pyridin-2-yl)-3-(6-((*S*)-4-isobutyl-3-((4-nitrophenyl)sulfonyl)-2-oxoimidazolidin-1-yl)pyridin-2-yl)-2-oxoimidazolidin-4-yl)propanoate **52** (5.0 mg, 0.005 mmol) and TFA (0.03 mL, 0.005 mmol) gave the *title compound* ***40*** (3.00 mg, 0.003 mmol, 68%) as an off-white film; R_f 0.0 (EtOAc/Pet 30:70); δ_H (500 MHz, CD_3OD) 8.41-8.39 (1H, m, H35), 7.92 – 7.87 (3H, m, H36, H37, H38), 7.87-7.79 (3H, m, 3 x pyr-H), 7.73 (1H, t, *J* 8.1, H3), 7.57 (2H, t, *J* 8.1, H3), 7.52 (2H, t, *J* 8.2, pyr-H), 7.35 (2H, t, *J* 8.2, H2), 7.09 (1H, t, *J* 7.4, H1), 4.69 (2H, m, H7, H18), 4.58 (1H, t, *J* 8.8, H29), 4.36 (1H, dd, *J* 10.9, 8.9, H28), 4.09 (1H, dd, *J* 9.6, 9.3, H17), 3.99 (2H, m, H28, H6), 3.90 (1H, dd, *J* 10.7, 3.9, H17’), 3.67 (1H, dd, *J* 9.0, 2.6, H6’), 2.50-2.33 (6H, m, H9, H20, H30), 2.03 (5H, m, H8, H19, H31); δ_C (125 MHz, $CDCl_3$) 177.2, 177.1 (C21, C10), 156.6 (C5), 155.9 (C16), 153.0 (C27), 151.7, 151.5, 151.4, 151.2 (4 x pyr-C), 150.2 (C34), 141.3 (C4), 140.4 (C24), 137.6 (C13), 136.2 (C35), 134.1 (C38), 133.4 (C33), 130.7 (C2), 126.7 (C37), 125.6 (C1), 120.9 (C3), 110.0, 109.6, 109.2, 108.8 (4 x pyr-**C**H), 55.7 (C29), 53.3 (C7), 53.0 (C18), 49.9 (C28), 49.4 (C6), 47.8 (C17), 47.1 (C30), 31.6, 31.0, 30.3, 30.2 (2 x CH_2CH_2), 26.5 (C31), 24.8, 23.1 (2 x C32); IR ($CHCl_3$) 3384, 2919, 2849, 2359, 2342, 1716, 1582, 1541, 1447, 1391, 1287, 1257, 1155, 798, 597; HRMS (ESI) calculated for $C_{41}H_{44}N_9O_{13}S$ $[M+H]^+$ 870.2876 found 870.2874; $[\alpha]_D^{22.0}$ +44.0 (*c* 0.10, CH_3OH).

2. NMR Spectra of Synthetic Compounds

(S)-(9H-Fluoren-9-yl)methyl tert-butyl (6-hydroxyhexane-1,5-diyl)dicarbamate 45

^{1}H, 400 MHz, CDCl$_3$

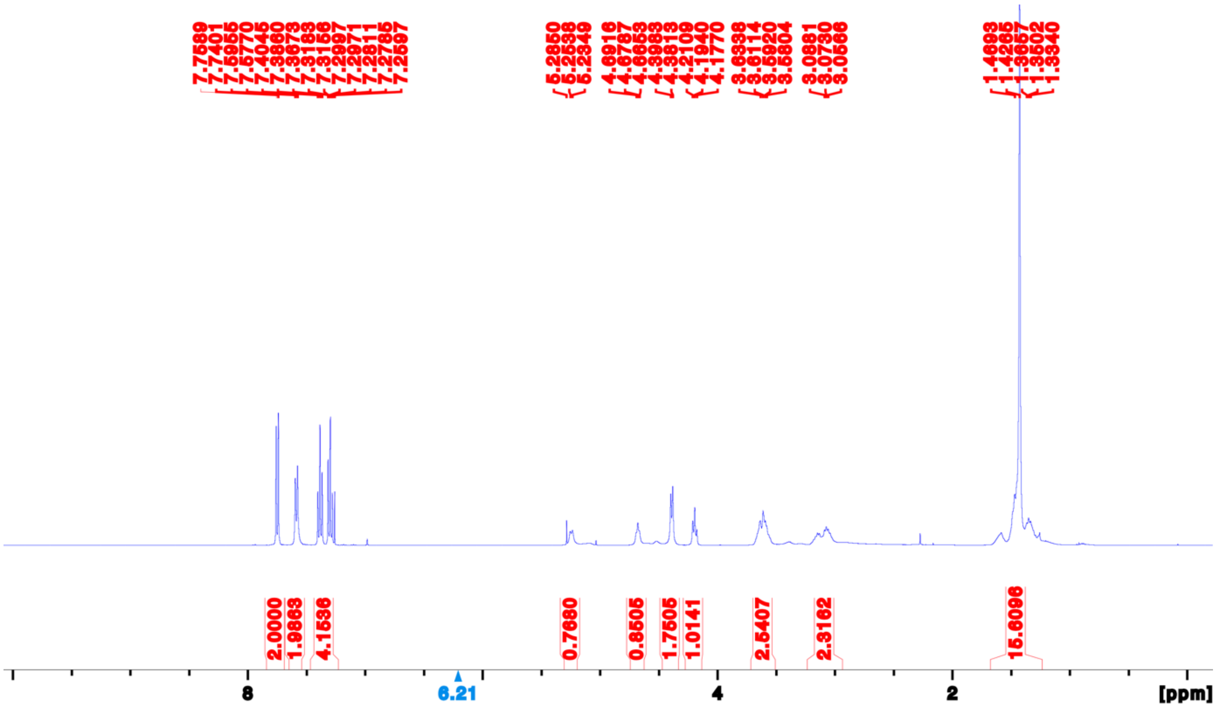

^{13}C, 101 MHz, CDCl$_3$

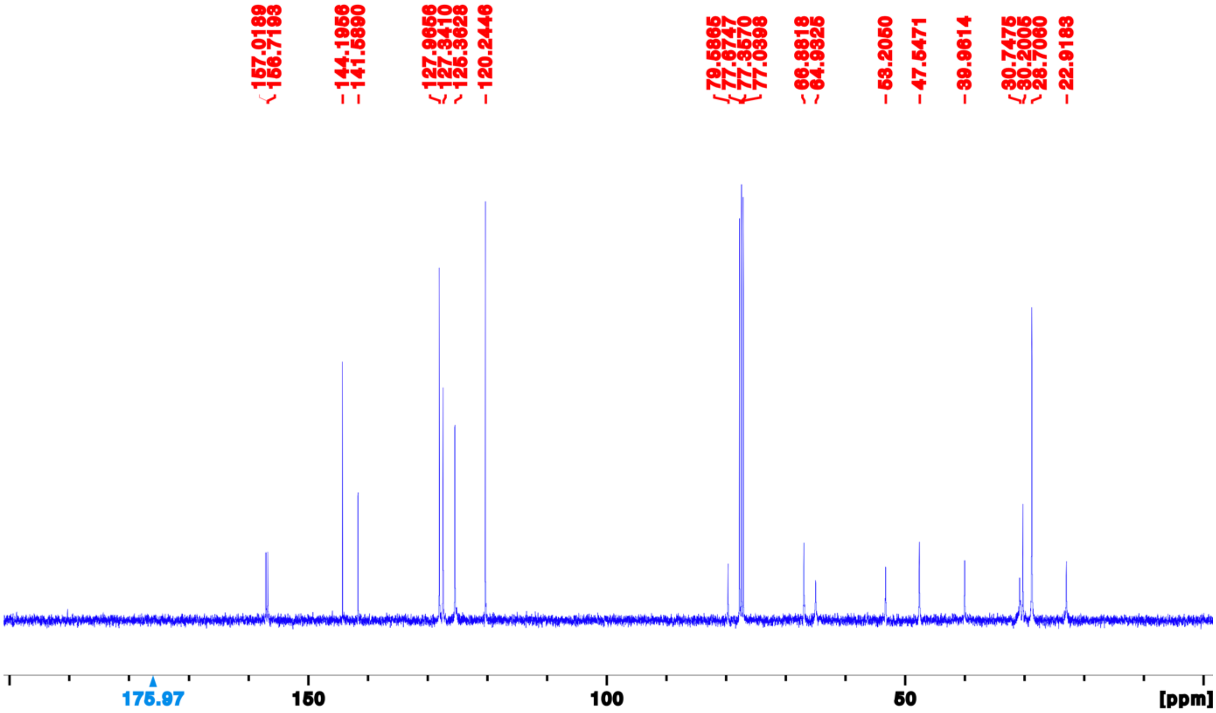

(S)-tert-Butyl (6-hydroxy-5-(4-methylphenylsulfonamido)hexyl)carbamate 2

^{1}H, 500 MHz, DMSO-d6

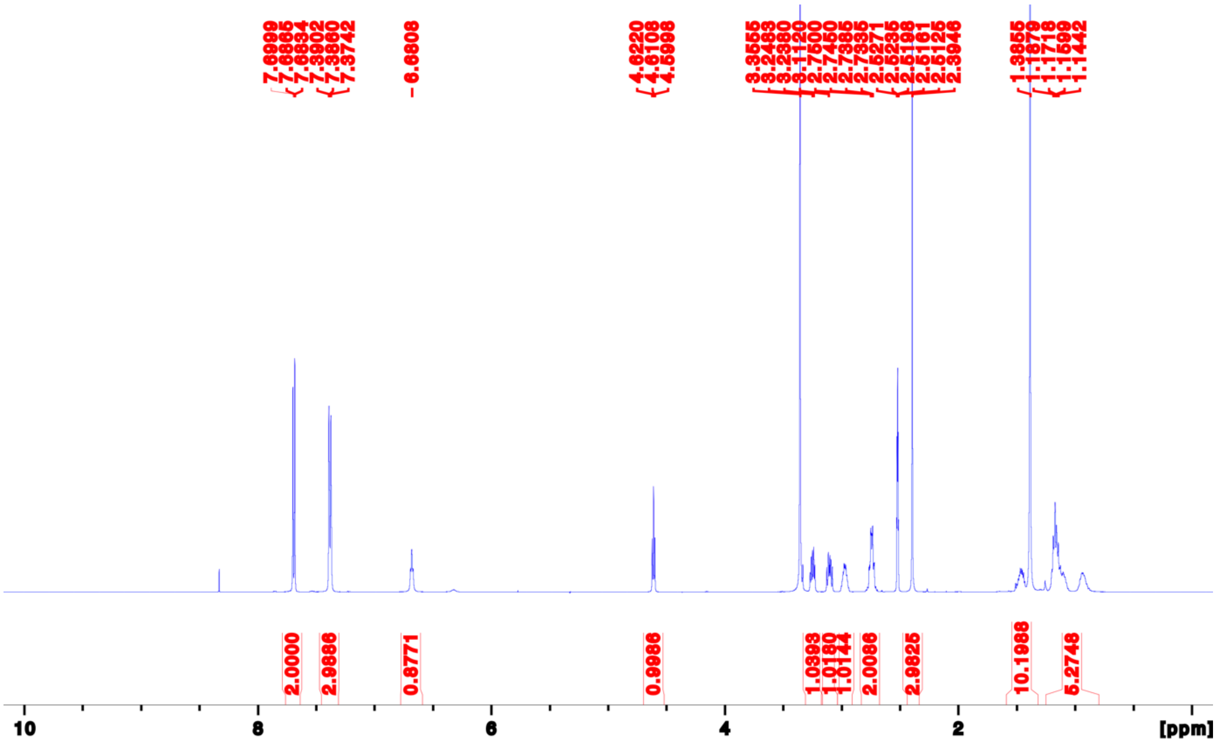

^{13}C, 126 MHz, DMSO-d6

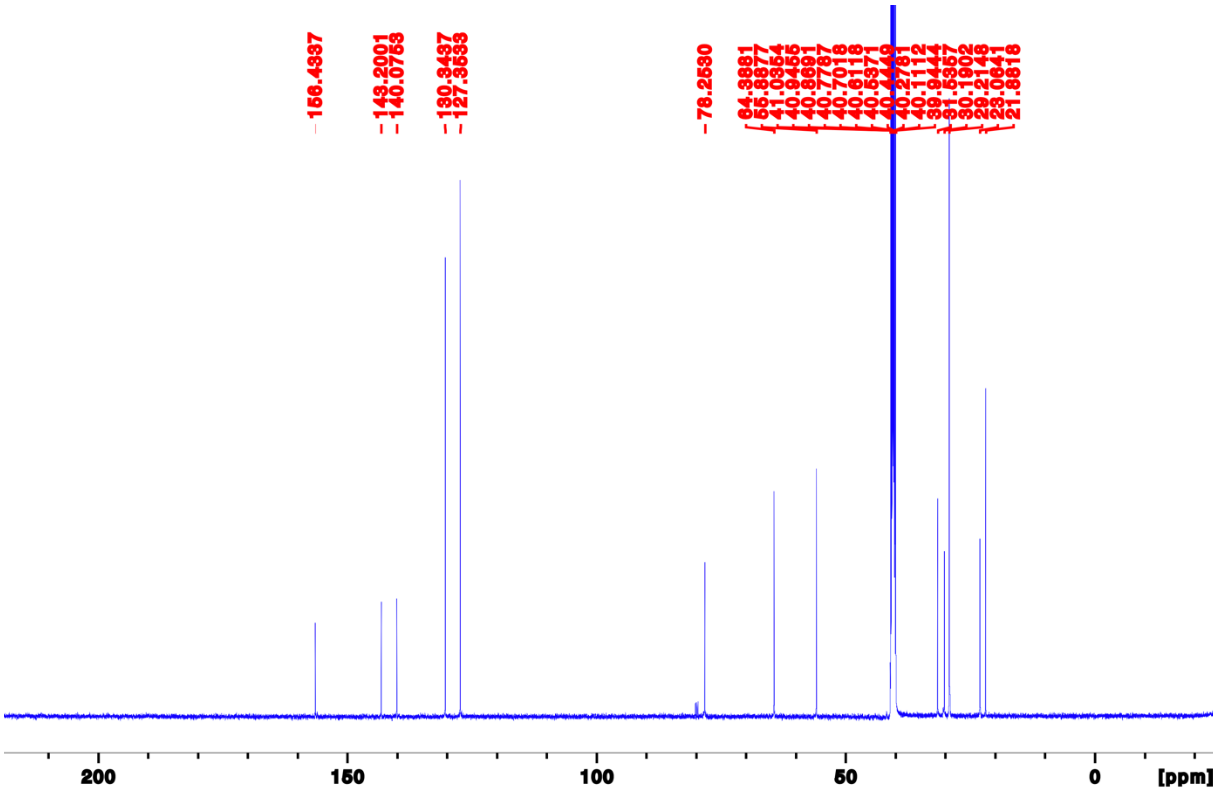

(S)-tert-Butyl (6-hydroxy-5-(4-nitrophenylsulfonamido)hexyl)carbamate 3

^{1}H, 400 MHz, $CDCl_3$

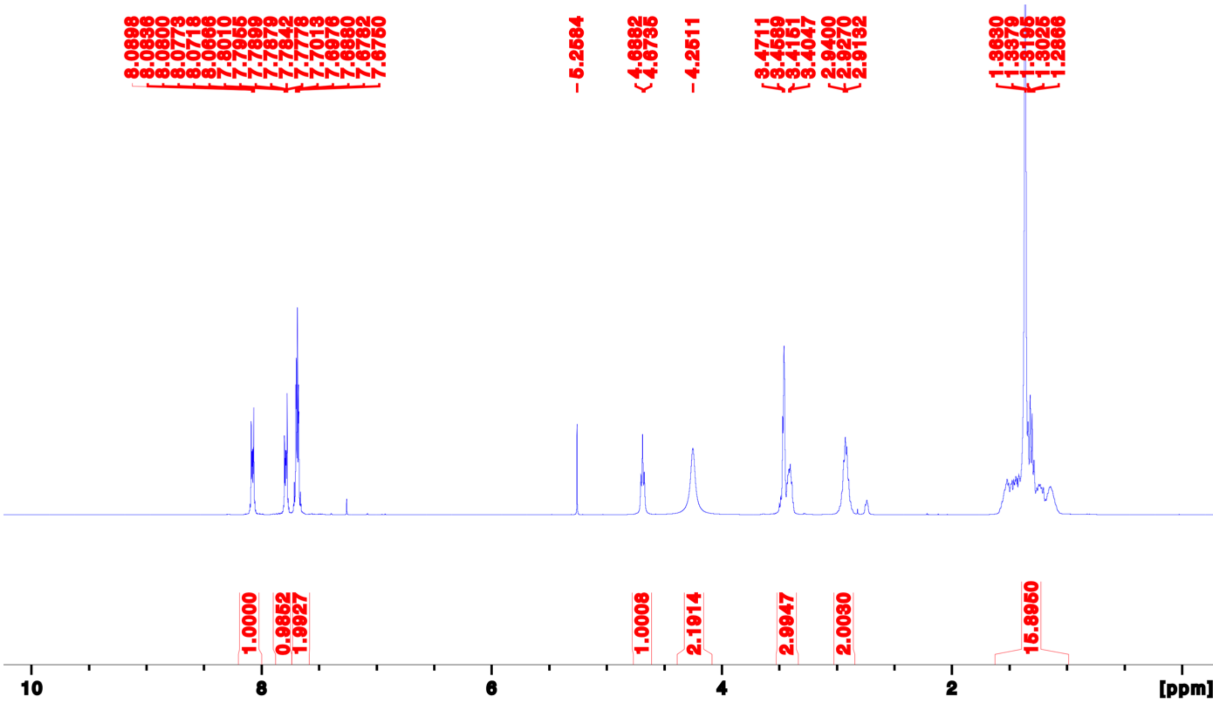

^{13}C, 101 MHz, $CDCl_3$

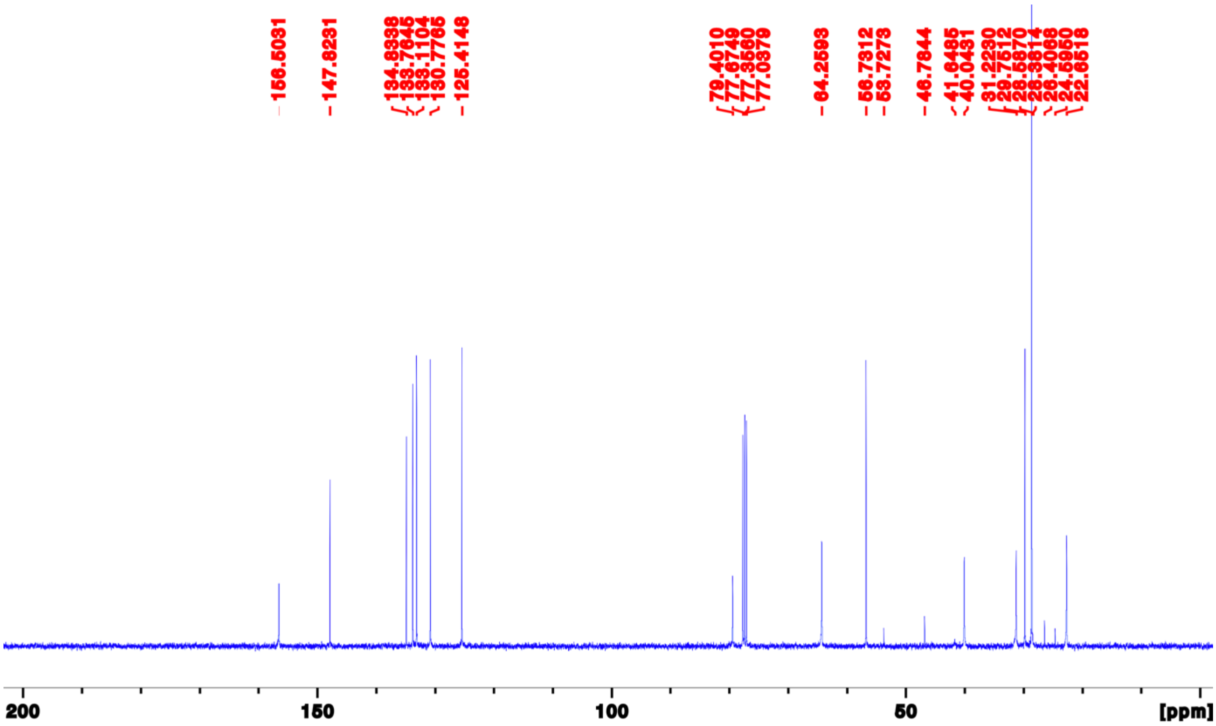

(S)-tert-Butyl (4-(1-tosylaziridin-2-yl)butyl)carbamate 4

^{1}H, 200 MHz, $CDCl_3$

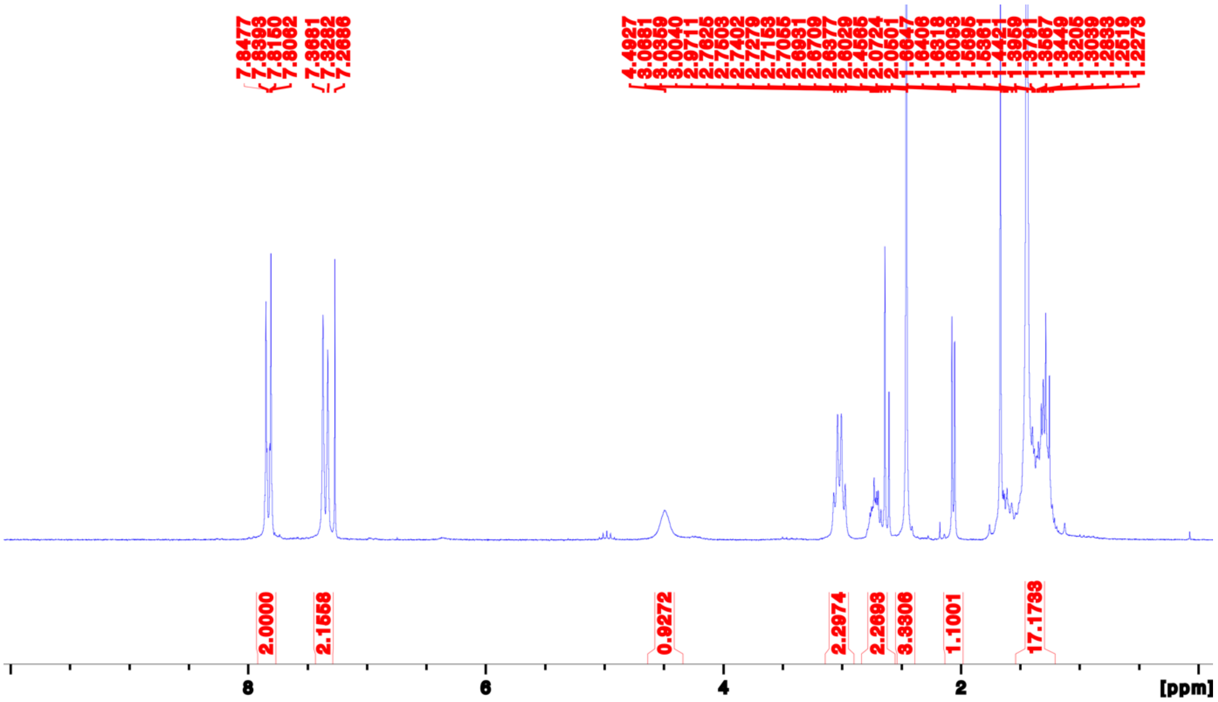

^{13}C, 101 MHz, $CDCl_3$

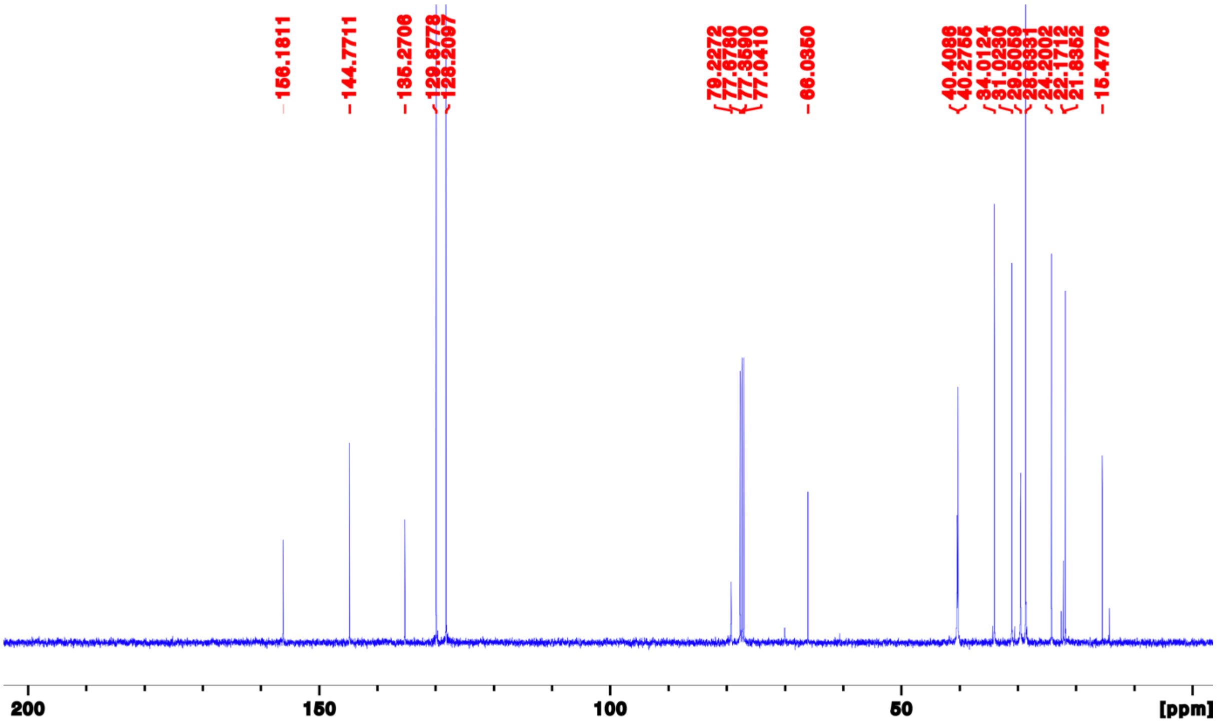

(S)-tert-Butyl (5-(4-methylphenylsulfonamido)-6-(phenylamino)hexyl)carbamate 6

^{1}H, 400 MHz, $CDCl_3$

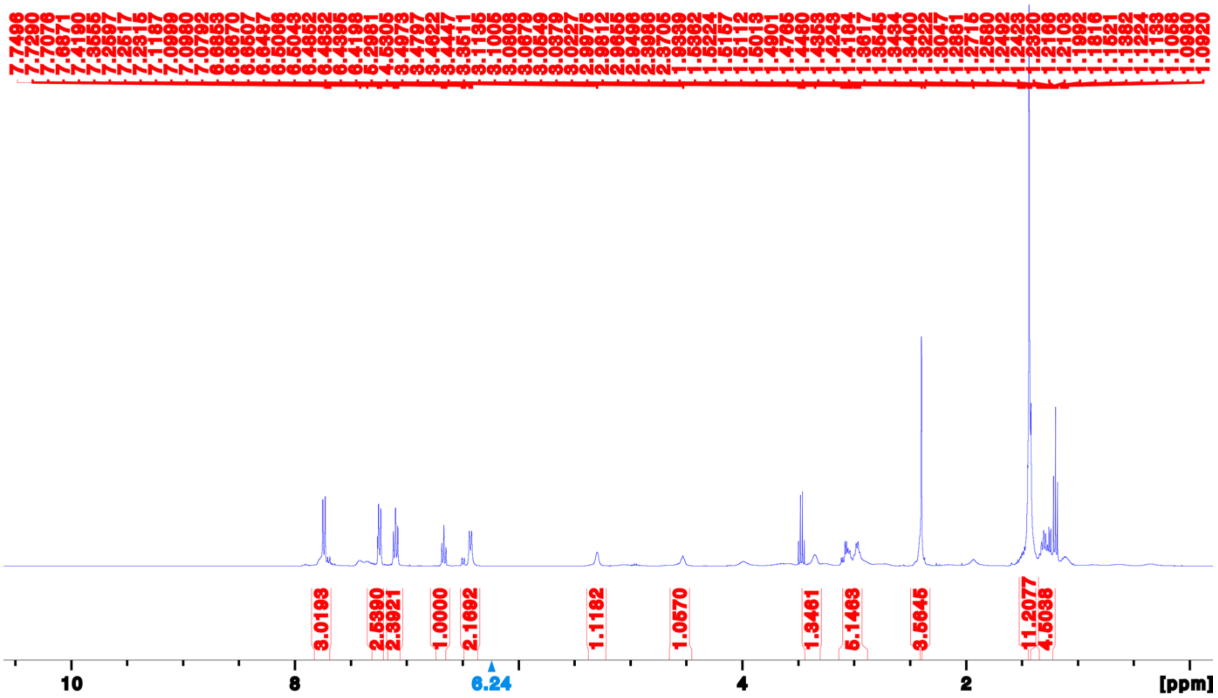

^{13}C, 101 MHz, $CDCl_3$

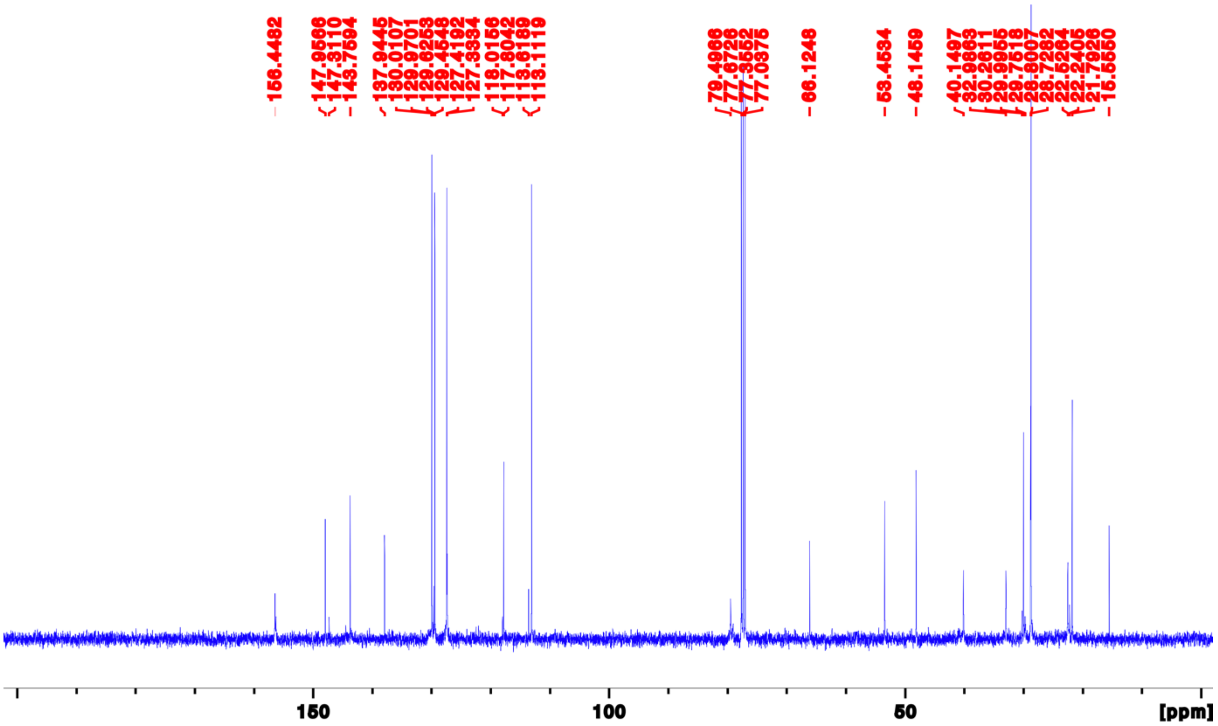

(S)-tert-Butyl (5-(4-nitrophenylsulfonamido)-6-(phenylamino)hexyl)carbamate 7

^{1}H, 400 MHz, $CDCl_3$

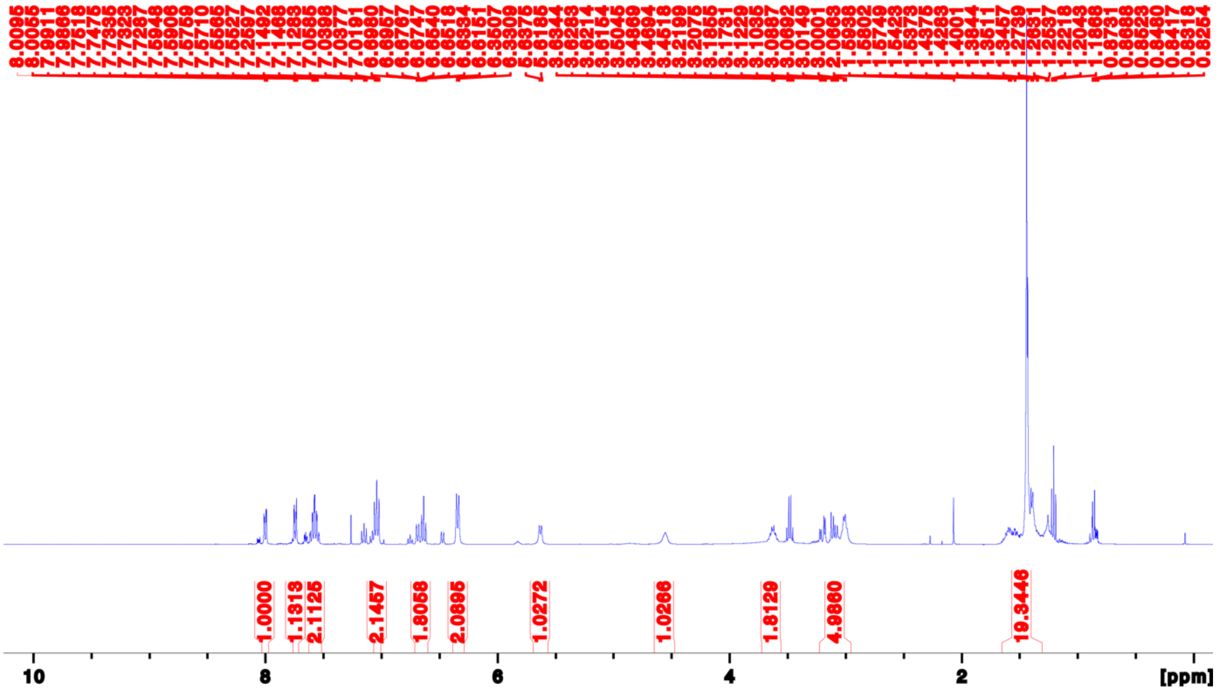

^{13}C, 101 MHz, $CDCl_3$

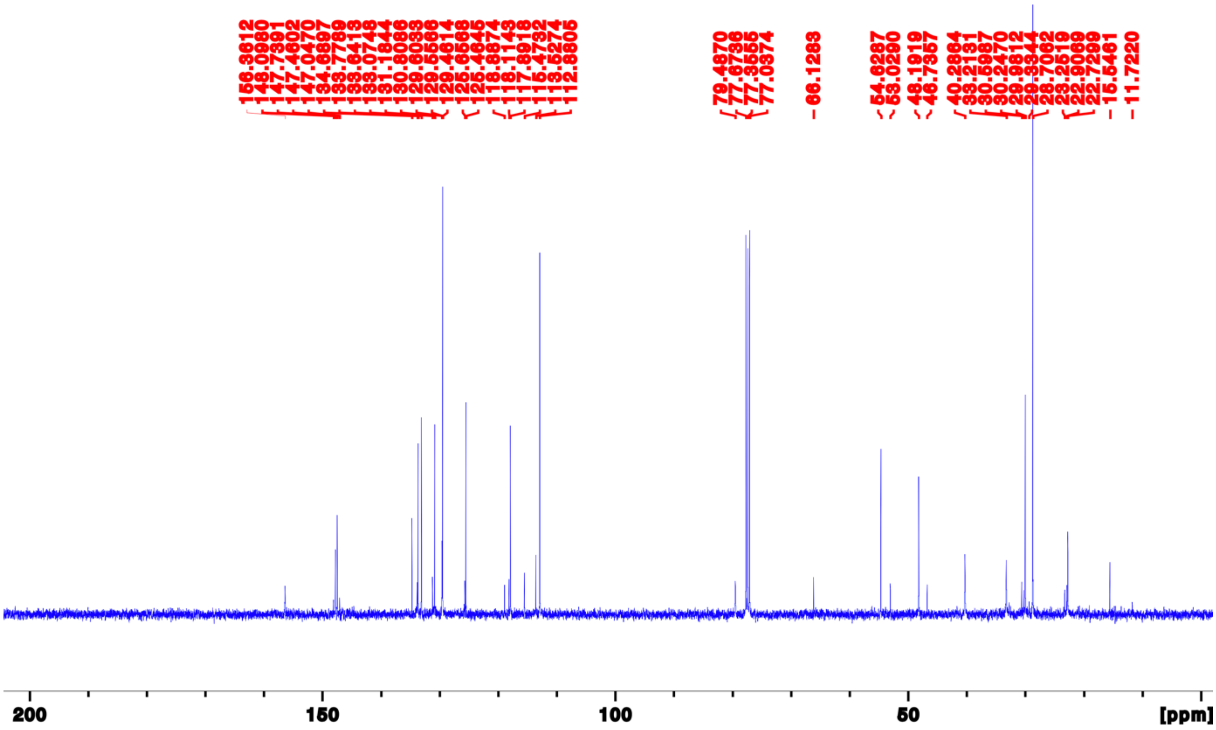

tert-Butyl (S)-(4-(2-oxo-1-phenyl-3-tosylimidazolidin-4-yl)butyl)carbamate 8

^{1}H, 400 MHz, $CDCl_3$

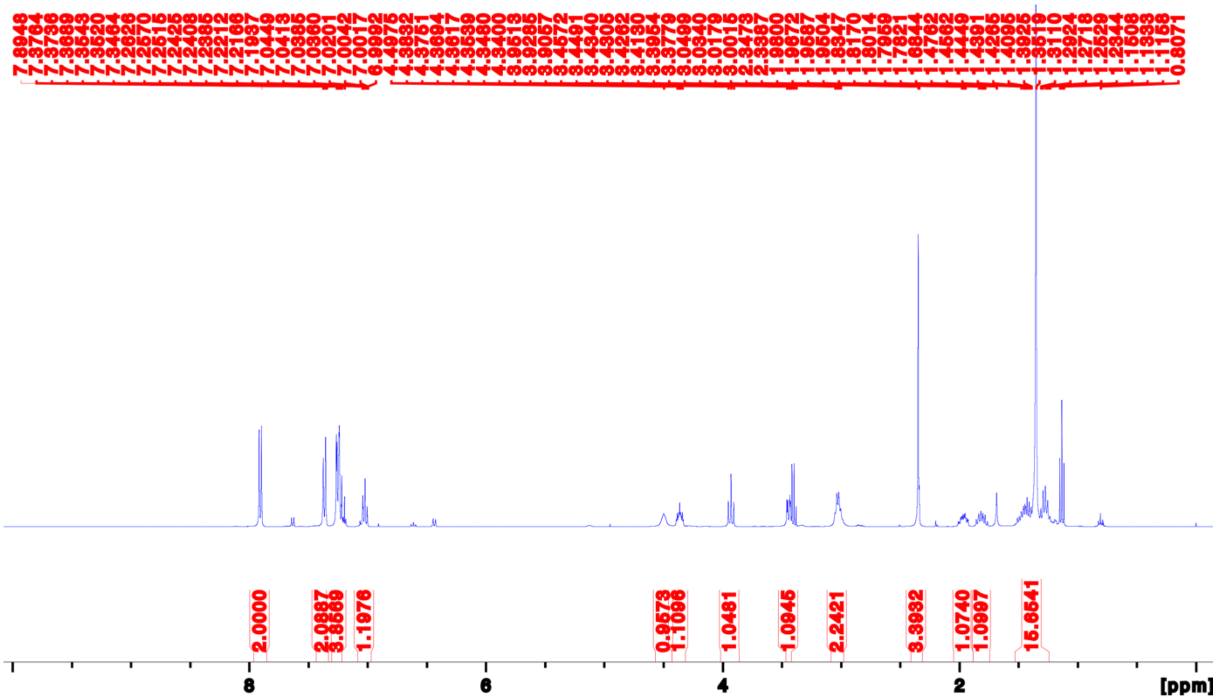

^{13}C, 101 MHz, $CDCl_3$

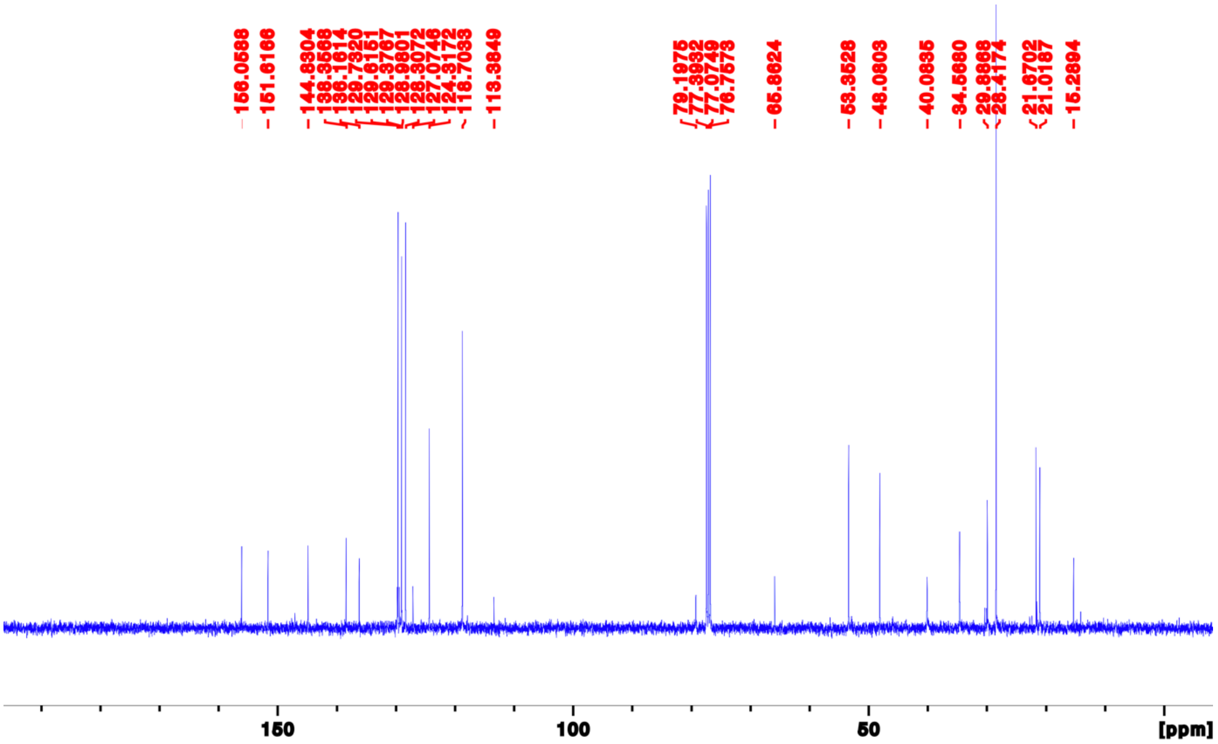

(S)-tert-Butyl (4-(3-((4-nitrophenyl)sulfonyl)-2-oxo-1-phenylimidazolidin-4-yl)butyl)carbamate 9

^{1}H, 400 MHz, CDCl$_3$

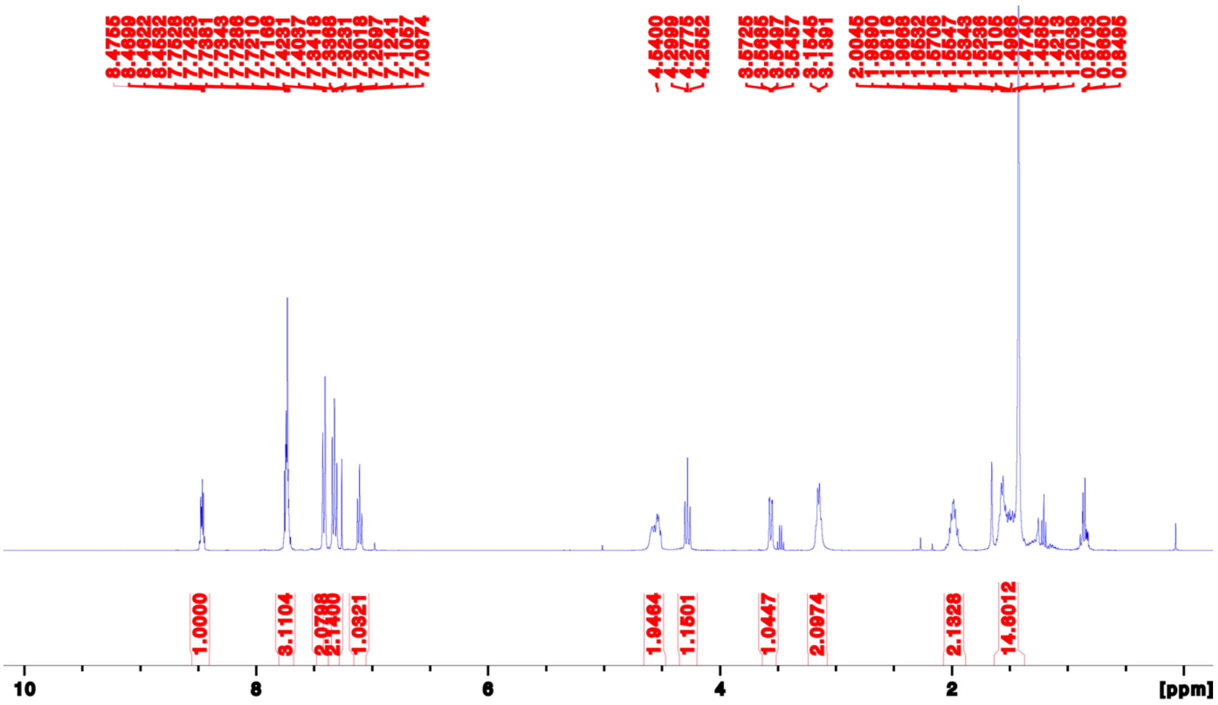

^{13}C, 101 MHz, CDCl$_3$

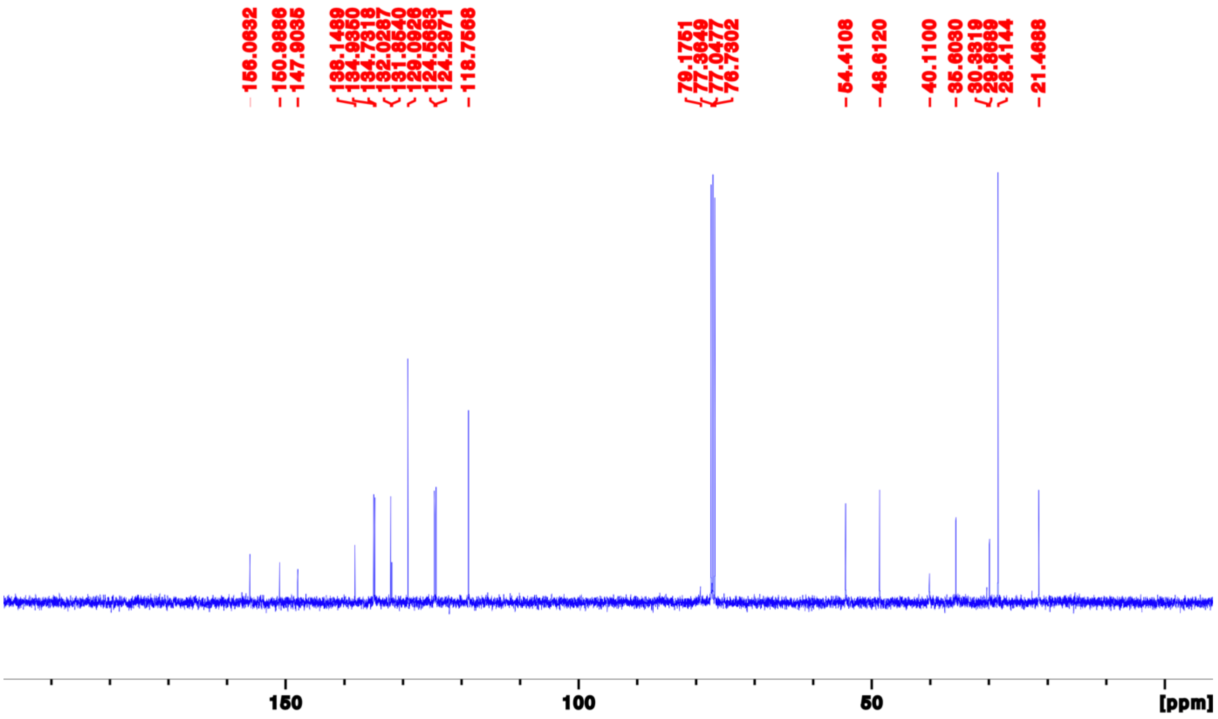

(S)-tert-Butyl (4-(2-oxo-3-tosylimidazolidin-4-yl)butyl)carbamate 12

^{1}H, 400 MHz, $CDCl_3$

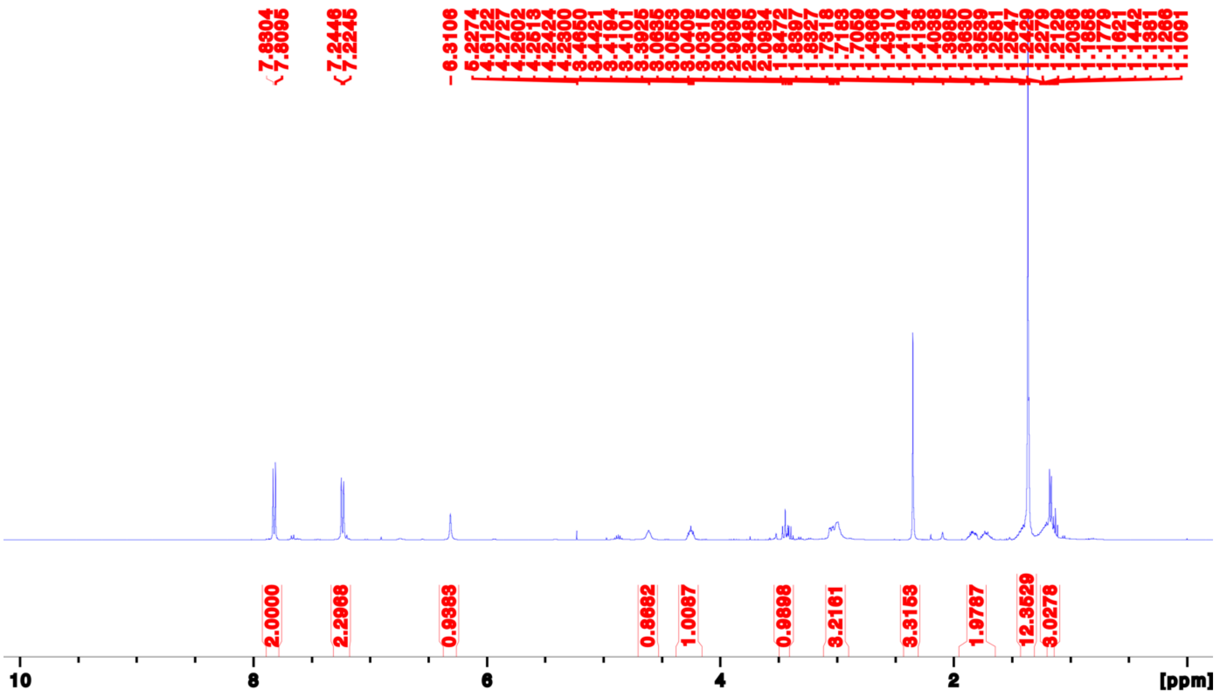

^{13}C, 101 MHz, $CDCl_3$

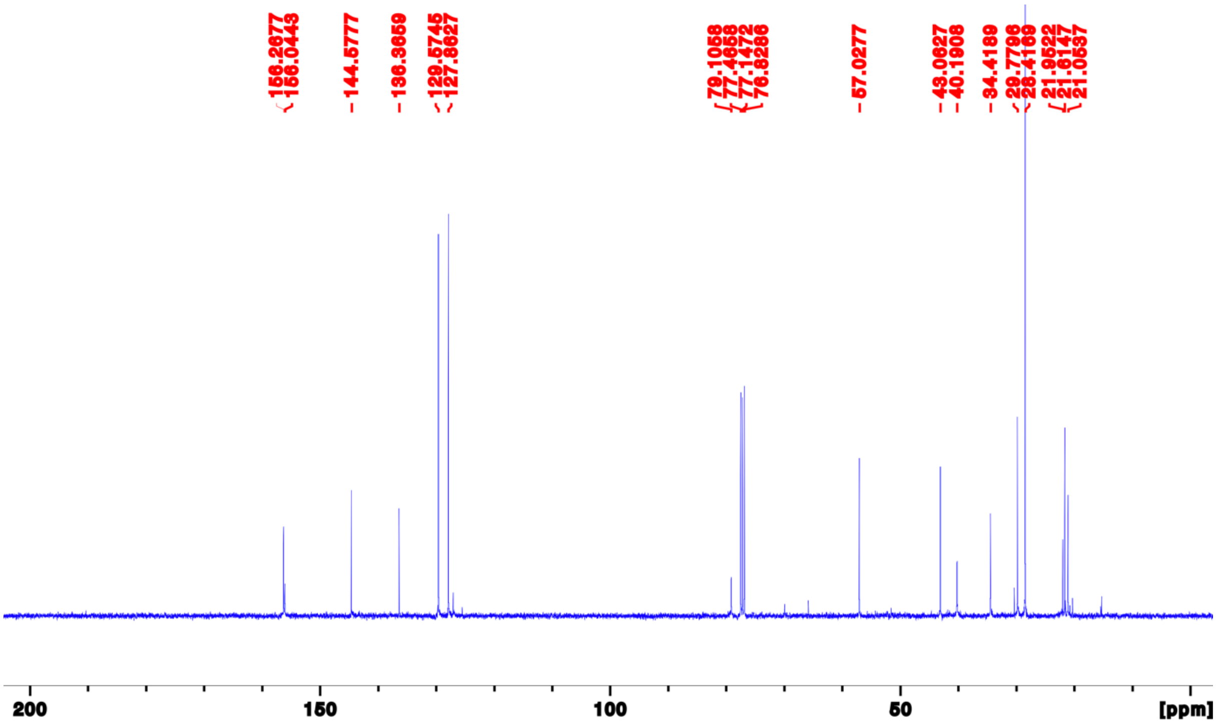

(S)-tert-Butyl (4-(3-((4-nitrophenyl)sulfonyl)-2-oxoimidazolidin-4-yl)butyl)carbamate 13

^{1}H, 400 MHz, $CDCl_3$

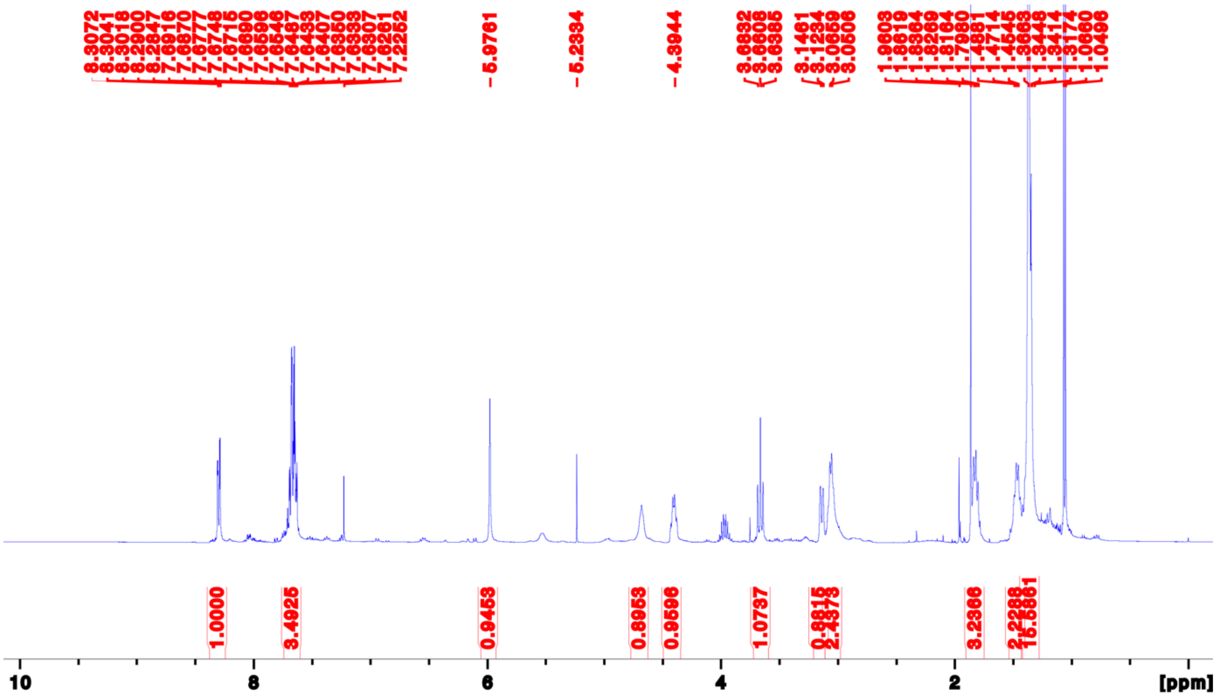

^{13}C, 101 MHz, $CDCl_3$

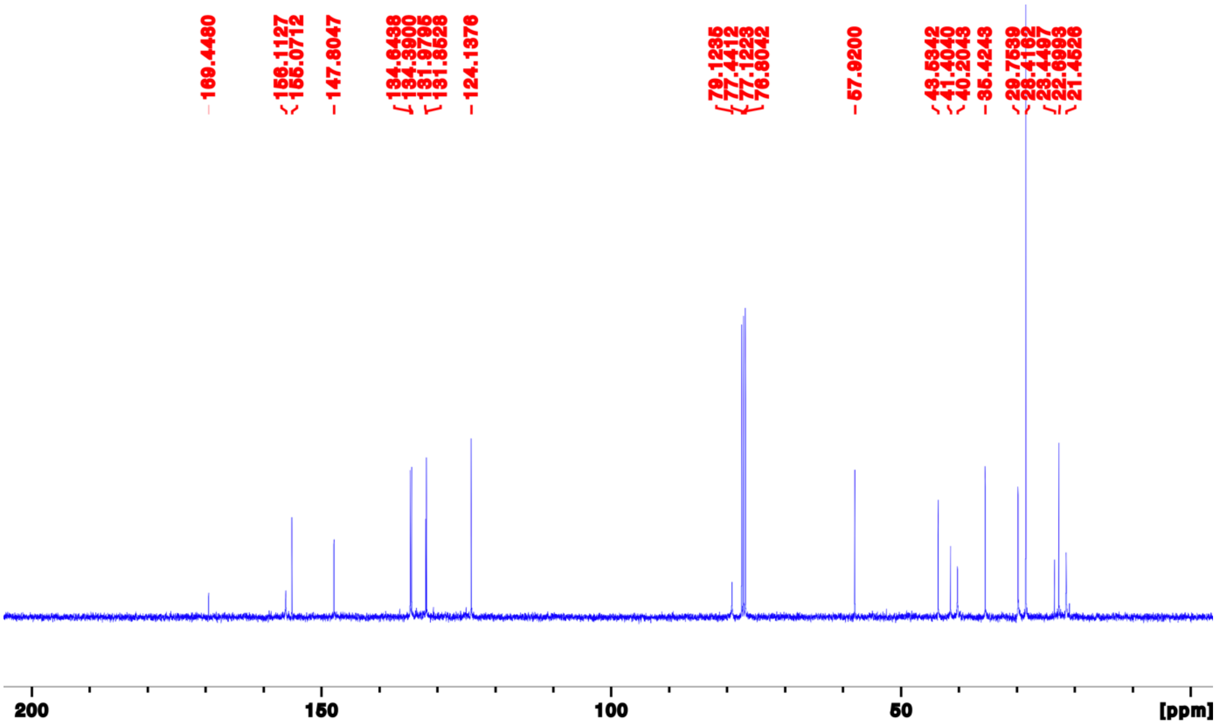

(S)-tert-Butyl (4-(1-(6-bromopyridin-2-yl)-2-oxo-3-tosylimidazolidin-4-yl)butyl)carbamate 14

^{1}H, 400 MHz, $CDCl_3$

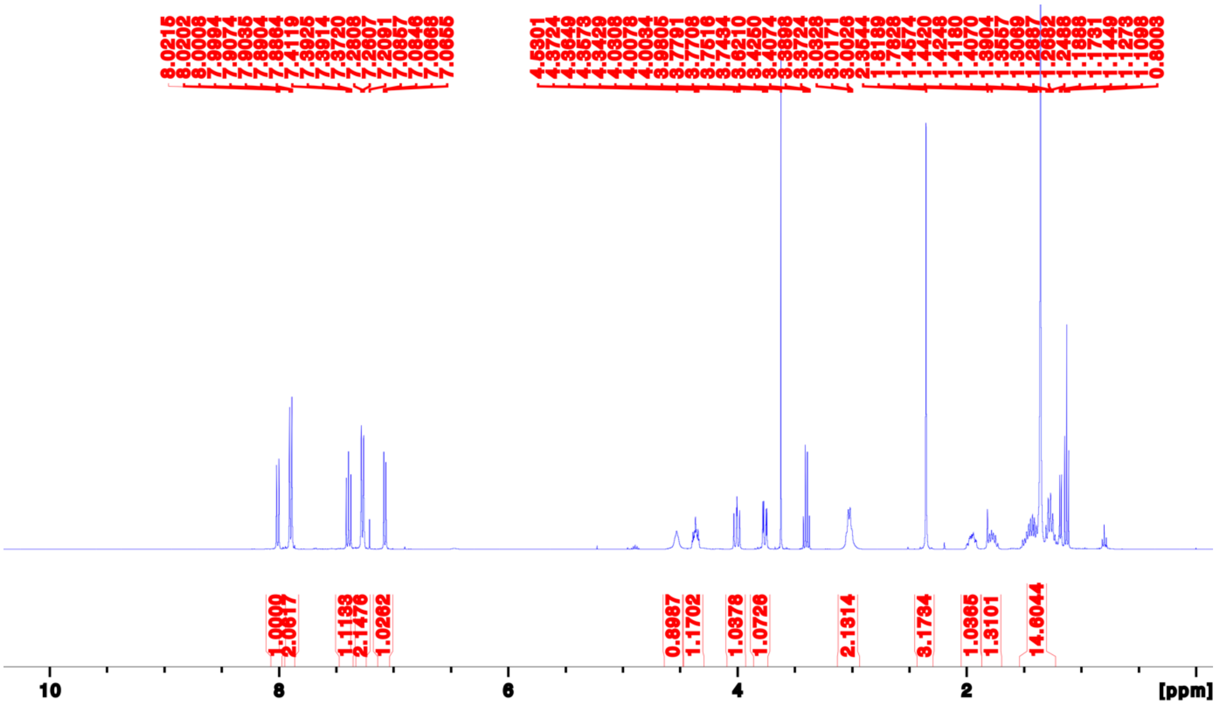

^{13}C, 101 MHz, $CDCl_3$

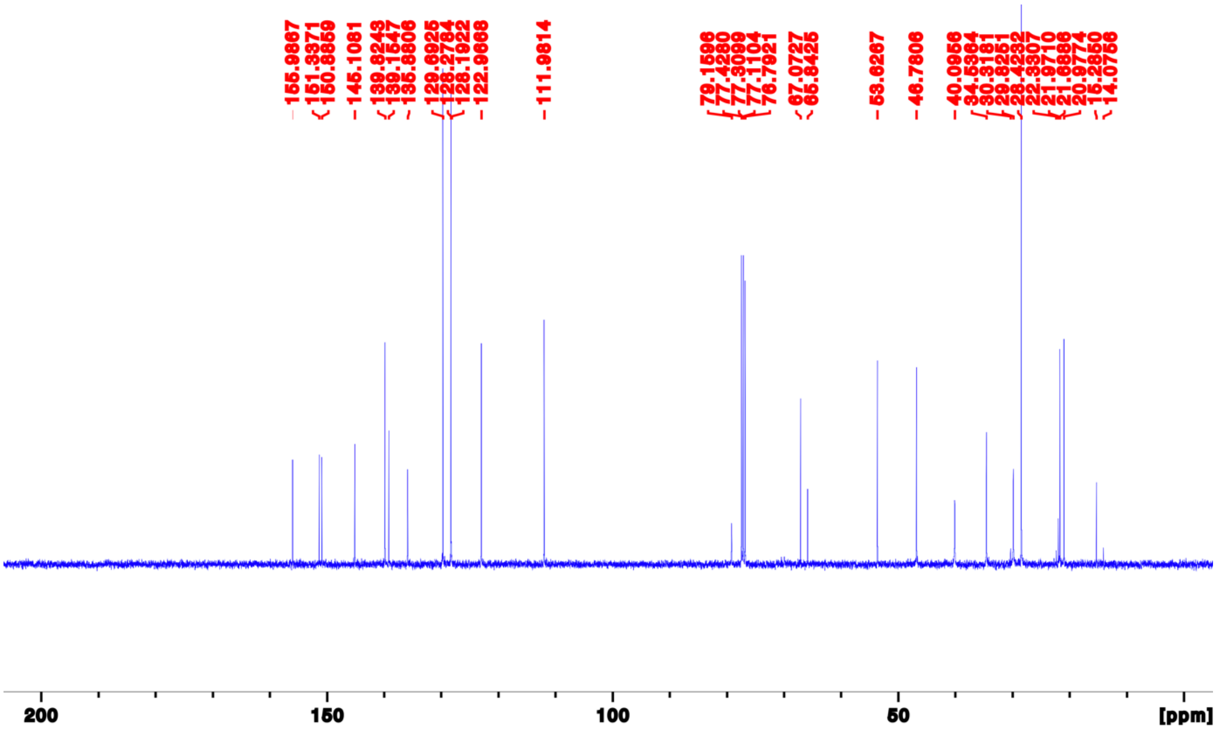

(S)-tert-Butyl (4-(1-(6-bromopyridin-2-yl)-3-((2-nitrophenyl)sulfonyl)-2-oxoimidazolidin-4-yl)butyl)carbamate 15

^{1}H, 400 MHz, $CDCl_3$

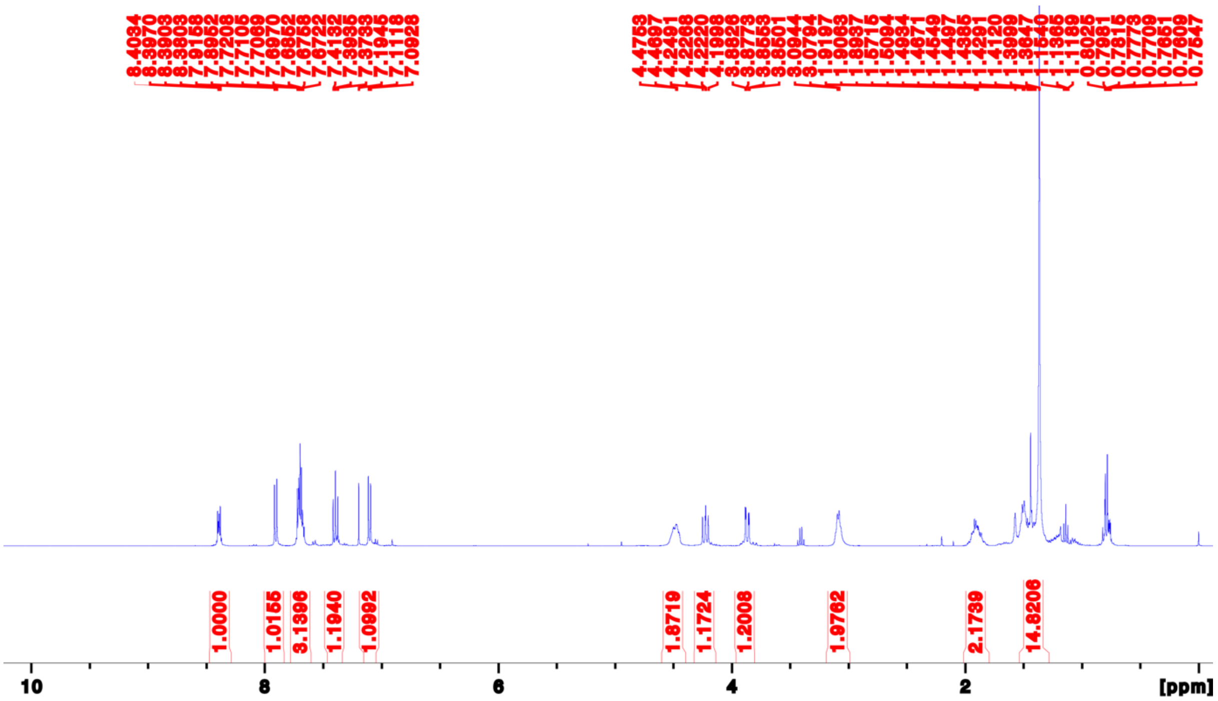

^{13}C, 101 MHz, $CDCl_3$

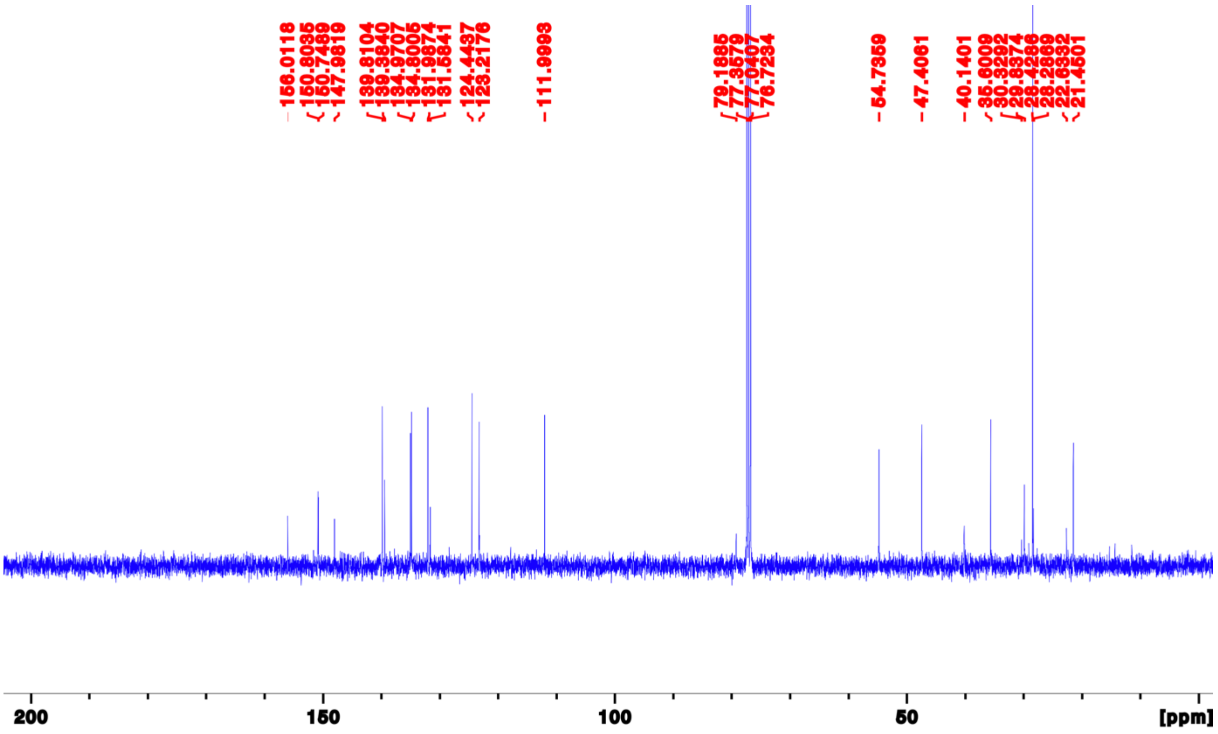

(S)-tert-Butyl (4-(2-oxo-1-phenylimidazolidin-4-yl)butyl)carbamate 16

^{1}H, 400 MHz, $CDCl_3$

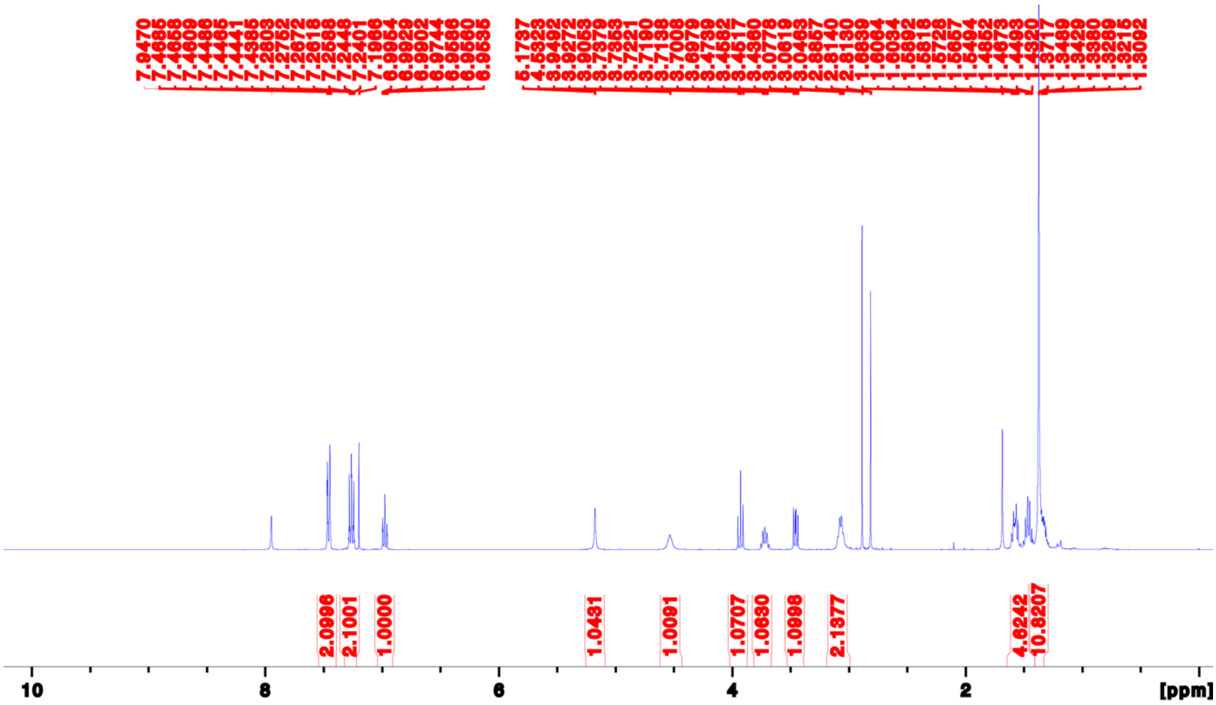

^{13}C, 101 MHz, $CDCl_3$

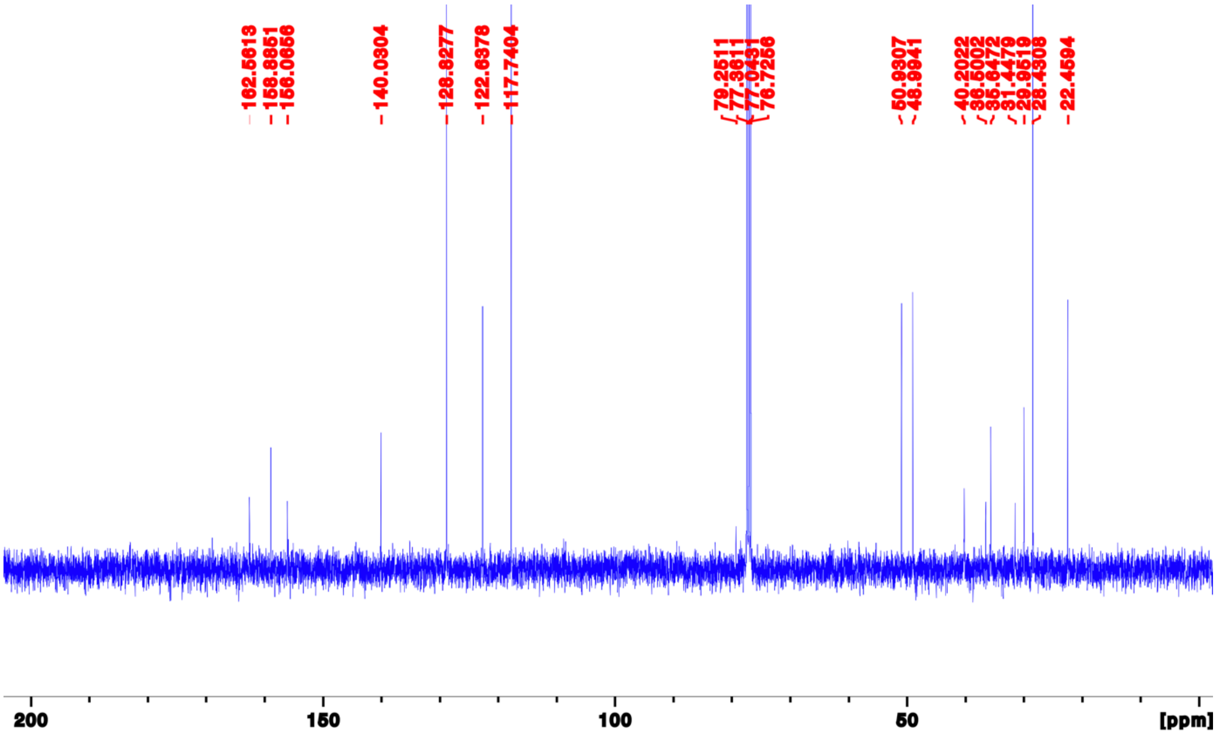

Tosyl-protected Lys-Lys dimer 17

^{1}H, 500 MHz, $CDCl_3$

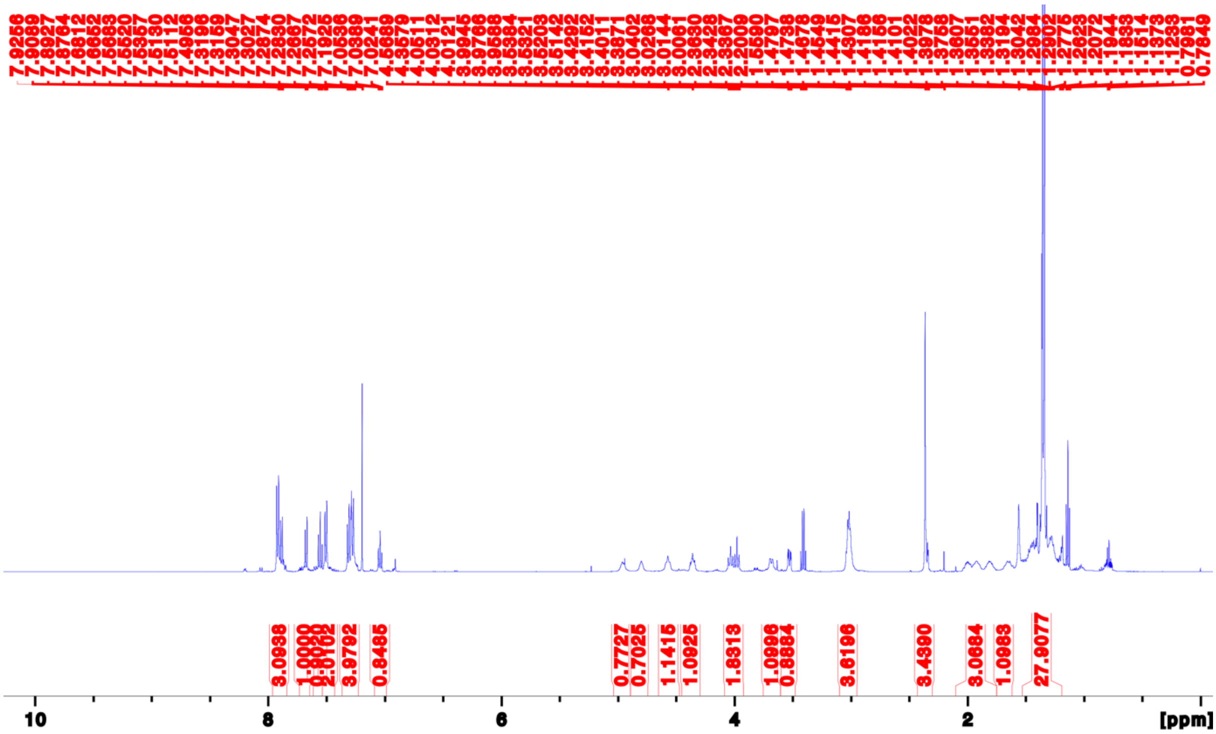

^{13}C, 126 MHz, $CDCl_3$

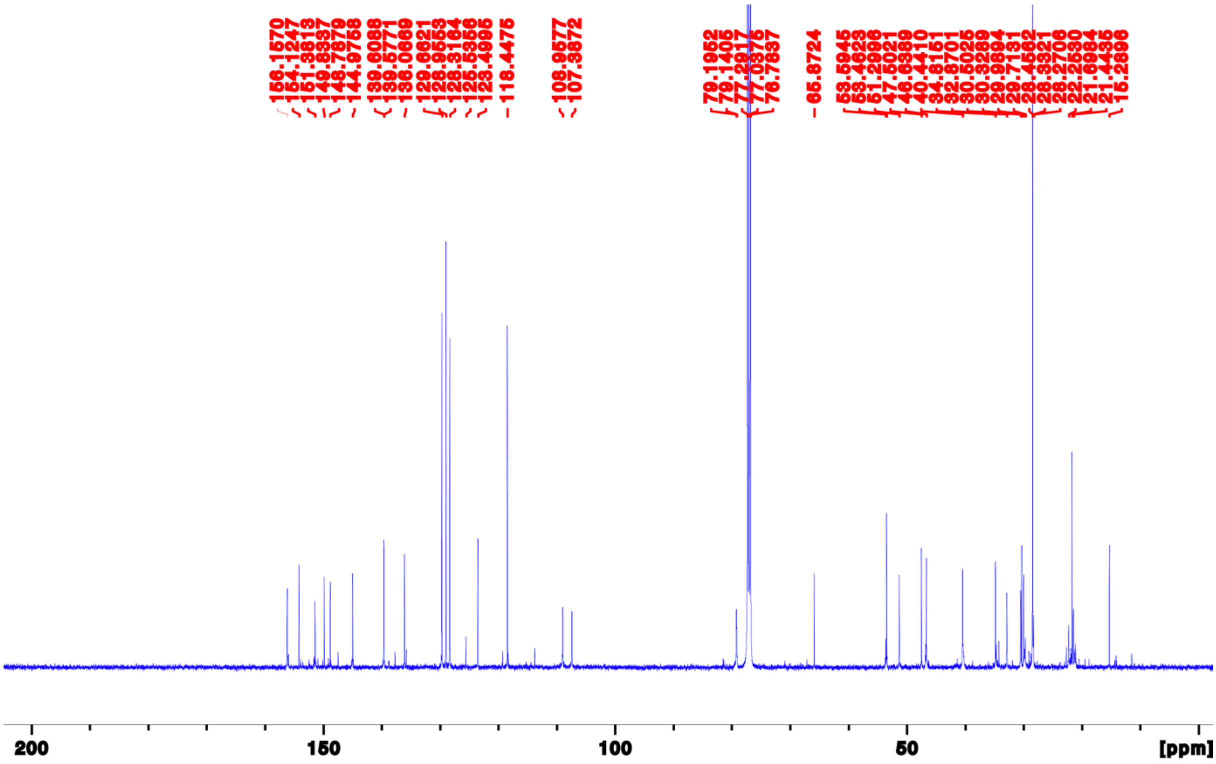

N-Deprotected Lys-Lys dimer 46

^{1}H, 500 MHz, $CDCl_3$

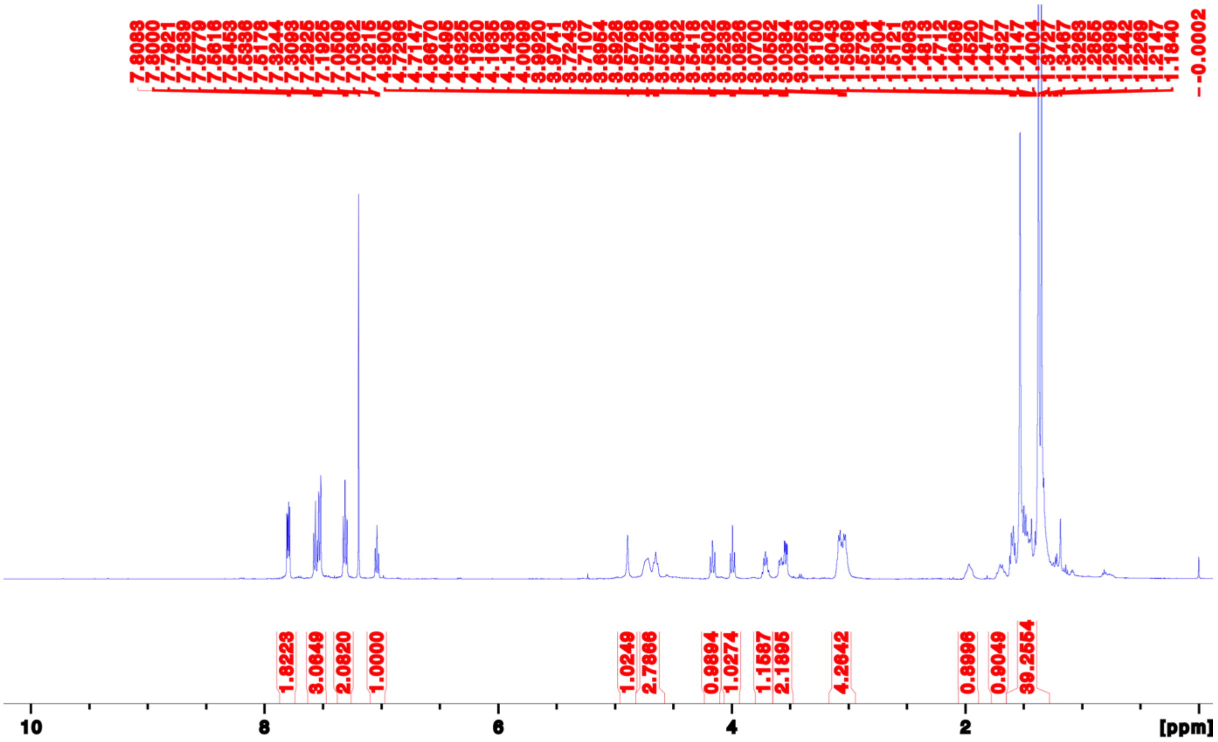

^{13}C, 151 MHz, $CDCl_3$

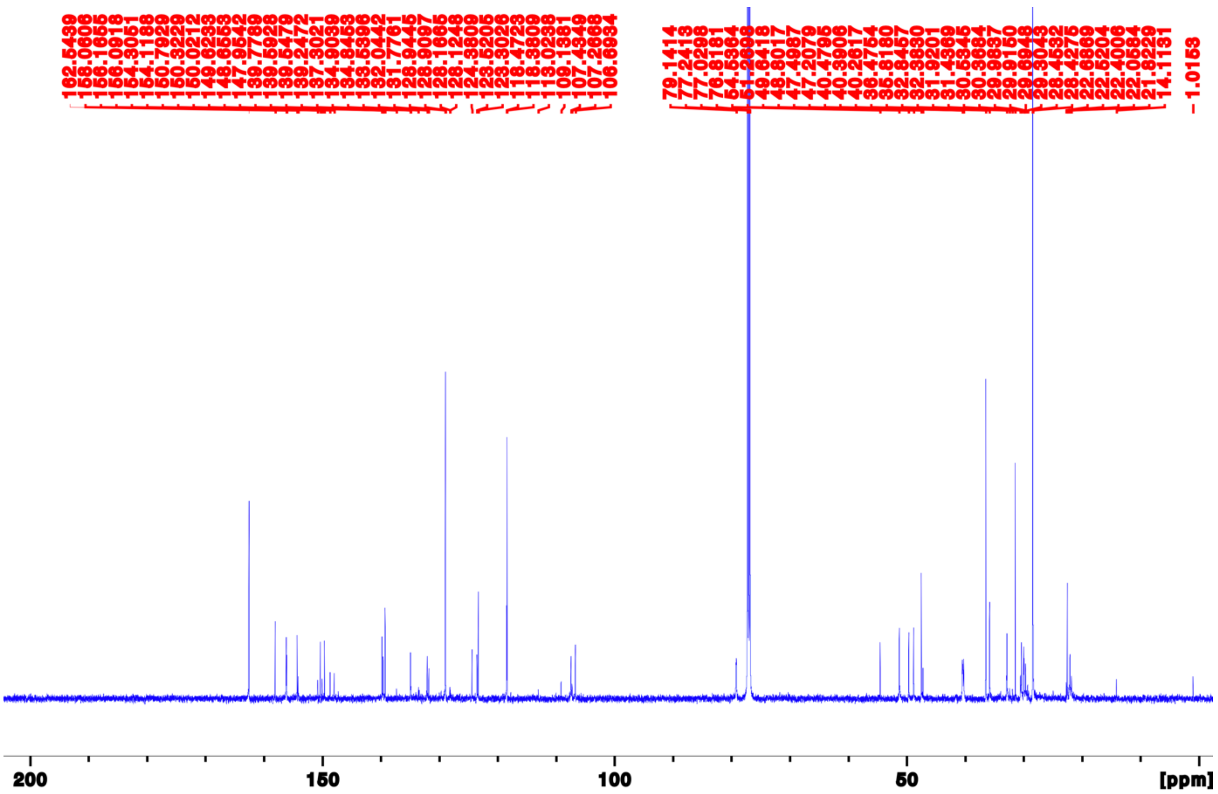

Nosyl-protected Lys-Lys dimer 18

^{1}H, 500 MHz, CDCl$_3$

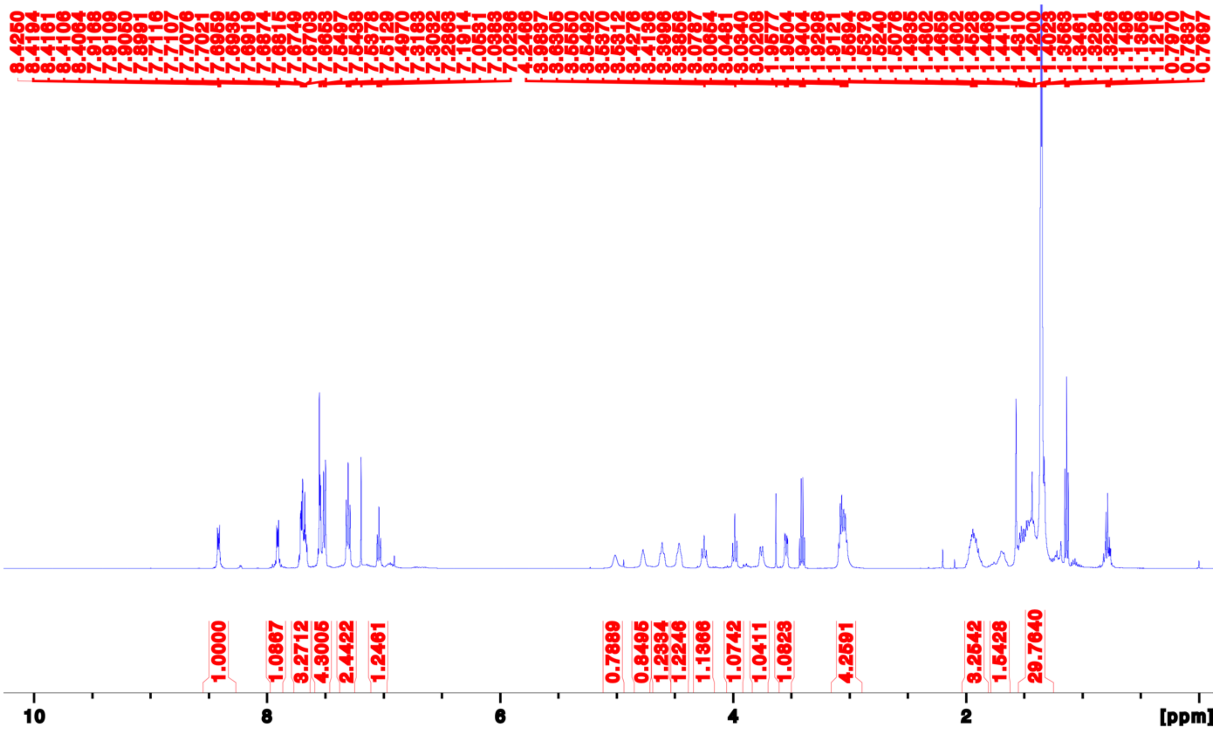

^{13}C, 126 MHz, CDCl$_3$

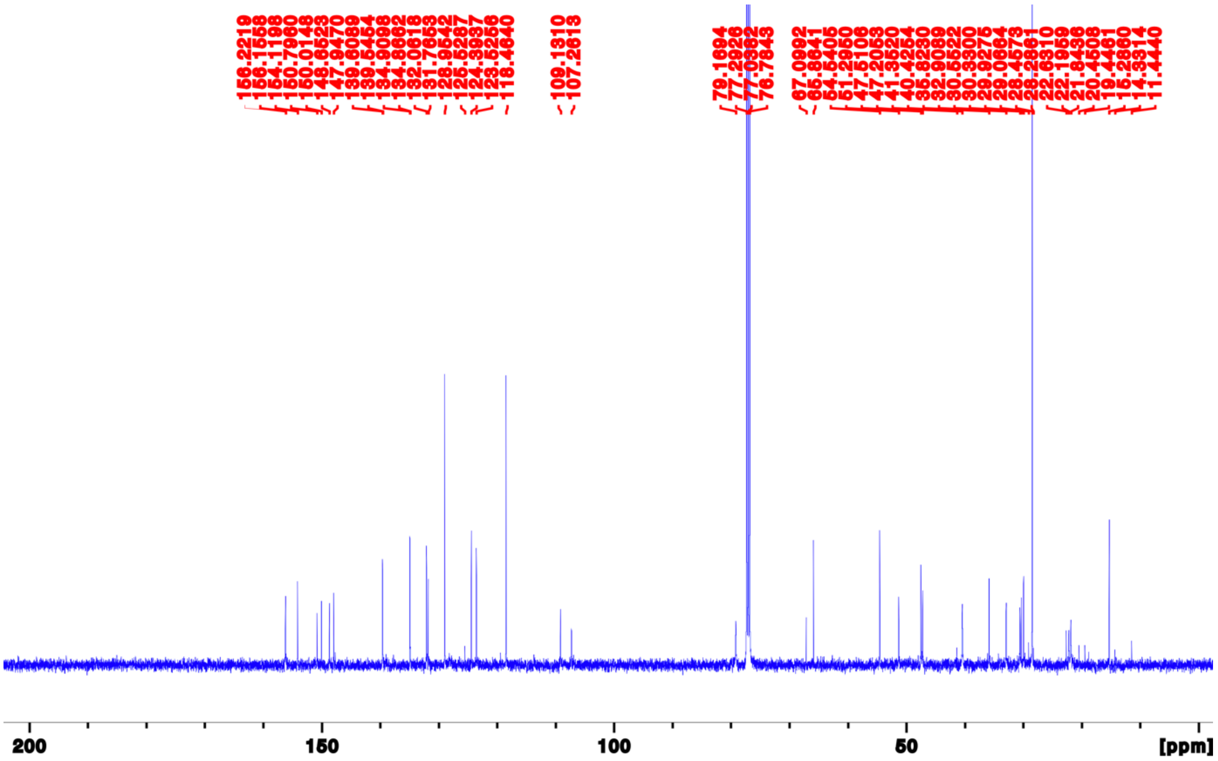

Boc-Protected Lys-Lys-Lys trimer 47

^{1}H, 400 MHz, $CDCl_3$

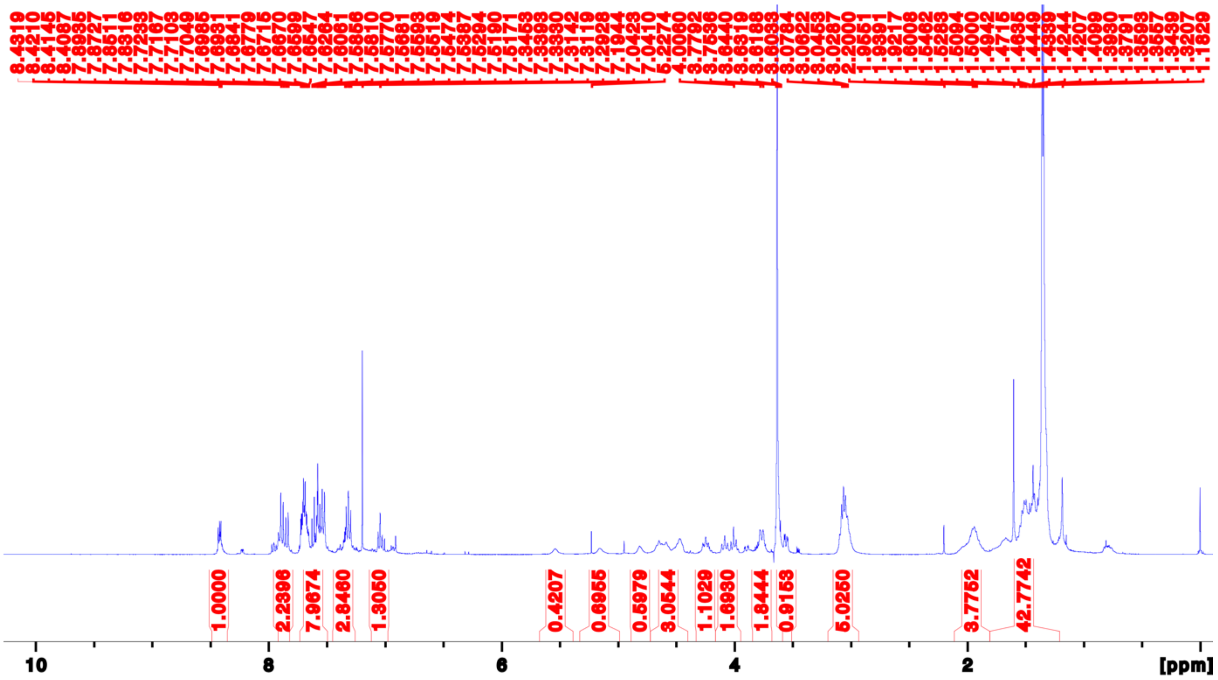

^{13}C, 150 MHz, $CDCl_3$

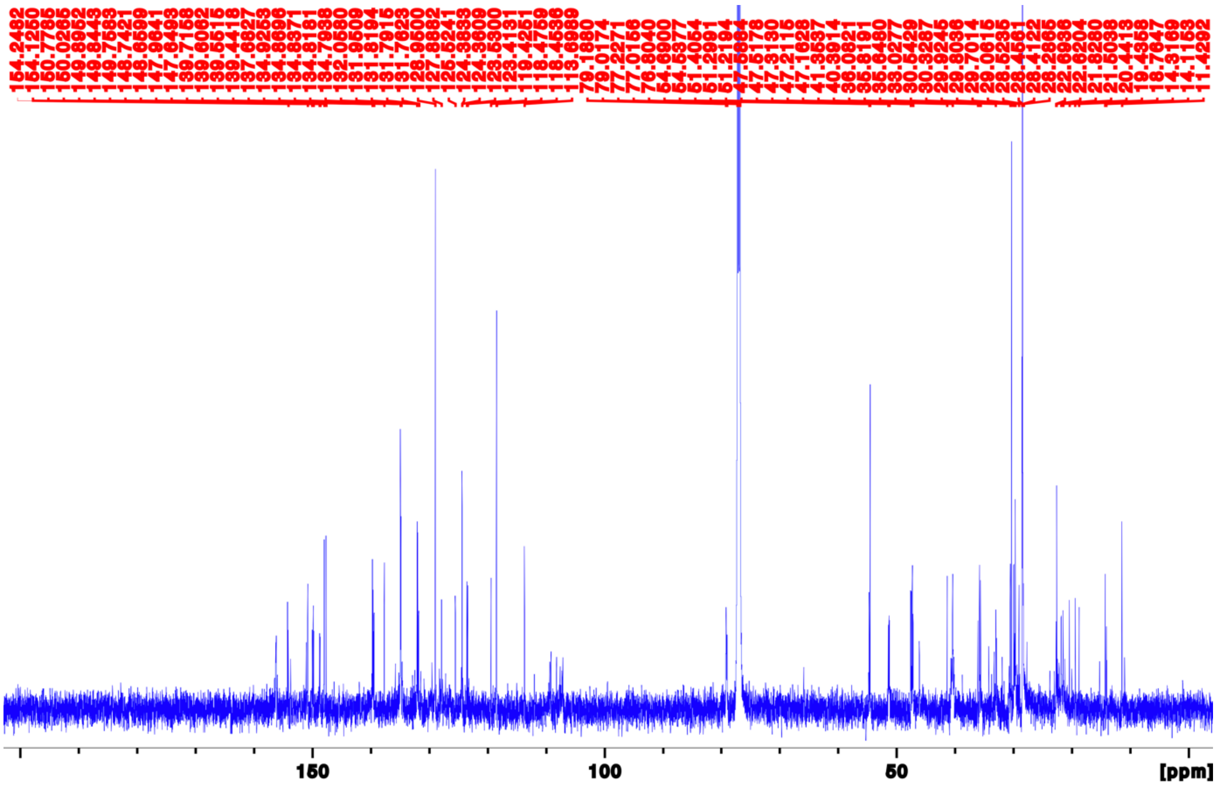

Deprotected Lys-Lys-Lys trimer 19

^{1}H, 500 MHz, CD_3OD

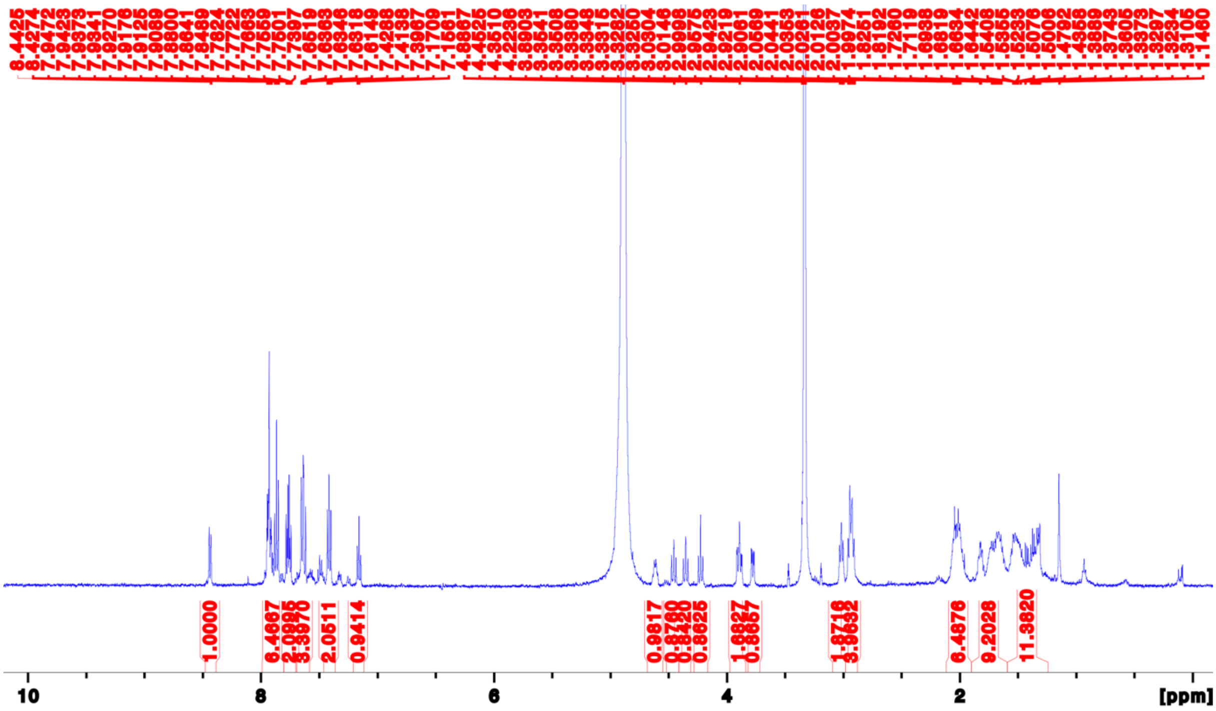

^{13}C, 126 MHz, CD_3OD

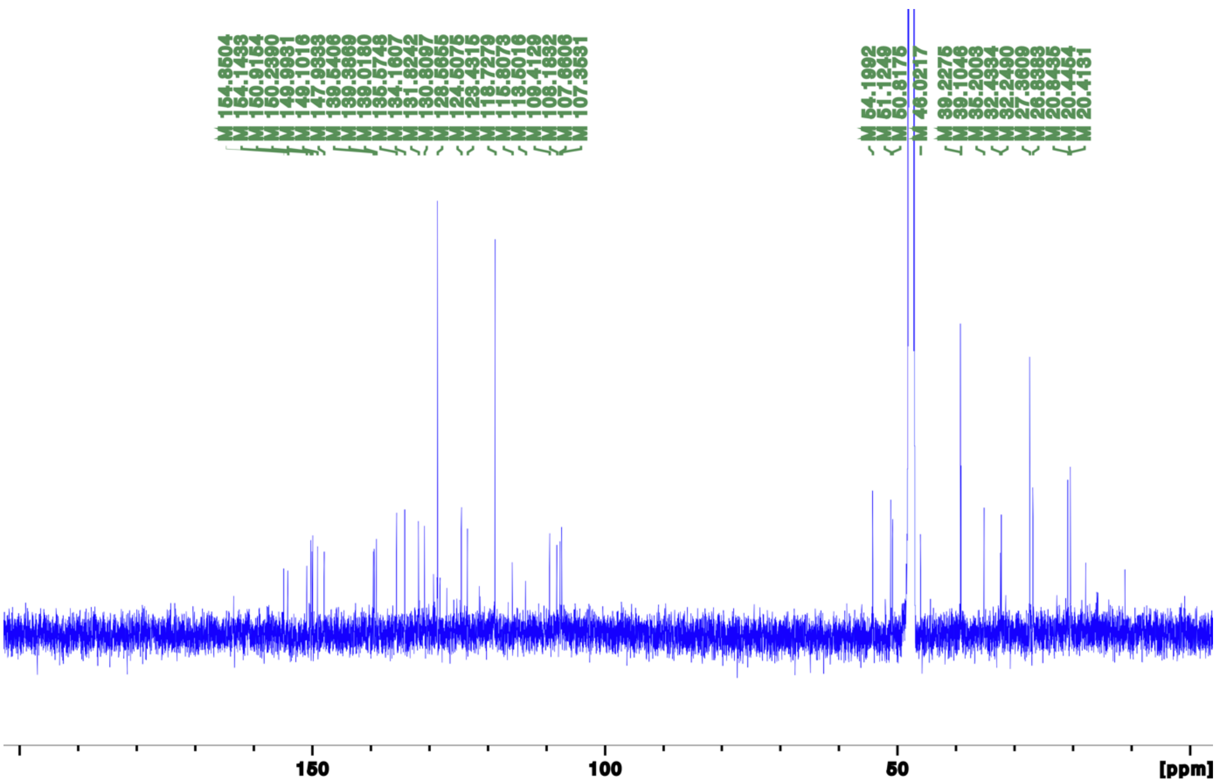

tert-Butyl (S)-4-(((benzyloxy)carbonyl)amino)-5-hydroxypentanoate 48

^{1}H, 400 MHz, $CDCl_3$

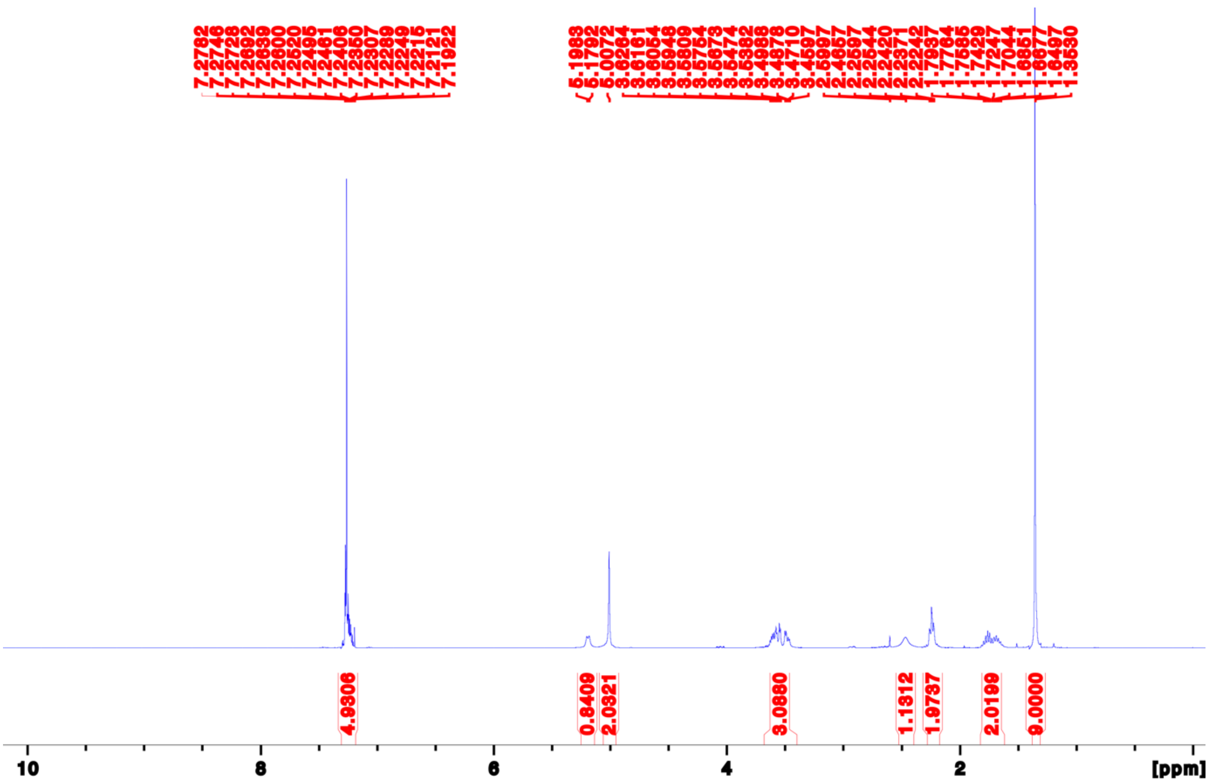

^{13}C, 100 MHz, $CDCl_3$

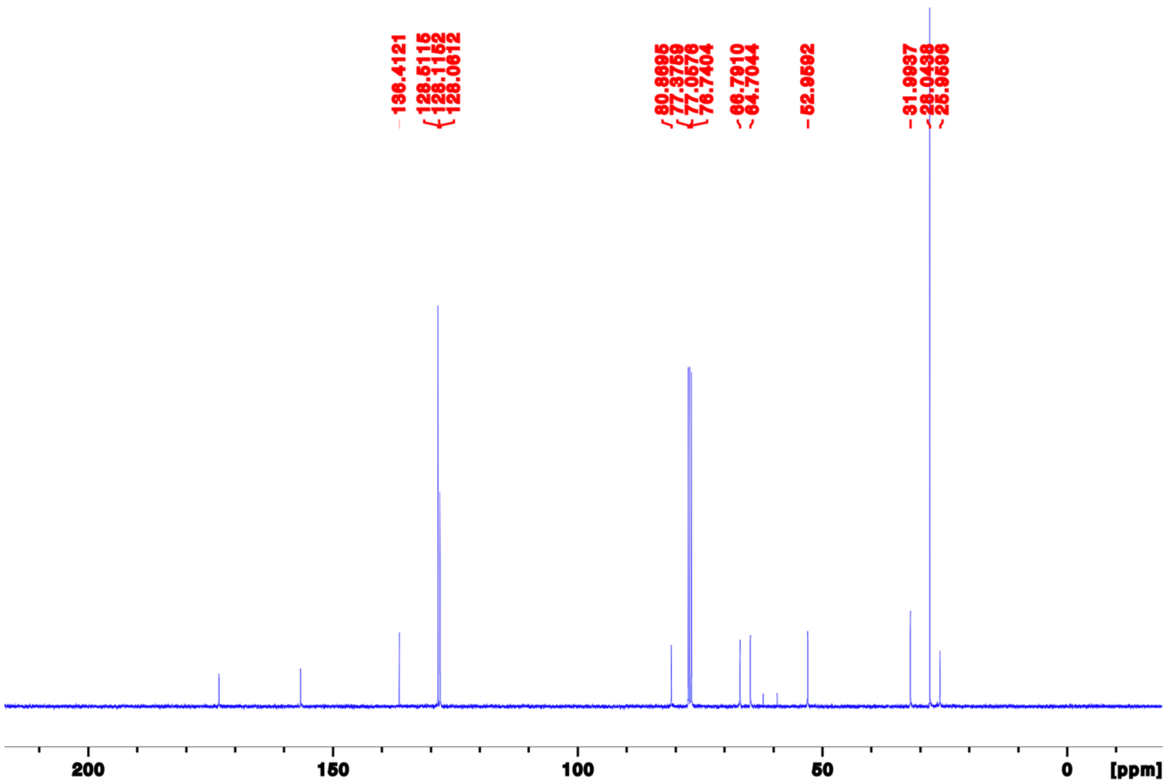

tert-Butyl (S)-5-hydroxy-4-((2-nitrophenyl)sulfonamido)pentanoate 23

^{1}H, 400 MHz, CDCl$_3$

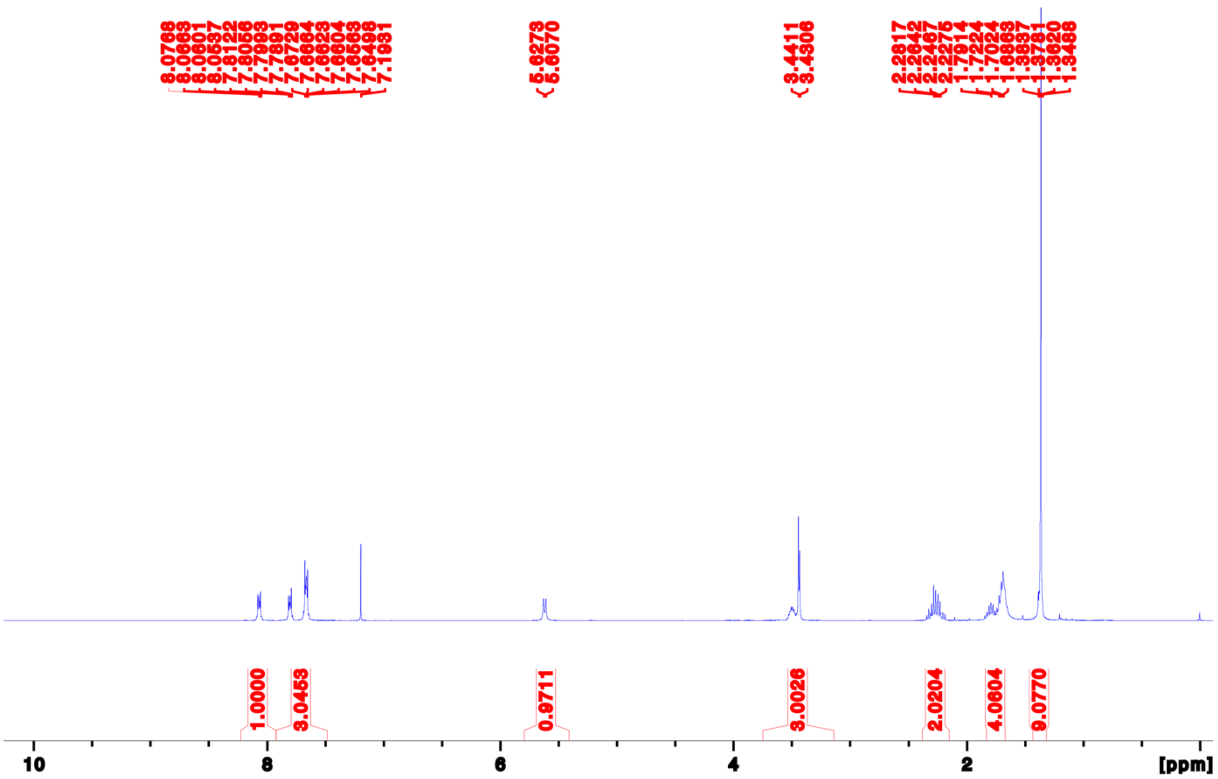

^{13}C, 100 MHz, CDCl$_3$

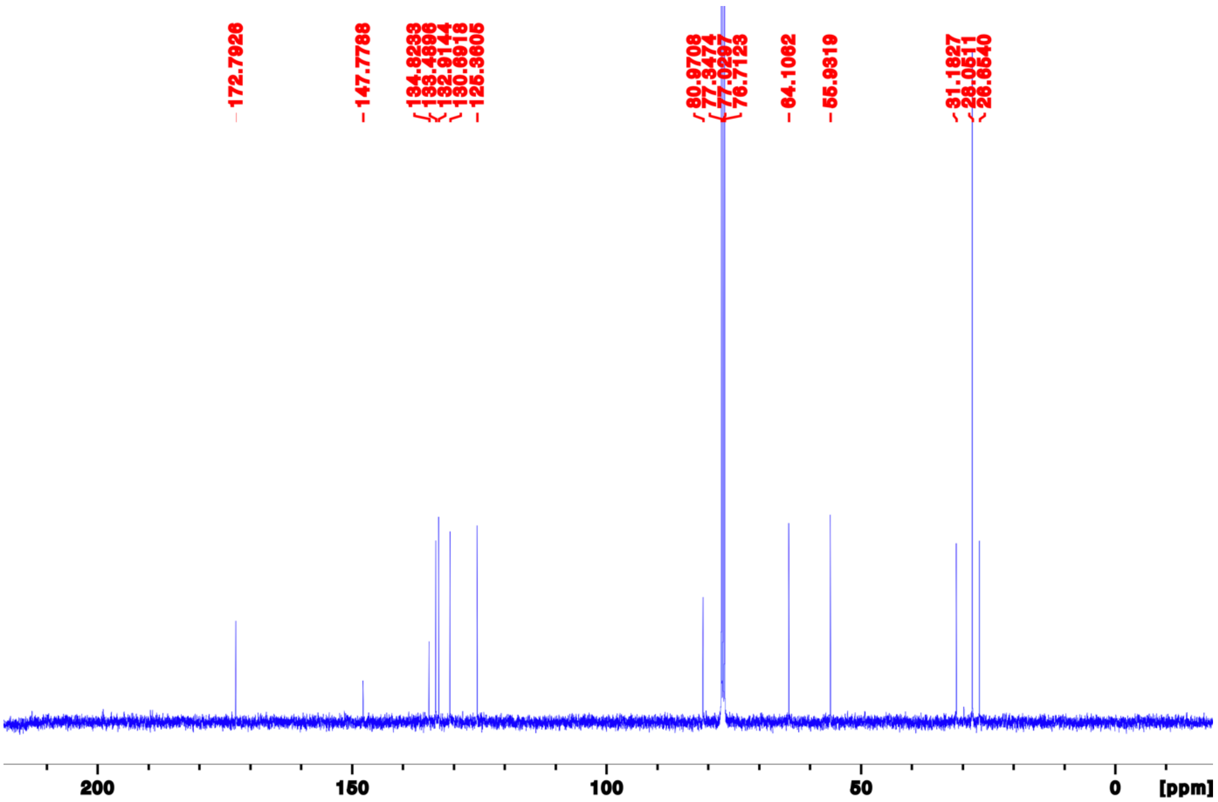

tert-Butyl (S)-5-hydroxy-4-((4-methylphenyl)sulfonamido)pentanoate 49

^{1}H, 400 MHz, $CDCl_3$

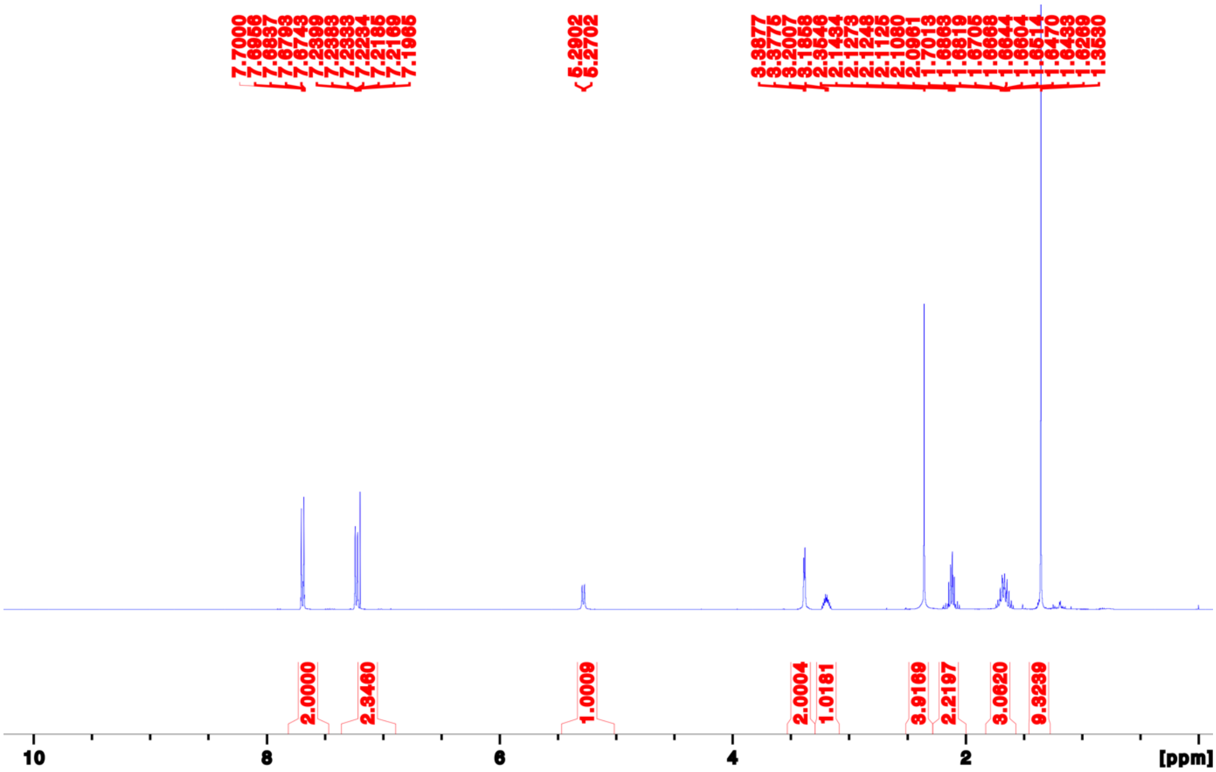

^{13}C, 100 MHz, $CDCl_3$

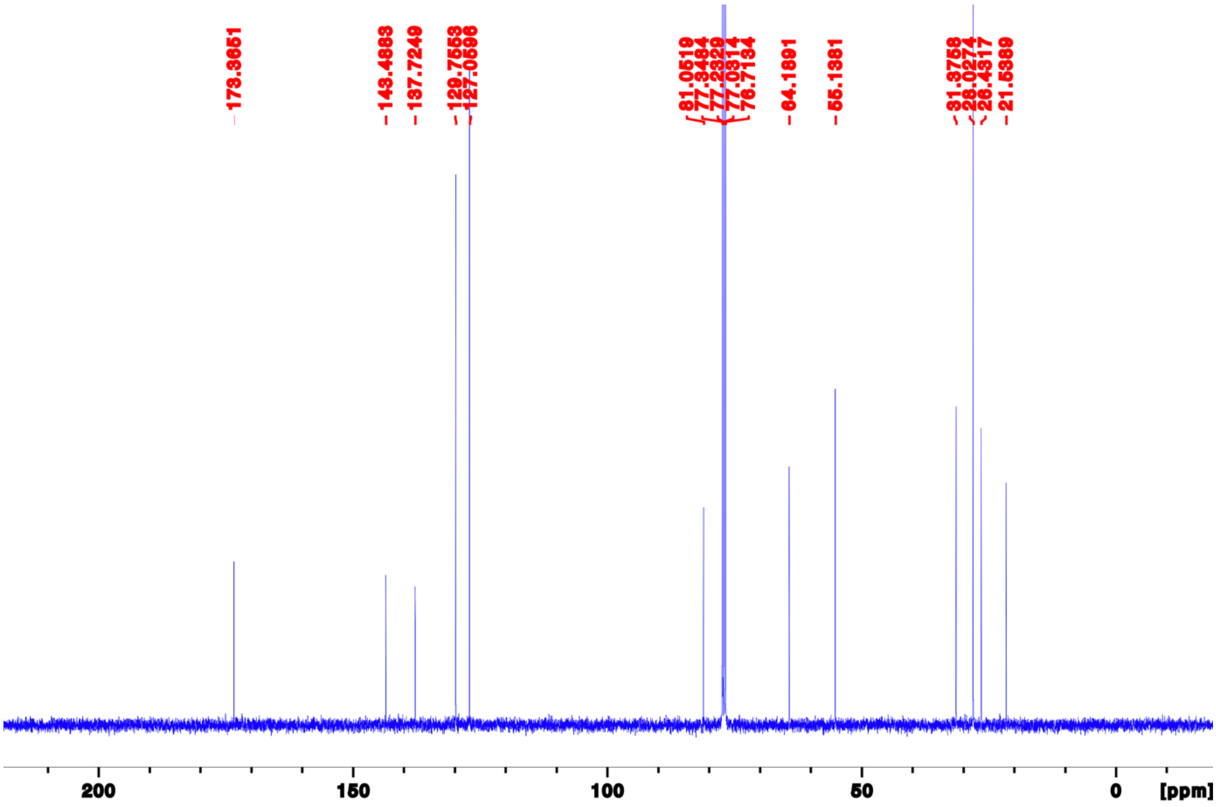

tert-Butyl (S)-3-(1-((2-nitrophenyl)sulfonyl)aziridin-2-yl)propanoate 25

^{1}H, 400 MHz, $CDCl_3$

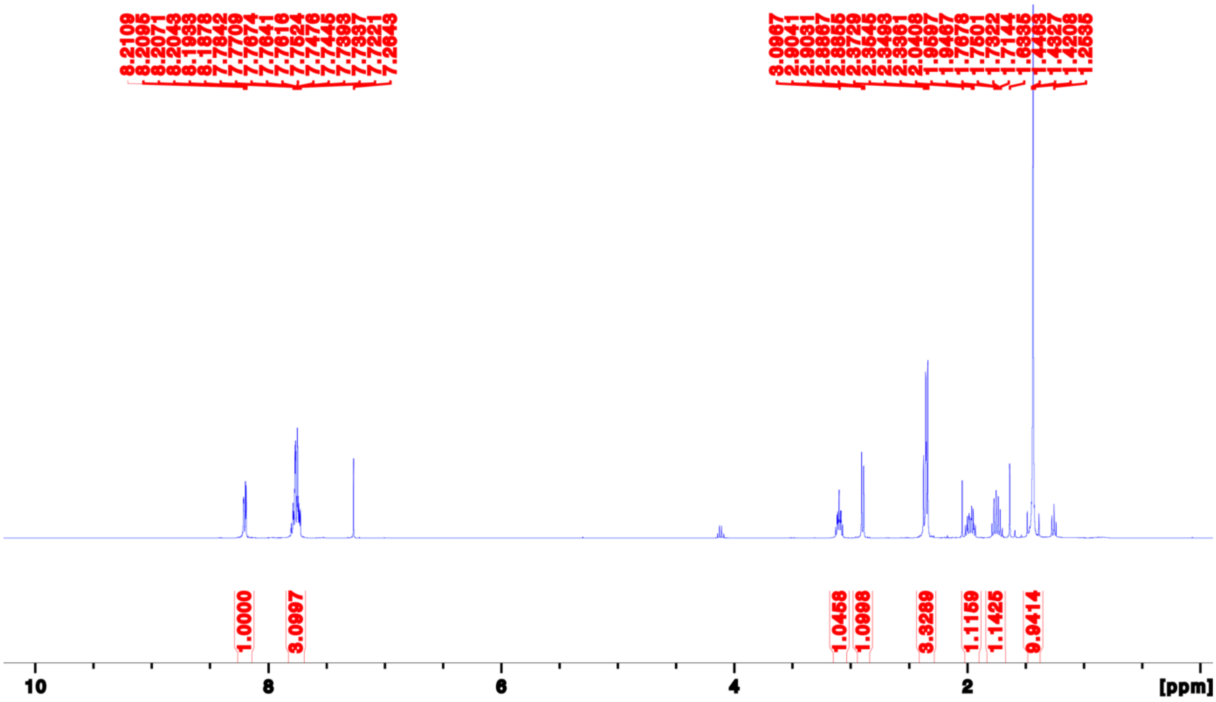

^{13}C, 100 MHz, $CDCl_3$

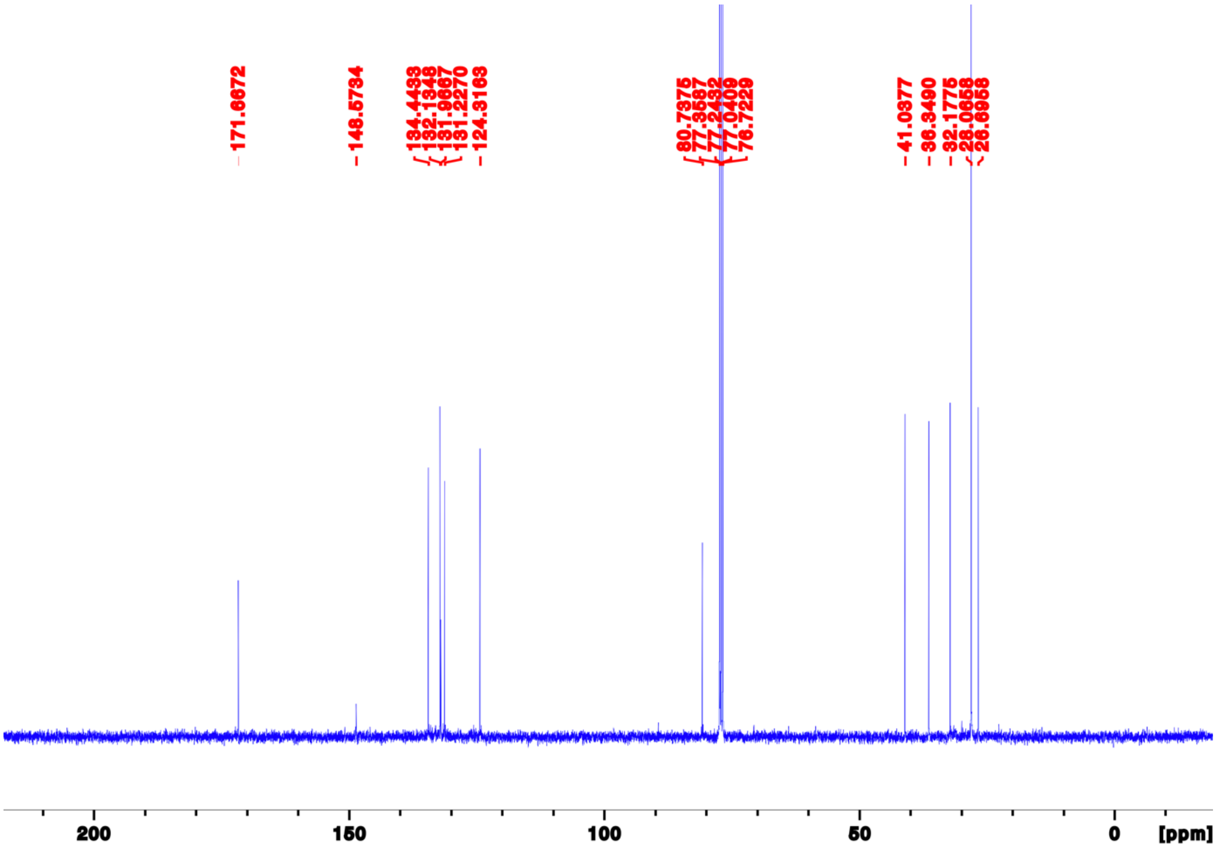

tert-Butyl (S)-4-((2-nitrophenyl)sulfonamido)-5-(phenylamino)pentanoate 27

^{1}H, 400 MHz, $CDCl_3$

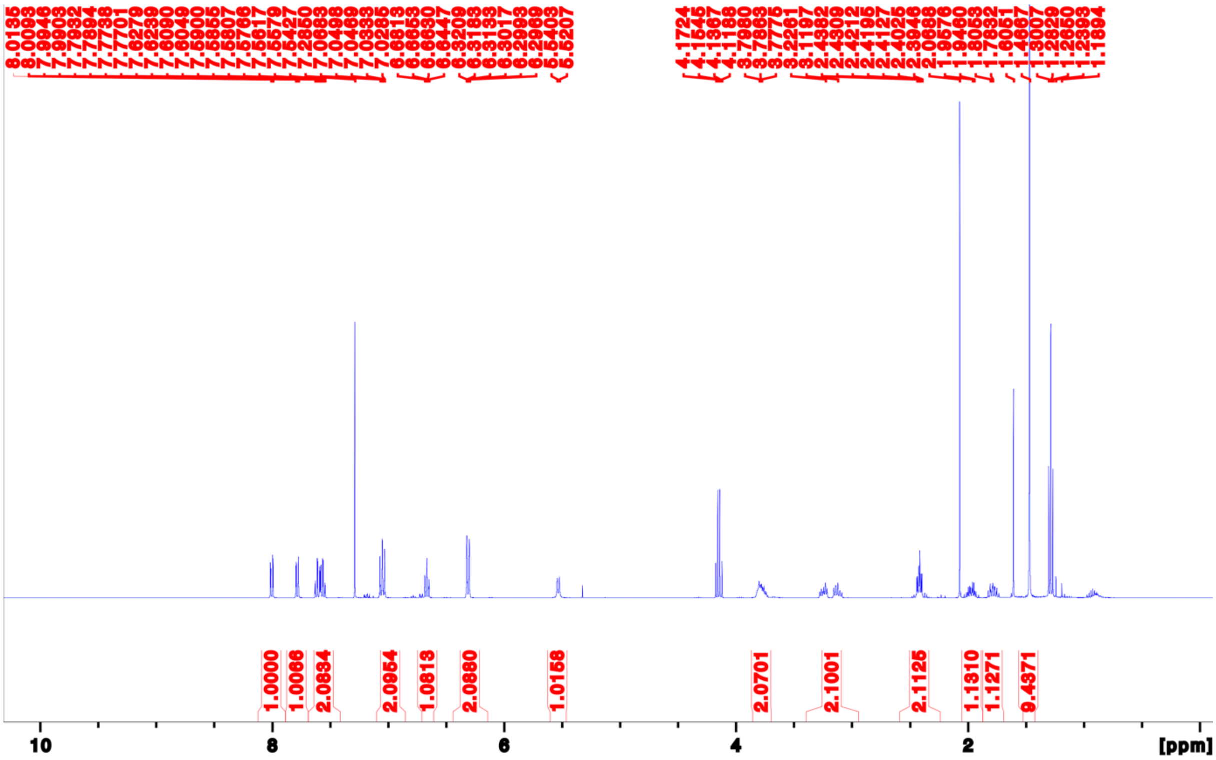

^{13}C, 100 MHz, $CDCl_3$

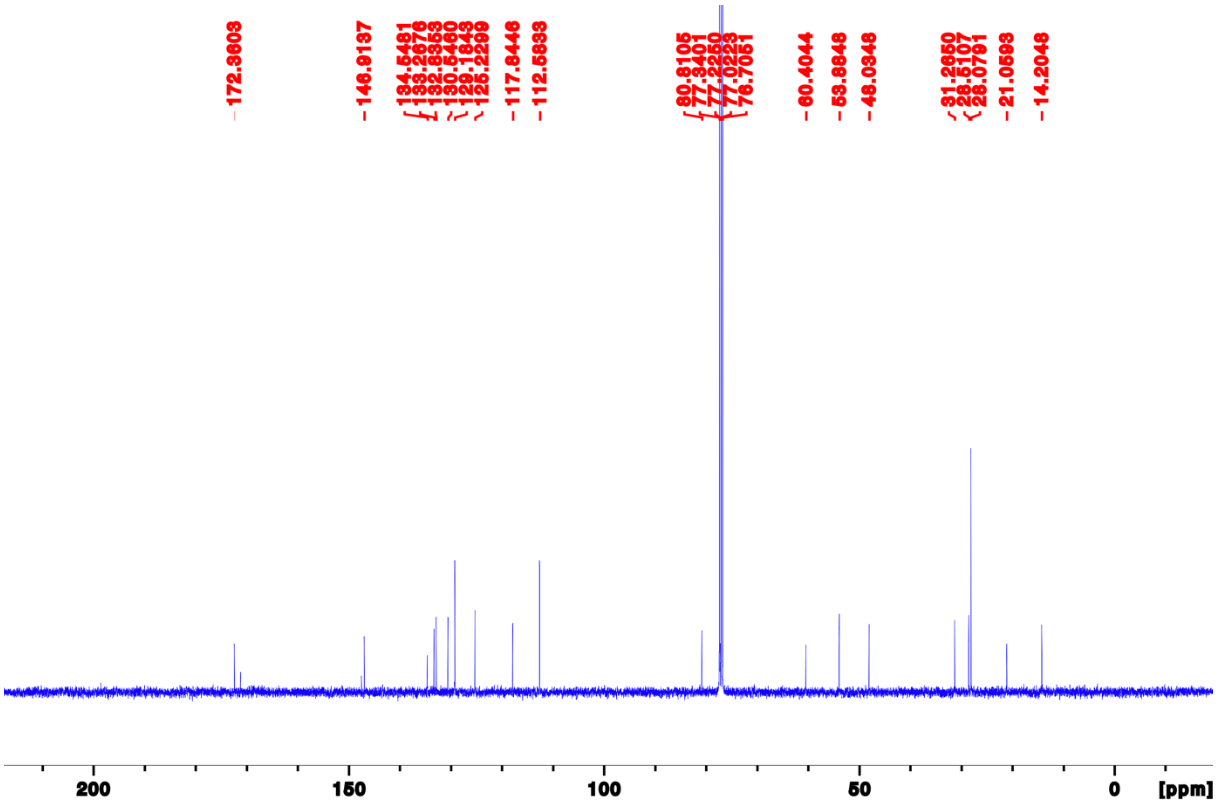

tert-Butyl (S)-3-(3-((2-nitrophenyl)sulfonyl)-2-oxo-1-phenylimidazolidin-4-yl)propanoate 50

^{1}H, 400 MHz, CDCl$_3$

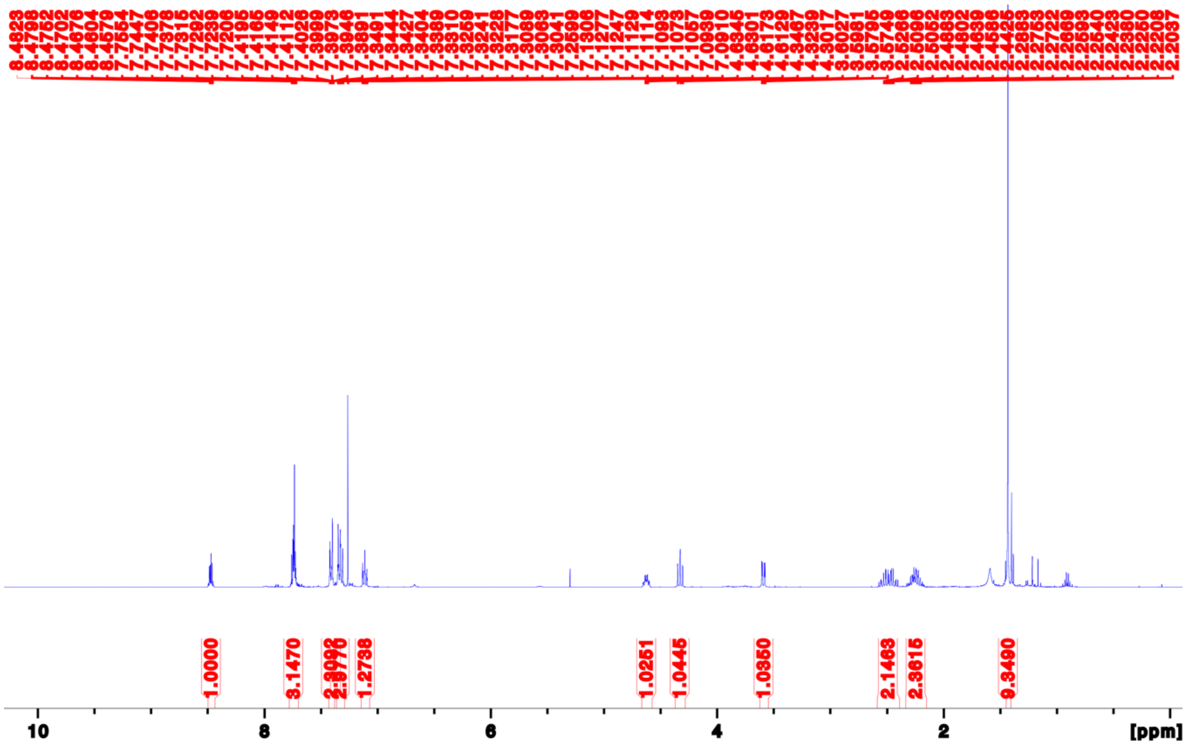

^{13}C, 100 MHz, CDCl$_3$

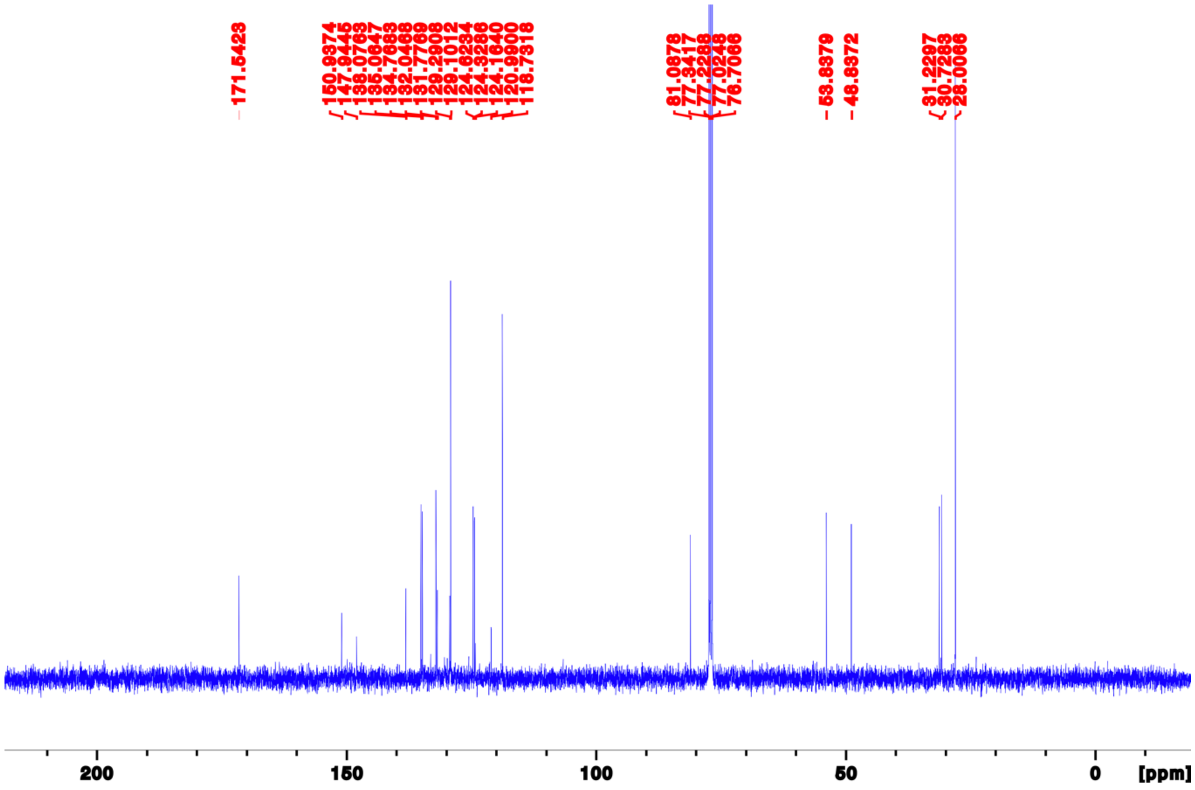

tert-Butyl (S)-3-(3-((2-nitrophenyl)sulfonyl)-2-oxoimidazolidin-4-yl)propanoate 33

^{1}H, 500 MHz, $CDCl_3$

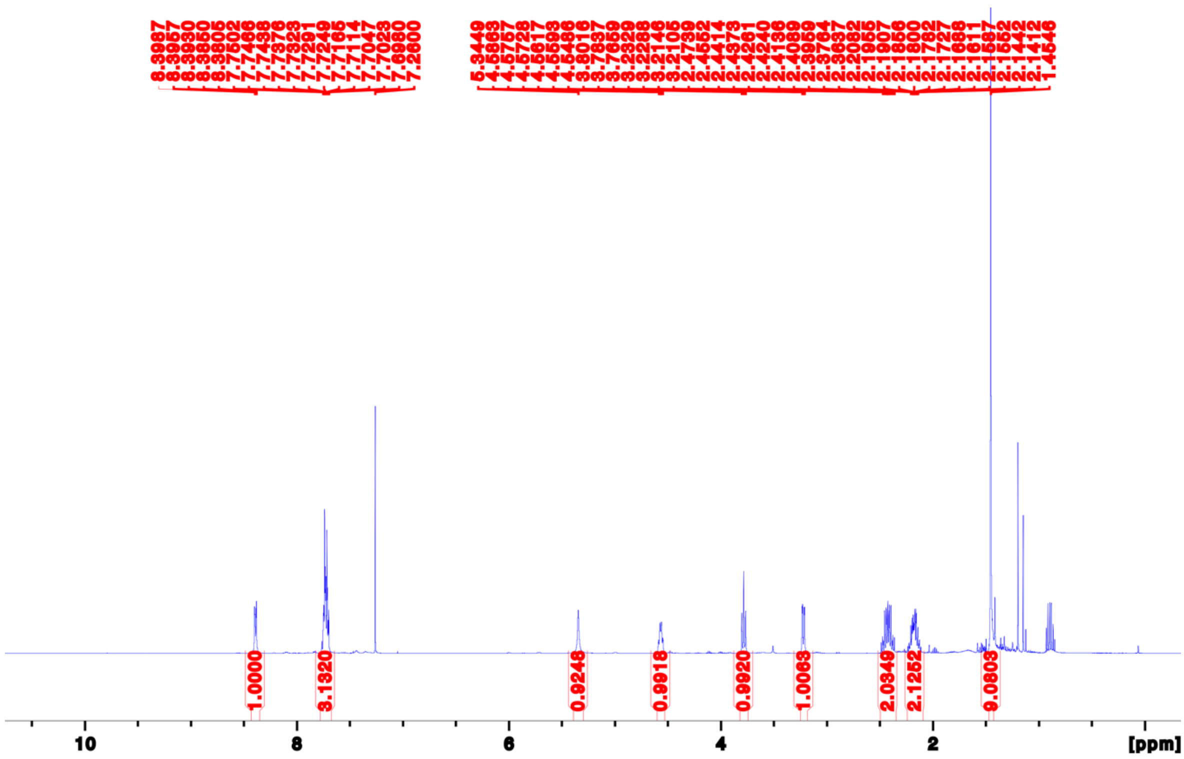

^{13}C, 125 MHz, $CDCl_3$

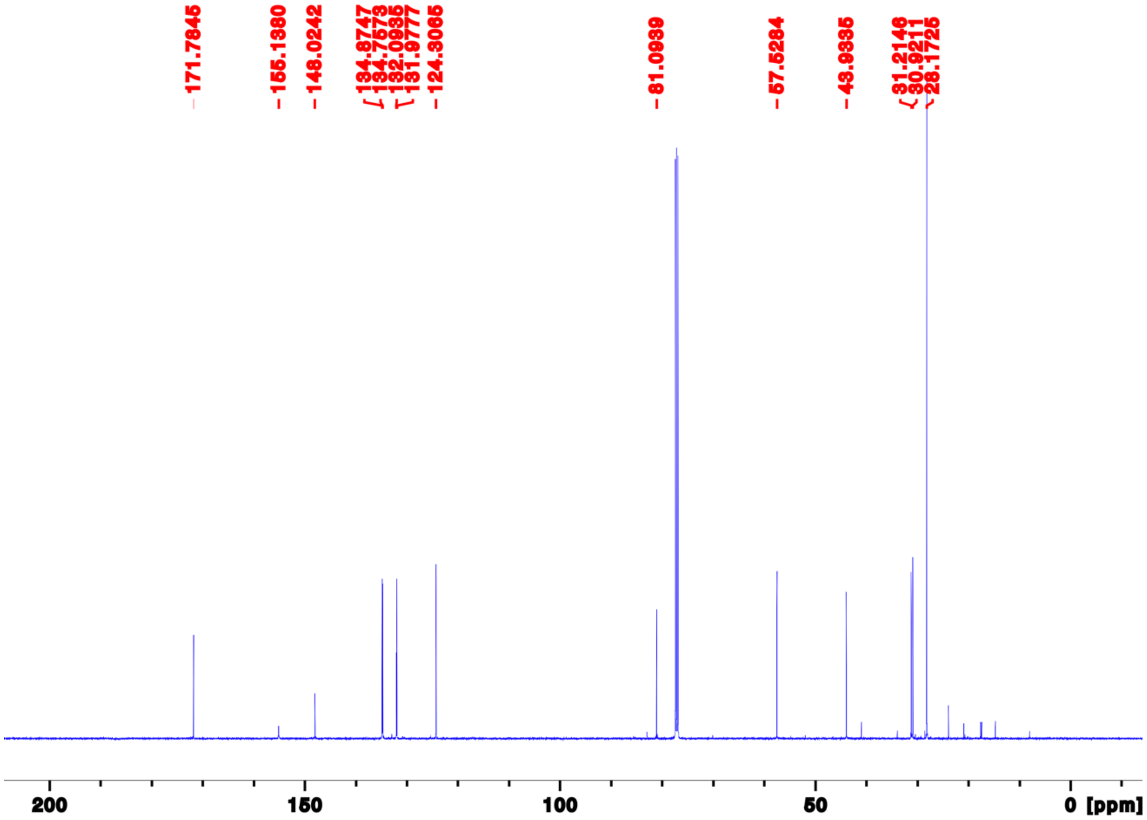

***tert*-Butyl (*S*)-3-(1-(6-bromopyridin-2-yl)-3-((2-nitrophenyl)sulfonyl)-2-oxoimidazolidin-4-yl)propanoate 35**

^{1}H, 400 MHz, $CDCl_3$

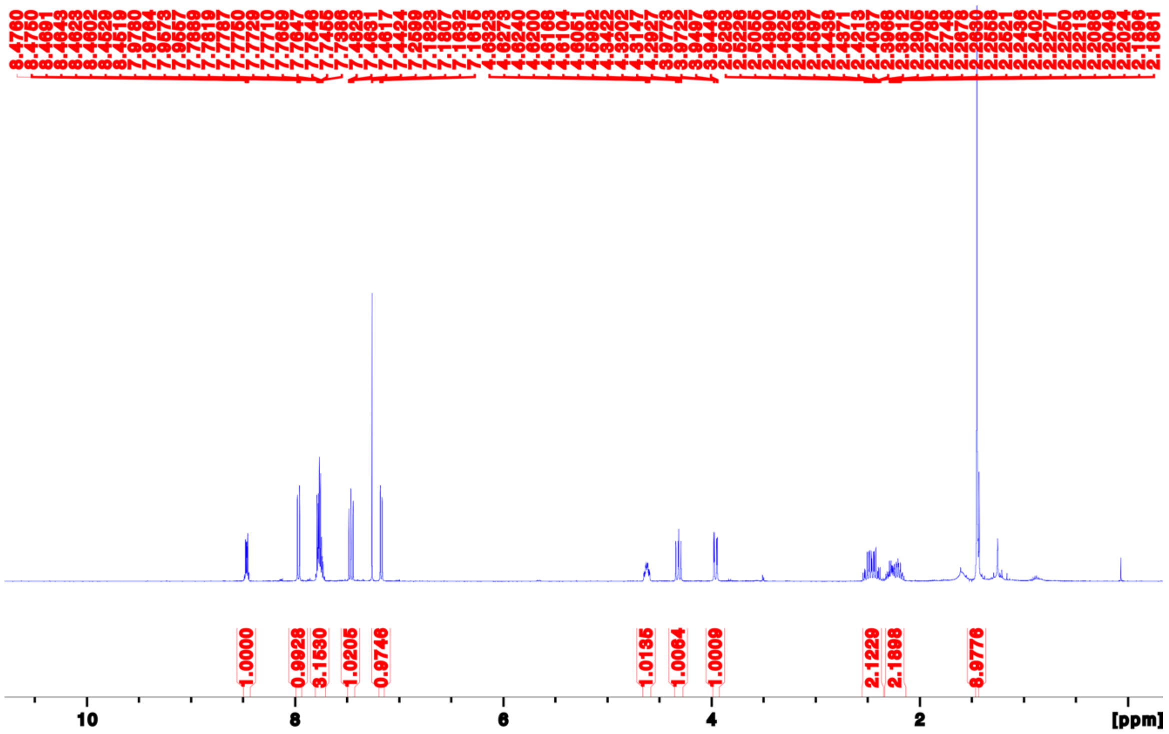

^{13}C, 100 MHz, $CDCl_3$

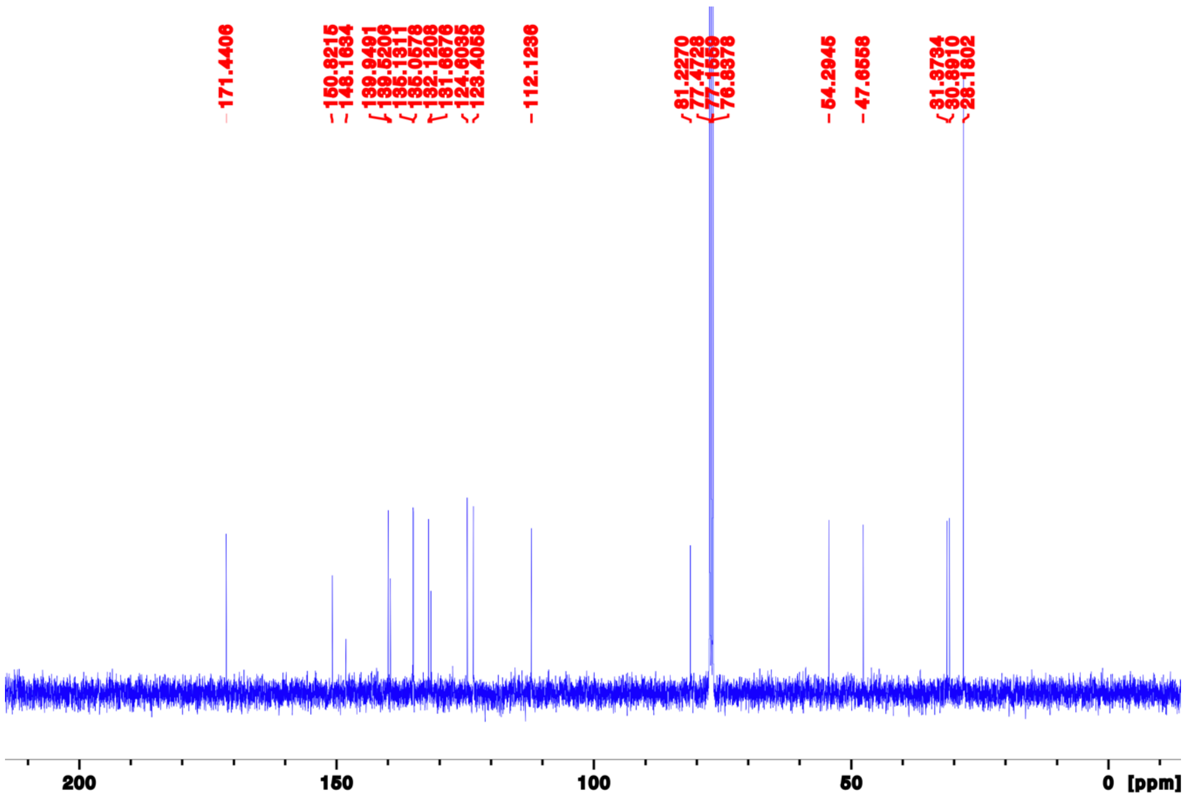

tert-Butyl (S)-3-(2-oxo-1-phenylimidazolidin-4-yl)propanoate 29

^{1}H, 400 MHz, $CDCl_3$

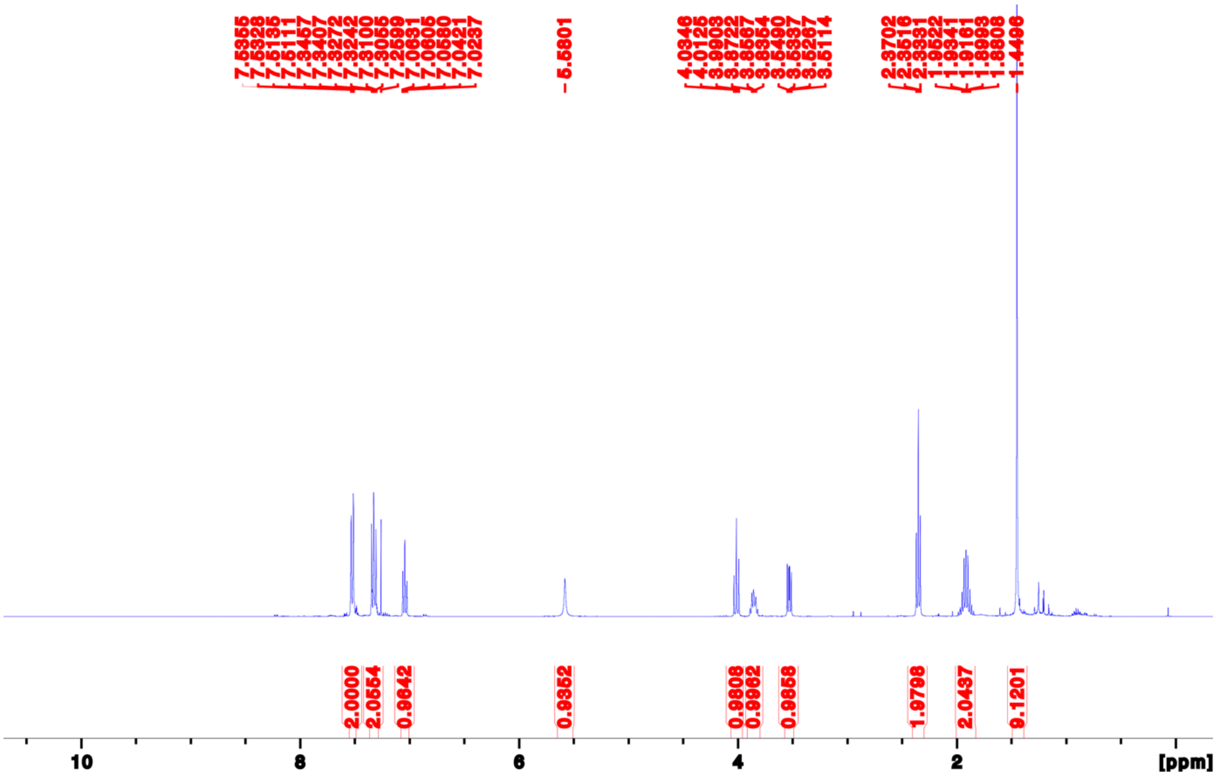

^{13}C, 100 MHz, $CDCl_3$

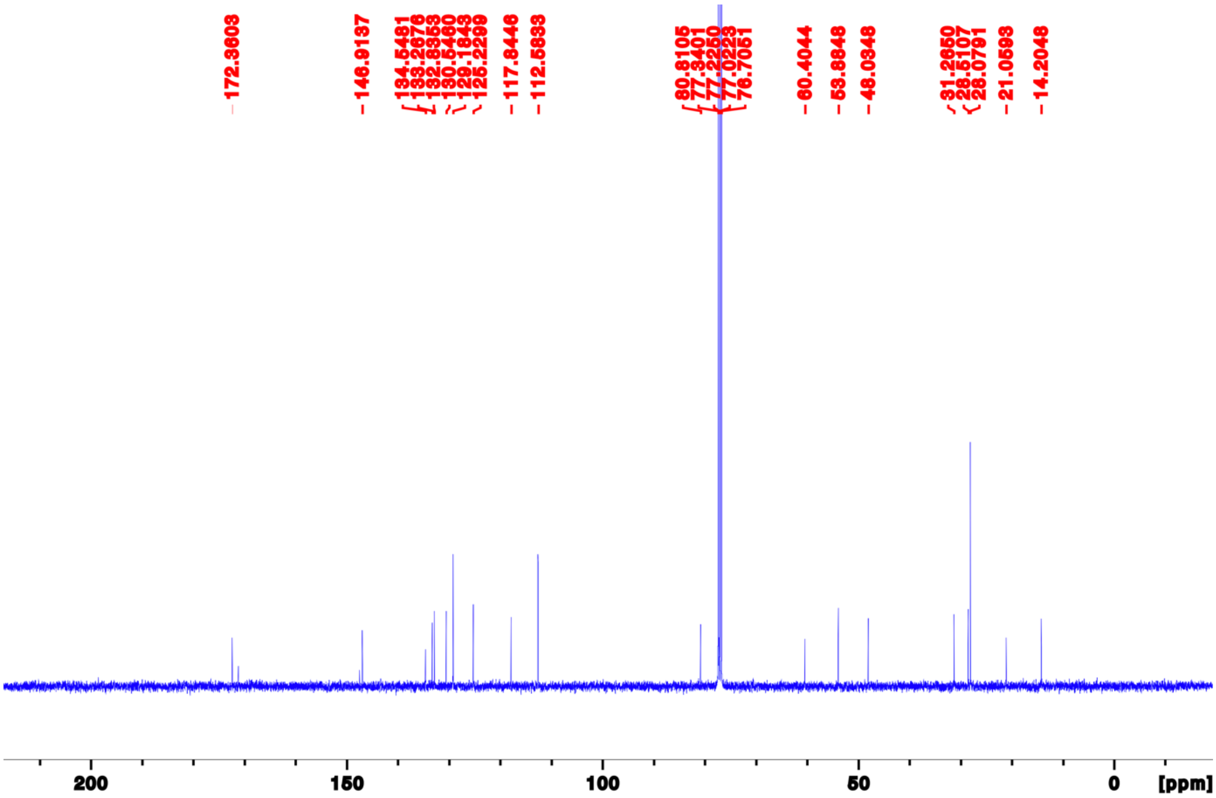

tert-Butyl 3-((S)-1-(6-((S)-5-(3-(tert-butoxy)-3-oxopropyl)-2-oxo-3-phenylimidazolidin-1-yl)pyridin-2-yl)-3-((2-nitrophenyl)sulfonyl)-2-oxoimidazolidin-4-yl)propanoate 37

^{1}H, 400 MHz, $CDCl_3$

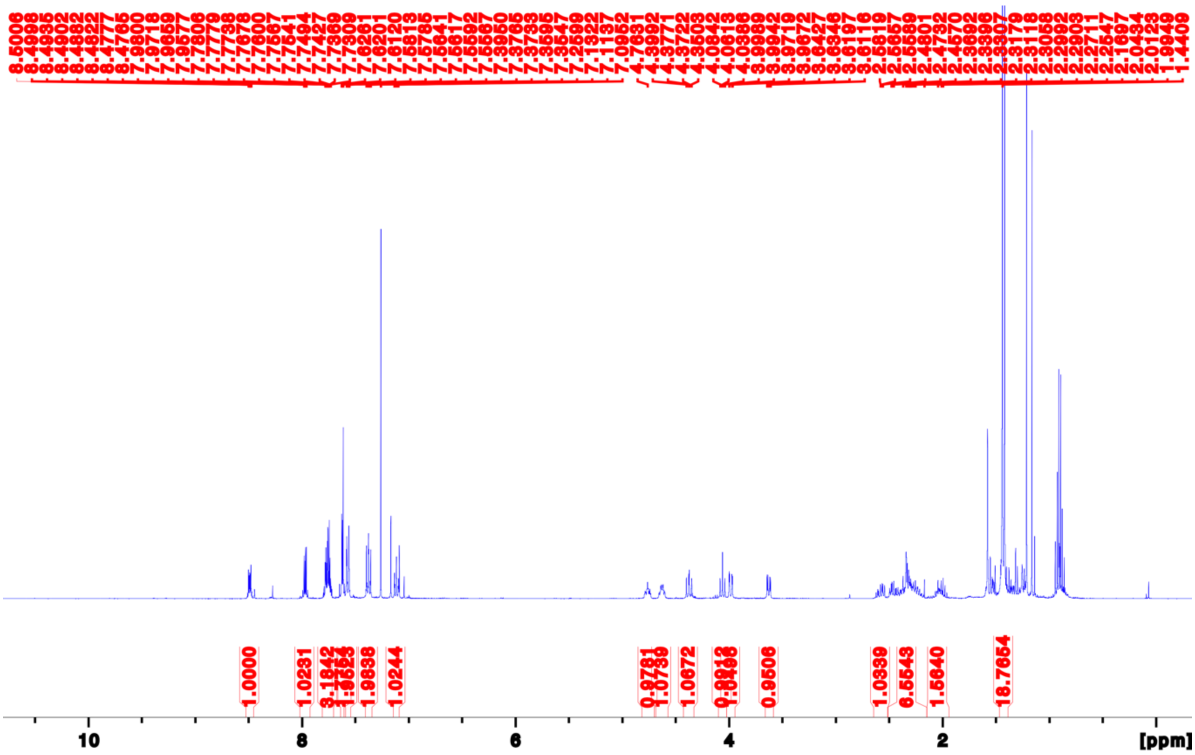

^{13}C, 100 MHz, $CDCl_3$

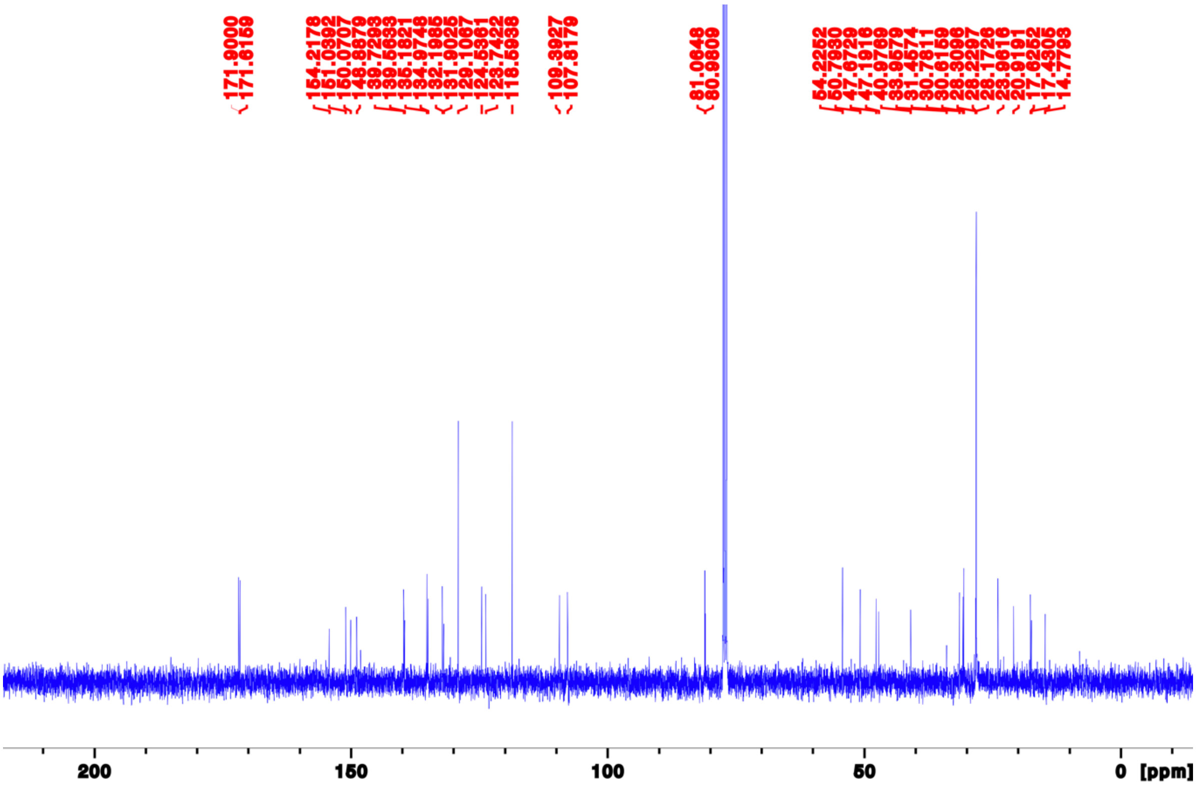

tert-Butyl 3-((_S_)-1-(6-((_S_)-5-(3-(_tert_-butoxy)-3-oxopropyl)-2-oxo-3-phenylimidazolidin-1-yl)pyridin-2-yl)-2-oxoimidazolidin-4-yl)propanoate 38

^{1}H, 400 MHz, $CDCl_3$

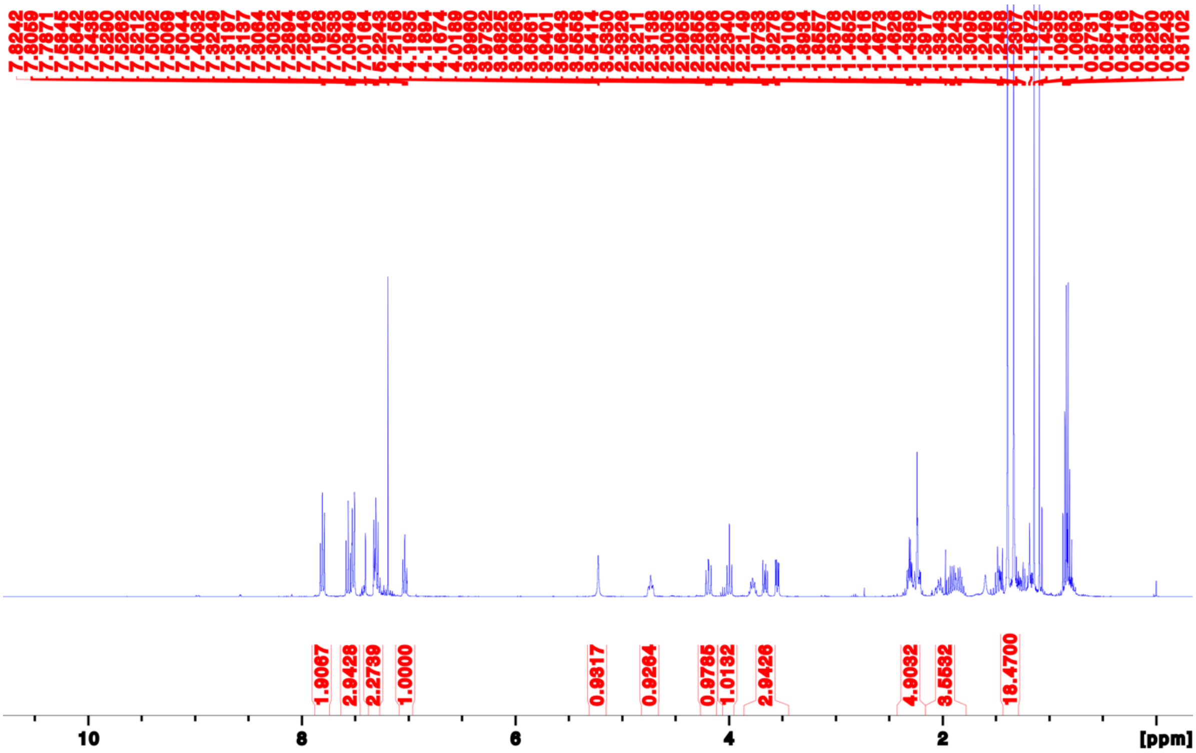

^{13}C, 100 MHz, $CDCl_3$

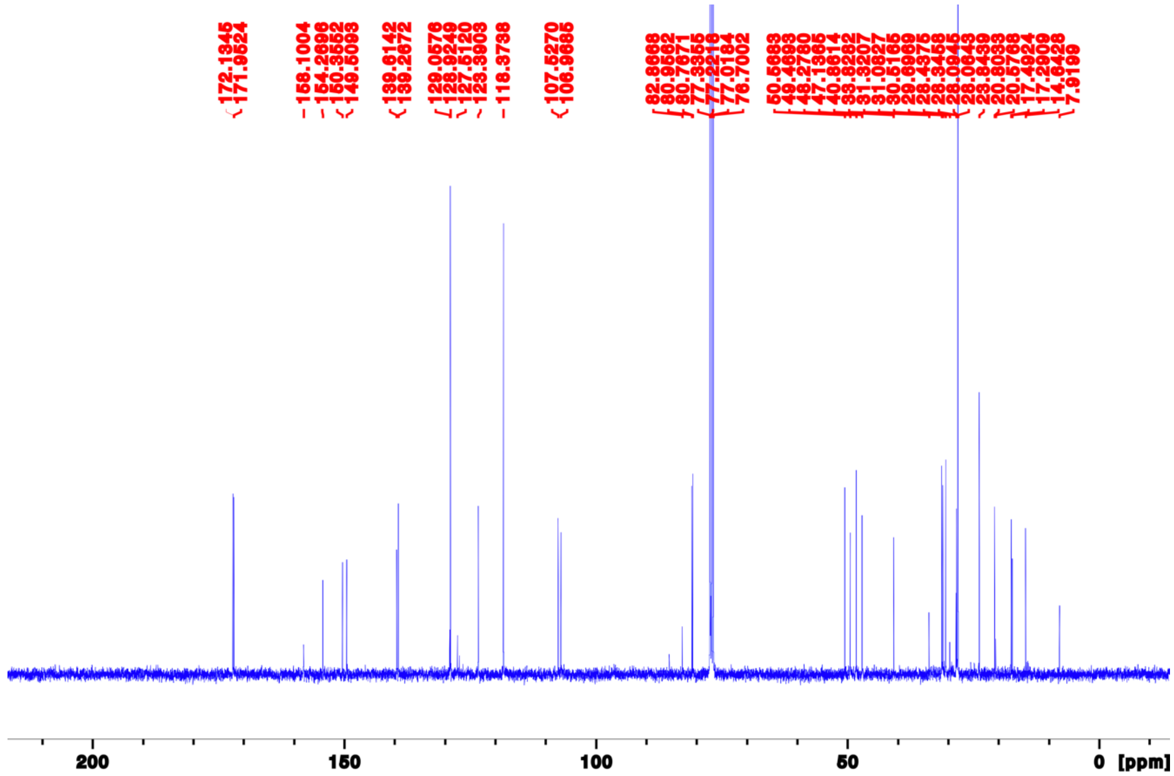

***tert*-Butyl 3-((*S*)-1-(6-((*S*)-5-(3-(*tert*-butoxy)-3-oxopropyl)-2-oxo-3-phenylimidazolidin-1-yl)pyridin-2-yl)-3-(6-((*S*)-4-(3-(*tert*-butoxy)-3-oxopropyl)-3-((4-nitrophenyl)sulfonyl)-2-oxoimidazolidin-1-yl)pyridin-2-yl)-2-oxoimidazolidin-4-yl)propanoate 51**

^{1}H, 500 MHz, CDCl$_3$

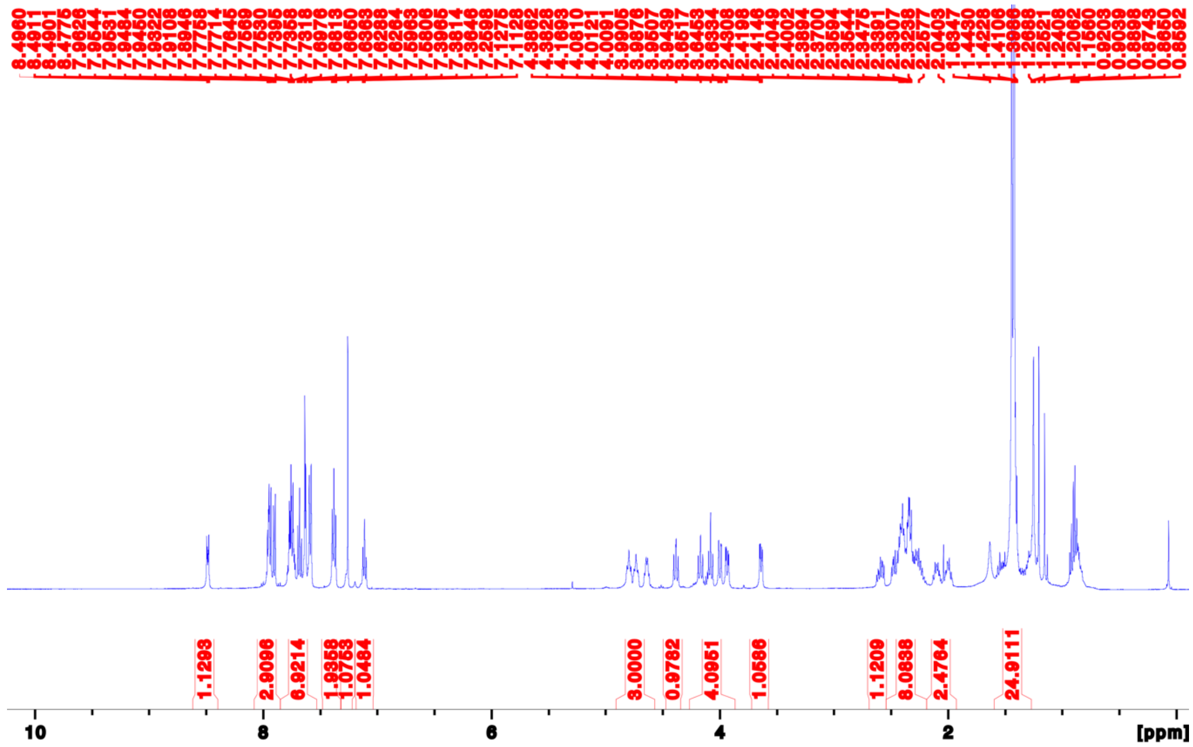

^{13}C, 125 MHz, CDCl$_3$

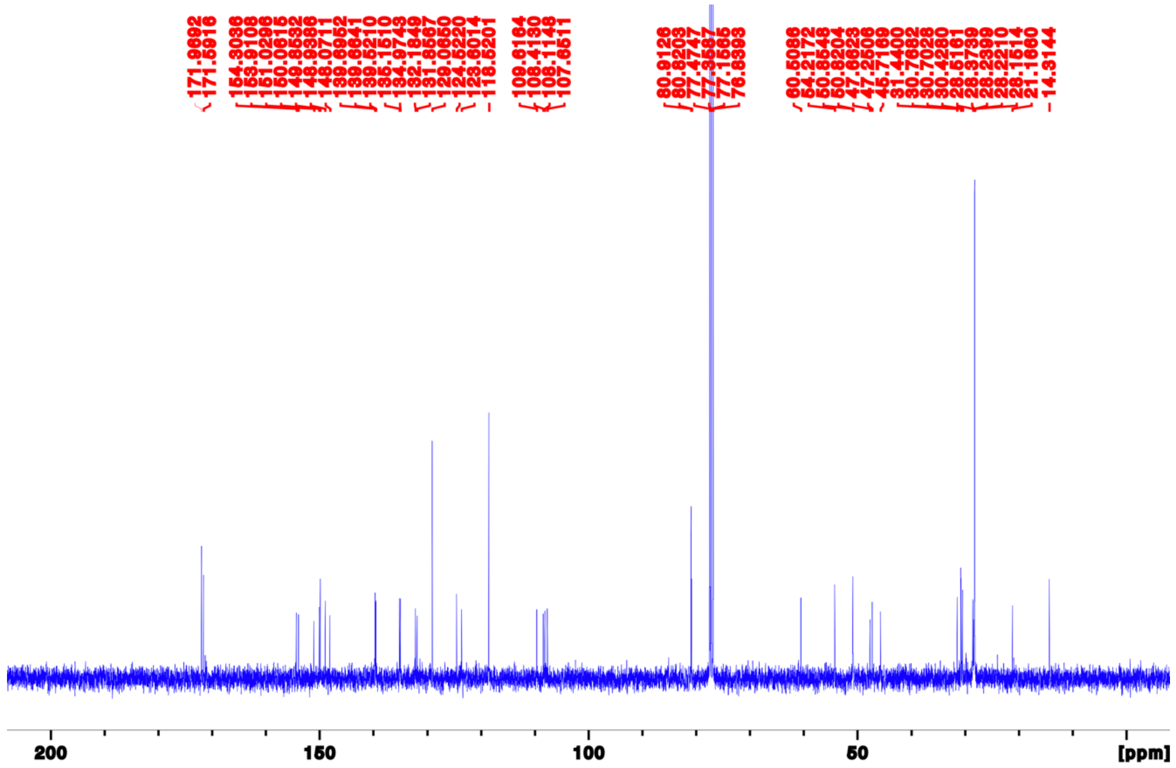

3-((S)-1-(6-((S)-5-(2-Carboxyethyl)-2-oxo-3-phenylimidazolidin-1-yl)pyridin-2-yl)-3-(6-((S)-4-(2-carboxyethyl)-3-((2-nitrophenyl)sulfonyl)-2-oxoimidazolidin-1-yl)pyridin-2-yl)-2-oxoimidazolidin-4-yl)propanoic acid 39

^{1}H, 500 MHz, CD_3OD

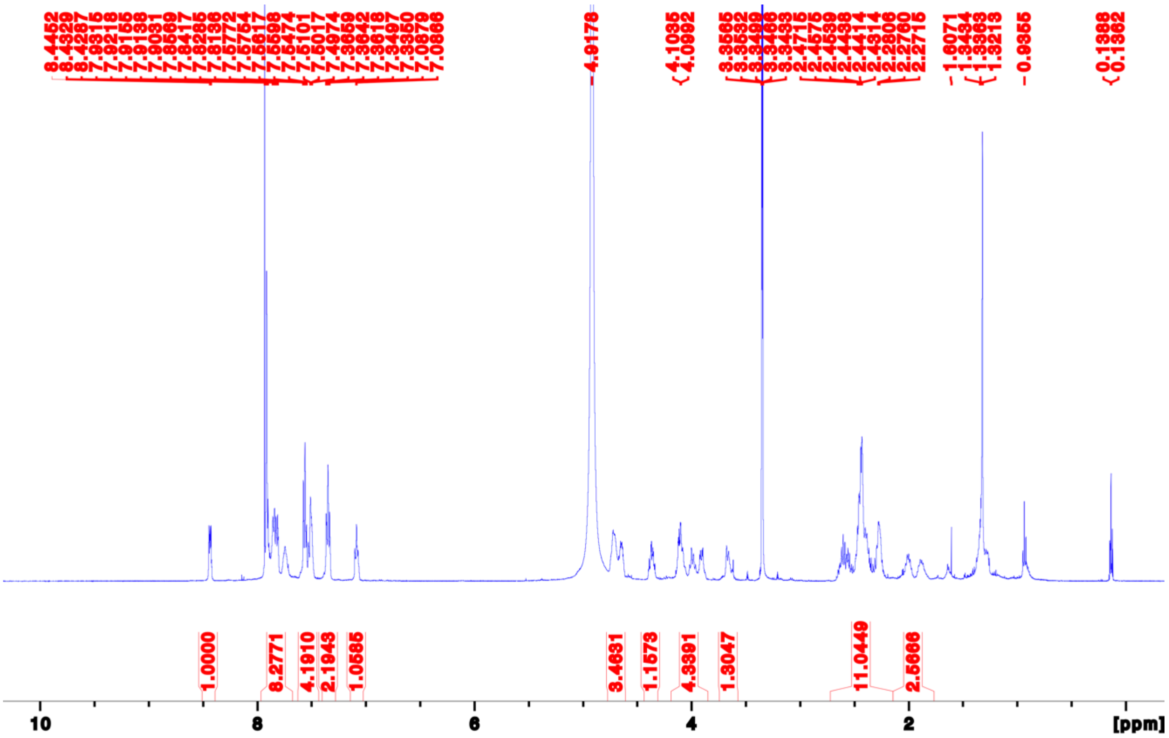

^{13}C, 125 MHz, CD_3OD

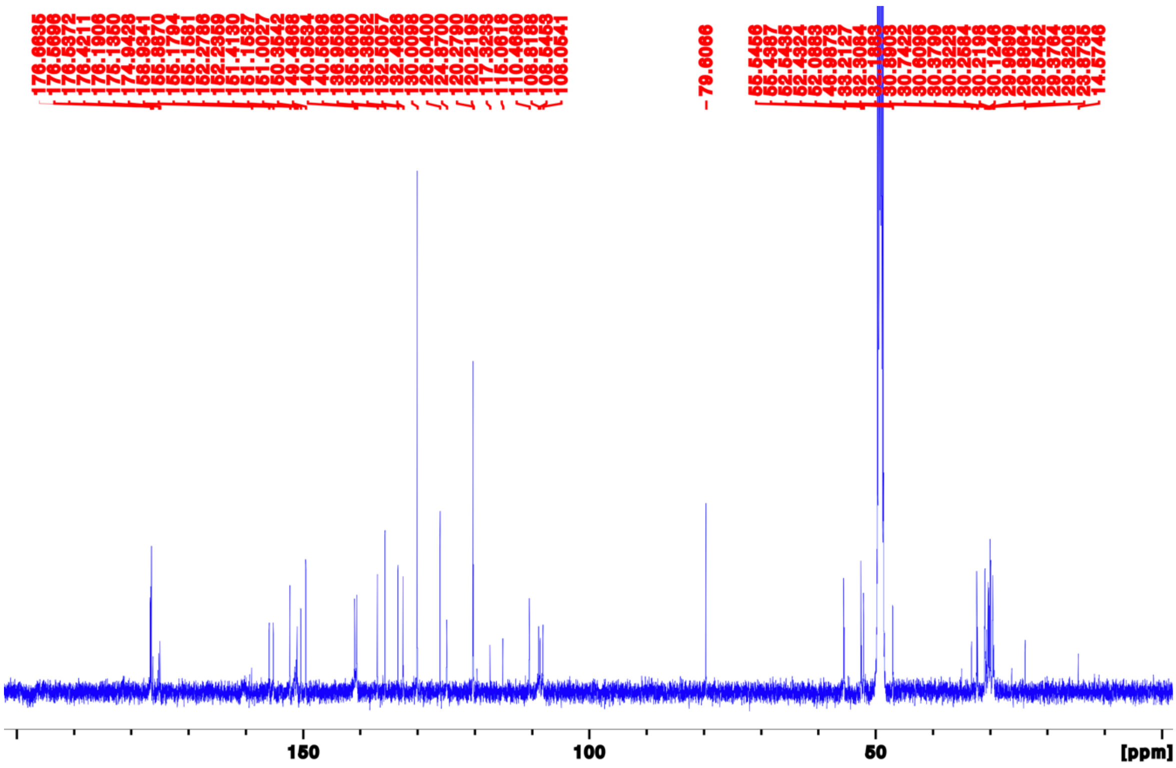

^{1}H, 500 MHz, pH 7.4 phosphate buffer

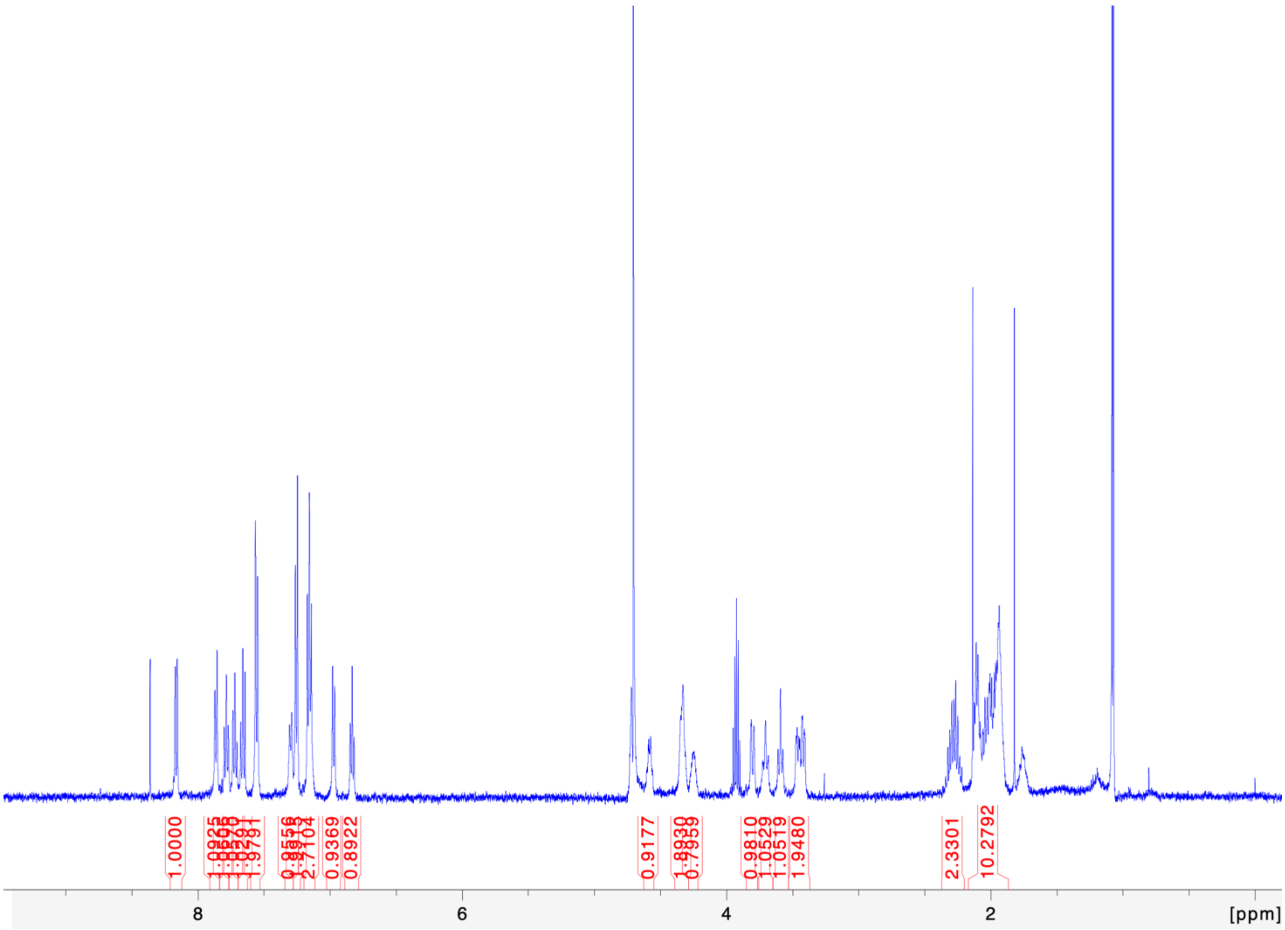

(S)-N-(1-Hydroxy-4-methylpentan-2-yl)-2-nitrobenzenesulfonamide 24

^{1}H, 400 MHz, $CDCl_3$

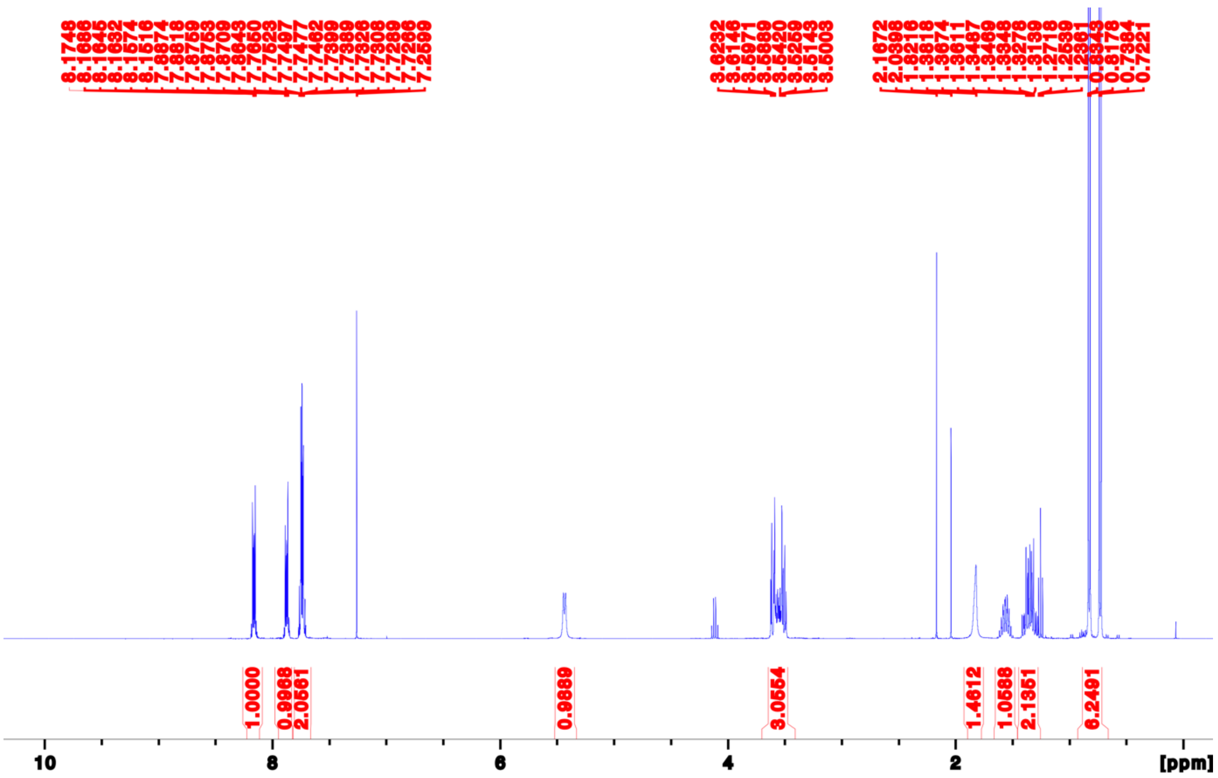

^{13}C, 100 MHz, $CDCl_3$

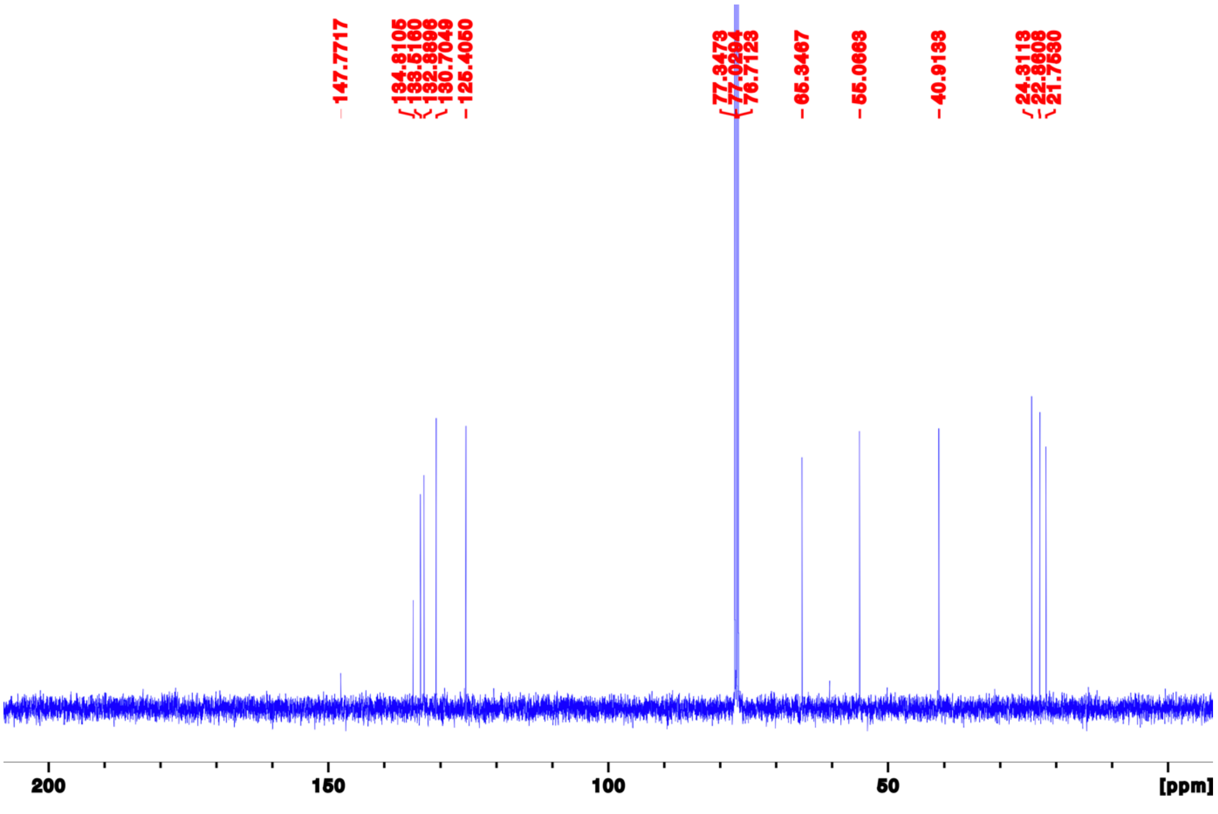

(S)-2-Isobutyl-1-((2-nitrophenyl)sulfonyl)aziridine 26

^{1}H, 500 MHz, $CDCl_3$

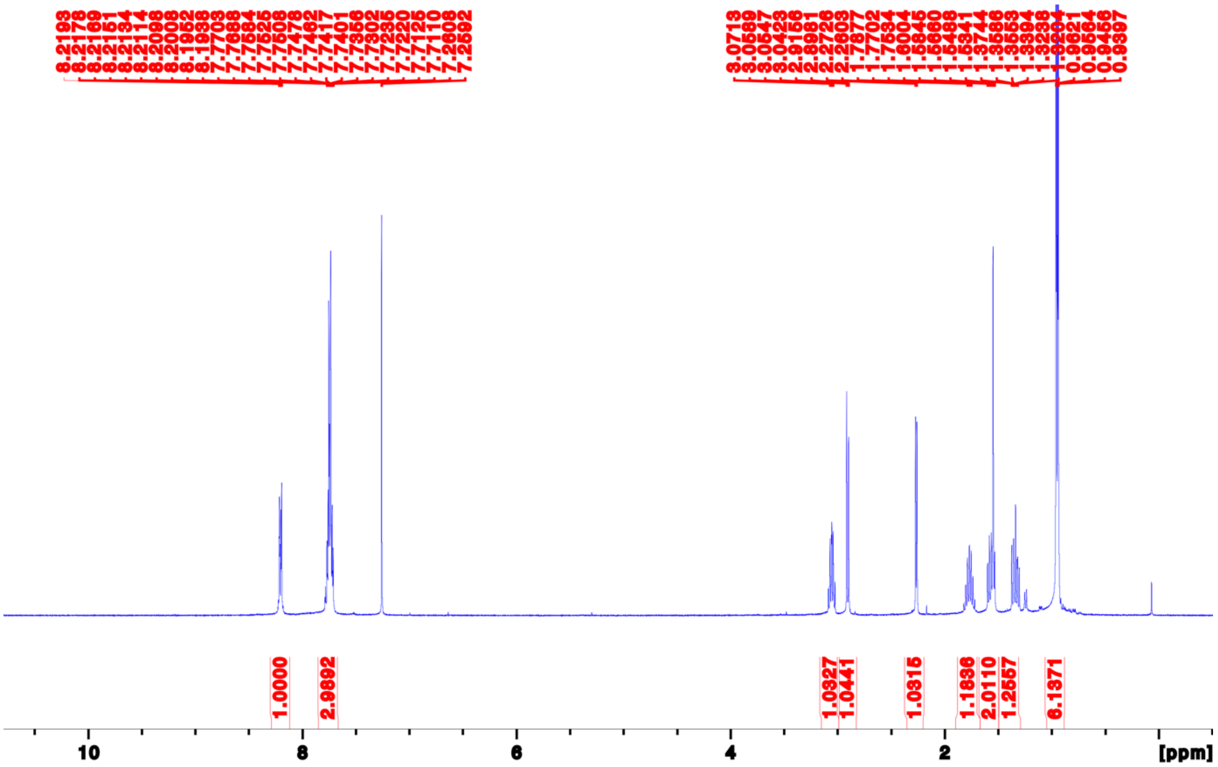

^{13}C, 125 MHz, $CDCl_3$

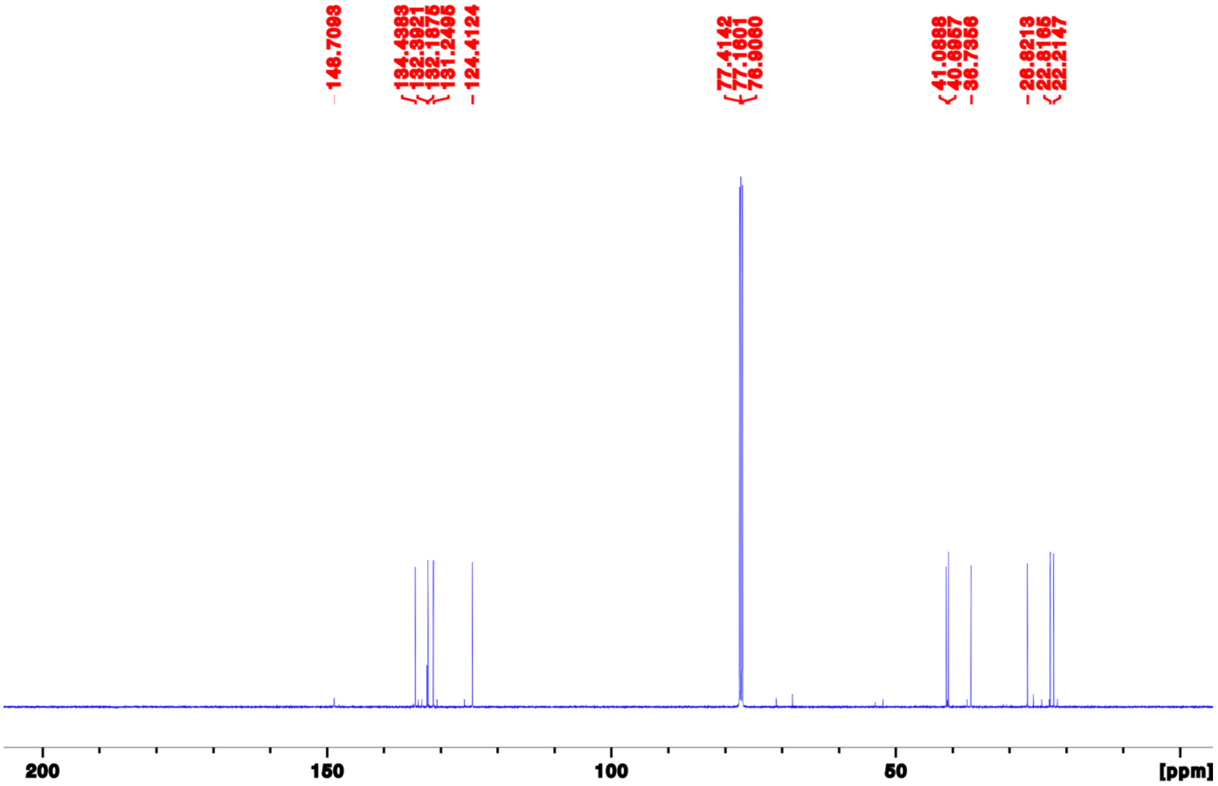

(S)-5-Isobutyl-1-((2-nitrophenyl)sulfonyl)imidazolidin-2-one 34

^{1}H, 400 MHz, $CDCl_3$

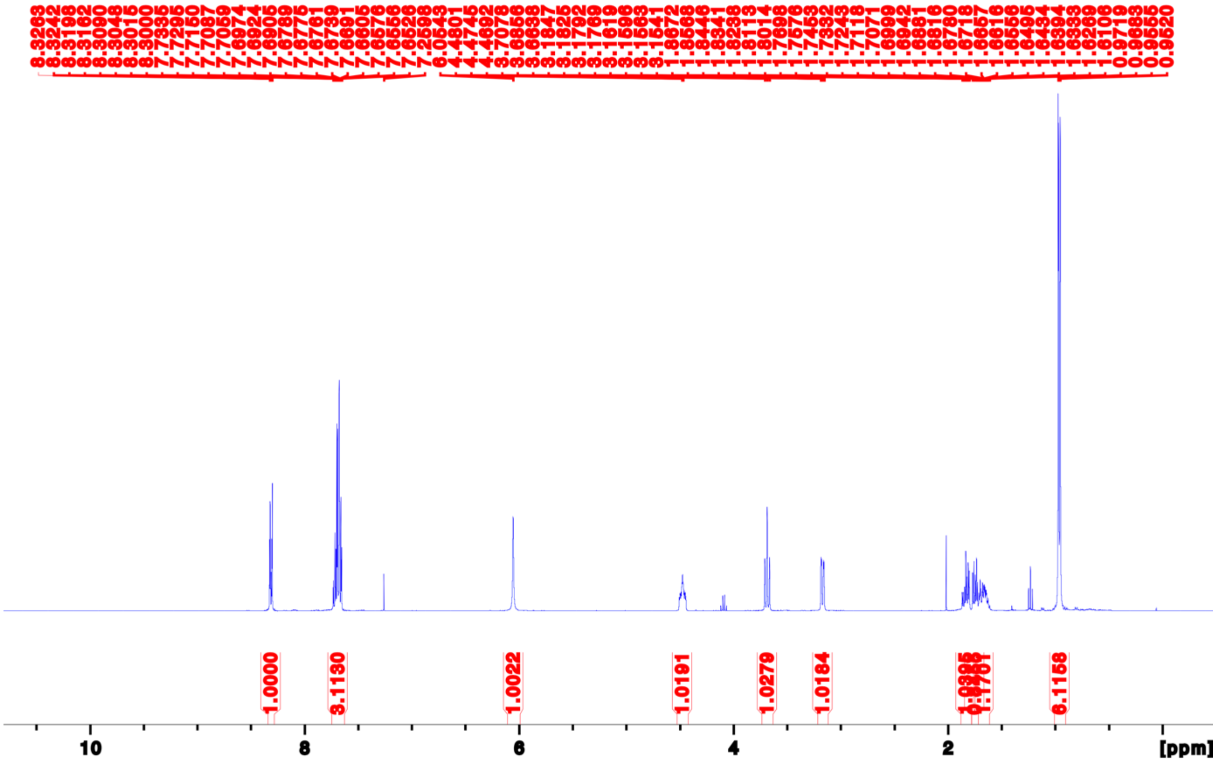

^{13}C, 100 MHz, $CDCl_3$

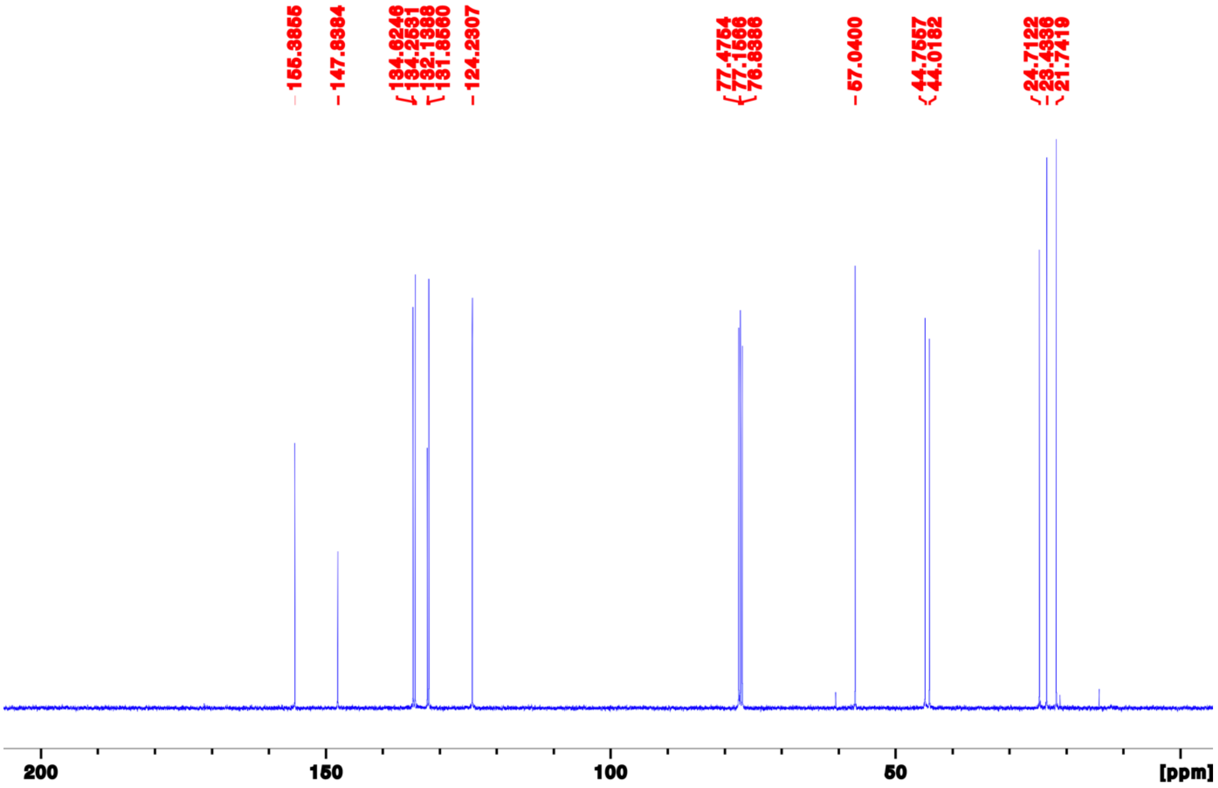

(S)-1-(6-Bromopyridin-2-yl)-4-isobutyl-3-((4-nitrophenyl)sulfonyl)imidazolidin-2-one 36

^{1}H, 400 MHz, $CDCl_3$

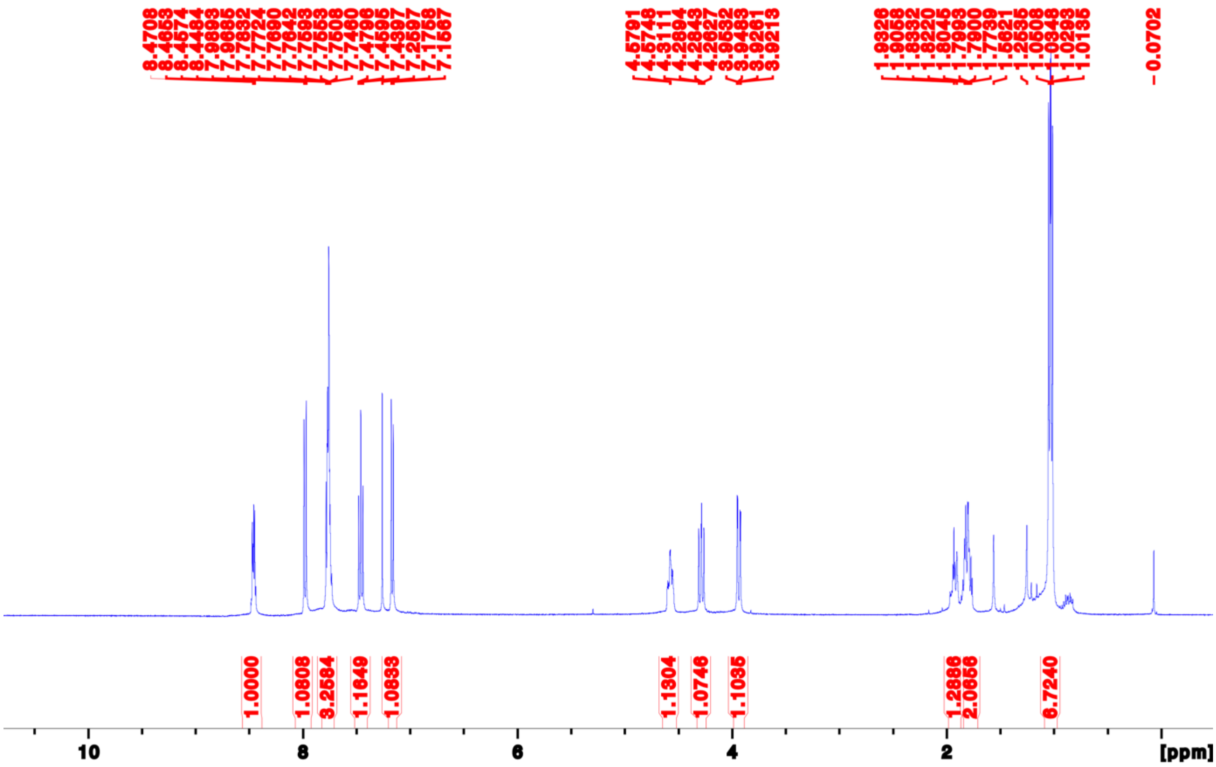

^{13}C, 100 MHz, $CDCl_3$

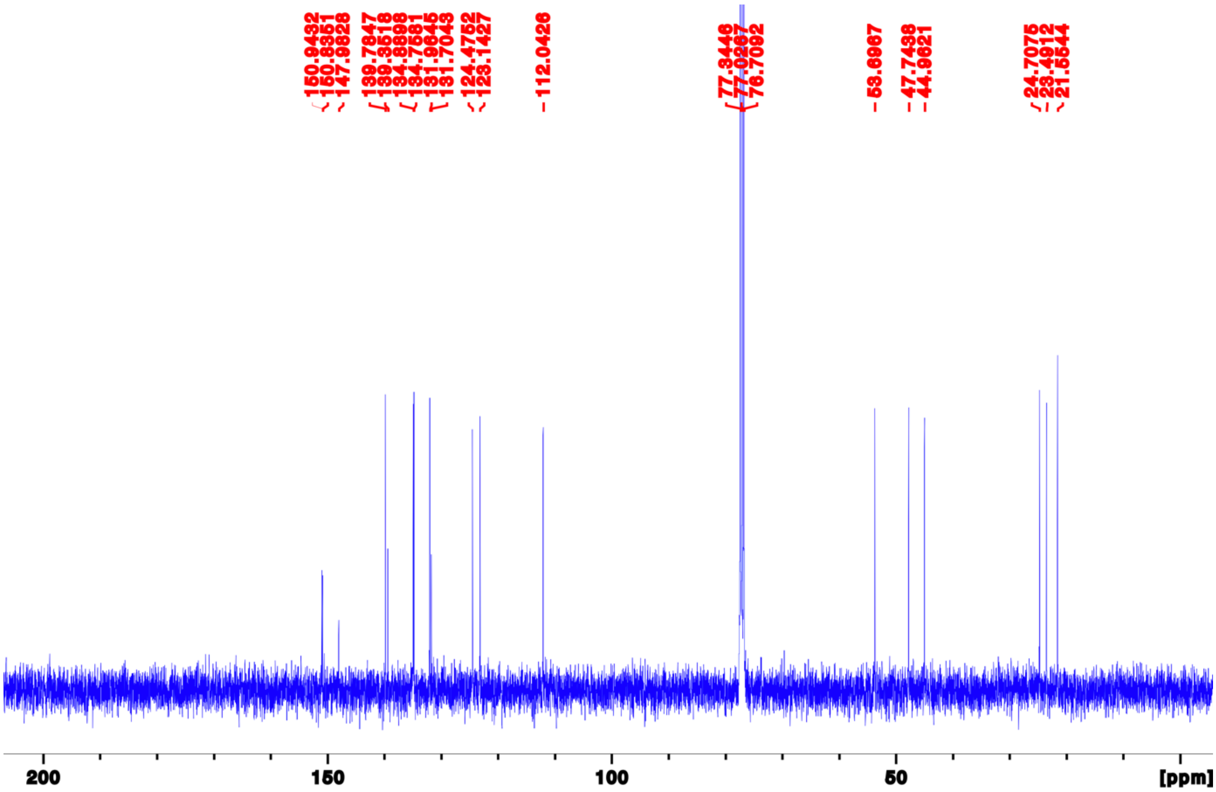

tert-Butyl 3-((S)-1-(6-((S)-5-(3-(tert-butoxy)-3-oxopropyl)-2-oxo-3-phenylimidazolidin-1-yl)pyridin-2-yl)-3-(6-((S)-4-isobutyl-3-((4-nitrophenyl)sulfonyl)-2-oxoimidazolidin-1-yl)pyridin-2-yl)-2-oxoimidazolidin-4-yl)propanoate 52

^{1}H, 500 MHz, $CDCl_3$

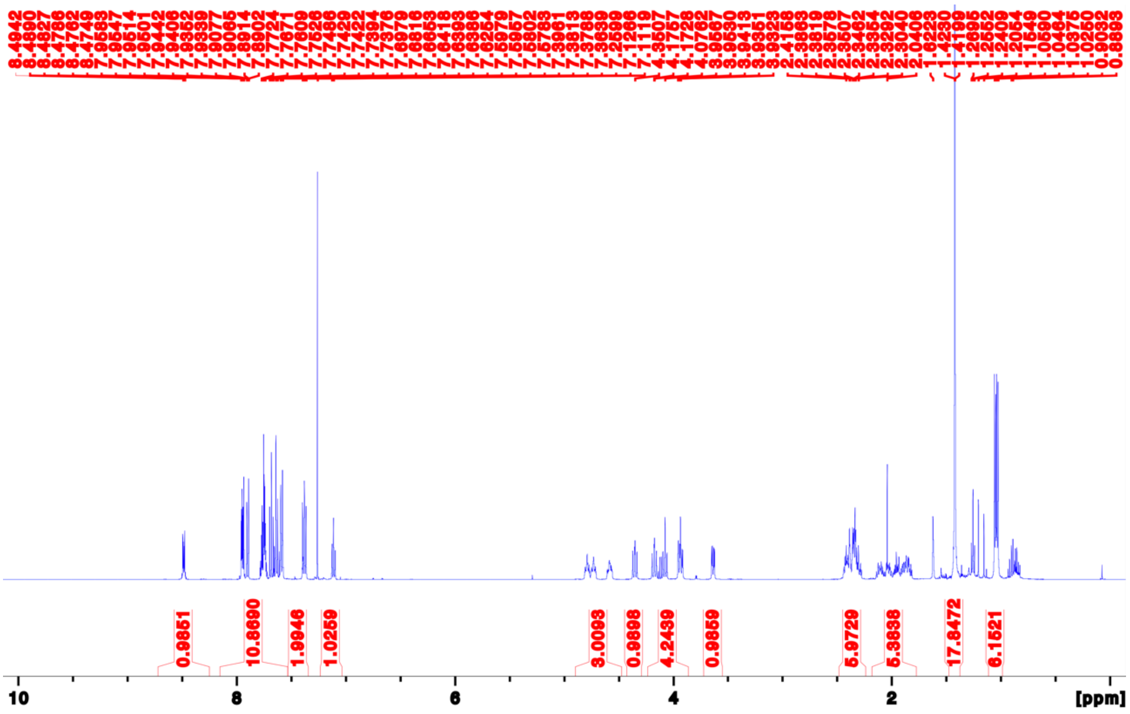

^{13}C, 125 MHz, $CDCl_3$

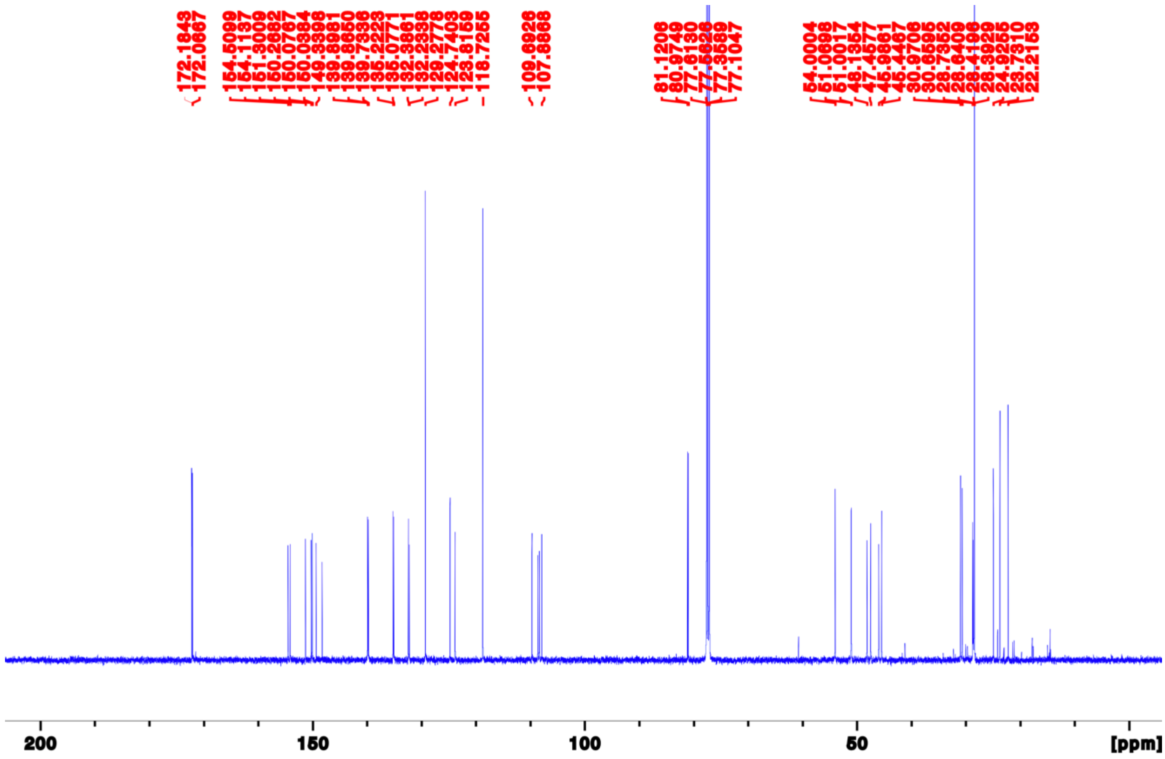

3-((S)-1-(6-((S)-5-(2-Carboxyethyl)-2-oxo-3-phenylimidazolidin-1-yl)pyridin-2-yl)-3-(6-((S)-4-isobutyl-3-((2-nitrophenyl)sulfonyl)-2-oxoimidazolidin-1-yl)pyridin-2-yl)-2-oxoimidazolidin-4-yl)propanoic acid 40

^{1}H, 400 MHz, CD_3OD

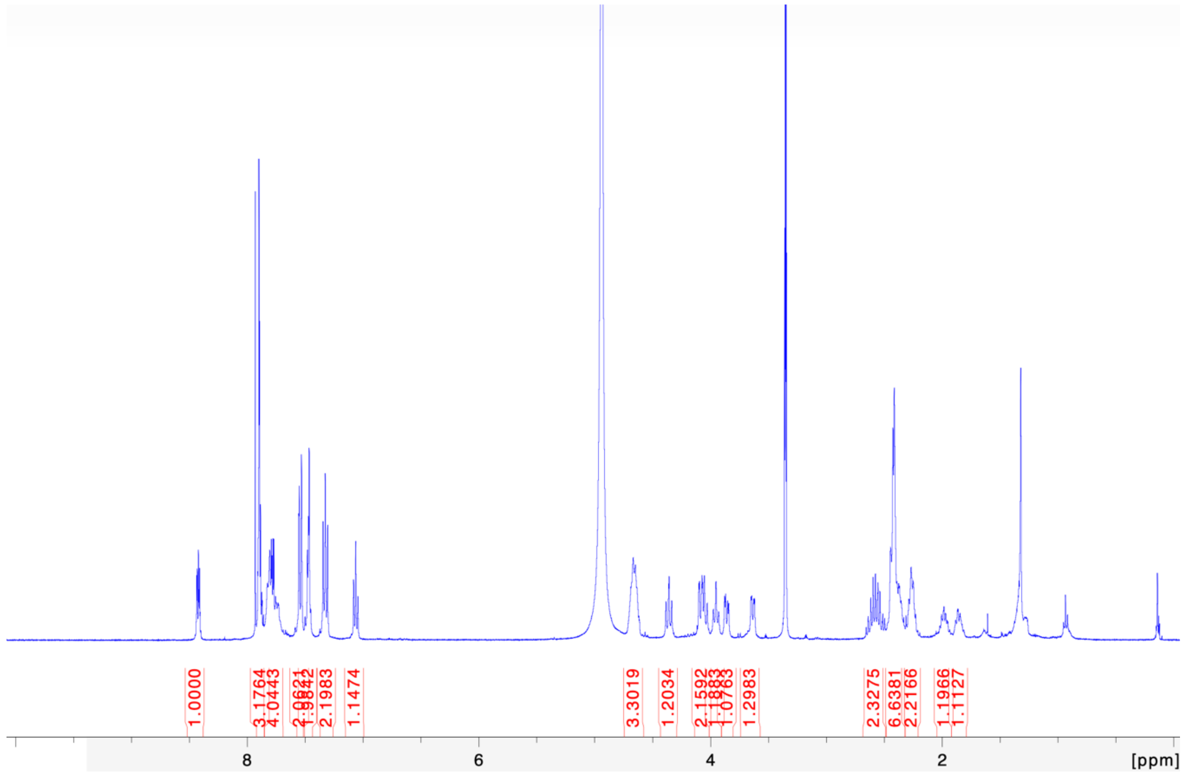

^{13}C, 125 MHz, CD_3OD

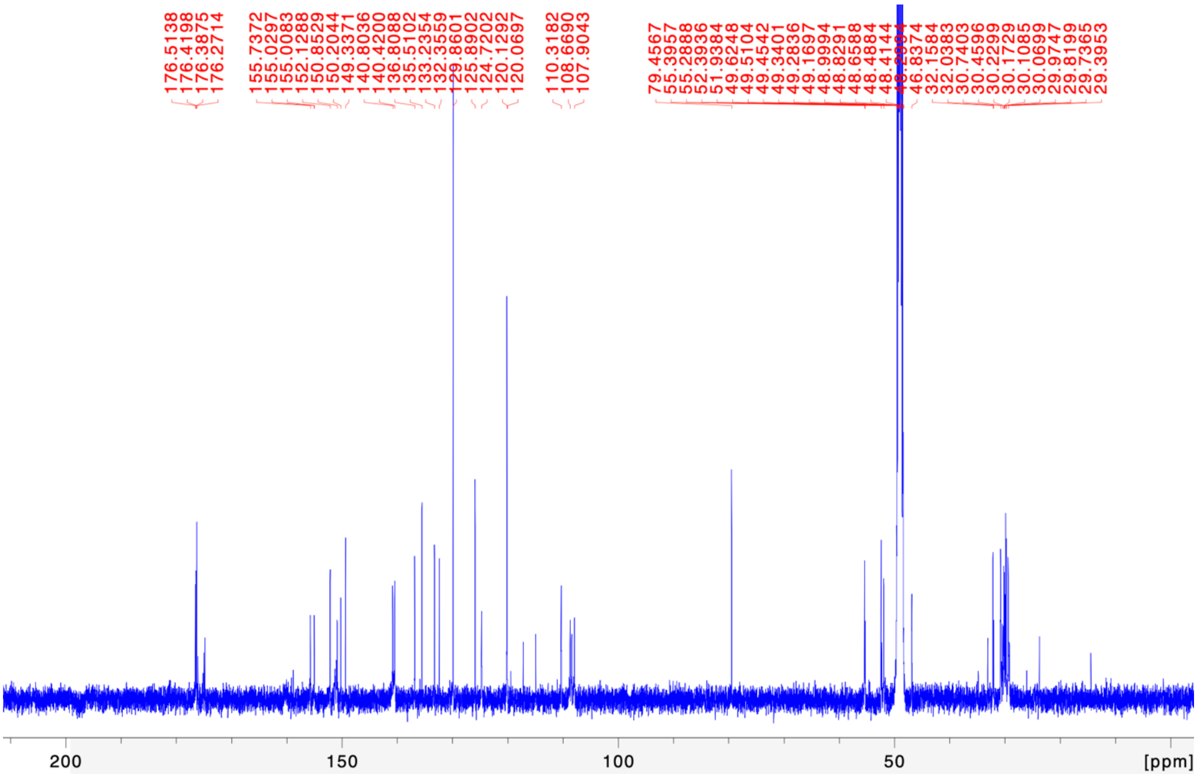
176.5138
176.4198
176.3875
176.2714
155.7372
155.0297
155.0083
152.1288
150.8529
150.2044
149.3371
140.8036
140.4200
136.8088
135.5102
133.2354
132.3559
129.8601
125.8902
124.7202
120.1292
120.0697
110.3182
108.6690
107.9043
79.4567
55.3957
55.2888
52.3936
51.9384
49.6248
49.5104
49.4542
49.3401
49.2836
49.1697
48.9994
48.8291
48.6588
48.4884
48.4144
46.8374
32.1584
32.0383
30.7403
30.4596
30.2299
30.1729
30.1085
30.0699
29.9747
29.8199
29.7365
29.3953
200
150
100
50
[ppm]

3. Solution-phase Conformational Analysis

Deprotected Lys-Lys-Lys trimer 19

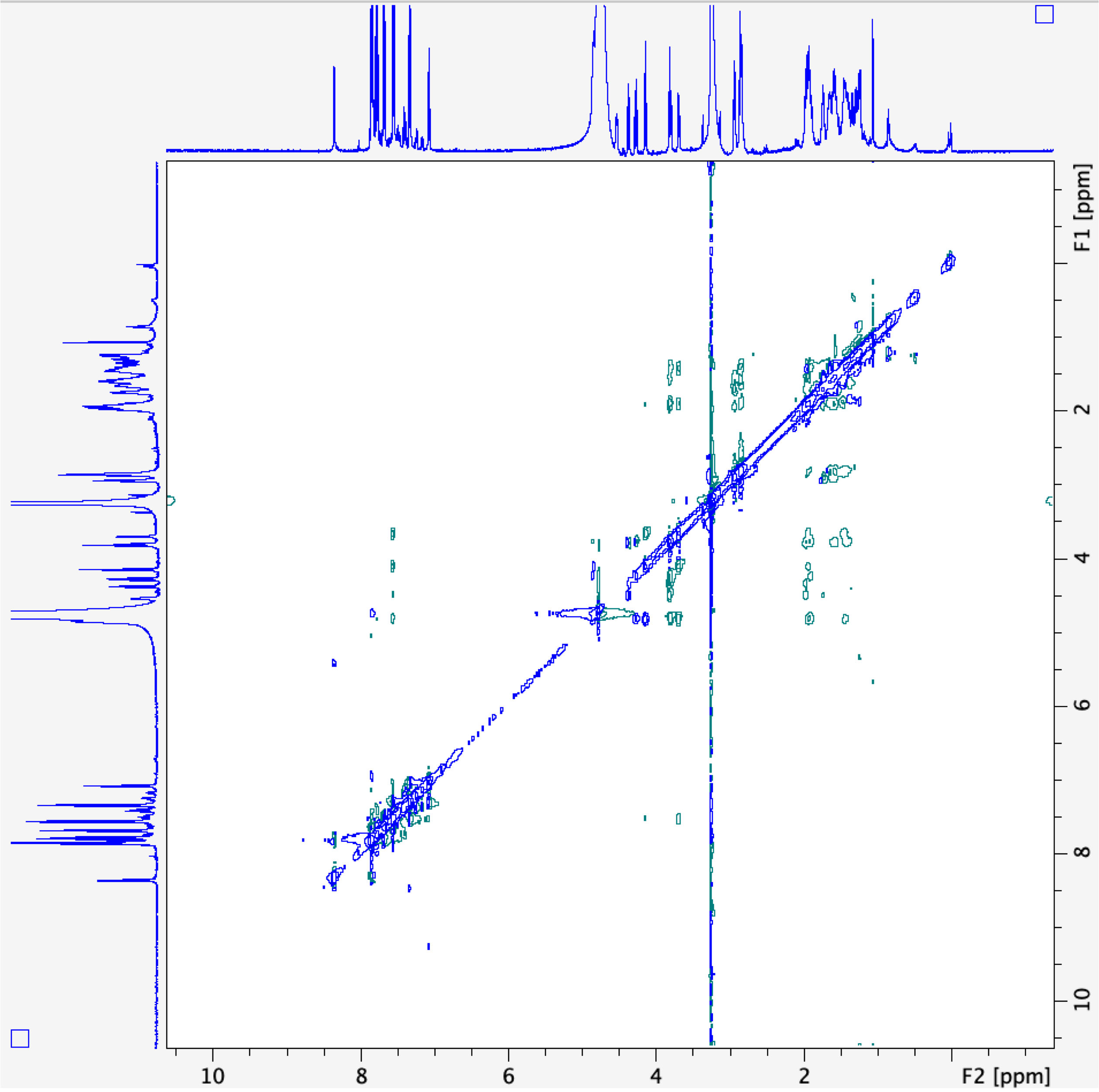

ROESY 600 MHz, spin lock = 400 ms, methanol-d_4, 298 K (above)

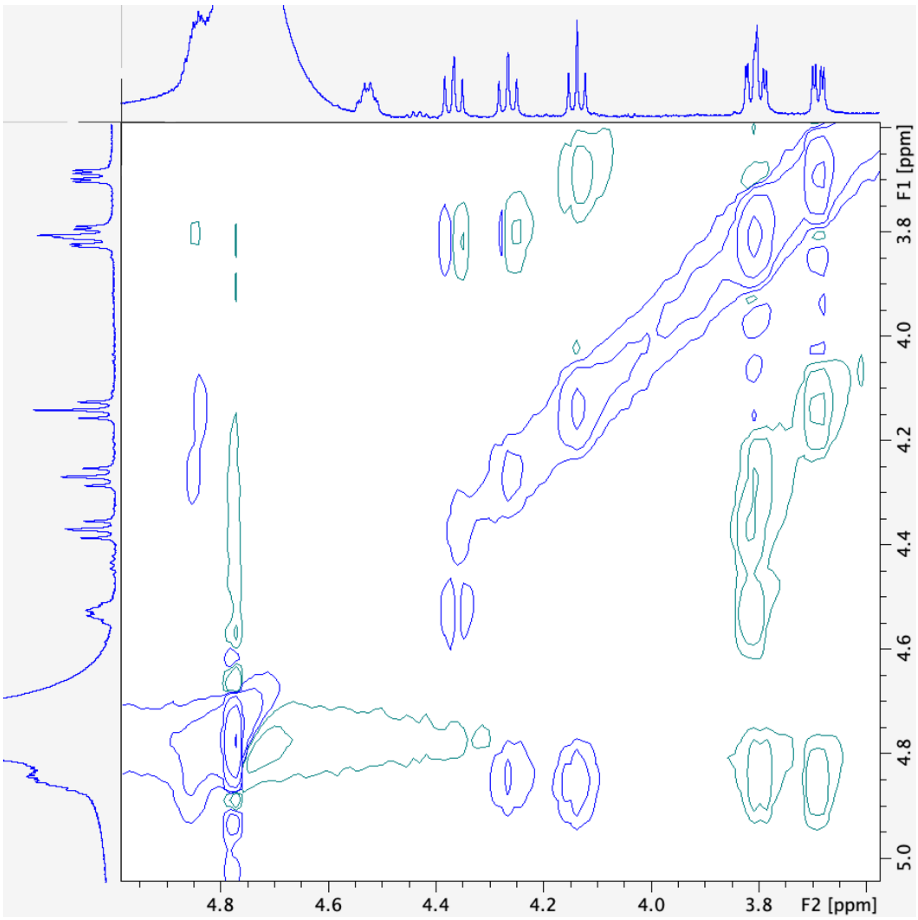

ROESY: Selected aliphatic/aliphatic region (above)

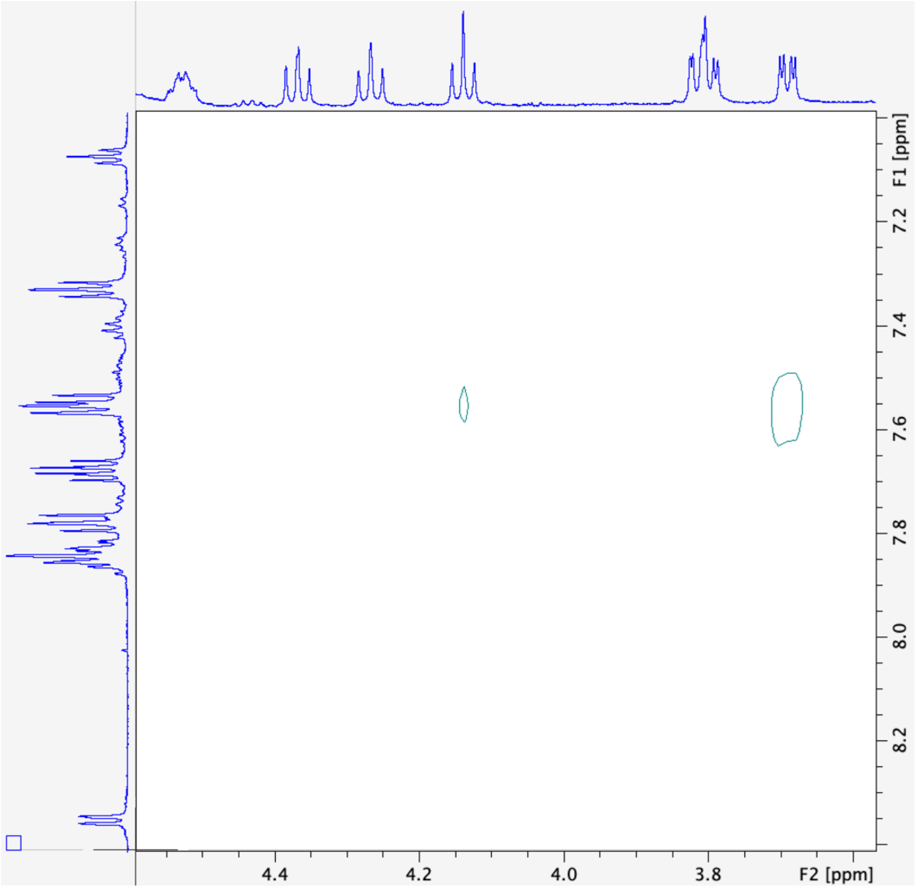

ROESY: Selected aliphatic/aromatic region (above)

H18 and H7 are not well resolved in the ^1H spectrum and are partially obscured by the solvent associated D_2O peak. However, from careful inspection of the COSY, H18 and H7 can be distinguished – allowing identification of their cross-peaks in the ROESY.

(a) Lys-Lys-Lys trimer 19 (in methanol-d_4): COSY

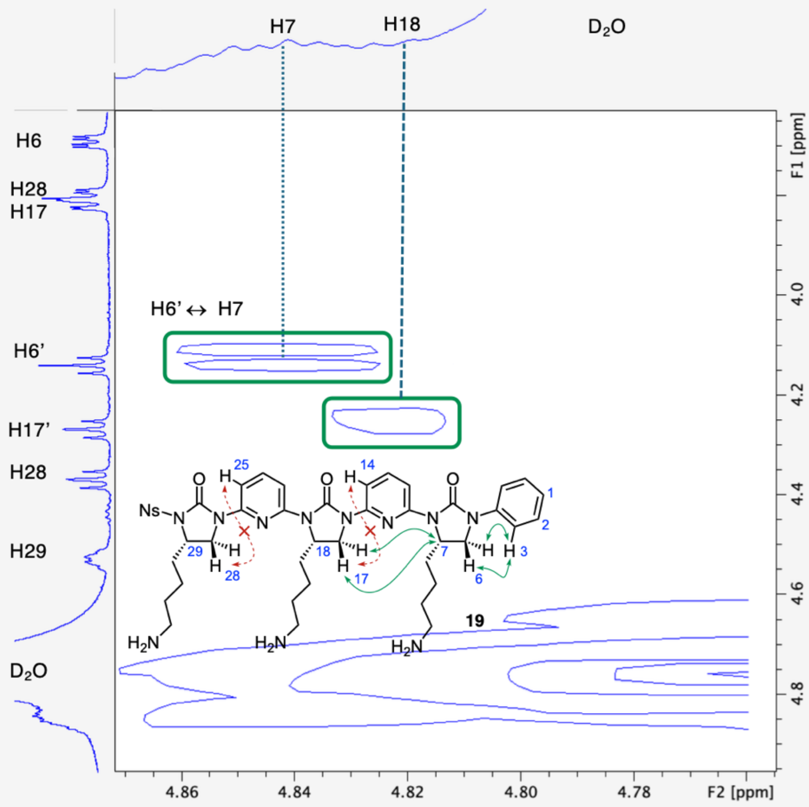

(b) Lys-Lys-Lys trimer 19 (in methanol-d_4): ROESY

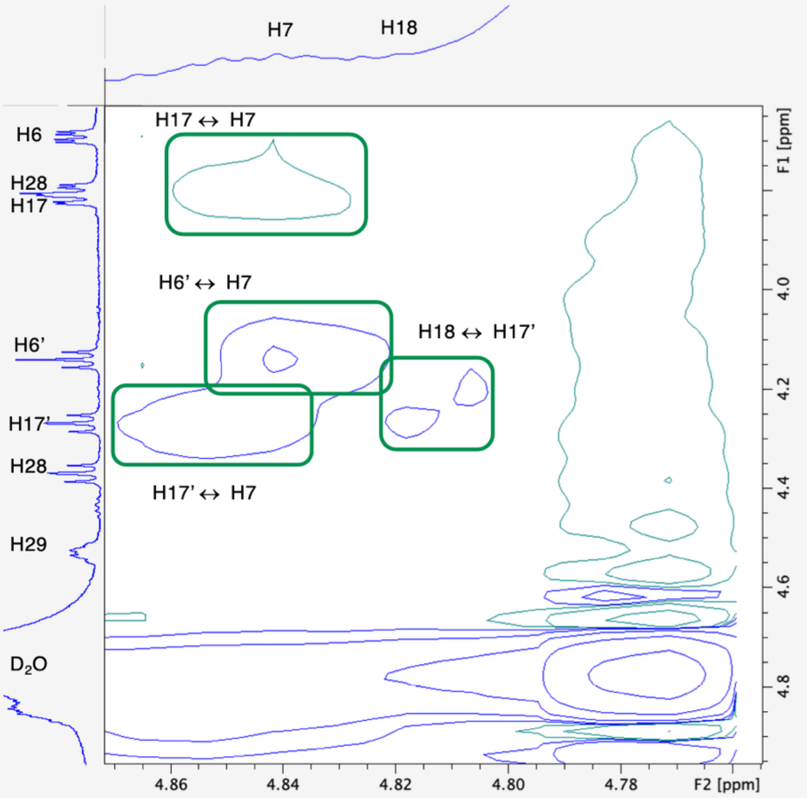

Leu-Glu-Glu trimer 40

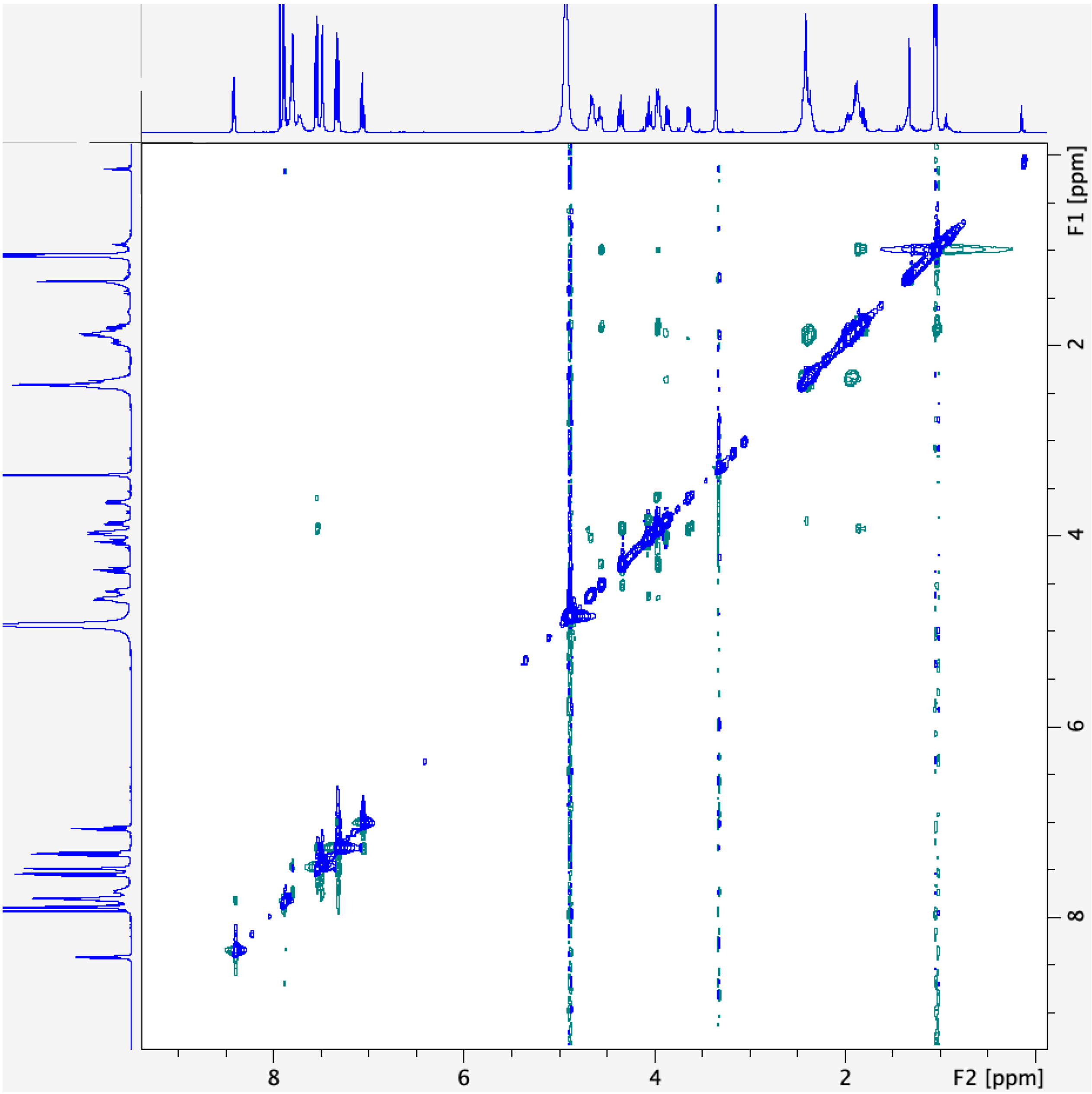

ROESY 500 MHz, spin lock = 400 ms, methanol-d_4, 298 K

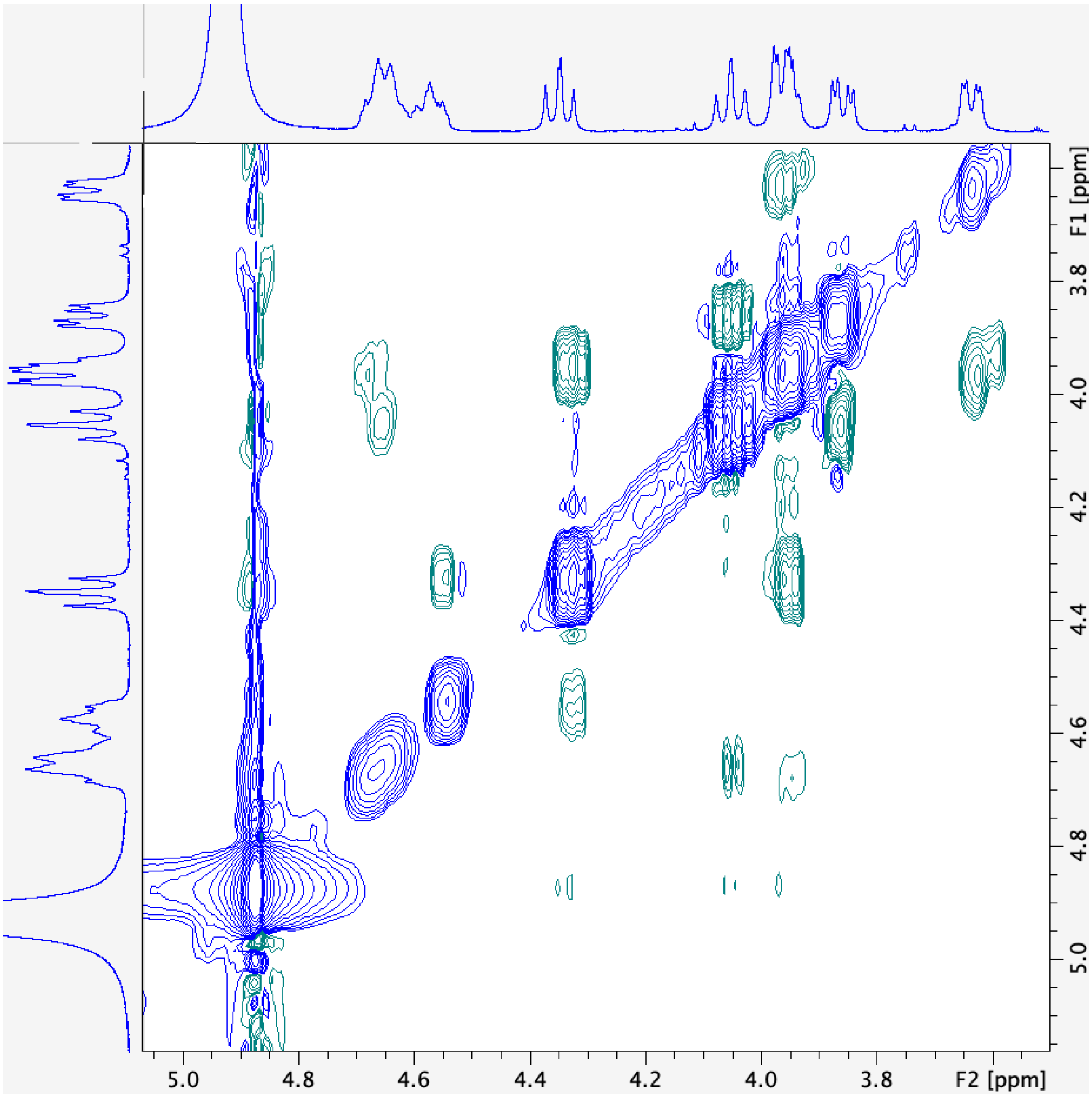

ROESY: Selected aliphatic/aliphatic region (above)

Glu-Glu dimer 41

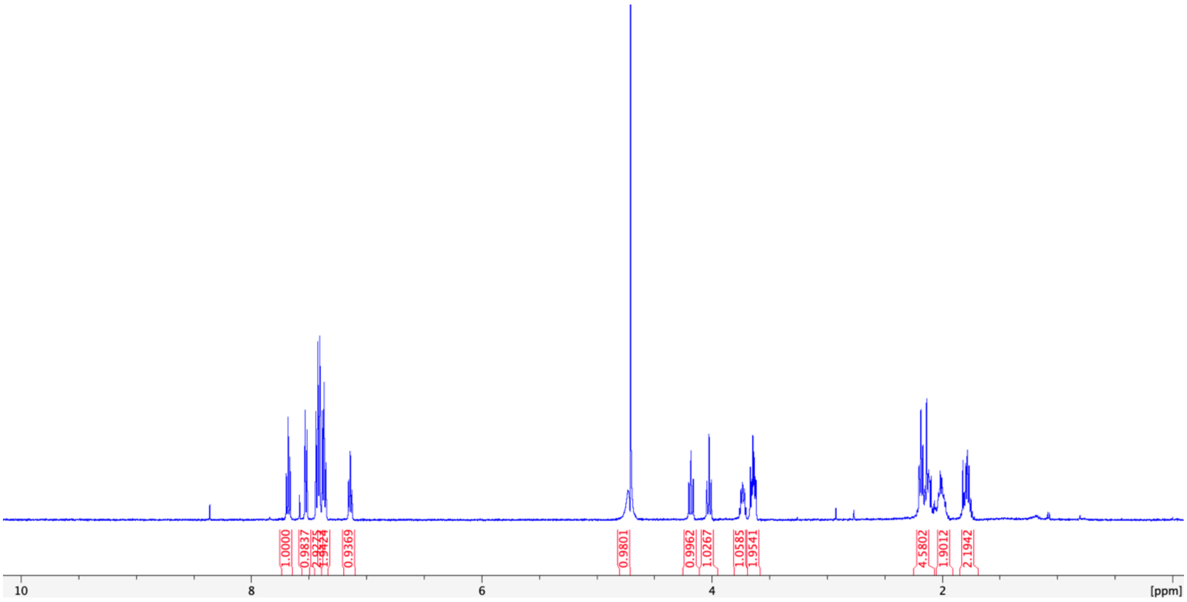

^{1}H 500 MHz, pH 7.4 phosphate buffer, 298 K (above)

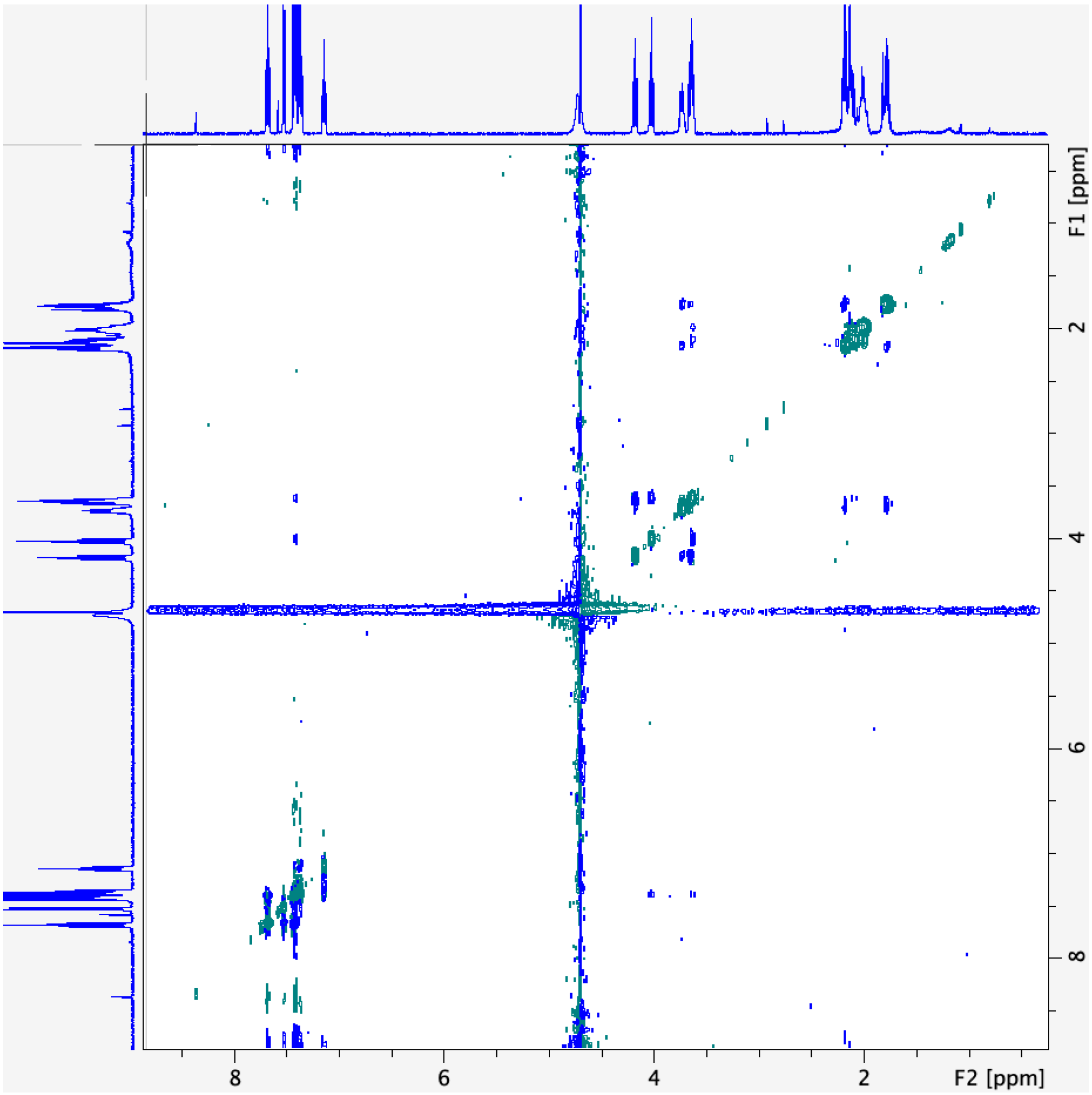

NOESY 500 MHz, mixing time = 800 ms, pH 7.4 phosphate buffer, 298 K (above)

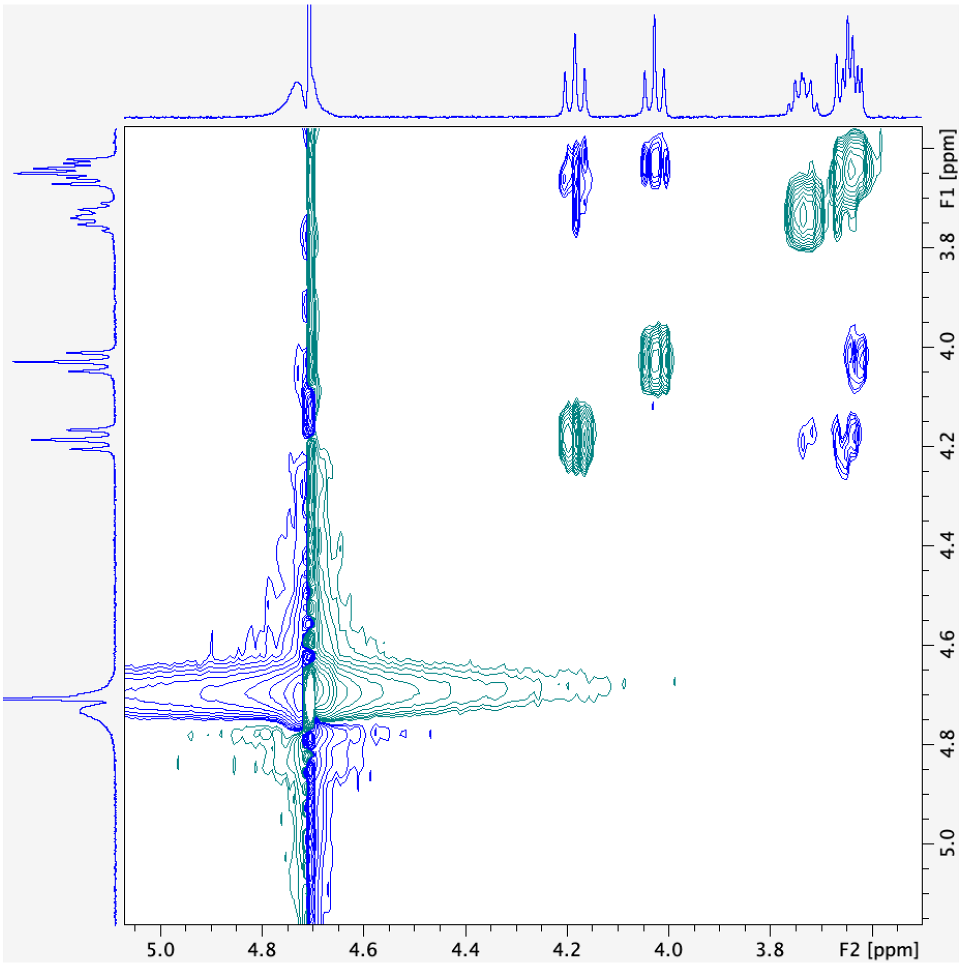

NOESY: Selected aliphatic/aliphatic region (above)

Glu-Glu-Glu trimer 39

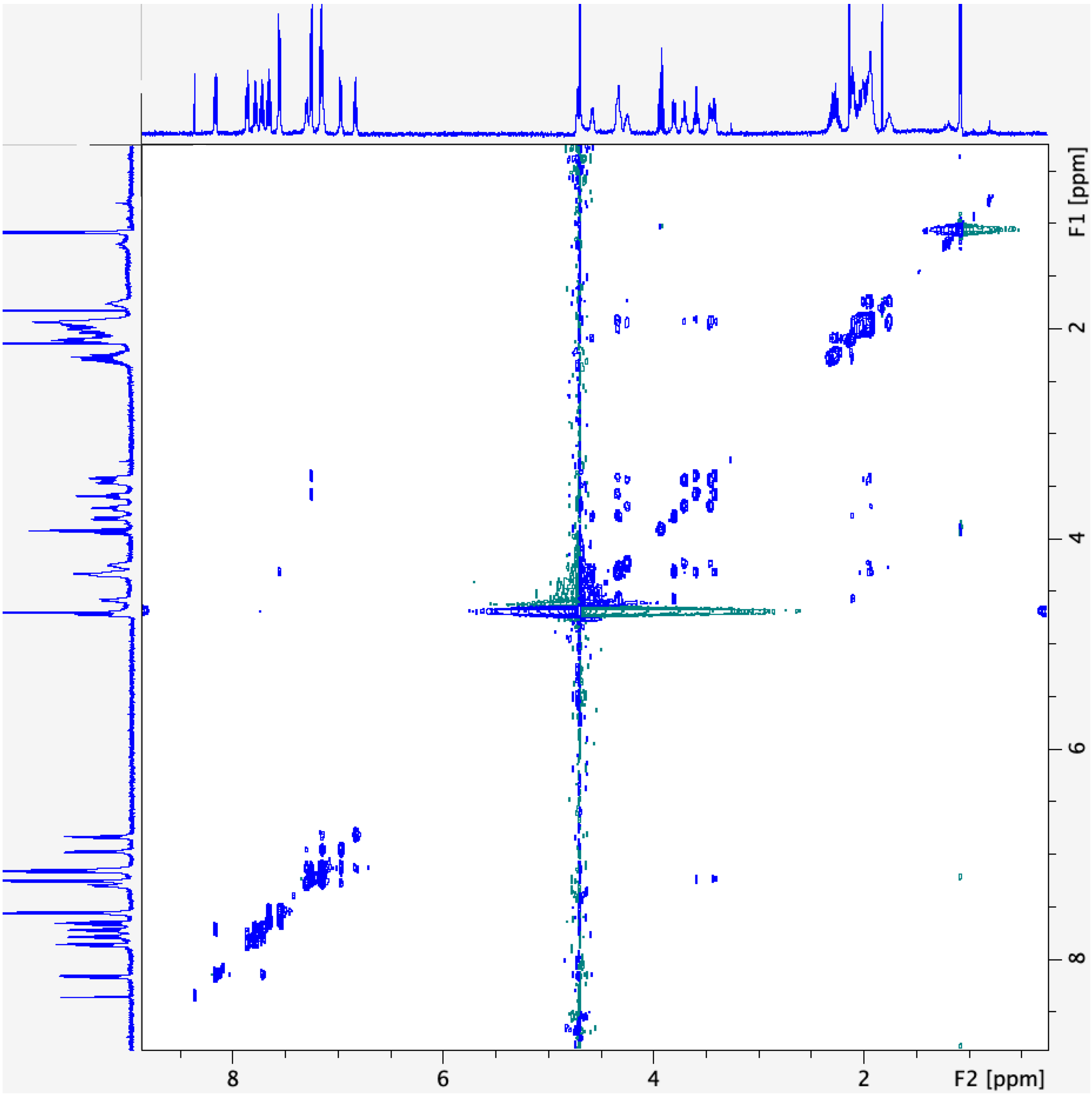

NOESY 500 MHz, mixing time = 800 ms, pH 7.4 phosphate buffer, 298 K (above)

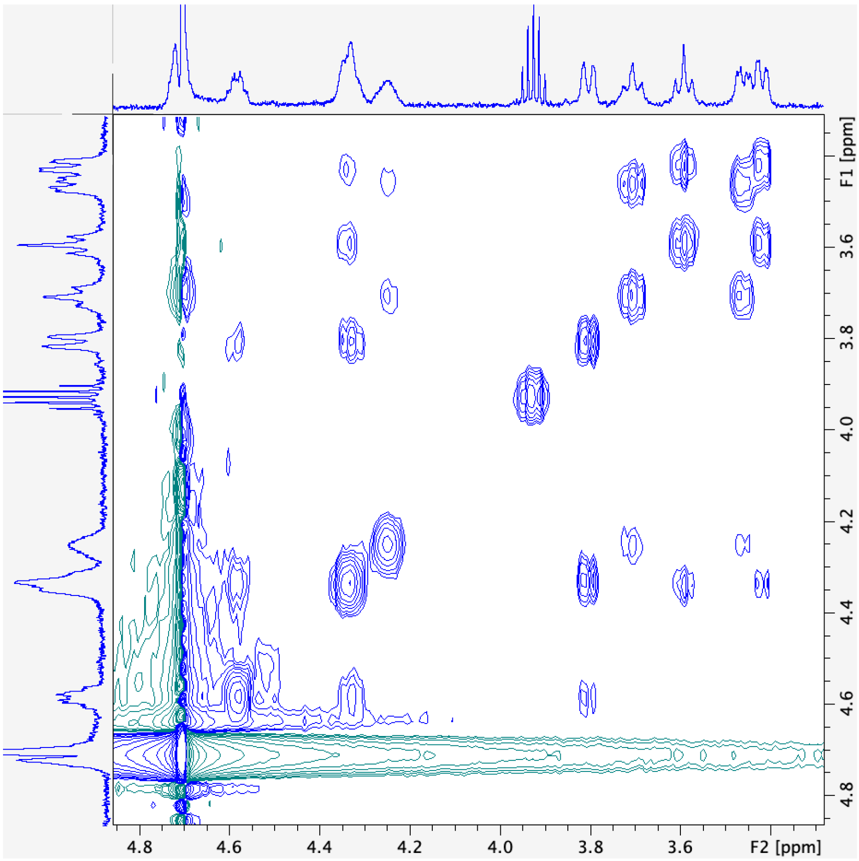

NOESY: Selected aliphatic/aliphatic region (above)

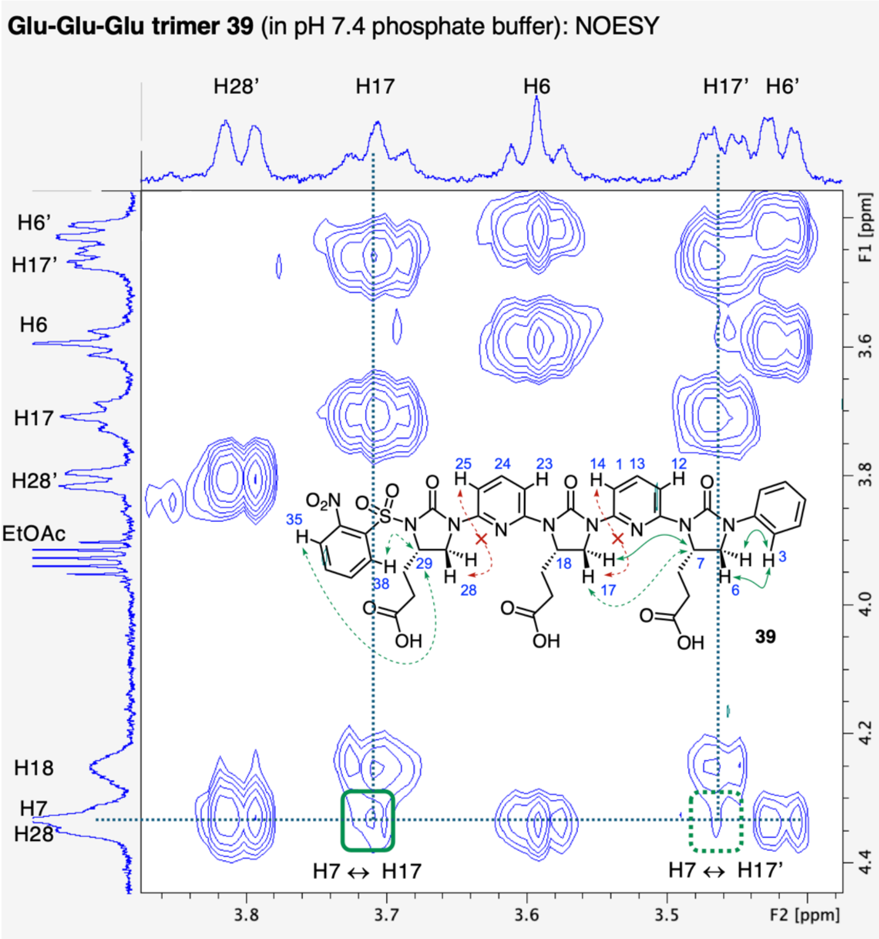

4. X-Ray Crystallography

4.1 Experimental

A suitable crystal was selected and mounted on a MITIGEN holder in oil on a Rigaku FRE+ diffractometer with Arc)Sec VHF (**28**, **33**) Varimax confocal mirrors, a UG2 goniometer and HyPix 6000HE detector or a Rigaku 007HF diffractometer with Arc)Sec VHF Varimax confocal mirrors, a UG2 goniometer and HyPix Arc-100 detector (**29**). The crystals were kept at a steady T = 100(2) K during data collection.

Data were measured using ω scans with Mo K_{α} (**28**, **33**) or Cu K_{α} (**29**) radiation. The diffraction patterns were indexed and the total number of runs and images were based on the strategy calculations from the program CrysAlisPro.[5,6]

Data reduction, scaling and a multi-scan absorption correction were performed using CrysAlisPro[5,6] empirical absorption correction using spherical harmonics, implemented in the SCALE3 ABSPACK scaling algorithm.

The structures were solved with the ShelXT 2014/5[7] (**28**) or 2018/2[7] (**29**,**33**) solution programs using dual methods and by using Olex2 1.5[8] as the graphical interface. The models were refined with ShelXL 2016/6[9] (**28**), 2018/3[9] (**29**) or olex2.refine 1.5alpha[10] using full matrix least squares minimisation on $|F|^2$. All non-hydrogen atoms were refined anisotropically. Hydrogen atom positions were calculated geometrically and refined using the riding model, except for **29** and **33** where H-atoms bonded to heteroatoms were initially located from the difference map and then refined using the riding model.

4.2 Results

**Identifier**	**28**	**29**	**33**
Compound	(*S*)-*N*-(4-Methyl-1-(phenylamino)pentan-2-yl)-4-nitrobenzenesulfonamide	*tert*-Butyl (*S*)-3-(2-oxo-1-phenylimidazolidin-4-yl)propanoate	*tert*-butyl (*S*)-3-(3-((2-nitrophenyl)sulfonyl)-2-oxoimidazolidin-4-yl)propanoate
Formula	$C_{18}H_{23}N_3O_4S$	$C_{16}H_{22}N_2O_3$	$C_{16}H_{21}N_3O_7S$
$D_{calc.}$/ g cm^{-3}	1.340	1.252	1.391
μ/mm^{-1}	0.201	0.704	0.213
Formula Weight	377.45	290.35	399.427
Colour	clear yellow	colourless	colourless
Shape	prism	plate	(cut) block
Size/mm^3	0.32×0.17×0.14	0.30×0.08×0.01	0.17×0.12×0.10
T/K	100(2)	100.00(10)	100.00(10)
Crystal System	monoclinic	monoclinic	orthorhombic
Flack Parameter	-0.03(2)	0.00(11)	-0.005(6)
Hooft Parameter	-0.013(15)	-0.04(3)	-0.005(6)
Space Group	$P2_1$	$P2_1$	$P2_12_12_1$
a/Å	9.79770(10)	5.58530(10)	6.67596(5)
b/Å	9.89090(10)	5.62830(10)	11.07572(8)
c/Å	10.4105(2)	24.5275(5)	25.80300(18)
a/°	90	90	90
b/°	111.955(2)	92.137(2)	90
g/°	90	90	90
V/Å^3	935.70(3)	770.50(3)	1907.90(2)
Z	2	2	4
Z'	1	1	1
Wavelength/Å	0.71073	1.54178	0.71075
Radiation type	Mo K_α	Cu K_α	Mo K_α
Θ_{min}/°	3.971	3.606	2.00
Θ_{max}/°	28.995	70.355	35.46
Measured Refl's.	99571	13116	153760
Indep't Refl's	4971	2895	8489
Refl's I≥2 σ(I)	4836	2870	8309
R_{int}	0.0979	0.0241	0.0348
Parameters	245	196	250
Restraints	1	1	0
Largest Peak	0.254	0.146	0.3735
Deepest Hole	-0.271	-0.213	-0.2144
GooF	1.055	1.056	1.0739
wR_2 (all data)	0.0692	0.0812	0.0594
wR_2	0.0686	0.0810	0.0589
R_1 (all data)	0.0266	0.0290	0.0220
R_1	0.0258	0.0289	0.0212
CCDC Dep. No.	2221030	2546212	2546213

4.3 Molecular Structures

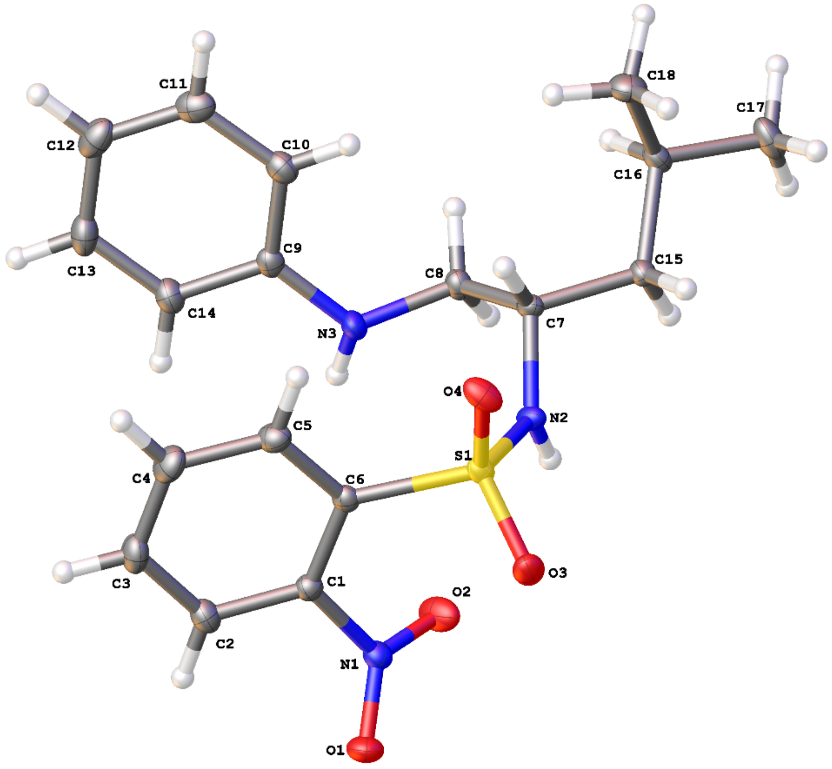

Figure S4.3.1. molecular structure of **28**, ADP ellipsoids at 50% probability

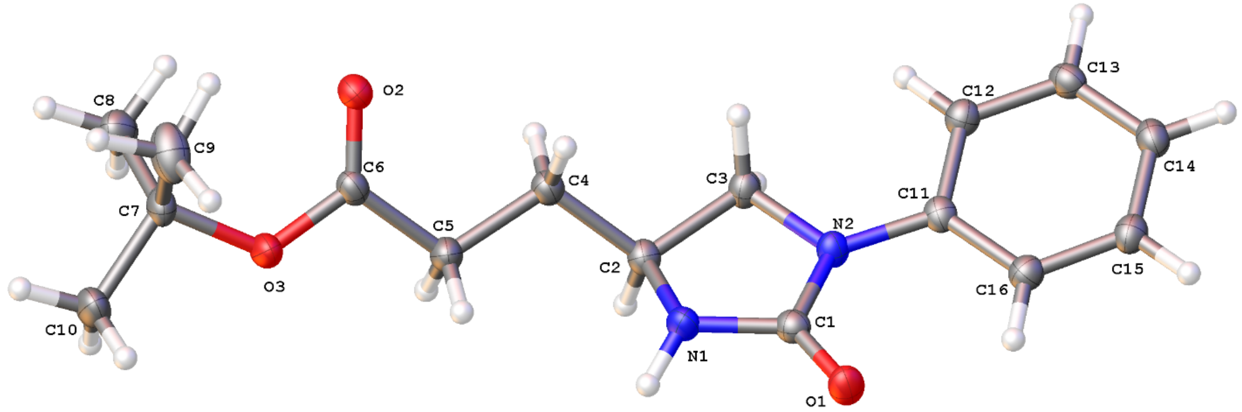

Figure S4.3.2. molecular structure of **29**, ADP ellipsoids at 50% probability

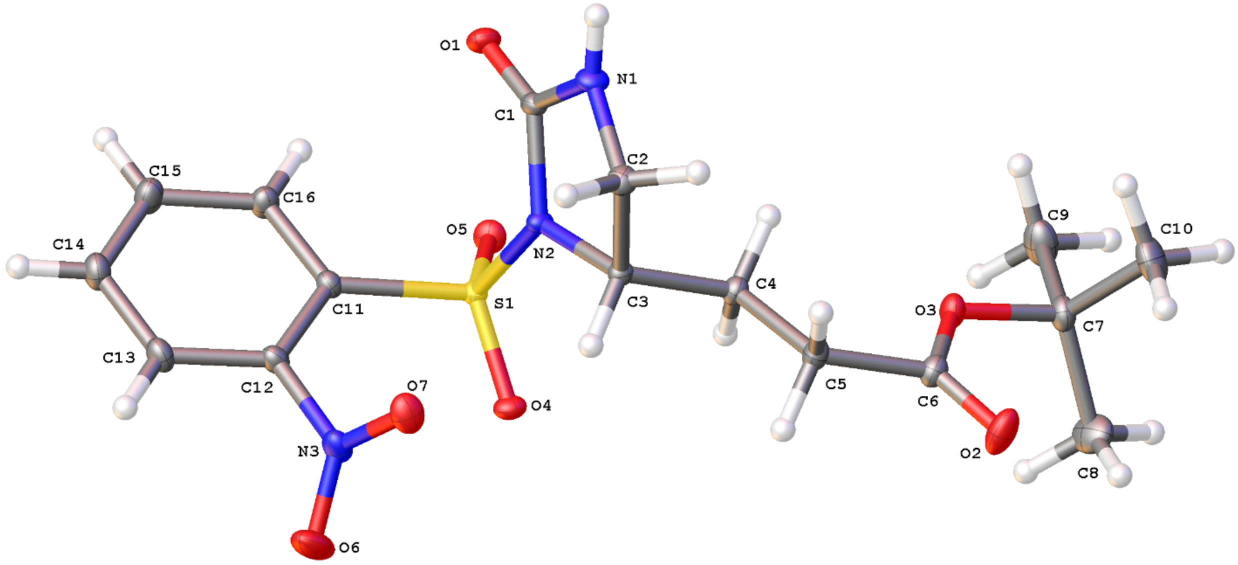

Figure S4.3.3. molecular structure of **33**, ADP ellipsoids at 50% probability

5. References

1 J. E. Aaseng and O. R. Gautun, Synthesis of substituted (*S*)-2-aminotetralins via ring-opening of aziridines prepared from l-aspartic acid β-*tert*-butyl ester, *Tetrahedron*, 2010, **66**, 8982–8991.

2 I. C. Stewart, C. C. Lee, R. G. Bergman and F. D. Toste, Living Ring-Opening Polymerization of N-Sulfonylaziridines: Synthesis of High Molecular Weight Linear Polyamines, *J. Am. Chem. Soc.*, 2005, **127**, 17616–17617.

3 P. C. Knipe, S. Thompson and A. D. Hamilton, Acid-mediated topological control in a functionalized foldamer, *Chem. Commun.*, 2016, **52**, 6521–6524.

4 T. Yamashita, P. C. Knipe, N. Busschaert, S. Thompson and A. D. Hamilton, A Modular Synthesis of Conformationally Preorganised Extended β-Strand Peptidomimetics, *Chem. Eur. J.*, 2015, **21**, 14699–14702.

5 In *CrysAlisPro Software System v1.171.42.72a, Rigaku Oxford Diffraction (2022)*.

6 In *CrysAlisPro Software System v1.171.42.93a, Rigaku Oxford Diffraction (2023)*.

7 G. M. Sheldrick, SHELXT – Integrated space-group and crystal-structure determination, *Acta Cryst.*, 2015, **A71**, 3–8.

8 O. V. Dolomanov, L. J. Bourhis, R. J. Gildea, J. a. K. Howard and H. Puschmann, OLEX2: a complete structure solution, refinement and analysis program, *J. Appl. Cryst.*, 2009, **42**, 339–341.

9 G. M. Sheldrick, Crystal structure refinement with *SHELXL*, *Acta Cryst.*, 2015, **C71**, 3–8.

10 L. J. Bourhis, O. V. Dolomanov, R. J. Gildea, J. a. K. Howard and H. Puschmann, The anatomy of a comprehensive constrained, restrained refinement program for the modern computing environment – Olex2 dissected, *Acta Cryst.*, 2015, **A71**, 59–75.
